# Quantifying demographic and socioeconomic transitions for computational epidemiology: an open-source modeling approach applied to India

**DOI:** 10.1186/s12963-015-0053-1

**Published:** 2015-08-01

**Authors:** Sanjay Basu, Jeremy D. Goldhaber-Fiebert

**Affiliations:** Stanford Prevention Research Center and Center on Poverty and Inequality Stanford University, Stanford, CA USA; Stanford Health Policy, Centers for Health Policy and Primary Care and Outcomes Research Stanford University, Stanford, CA USA

**Keywords:** Mathematical model, India, Demography, Socioeconomic factors, Population health

## Abstract

**Background:**

Demographic and socioeconomic changes such as increasing urbanization, migration, and female education shape population health in many low- and middle-income countries. These changes are rarely reflected in computational epidemiological models, which are commonly used to understand population health trends and evaluate policy interventions. Our goal was to create a “backbone” simulation modeling approach to allow computational epidemiologists to explicitly reflect changing demographic and socioeconomic conditions in population health models.

**Methods:**

We developed, evaluated, and “open-sourced” a generalized approach to incorporate longitudinal, commonly available demographic and socioeconomic data into epidemiological simulations, illustrating the feasibility and utility of our approach with data from India. We constructed a series of nested microsimulations of increasing complexity, calibrating each model to longitudinal sociodemographic and vital registration data. We then selected the model that was most consistent with the data (i.e., greater accuracy) while containing the fewest parameters (i.e., greater parsimony). We validated the selected model against additional data sources not used for calibration.

**Results:**

We found that standard computational epidemiology models that do not incorporate demographic and socioeconomic trends quickly diverged from past mortality and population size estimates, while our approach remained consistent with observed data over decadal time courses. Our approach additionally enabled the examination of complex relations between demographic, socioeconomic and health parameters, such as the relationship between changes in educational attainment or urbanization and changes in fertility, mortality, and migration rates.

**Conclusions:**

Incorporating demographic and socioeconomic trends in computational epidemiology is feasible through the “open source” approach, and could critically alter population health projections and model-based evaluations of health policy interventions in unintuitive ways.

**Electronic supplementary material:**

The online version of this article (doi:10.1186/s12963-015-0053-1) contains supplementary material, which is available to authorized users.

## Background

To understand and address population health trends and to evaluate potential health policy interventions, mathematical simulation models are commonly used—relating individual-level risk factor exposures and interventions to population-level health outcomes. Demographic and socioeconomic conditions that shape population health are changing rapidly in many low- and middle-income countries. These changes are challenging to incorporate into models, as they affect population health in complex ways. For example, rapid urbanization may have both positive and negative effects on population health. Urbanization can increase access to skilled medical care, and potentially facilitate higher household earnings that correlate with improved health outcomes [1]. However, rural migrants to urban areas often encounter increased exposure to environmental pollution, slum living, and disease risks stemming from unhealthy diets [2–4]. Large developing countries are shifting from being majority rural to mostly urban by 2050, highlighting the pressing importance of understanding the health effects of complex socioeconomic transitions [5]. In addition to urbanization, other complex socioeconomic transitions include the increase in age-associated disability and chronic diseases [6, 7]. Educational attainment and literacy levels are also increasing [8, 9], and accompany lower fertility, higher female labor force participation and associated complex changes in maternal and child health outcomes [10].

Modeling the complex interactions of demographic and socioeconomic conditions requires accounting for simultaneous, interacting exposures experienced by individuals over their lifetimes [11]. In the past, numerous high-quality health policy models implicitly assumed that current exposures to demographic and socioeconomic conditions would remain the same in the future [12–15]. This assumption is understandable, given the challenges of estimating parameters to describe how different birth cohorts experience varying exposures; however, such factors may influence the accuracy of projections made by these models.

The goal of our study is to bridge the gap between available data on demographic and socioeconomic changes in low- and middle-income countries and simulation models of population health and health policy. Our specific aim in this paper is to create, validate, and “open source” a simulation modeling approach that allows population health modelers to explicitly reflect changing demographic and socioeconomic conditions. Our approach is intended to be intuitive and simple enough to be easily incorporated into health policy simulations, but also faithful to available data. We illustrate it using India’s demographic and socioeconomic data on trends in fertility, all-cause mortality, education, and migration, using the types of datasets available from many developing countries [16]. To facilitate replication and extension of our approach, we provide our complete data and model code (see Additional File 1 and Tables AF1-AF5).

## Methods

### Overview

We developed the Stanford Project for Open Knowledge in Epidemiology in India model (SPOKE-I). An overview of the process used to develop the model is as follows: First, we constructed a series of nested simulation models of increasing complexity. Second, using multiple empirical data sources detailed below and itemized in Table 1, we produced a set of targets for modeled outcomes to match. Third, using a Bayesian approach [17], we calibrated each model’s simultaneous fit to these empirical targets. Fourth, we selected the simulation model that was most consistent with the empirical data (i.e., greater accuracy) while penalizing models with larger numbers of parameters (i.e., favoring parsimony over complexity) [18]. Fifth, we assessed the validity of the selected model’s projections against independent data not used for calibration—specifically, life expectancy estimates from the World Bank, which used independent surveys for estimation [19]. Sixth, we evaluated the importance of incorporating demographic and socioeconomic trends in the selected model by comparing the model’s projections to that of a traditional static population health model, comparing the two against both historical data and independent projections of population size and life expectancy [6, 19].

**Table 1:** Data used for model calibration and parameter estimation^a^

Parameter	Data sources	Data details
Fertility	National Family Health Survey waves 1–3 (NFHS): 1992–3, 1998–9, 2005–6) [24–26]	Number of total children ever born to mother, by maternal age and urban/rural residence, Additional file 1: Table AF1
Mortality	Sample Registration System (SRS): 1972–2008 [23]	Probability of death by calendar year, age, sex, and urban/rural residence, Additional file 1: Table AF2
Educational attainment	NFHS 1–3 (1992–3, 1998–9, 2005–6) and District-Level Household Survey wave 3 (DLHS-3): 2007–8 [24–27]	Prevalence of no education, primary school, secondary school, and greater than secondary school education, by age, sex, and urban/rural residence, Additional file 1: Table AF3
Migration	NFHS 2 and 3 (1998–9, 2005–6) [25, 26]	Proportion of women who had migrated from urban to rural areas or vice versa within the last 6 years, 12 years, and ever in their lifetime, stratified by age, sex, and urban/rural residence, Additional file 1: Table AF4
Population size	United Nations Population Division, 1992–2010 [6]	Absolute population size by calendar year, stratified by urban/rural residence, Additional file 1: Table AF5

^a^ Full data disaggregated by age, sex, urban/rural residence, and educational attainment status are provided in Additional file 1: SI Tables AF1–S5

**Table 2:** Comparisons of three calibrated models reveal that one incorporating secular trends is more consistent with the observed data^a^

Model	Components	ΔDIC when fit against Table 1 data sources
1	Age, sex, urban/rural residence, fertility, mortality	Reference
2	Age, sex, urban/rural residence, fertility, mortality, educational attainment	+5.2 versus model 1
3	Age, sex, urban/rural residence, fertility, mortality, educational attainment, migration	−259.1 versus model 2

^a^ A model incorporating both education and migration rates best explains the variance in the data, even when penalizing the use of more parameters using the deviance information criterion (DIC). Note that lower DIC scores are considered better (reflecting better fit to data and fewer parameters to accomplish the fitting), and a >10 point difference is considered meaningful [42]

### Model structures

**Fig. 1:**
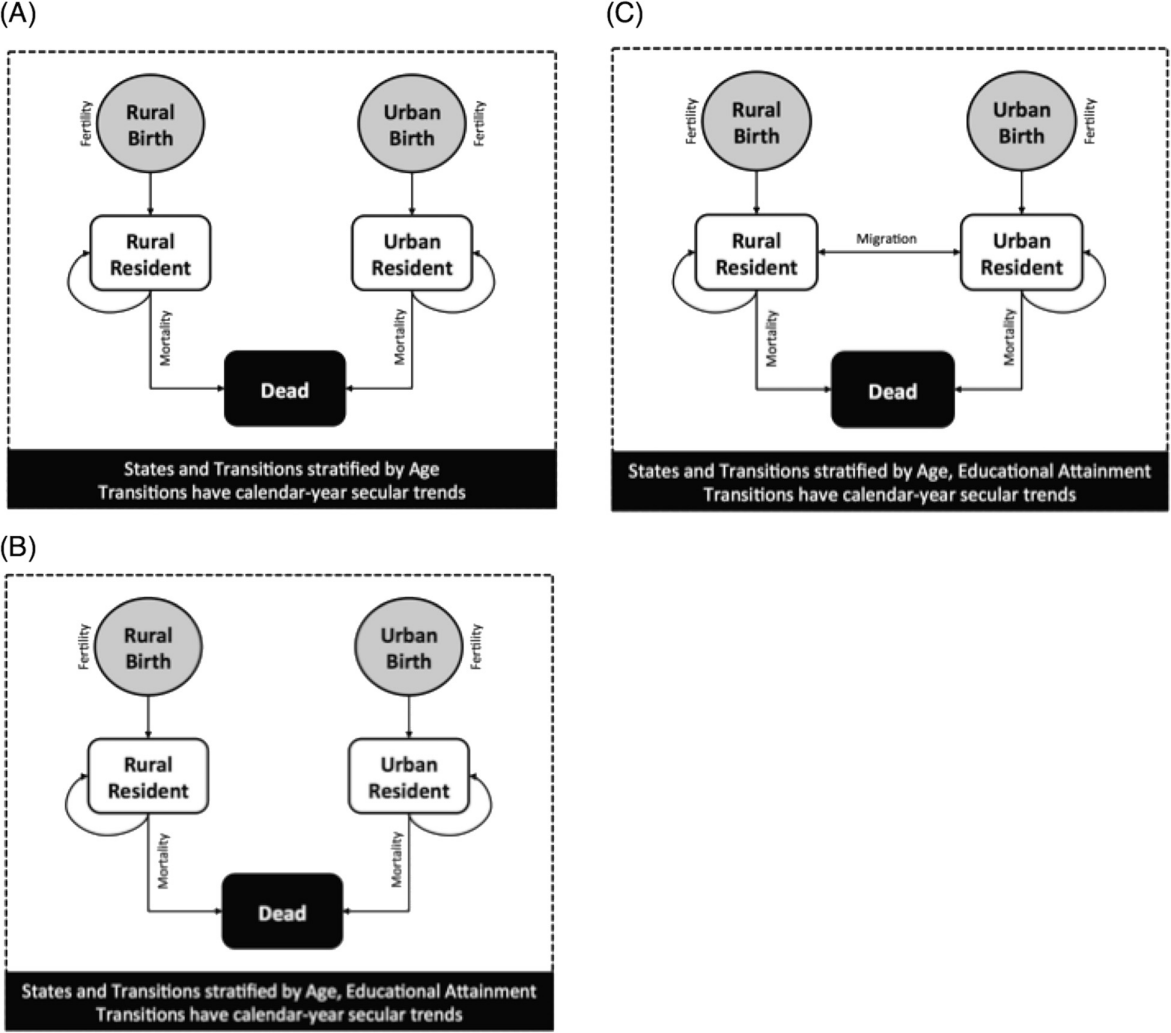
Model diagrams of three population models of increasing complexity. Three models were compared in the study, of increasing complexity. The simplest model (**a**) was stratified by age and urban/rural residence and included only fertility and mortality rates and their secular trends, aggregating populations across all educational attainment categories and ignoring migration. The next model (**b**) included stratification by educational attainment categories (0, 1–5, 6–12, or >12 years of schooling) and secular trends in educational attainment, but ignored migration. The most complex model (**c**) included migration rates and secular trends in migration rates

We modeled historic and future cohorts of Indian females, focusing on fertility, mortality, educational attainment, and urban/rural migration given the strong evidence linking these indicators to population health in India [2, 20, 21]. We designed and constructed three nested models of increasing complexity (Fig. 1). The simplest model was stratified by age and urban/rural residence and included only fertility and mortality rates and their secular trends, aggregating across all educational attainment categories and ignoring migration. The next model included stratification by educational attainment categories (0, 1–5, 6–12, or >12 years of schooling) and secular trends in educational attainment, but ignored migration. The third model also included migration rates and secular trends in migration rates. We implemented the models as stochastic microsimulations in which we simulated a virtual population of multiple birth cohorts of Indian females stratified by age, urban/rural residence, and (if included) educational attainment. These groups were subjected to correspondingly stratified annual fertility, mortality, and migration rates using a standard competing risks approach [22]. Modeled outcomes included population size, total fertility rate, mortality rate, education prevalence, number of migrants for each cohort, and life expectancy from 1992 to 2025 (see Additional file 1: AF1).

### Data sources

#### Overview

We used population-representative data sources (Table 1). These included the United Nations Population Division’s (UNPD) historic estimates and future projections of population size and composition by sex and urban/rural residence (1992–2025) [6], as well as India’s Sample Registration System (SRS) and four large-scale household surveys conducted in India between 1992 and 2008 [23–27]. Household surveys included all available waves of India’s National Family and Health Survey (NFHS-1 [1992–3], NFHS-2 [1998–9], and NFHS-3 [2005–6]), which are part of the Demographic and Health Surveys conducted in over 90 countries every five years [16]. We also used India’s District Level Household and Facility Survey (DLHS-3 [2007–8]) to provide more recent data [27]. All analyses of the household surveys employed sample weights to adjust for non-coverage and non-response, allowing us to calculate nationally-representative estimates.

#### Starting population size and composition

The starting size and composition of India’s female population in 1992 was input into the models based on the UNDP overall female population size and its urban/rural-specific age distribution [6]. The educational attainment category distribution (0, 1–5, 6–12, or >12 years of schooling) for each age and urban/rural specific subgroup was determined from the NFHS-1 [24].

#### Fertility

The NFHS provided data on fertility. Specifically, each NFHS wave provided estimates of the number of children born to a woman stratified by a woman’s age, urban/rural residence, educational attainment category, and calendar year.

#### Urban/rural migration

The NFHS provided information on migration between rural and urban areas in India, specifically the proportion of women who had migrated from rural to urban areas or vice versa within the previous six years, 12 years, and ever in their lifetime, stratified by age, urban/rural residential status, educational attainment category, and calendar year.

#### Mortality

The SRS provided the main data on mortality [23]. Specifically, the SRS life tables for 1972 to 2008 contain estimated death rates stratified by age, sex, urban/rural residence, and calendar year. However, as the SRS life tables are not stratified by educational attainment, we supplemented the SRS data with information derived from the DLHS-3 on mortality stratified by educational attainment from verbal autopsies of household members (further detailed below in the Mortality section of ‘Modeled Processes and Inputs’) [27].

### Modeled processes and inputs

#### Overview

As illustrated in Fig. 1, individual women in our models age deterministically and experience annual risks of fertility, mortality, and (when included in the model) migration. Risks depend on the calendar year of a woman’s birth, her current age, urban/rural residence, and (when included) educational attainment category. Below we summarize these modeled processes (further details in Additional file 1: AF1).

#### Fertility

In the model, births in a given year for women depend on their age, urban/rural status, and (when included) educational attainment category. We use a standard demographic model of fertility (see Additional file 1: AF1), specifically, the Gompertz-Pasupuleti model (G–P) [28] which estimates the cumulative age-specific fertility rate as:

1 $$ G(t)=F\kern0.5em {\left(\frac{1}{2}\right)}^{{\left(\frac{ \log\;(0.95)}{ \log\;(0.05)}\right)}^{\frac{t-a}{b}}},\kern0.5em \mathrm{where}\kern0.5em F>0,\kern0.5em a>0,\kern0.5em \mathrm{and}\kern0.5em b>0 $$

In equation 1, *G(t)* is the cumulative age-specific fertility rate up to the age *t* (i.e., total births per woman of each age in in a given NFHS survey wave). The parameter *F* is the cumulative total fertility rate for women of age *t* in the given survey wave, *a* is the median age of fertility (age of giving birth to half of the total number of children that will ever be born to that woman), and *b* is the length of the age interval during which the fertility level rises from 5 % to 95 % of the cumulative rate. The parameters for the G–P model along with a linear secular trend in fertility were fit to the empirical data as part of our overall calibration procedure, which uses the Markov Chain Monte Carlo (MCMC) estimation approach detailed below. Newborn girls enter the model via this fertility process and are then included as members of new birth cohorts, such that they age and potentially experience fertility themselves in future years. The newborn girls share the same urban/rural residential status as their mothers at the time of their birth and are placed into educational attainment categories based on parameters fit to ensure that the educational attainment of these girls match secular trends in education prevalence when they reach the 20 to 24 year age range (see below under ‘Educational Attainment’).

#### Mortality

The model requires female mortality rates stratified by age, urban/rural status, calendar year, and (when included) educational attainment. However, SRS life tables are not stratified by educational attainment nor do they include future trend projections. To stratify by educational attainment, we applied a standard decomposition from which the mortality rate for a given age, urban/rural status, and calendar year-defined group was decomposed into mortality rates specific to subgroups defined by educational status as well [29]:

2 $$ {\mu}_{\alpha, \rho, y}={\displaystyle {\sum}_{\varepsilon}\left[{p}_{\alpha, \rho, \varepsilon, y}R{R}_{\alpha, \rho, \varepsilon }{\mu}_{\alpha, \rho, \varepsilon =0,y}\right]} $$

In equation 2, *μ* is the female mortality rate, *α* is age, *ρ* is urban/rural residential status, *y* is calendar year, *ε* is educational attainment category (index 0 represents the lowest attainment level, the reference category), *p* is proportion of the overall group within each educational attainment category, and *RR* is relative risk of death in each educational attainment category with respect to the reference category. This equation can be rearranged to solve for the mortality rate in the reference category for each age, urban/rural, and calendar year specific group:

3 $$ {\mu}_{\alpha, \rho, \varepsilon =0,y}={\mu}_{\alpha, \rho, y}/\left[{p}_{\alpha, \rho, \varepsilon =0,y}+\kern0.5em {p}_{\alpha, \rho, \varepsilon =1,y}\kern0.5em R{R}_{\alpha, \rho, \varepsilon =1}+\kern0.5em {p}_{\alpha, \rho, \varepsilon =2,y}R{R}_{\alpha, \rho, \varepsilon =2}+{p}_{\alpha, \rho, \varepsilon =3,y}R{R}_{\alpha, \rho, \varepsilon =3}\right] $$

Estimated the relative risk of death based on educational attainment for a woman of a given age and urban/rural status from the DLHS (see Additional file 1: AF1 for details on estimating the relative risk). The NFHS provided estimates of the proportion *p* of the population in each educational attainment category for each group in the three calendar years of the NFHS waves; we focused on 20 to 24 year-olds in the decompositions, since educational attainment had plateaued by this age.

After decomposing the data into the mortality rate for the reference group (lowest educational attainment level) and the relative risk of death for each higher educational attainment group relative to the reference group, we fit a standard Lee-Carter-type model to the decomposed mortality rates to project future trends in mortality [30]. The model fits three parameters to the log mortality rate: a constant, a parameter multiplied by calendar year, and a parameter multiplied by age. The three parameters were fit to the data for each of three age clusters (<1 year olds, 1–10 year olds, >10 year olds) along with all other model parameters (e.g., those describing fertility and migration) as part of a single Markov Chain Monte Carlo (MCMC) fitting process described below.

#### Educational attainment

Educational attainment in each of the four categories (0, 1–5, 6–12, or >12 years of schooling) was assigned to each newborn girl based on birth year and urban/rural residence, accounting for the linear secular trends in educational attainment by calendar year such that the educational prevalence each year follows the trend among women aged 20 to 24 years old.

#### Urban/rural migration

The NFHS-2 provides counts of how many urban dwellers in 1998 lived in a rural zone in 1992, and conversely how many rural dwellers in 1998 lived in an urban zone in 1992 [25]. The NFHS-3 similarly reveals how many urban dwellers in 2005 lived in a rural zone in 1999 and 1993, and how many rural dwellers in 2005 lived in an urban zone in 1999 and 1993 [26]. We estimated, through the MCMC fitting procedure detailed below, the annual net rate of rural-to-urban migration and the linear trend in this rate to match the NFHS data.

### Model targets, calibration, selection, and validation

We sought a model that accurately and parsimoniously reproduced the observed data on levels and trends in fertility, mortality, urban/rural status, educational attainment and migration, and could help us infer the joint uncertainty distribution around parameter estimates describing these processes to learn about the correspondence between these demographic and socioeconomic factors. Such a model and joint uncertainty distribution can then be used to make future population projections with uncertainty bounds and be incorporated into decision-analytic health policy models to examine the effects of interventions over time. Therefore, we calibrated each of our three candidate models (Fig. 1) to a set of empirical targets using a standard MCMC algorithm [17].

The model targets for calibration included those listed in Table 1 (see complete data in Additional file 1: Tables AF1-AF5). The MCMC algorithm updated vague prior distributions on a set of calibrated parameters to fit these targets. These parameters included the cumulative total fertility rate, median age of fertility, length of the age interval by urban/rural residence, and (when included) by educational attainment level, along with a linear secular trend in fertility (Equation 1 above); the Lee-Carter parameters for mortality rate along with the relative risk of mortality by urban/rural residence and (when included) by educational attainment level; the net rural-to-urban migration rate and linear secular trend in migration rate by (when included) educational attainment level; and (when included) the linear secular trend in educational attainment by urban/rural residence. We repeated the fitting process 10 times from random starting points to ensure convergence to a stable posterior joint distribution of parameter estimates (see Additional file 1: Figure AF1). We then selected among the three candidate models using the Deviance Information Criterion (DIC) to choose the calibrated model that best fit the data relative to its complexity [31] (see Additional file 1: AF1 for details of the MCMC calibration, including convergence statistics).

Two forms of model validation were performed [32, 33]. We evaluated internal validity by ensuring that modeled outcomes fit all input data shown in Table 1. We then evaluated external validity by ensuring that modeled estimates of life expectancy among simulated individuals matched independent estimates [19].

### Assessing the importance of capturing secular trends

To assess the degree to which these efforts to model trends in demographic and socioeconomic conditions could alter model projections, we compared the chosen model to a static model equivalent that included all model components but did not include changes over time in any of the demographic or socioeconomic inputs; that is, fertility, mortality, migration or educational attainment parameters were held fixed at their starting-year values, as is the current standard approach [12–15]. We first compared the two models over the period 1992 to 2010, contrasting their urban- and rural-specific population size estimates with observed data [6]. We next compared the two models starting from the year 2010 and projecting a further 15 years into the future, in order to characterize the degree of divergence between the two sets of model estimates (fixed and with trends) and independent United Nations population projections [6]. We finally compared life expectancy estimates from the two models in terms of both historical (1992–2010) and future projections (2010–2025), and contrasted predictions from the models for the impact of a simulated intervention: efforts to increase the level of educational attainment achieved by rural women, which would have provided universal primary education to rural females in the year 2000 [34]. Prior intervention studies (i.e., cluster randomized trials and natural experiments) have established that increasing primary education availability to women lowers mortality through a number of complex mechanisms such as reducing early marriage and associated premature fertility that increases the risk of maternal mortality [35, 36]. We increased the educational attainment rates among rural females to simulate universal primary education in 2000, comparing the resultant estimated life expectancy differences between the two models over subsequent years.

### Technical details

Model code is provided in Additional file 1: AF1 in accordance with IPSOR-SMDM Modeling Good Research Practices guidelines [32]. All model calculations and simulations were performed in MATLAB version R2013b (The Mathworks, Cambridge, MA, USA). All analyses of survey data used to construct model inputs and targets were performed in Stata 13.1 (StataCorp LP, College Station, TX, USA).

## Results

### Model selection, calibration, and validation

The SPOKE-I model – the model including fertility, mortality, educational attainment, migration, and trends in each of these variables – fit the data listed in Table 1 better than the two simpler models, even after being penalized for increased complexity (Table 2). Specifically, the SPOKE-I model’s *Δ*DIC was >10 points lower than either of the other two models (R^2^ > 86 % for the preferred model fit relative to all data in Table 1).

**Table 3:** Relative risk of death declines significantly with education^a^

Educational level	RR of death – urban	RR of death – rural
0 years	0.92 (0.87–0.97)	1.00 (referent)
>0–6 years	0.76 (0.62–0.90)	0.89 (0.85–0.95)
>6–12 years	0.55 (0.48–0.63)	0.72 (0.68–0.76)
>12 years	0.38 (0.19–0.56)	0.56 (0.41–0.71)

^a^ Estimated mean relative risk of death by educational attainment category is listed with 95 % confidence intervals in parentheses

**Fig. 2:**
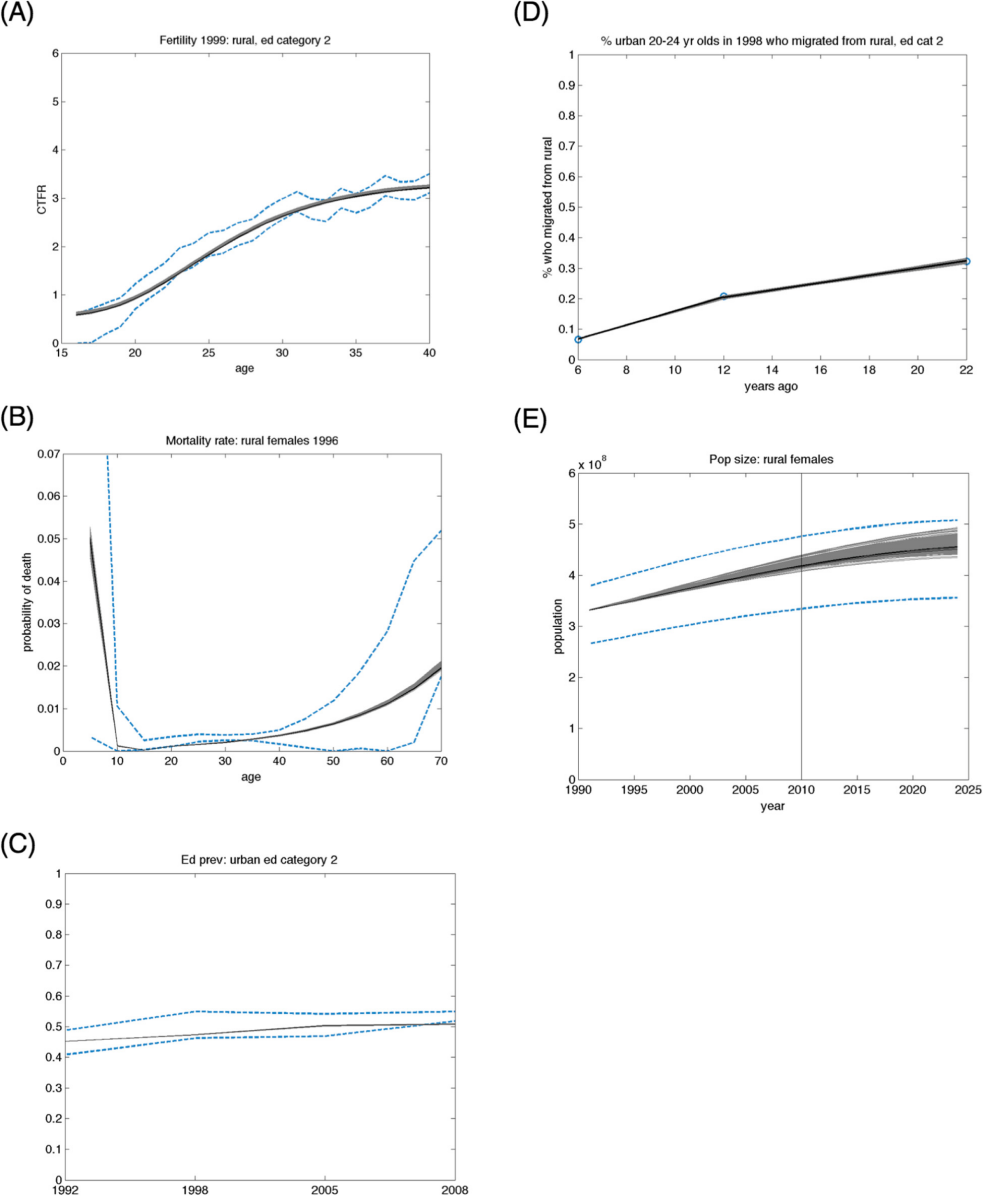
Correspondence between empirical data and the output of the calibrated models over multiple calendar years. The fitted model’s estimates of population dynamics correspond closely to multiple alternative datasets from India. A representative sample of model fits to Indian datasets (listed in Table 1) are illustrated here, including (**a**) fertility, (**b**) mortality, (**c**) educational attainment, (**d**) migration, and (**e**) population size fits. Model fits to the complete datasets for all population cohorts are provided in Additional file 1: Figures AF1–S4. Here, a random subsample of fits are provided as illustrations of how the model was fitted to disaggregated data for various calendar years and birth cohorts, where education category (“ed category”) for each cohort was categorized as 0: no education, 1: >0–6 years education, 2: >6–12 years, and 3: >12 years. Gray shaded areas are results of 10,000 repeated samples from the posterior joint distribution of the fitted model (Fig. 1c), with samples from the interquartile range as black lines and data displayed as dashed blue lines or circles reflecting the 95 % confidence intervals of the input datasets. The solid vertical bar in the population size figure (**e**) reflects the point at which UN data transition from recorded values to model-based estimates

**Fig. 3:**
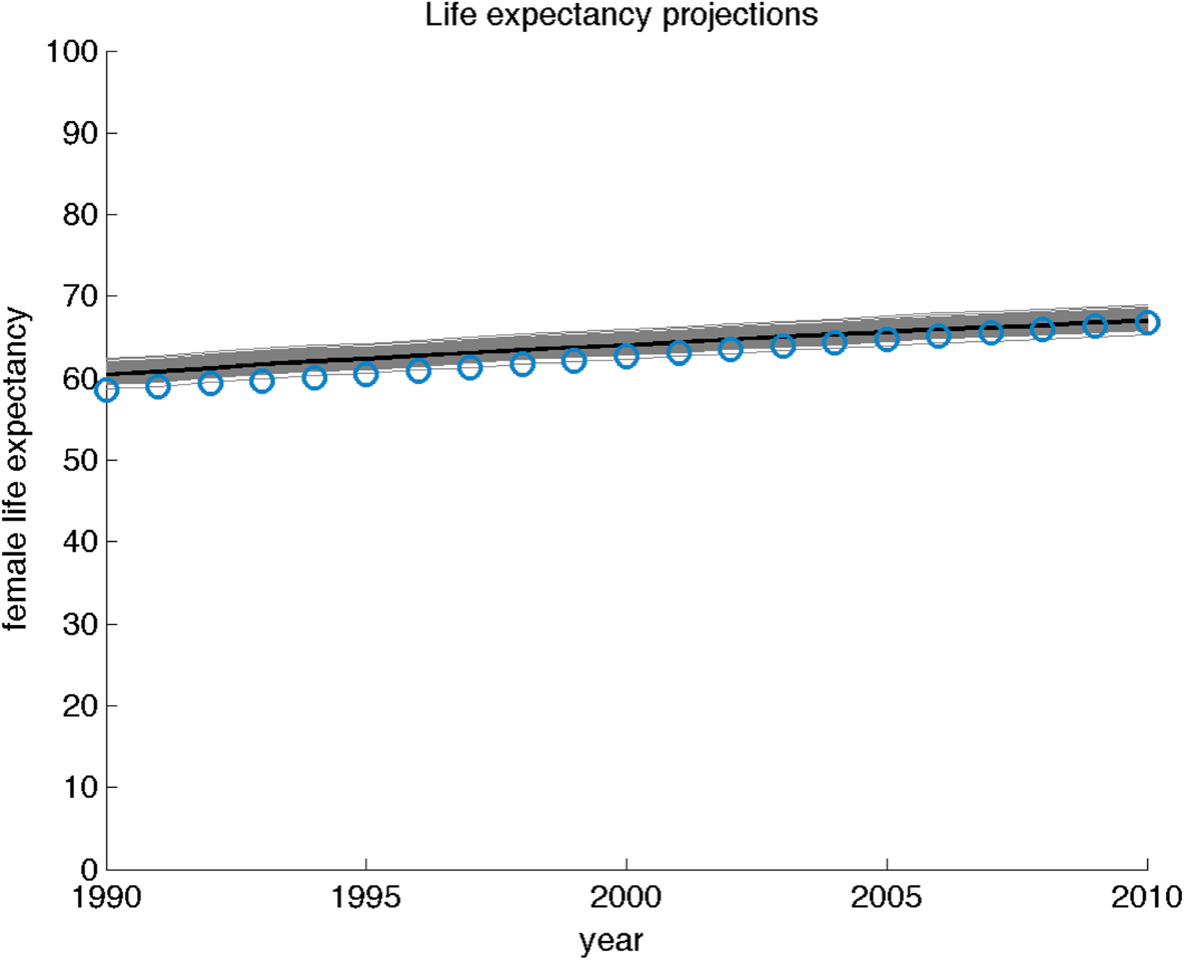
Model-predicted life expectancy validated against independent estimates. Model-predicted life expectancy has face validity when compared against independent World Bank estimates. Gray lines reflect results of 10,000 repeated samples from the posterior joint distribution of the fitted model (Fig. 1c), black lines refer to the samples from the interquartile range of the probability distributions, and blue circles reflect data and its 95 % confidence intervals (diameter of circles)

Modeled outcomes from the calibrated model not only were highly consistent with the calibration data (internal validity) but also achieved consistency with data that were not used for calibration (external validity). Figure. 2 illustrates a sample of typical model fits to the calibration targets derived from these datasets (internal validation), which are further detailed in Additional file 1: Figures AF2-AF6. Fig. 3 shows the comparison of model-predicted life expectancy to independent estimates for life expectancy by year (external validation).

Calibration resulted in a posterior joint distribution of model parameters that revealed the magnitude of important relationships between key demographic, socioeconomic, and population health variables, such as the relationship between higher educational attainment and the higher probability of rural-to-urban migration. Additional file 1: Figure AF1 displays the posterior probability distributions of the fitted parameters, which are further detailed in Additional file 1: Table AF6. Implications of these relationships are described below.

### Modeled fertility trends

**Fig. 4:**
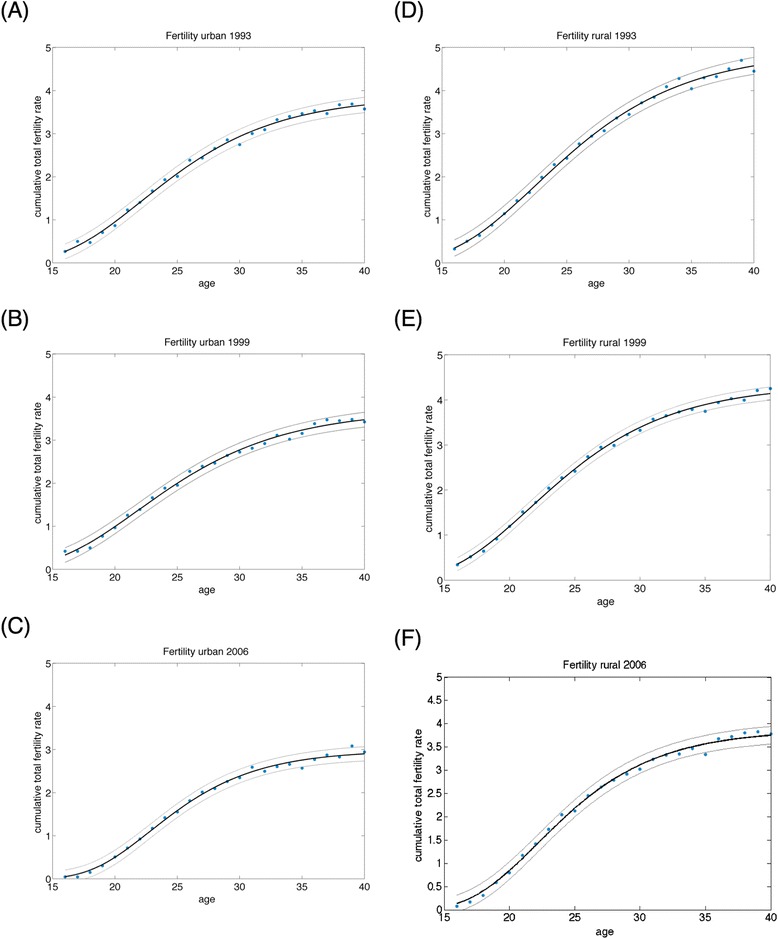
Trends in fertility rates from the calibrated fitted model correspond closely to those estimated from India’s National Family Health Survey. Cumulative fertility rates in India among urban women in (**a**) 1993, (**b**) 1999, and (**c**) 2006, and for rural women in (**d**) 1993, (**e**) 1999, and (**f**) 2006 showing model fits red with 95 % confidence intervals, and data as blue dots. Disaggregated data and model fits by age, birth cohort, urban/rural residence, and educational attainment are provided in Additional file 1: SI Figure AF2. Fertility data were fitted using the Gompertz-Pasupuleti model (Equation 1 in the main text)

Calibrated fertility patterns exhibited a declining time trend and were differential between urban and rural areas and across educational attainment levels. Fig. 4 provides cumulative fertility rates in India among urban and rural women over time along with model fits, and Additional file 1: SI Figure AF2 provides the disaggregated fits by age, birth cohort, urban/rural residence, and educational attainment (R^2^ > 83 % for the model fit to the fertility data). Between 1992 and 2010, model outcomes were that women had 3.4 births on average in their lifetime (95 % CI: 2.3–4.5), with a median age of fertility of 22.4 years (95 % CI: 15.3–29.6). The number of lifetime births declined from an estimated 3.7 per woman (95 % CI: 2.4–5.0) in 1992 to 3.0 (95 % CI: 2.2–3.9) in 2006. The most educated women (those with >12 years of education) experienced 42 % fewer births in their lifetimes (95 % CI: 41–43 %) than women having the lowest educational attainment (those without any schooling). The largest relative difference in fertility between educational categories was between women having 6–12 years of schooling and those having >12 years of schooling (25 % fewer births among the more highly-educated group, 95 % CI: 24–26 %). The relationship between fertility and education was larger than that between fertility and urban/rural residence; urban women were estimated to experience 20 % fewer births than rural women (95 % CI: 6–34 %). The differential impact of attaining >12 years of schooling versus 6–12 years of schooling was greater among urban than rural women (22 % fewer births among urban women with >12 years of schooling than among those with 6–12 years, 95 % CI: 21–23 %, as compared to 29 % fewer births among rural women with >12 years versus 6–12 years, 95 % CI: 28–29 %).

### Modeled mortality trends

**Fig. 5:**
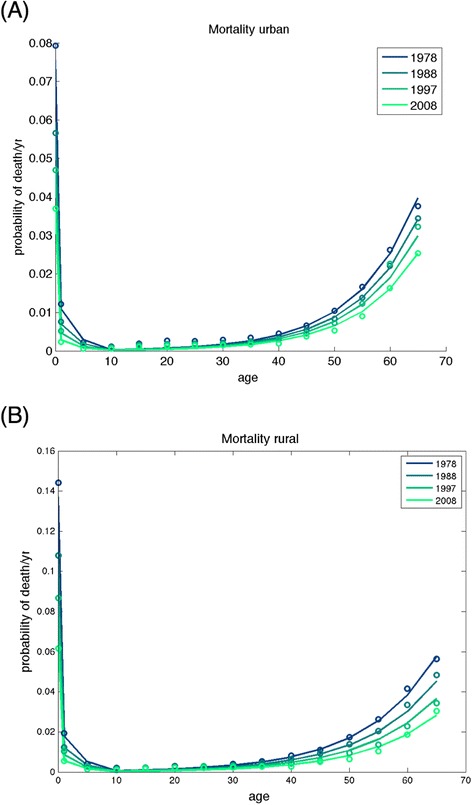
Historical trends in mortality rates produced by the calibrated model correspond closely to rates to those reported by India’s Sample Registration System. Models are displayed as lines against data as circles. Disaggregated mortality data by age, birth cohort, urban/rural residence, educational attainment category, and calendar year, with 95 % confidence intervals, are provided in Additional file 1: SI Figure AF3

Calibrated mortality patterns exhibited strong secular trends and were differential between urban and rural areas and across educational attainment levels. Fig. 5 provides mortality trends in India among urban and rural women over time along with model fits, and Additional file 1: SI Figure AF3 provides the disaggregated model fits by age, birth cohort, urban/rural residence, and educational attainment (R^2^ > 92 % for the model fit to the mortality data). Overall modeled life expectancy improved from 61.3 years for women in 1992 (95 % CI: 59.4–63.2) to 67.0 years in 2010 (95 % CI: 65.0–69.0). As compared to rural women with no educational attainment, the relative risk of death was 0.56 (95 % CI: 0.41–0.71) for rural women with >12 years education and 0.38 (95 % CI: 0.19–0.56) for urban women with >12 years education (Table 3). Mortality rates were approximately 34 % higher among rural than urban women (95 % CI: 30–38 %). There was an estimated decline in the annual probability of death of 0.014 (95 % CI: 0.009 to 0.020) over the period 1992 to 2010.

### Modeled migration trends

We estimated a significant transition towards increasing exposure to urban environments through migration and urbanization. The overall trend suggested that 8 % of rural women would transition to urban areas between 1992 and 2010 (95 % CI: 6–10 %). Additional file 1: Figure AF4 illustrates the detailed model fits to migration rates across all available years of data (R^2^ > 81 % for the model fit to the migration data). Rural-to-urban transitions were particularly common for women in their early 20s (20–24 year-olds had a 16 % chance of transitioning to urban areas between 1992 and 2010, 95 % CI: 14–17 %). The probability of such a rural-to-urban transition was significantly higher for women with greater educational attainment; the probability was only 5 % for 20–24-year-old rural women in the lowest educational attainment category (95 % CI: 4–6 %), but was 33 % for 20–24 year-old rural women in the highest educational attainment category (95 % CI: 32–35 %).

### Modeled educational attainment trends

Calibrated educational attainment exhibited an increasing secular trend and strong urban/rural differences. The proportion of the population in the lowest educational category decreased by 1.4 % (95 % CI: 1.4–1.5 %) among urban and 2.0 % (95 % CI: 1.5–2.6 %) among rural populations over the simulation period, while the proportion of the population in the highest educational category increased non-significantly by 1.3 % among urban (95 % CI: −1.4-3.9 %) and by 0.3 % among rural populations (95 % CI: −0.3-1.0 %). The largest category among urban dwelling women by 2008 was women with 6 to 12 years of schooling, making up 54 % of the urban population but only 39 % of the rural women, among whom the largest group was those with no education (41 %). Additional file 1: SI Figure AF5 illustrates educational attainment trends in India over time along with the model fits disaggregated by age, birth cohort, and urban/rural residence (R^2^ > 97 % for model fits to the educational data).

### Model comparison: Are demographic and socioeconomic time trends necessary for modeling important population health risk factors?

Since many current population health models assume that demographic and socioeconomic exposures are fixed in time, we examined whether our modeling approach that included time trends in these exposures might influence modeled outcomes in important ways. We found that including trends is important for predicting population sizes that remain consistent with both past observed data and future demographic projections made from more complex demography models that are not typically possible to incorporate into health policy simulations. Specifically, we compared our model to an equivalent static model that did not include changes in educational attainment nor in risks of fertility, mortality, or migration over time—a proxy for many models in the current literature.

**Fig. 6:**
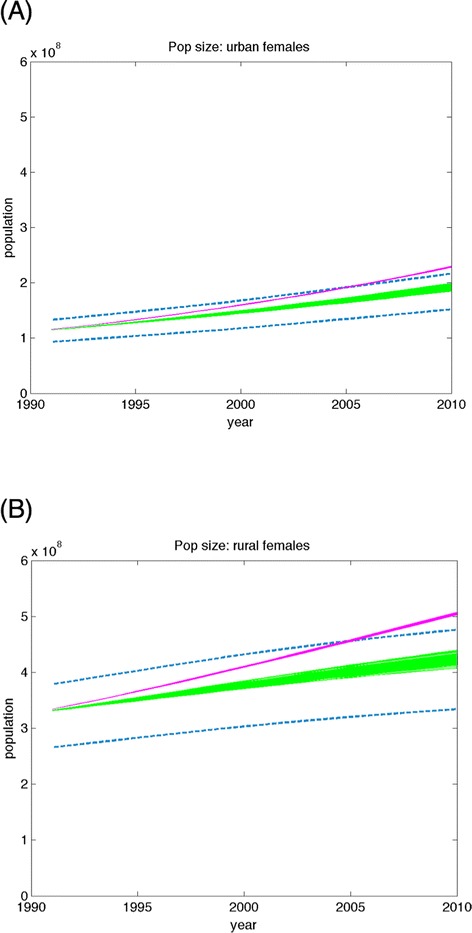
Population models with secular trends in their risk exposure match United Nations historical data on population size more closely than static models that do not include these trends. Historical population size estimates deviate significantly from United Nations projections (dashed blue lines) when using a standard model (magenta) that assumes no change in demographic or socioeconomic variables over time, as compared to our fitted model (green) that includes time trends in demographic and socioeconomic variables. Results are displayed for both (**a**) urban and (**b**) rural females from 1990 to 2010

Figure 6 contrasts the population size predictions from the two models, starting both in 1990 and observing their predictions through 2010. The simulations were carried out 10,000 times through repeated sampling from the parameter uncertainty distributions, and show that the model incorporating demographic and socioeconomic trends is much more likely to make predictions falling within the 95 % confidence intervals of the data, while the current standard model was very likely to deviate significantly from the data within the first few years of the simulation.

**Fig. 7:**
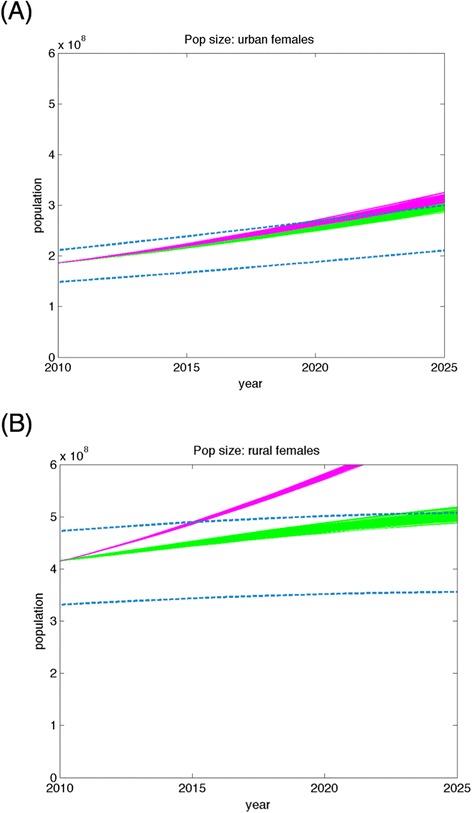
Population models with secular trends in their risk exposure match United Nations future projections of population size more closely than static models that do not include these trends. Future population size estimates deviate significantly from United Nations projections (dashed blue lines) when using a standard model (magenta) that assumes no change in demographic or socioeconomic variables over time, as compared to our fitted model (green) that includes time trends in demographic and socioeconomic variables. Results are displayed for both (**a**) urban and (**b**) rural females from 2010 to 2025

The importance of this deviation is illustrated by the future predictions made by both models starting from the actual population in 2010. As shown in Fig. 7, the model without future trends is likely to overestimate future population size, particularly for rural populations, by failing to account for educational attainment improvements and migration trends that are associated with lower fertility and mortality rates. The overestimation is so severe that the rural female population size estimate for the static model moves outside the 95 % confidence intervals for standard United Nations population estimates by the year 2015, as illustrated in Fig. 7.

**Fig. 8:**
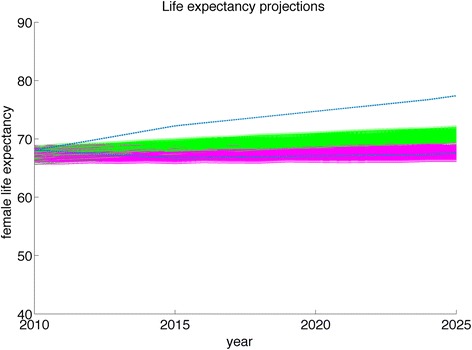
Population models with secular trends in their risk exposure match United Nations future projections of life expectancy more closely than static models that do not include these trends. Life expectancy projections for Indian females from 2010 to 2025 deviate significantly from United Nations projections (dashed blue lines) when using a standard model (magenta) that assumes no change in demographic or socioeconomic variables over time, as compared to our fitted model (green) that includes time trends in demographic and socioeconomic variables

Similarly, as illustrated in Fig. 8, the model without future trends is likely to underestimate life expectancy substantially within a few years of simulation, particularly for rural women. This is due to the large overestimation of mortality rates among this group in a model without trends, as mortality has been falling over time, which could cause a population health model to underestimate years of life lived with disability or to underestimate the years of life saved from public health interventions.

**Fig. 9:**
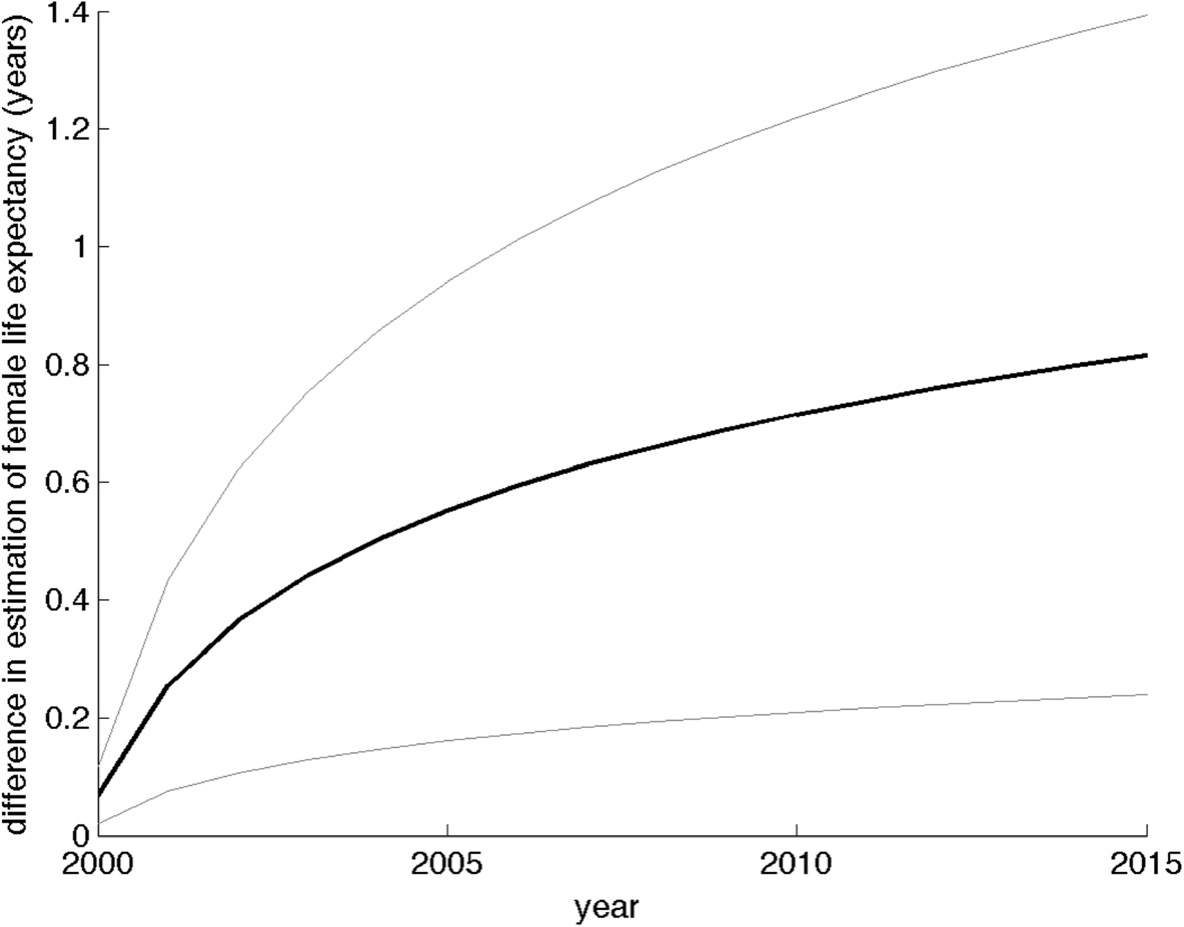
Estimates of life expectancy gains from universal primary education interventions using population models with secular trends are systematically and increasingly higher than from those without secular trends. Life expectancy projections for a simulated population health intervention: the expansion of universal primary education for rural women in the year 2000. The graphs shows a “difference in differences” projection: the incremental benefit of the education intervention according to the SPOKE-I model with demographic and socioeconomic time trends, minus the incremental benefit of the education intervention according to the static model (which assumes no change in demographic or socioeconomic variables over time). The y-axis reflects the difference in incremental life expectancy benefits between the two models. The dark line reflects the median difference, and light gray lines are the 95 % confidence intervals around the result from 10,000 repeated simulations

Explicitly considering a hypothetical intervention to improve rural female educational attainment levels, we compared the model without future trends to the SPOKE-I model in terms of their predictions of incremental life expectancy gains from such an intervention (Fig. 9). When simulating the impact of a proposal to expand primary education to all rural women in the year 2000, the model without future trends estimated a life expectancy benefit that was 0.8 years lower than the dynamic model’s estimate (95 % CI: 0.2–1.4 years). The SPOKE-I model’s estimate incorporated complex effects: the lower mortality risks associated with higher educational attainment, and the simultaneously higher rate of migration to urban areas over time among rural women with higher levels of educational attainment, which carries associated time-varying changes in mortality risk (Table 3).

## Discussion

There is increasing recognition that demographic and socioeconomic conditions can profoundly affect population health and that in many countries these conditions are changing. Yet changes in such conditions are rarely included in mathematical simulation models of population health. Our goal in this paper was to create, validate, and “open source” a simulation modeling approach to allow population health modelers to explicitly reflect changing demographic and socioeconomic conditions.

Our approach allowed us to account for simultaneous, interacting exposures experienced by individuals over their lifetimes – in this case, exposures related to urban/rural residence, educational attainment, and migration. The approach also facilitated quantification of complex correlations among demographic and socioeconomic factors important to health, such as the relations between educational attainment, fertility, migration, and mortality risk.

The experiments conducted in this study add important knowledge to the existing literature on population health modeling. Numerous methods have been proposed to fit increasingly complex models to empirical data [11, 18, 37–41]; our approach focuses specifically on estimation of exposures and trends in exposure in a manner that is both accurate but also simple [18]. The data fitting and calibration approach itself is not new, relying on Markov Chain Monte Carlo methods that are freely-available [42]. The key challenge we addressed here is how to incorporate common demographic and socioeconomic data into a framework that will allow their rapid integration into population health and health policy models. We focused on fertility, mortality, education, and migration data, but our approach can be expanded to other exposures like those repeatedly documented in demographic and health surveys [16]. Incorporating time trends in such data allowed us to generate more accurate population size and life expectancy projections than would have been the case if we had relied on a classical model assuming no change in demographic or socioeconomic factors. Our approach was found to avert potentially serious errors in projections of population health trends. Of note, several HIV-specific models have incorporated some demographic parameters in the past (i.e., migration rates) [43]. Our approach here adds to that literature by permitting systematic evaluation of the importance of each of the standard types of demographic parameters (birth rates, death rates, education rates, migration rates), to develop a routine approach to determining whether or not a parameter would add value or unnecessary complexity to a given model.

As with all mathematical models, our model requires assumptions and associated caveats. Our goal was not to capture all aspects of complex demographic and socioeconomic factors that could influence population size, fertility, or mortality. Rather, we purposefully focused on a model that incorporated several important factors while remaining reasonably parsimonious. The approach is readily adaptable and expandable to other modeling situations, and we have aimed to support this by “open-sourcing” the model code. Nevertheless, the model employed household survey-based data. Such data can suffer from various biases like those related to recall and self-report, which may, for example, lead to some misclassification of educational attainment, particularly as we had to impute mortality rates by educational attainment category given the absence of detailed mortality rates by education class in India’s vital registration database. Nevertheless, our selected model simultaneously fit multiple independent data sources providing information on multiple population health metrics.

Our model is less detailed than many formal models in demography and is intended to be used over short decadal time scales. Our goal was to bridge the gap between health policy models focused on detailed disease natural histories and intervention delivery, and formal demographic models that seek to provide general, very long-range population size projections [7]. Rather than using classical demographic modeling techniques for long-term population size projections, our objective was to find key parameters that could be manipulated to allow public health intervention simulations delivered over 10 to 20 years, such as interventions targeting rural-to-urban migrants, or interventions addressing low educational attainment among rural women [21, 44]. This required modeling both population size itself (as in the demography models) and also generating parameters that characterize underlying factors and relationships linked to changes in population size and mortality (which must be manipulated to simulate interventions). Most formal demographic models capture long-term population trends, but do not include the underlying factors that generate such trends.

Future research should address the question of how data on demographic and socioeconomic conditions might be better standardized across countries. Much of our effort involved data gathering, cleaning, and organization as shown in the Additional file 1: SI Tables to enable easier entry into the model generation and comparison process. Providing organized data in formats that allow similar model comparisons across countries could greatly assist in comparing interventions across low- and middle-income countries [45]. Open databases for commonly-used data collected at multiple time points would provide opportunities to understand how social dynamics affect the vulnerability or resilience of different populations, particularly as population processes such as urbanization offer highly complex outcomes that are not intuitive to anticipate.

## Conclusions

We find that incorporating demographic and socioeconomic trends into mathematical models of population health and health policy is important, as the omission of such trends influences model-projected outcomes in complex ways. Incorporating demographic and socioeconomic trends is currently highly feasible through an “open source” approach developed in this study, and is becoming even more feasible as data from low- and middle-income countries continue to become widely available.

## Additional file

Additional file 1:
**This Additional File text provides further details on the mathematical modeling approach described in the main text, including model code and relevant methodological details.** (DOCX 2291 kb)

## Abbreviations

DIC: Deviance Information Criterion
DLHS: District Level Household and Facility Survey
NFHS: National Family and Health Survey
RR: Relative risk
SPOKE: Stanford Project for Open Knowledge in Epidemiology
SRS: Sample Registration System of India
UNPD: United Nations Population Division
