# Supplementary material for: Quantifying demographic and socioeconomic transitions for computational epidemiology: an open-source modeling approach applied to India
Source: Popul Health Metr. 2015 Aug 1;13:19. doi: 10.1186/s12963-015-0053-1 (PMC4521358; doi:10.1186/s12963-015-0053-1)
Supplement: Additional file 1: — This Additional File text provides further details on the mathematical modeling approach described in the main text, including model code and relevant methodological details. (DOCX 2291 kb) [file 12963_2015_53_MOESM1_ESM.docx]

Additional Files

Table of Contents

1 Fertility model 1

2 Mortality model 2

3 MCMC procedure 3

4 Model code 3

5 References 9

6 Table AF1. Fertility data. 10

7 Table AF2. Mortality data. 13

8 Table AF3. Educational attainment data. 17

9 Table AF4. Migration data. 18

10 Table AF5. Population size data. 22

11 Table AF6. Fitted model parameters. 23

12 Table AF7. Comparison of models. 24

13 Table AF8. Relative risk of death, by education level 25

14 Figure AF1: Probability distributions of fitted parameters. 26

15 Figure AF2: Model fit to fertility data 27

16 Figure AF3: Model fits to mortality data 51

17 Figure AF4: Model fits to migration data 99

18 Figure AF5: Model fits to education data 132

19 Figure AF6: Model fits to life expectancy data 140

Text AF1

This Additional File text provides further details on the mathematical modeling approach described in the main text, including model code and relevant methodological details.

## 1 Fertility model

As noted in the main text, a Gompertz-Pasupuleti model (G-P) was used to model age-specific cumulative fertility (mean number of births experienced by a mother by age of the mother, over the age range 16-49 years) across three waves of NFHS data for which such fertility information was available (wave 1: 1992-3, wave 2: 1998-9, and wave 3: 2005-6). As part of the process of model selection for the fertility model, we fit and compared three alternative statistical models (a standard gamma distribution model, a standard negative binomial distribution model, and the G-P model) to describe the cumulative age-specific fertility rate in each survey wave for groups disaggregated by birth cohort, urban/rural residence and educational attainment level (data shown in Table AF1).

We selected the G-P model as it minimized Akaike’s Information Criterion (AIC) [1], a model selection criterion that selects a model with best fit using the least number of parameters (as an equivalent to DIC for use in ordinary least squares as opposed to MCMC fitting). The model fits to all available, fully disaggregated fertility data across all available years are provided in Figure AF2.

## 2 Mortality model

We estimated mortality rates stratified by age, urban/rural status, calendar year, and educational attainment. In estimating the relative risk of death by educational attainment, a challenge we faced was that in extant data sets describing deaths, the educational attainment level of the deceased is not reported. To overcome this data limitation and estimate the relationship between female educational attainment and mortality, we designed and tested a multistep process described in the main text. First, we predicted female educational attainment level based on household characteristics, using ordinal logistic regression to estimate the relationship between category of educational attainment of women alive in DLHS households. The regression model included: age of the woman, religion of household head, household wealth index, urban/rural residence, total number of marriages in the household, household size and squared household size, and whether the household belonged to a scheduled tribe or scheduled caste.

Second, using the results of this regression, we predicted the likelihood of being in each educational attainment category for women from all households in DLHS, including those who had died and therefore whose actual educational attainment was unobserved. Since the sample was large, we drew a random number to singly impute the educational attainment category for each woman.

Third, we estimated a logistic regression on the likelihood that a given woman had died based on the fully interacted model of predicted educational attainment category, woman’s age, and her urban/rural residential status. For each age and urban/rural birth cohort, the educational attainment-specific relative risk of death was computed as the predicted marginal probability of death for a given group compared to the category of women with the lowest level of educational attainment (equation 3 in the main text).

The fully disaggregated mortality rate estimates are provided in Table AF2. We fitted a Lee-Carter-type model to the log mortality rate [2], by fitting a constant, a parameter multiplied by calendar year, and a parameter multiplied by age to log-mortality rates. We fit the log mortality rates in three age clusters, <1 year olds, 1-10 year olds, >10 year olds, because the log mortality rates have clear breakpoints at these age divisions, allowing for simple decomposition of the rates into three linear models. The model fits to the fully disaggregated data are provided in Figure AF3.

## 3 MCMC procedure

To perform Markov Chain Monte Carlo (MCMC) estimation of parameter values fitting the overall model to (simultaneously) all available data (Tables SI1-SI5), we used a standard MCMC approach that involves an efficient implementation of an adaptive Metropolis sampler with delayed rejection [3]. We specifically used the freely-available MCMC package in the program MATLAB (version R2013b, The Mathworks, Cambridge, MA, USA), which samples from multi-dimensional prior distributions for the parameters being fitted, and uses a rejection algorithm to sample increasingly from joint probability regions where the posterior probability is high by determining whether the model output of a new parameter sample is closer or farther away from the 95% confidence interval of the target data. See details in reference [3]. Installation instructions are provided with the model code below. We started with flat prior distributions defined as inverse chi-squared distributions with mean zero and infinite standard deviation. The algorithm was run over 100,000 iterations after a 10,000 iteration burn-in period and 1:10 thinning to generate a joint posterior distribution for the parameters, illustrated in Figure AF1 and detailed further in Table AF6. We used standard Geweke criteria to test for convergence [4], the results of which are also provided in Table AF6.

To ensure stability of our estimates, we repeated the process from ten randomly-initiated start points for all parameters, sampling randomly from the flat prior distributions and ensuring stable convergence to the same joint posterior distribution to three decimal place values for all parameters.

In order to produce population demographic and life expectancy estimates from the model, we sampled 10,000 times from the joint posterior probability distribution of the parameters (Figure AF1 and Table AF6) and re-ran the model using each of these 10,000 parameter sets.

## 4 Model code

The model code is organized as a series of MATLAB functions, with instructions and labels provided as comments designated by the percent (%) symbol. Note that the code is designed for readability and easy interpretability by other users, but is not necessarily the most efficient possible code; in areas, labeled below, multiple lines of code or functions can be consolidated as indicated in order to produce more parsimonious and efficient simulations, although we provide the long-hand code here for ease of interpretability. We recommend that the code be run on computers with at least 4GB RAM and 1GB free memory; please note that the MCMC procedure can require several minutes to complete, and we recommend closing other applications during its execution.

The code performs the following functions: (a) the data provided in Tables SI1-SI5 are imported (requires an Internet connection) and the model specifies parameters to be fit; (b) the model calls a function to simulate fertility, mortality, educational attainment and migration over time periods specified by the user, using a stochastic discrete-time microsimulation approach with user-specified time intervals; (c) the model processes population size estimates by cohort (where cohorts are defined by all combinations of current urban/rural residence, educational attainment in the four categories described in the main text, and age in categories of 0-4, 5-9, 10-14, 15-19, 20-24, 25-44, 45-64, 65-79, and 80+ years); (d) the fitted parameters are displayed as joint posterior probability distributions with summary statistics and associated figures; and (e) the distributions are sampled to generate life expectancy estimates for simulated cohorts over user-specified time scales.

A copy of the sourcecode for the anthropometric model, along with a link to example data to which it can be applied is available at: https://github.com/sanjaybasu/SPOKE

% (Licensed under a [Creative Commons Attribution-NonCommercial-ShareAlike 4.0 International License](http://creativecommons.org/licenses/by-nc-sa/4.0/) by Basu & Goldhaber-Fiebert, 2014)

function anthroex

clear; clc;

% load data

data = urlread('http:// www.stanford.edu/~basus/spoke/india-anthropom.txt'); % data available concurrent with publication

model.ssfun = @anthross;

% load MCMC package: http://helios.fmi.fi/~lainema/mcmc/mcmcstat.zip

% Define parameter sampling constraints if any.

% {'name', start, [min,max], N(mu,s^2)}

params = {data.TabS6}; % initial parameters loaded, see Table AF6 for descriptions

% default prior distribution is sigma2 ~ invchisq(S20,N0), the inverse chi

% squared distribution (see for example Gelman et al.). The

% components (female urban, female rural) all have separate variances.

model.S20 = std(data.ydata);

model.N0 = mean(data.ydata);

% First generate an initial chain.

options.nsimu = 1000; % burn-in period

[results, chain, s2chain]= mcmcrun(model,data,params,options);

% Then re-run starting from the results of the previous run;

% this may take several minutes

options.nsimu = 10000;

[results, chain, s2chain] = mcmcrun(model,data,params,options, results);

% Chain plots should reveal that the chain has converged and we can

% use the results for estimation and predictive inference.

figure

mcmcplot(chain,[],results,'pairs');

figure

mcmcplot(chain,[],results,'denspanel',2);

% Function |chainstats| calculates mean and std from the chain and

% estimates the Monte Carlo error of the estimates. Number |tau| is

% the integrated autocorrelation time and |geweke| is a test

% for a null hypothesis that the chain has converged.

chainstats(chain,results)

% In order to use the |mcmcpred| function we need

% function |modelfun| with input arguments given as

% |modelfun(xdata,theta)|. We construct this as an anonymous function.

modelfun = @(d,th) anthrofunpred(data,th);

% We sample parameter realizations from |chain| and |s2chain|

% and calculate the predictive plots.

nsample = 10000;

out = mcmcpred(results,chain,s2chain,data.ydata,modelfun,nsample);

figure

% add the 'y' observations to the plot

hold on

for i=1:55 % distinct data columns being fitted

subplot(56/4,4,i)

hold on

plot(data.ydata(:,i),'o');

hold off

end

mcmcpredplot(out);

function ss = anthross(theta,data)

ydata = data.ydata;

ymodel = anthrofun(theta);

ss = (sum((ymodel - ydata).^2));

% ydata = [{'fertdata92'}, {'fertdata98'}, {'fertdata05'},{'urbandeathdata'},{'ruraldeathdata'},{'unpopproj'},{'edprev'}]

% organization of the data are as follows, by column:

% fertdata92 = NFHS1 rows age 16-49, columns 1-4 urban, 5-8 rural, 1/2/3/4/1/2/3/4 ed levels

% fertdata98 = NFHS2

% fertdata05 = NFHS3

% urbandeathdata = rows = age (0-65 by increments of 5), columns = years

% ruraldeathdata

% unpopproject = 1992 - 2025 un projections female urban, then female rural (2nd col)

% edprevdata = [edprev 1992; 1998; 2005; 2008];

function ydot=anthrofun(theta)

% individuals are defined by age, sex, urban/rural, and education level (none, primary, secondary, more)

% sex = 0 if male, 1 if female

% urban/rural = 1 if urban, 2 if rural

% education = 0, 1, 2, 3 in order of increasing ed (0 years, 1-6 yrs, 6-12 yrs, >12 yrs)

% cohorts = 1-8, first four urban, second four rural; first four education 0/1/2/3, second four education 0/1/2/3

dt=1/365; %time step, default set to 1 day

yrs=[user-defined starting year]:dt:[user defined ending year]; % time range simulated; note, change starting conditions below (labeled) if changing years of simulation period

% fertility rate by age and cohort, GP fertility model of NFHS data

% cumulative fertility rate =f.*0.5.^((log(0.95)/log(0.05)).^((maternal age -a)/b));

% f is the saturation level (cumulative total fertility rate)

% a is median age of fertility (age of giving birth to half of the total number of children)

% b is length of the age interval during which the fertility level rises from 5% to 95% of the saturation level

f=repmat(theta(1),8,length(yrs));

a=repmat(theta(2),8,length(yrs));

b=repmat(theta(3),8,length(yrs));

rrf=[1 theta(17) theta(18) theta(19) theta(20) theta(21) theta(22) theta(23)]; % RR of fertility by cohort

for age=14:50;

ctfr(:,:,age)=(f.*0.5.^((log(0.95)/log(0.05)).^((age-a)./b))+repmat(theta(24)*yrs,8,1)).*repmat(rrf',1,length(yrs)); % cum tot fert rate by year, SG model

end

birthrate(:,:,15:50)=ctfr(:,:,15:50)-ctfr(:,:,14:49); % birth rate by cohort (rows), year (columns), and maternal age (depth)

for age=16:49

fertmodel92(age-15,:)=ctfr(:,1,age)'; % generate output vectors for fitting fertility outcomes to three survey waves

fertmodel98(age-15,:)=ctfr(:,7,age)';

fertmodel05(age-15,:)=ctfr(:,14,age)';

end

rrm=[1 theta(11) theta(12) theta(13) theta(35) theta(11)*theta(35) theta(12)*theta(35) theta(13)*theta(35)]; % RR of death by cohort (urban/rural and ed category)

for age = 1:1 % infant deaths

mdr(:,:,age)=exp(repmat(theta(25),8,length(yrs))+repmat(theta(26)*yrs,8,1)).*repmat(rrm',1,length(yrs));

fdr(:,:,age)=exp(repmat(theta(27),8,length(yrs))+repmat(theta(28)*yrs,8,1)).*repmat(rrm',1,length(yrs));

end

for age = 2:10 % older deaths

mdr(:,:,age)=exp(repmat(theta(29),8,length(yrs))+repmat(theta(30),8,length(yrs))*age+repmat(theta(31)*yrs,8,1)).*repmat(rrm',1,length(yrs));

fdr(:,:,age)=exp(repmat(theta(32),8,length(yrs))+repmat(theta(33),8,length(yrs))*age+repmat(theta(34)*yrs,8,1)).*repmat(rrm',1,length(yrs));

end

for age = 11:100 % older deaths

mdr(:,:,age)=exp(repmat(theta(4),8,length(yrs))+repmat(theta(5),8,length(yrs))*age+repmat(theta(6)*yrs,8,1)).*repmat(rrm',1,length(yrs));

fdr(:,:,age)=exp(repmat(theta(7),8,length(yrs))+repmat(theta(8),8,length(yrs))*age+repmat(theta(9)*yrs,8,1)).*repmat(rrm',1,length(yrs));

end

clear umd rmd ufd rfd

edage=[starting dist of ed prev for starting year];

% sorting to match data matrices for fitting

for age=5:5:65

umd(1+age/5,:)=sum(mdr(1:4,:,age).*repmat(edage(1:4),1,length(yrs)));

rmd(1+age/5,:)=sum(mdr(5:8,:,age).*repmat(edage(1:4),1,length(yrs)));

ufd(1+age/5,:)=sum(fdr(1:4,:,age).*repmat(edage(1:4),1,length(yrs)));

rfd(1+age/5,:)=sum(fdr(5:8,:,age).*repmat(edage(1:4),1,length(yrs)));

end

umd(1,:)=sum(mdr(1:4,:,1).*repmat(edage(1:4),1,length(yrs)));

rmd(1,:)=sum(mdr(5:8,:,1).*repmat(edage(1:4),1,length(yrs)));

ufd(1,:)=sum(fdr(1:4,:,1).*repmat(edage(1:4),1,length(yrs)));

rfd(1,:)=sum(fdr(5:8,:,1).*repmat(edage(1:4),1,length(yrs)));

umd=umd';rmd=rmd';ufd=ufd';rfd=rfd';

umd=[umd(1,:);umd(4,:);umd(5,:);umd(8:16,:)];

rmd=[rmd(1,:);rmd(4,:);rmd(5,:);rmd(8:16,:)];

ufd=[ufd(1,:);ufd(4,:);ufd(5,:);ufd(8:16,:)];

rfd=[rfd(1,:);rfd(4,:);rfd(5,:);rfd(8:16,:)];

udmodel=[umd; ufd];

udmodel(25:34,:)=zeros(10,14);

rdmodel=[rmd; rfd];

rdmodel(25:34,:)=zeros(10,14);

% create initial population in starting year

pop=[user defined initial pop for starting year]; % pop size total

sexratio=[user defined sex ratio for starting year]; % proportion female at birth

urban=[user defined urban ratio for starting year]; % proportion urban in starting year

distage=[user defined age ratio for starting year]; % starting distribution of females into age groups

fempop=repmat([distage.*pop.*sexratio]',8,1); % fem pop size, cohorts in rows, columns are age groups, 0-4, 5-9, 10-14, 15-19, 20-24, 25-44, 45-64, 65-79, 80+

fempop=fempop.*repmat(edage,1,length(fempop));

fempop(1:4,:)=fempop(1:4,:)*urban; % create urban/rural distribution for starting year

fempop(5:8,:)=fempop(5:8,:)*(1-urban);

% pop change in each year of simulation: births, deaths, aging

for time=1:length(yrs) births(:,time)=dt.*(birthrate(:,time,2).*fempop(:,1)+birthrate(:,time,7).*fempop(:,2)+birthrate(:,time,12).*fempop(:,3)+birthrate(:,time,17).*fempop(:,4)+birthrate(:,time,22).*fempop(:,5)+birthrate(:,time,35).*fempop(:,6)+birthrate(:,time,50).*fempop(:,7));

fempop(:,1)=fempop(:,1)+sexratio.*births(:,time);

deathf(:,:,time)=dt.*[fdr(:,time,1).*fempop(:,1) fdr(:,time,7).*fempop(:,2) fdr(:,time,12).*fempop(:,3) fdr(:,time,17).*fempop(:,4) fdr(:,time,22).*fempop(:,5) fdr(:,time,35).*fempop(:,6) fdr(:,time,55).*fempop(:,7) fdr(:,time,72).*fempop(:,8) fdr(:,time,90).*fempop(:,9)];

fempop=fempop-deathf(:,:,time);

% aging across cohorts

fempop(:,1)=fempop(:,1)-dt.*fempop(:,1)/5;

fempop(:,2)=fempop(:,2)+dt.*fempop(:,1)/5-dt.*fempop(:,2)/5;

fempop(:,3)=fempop(:,3)+dt.*fempop(:,2)/5-dt.*fempop(:,3)/5;

fempop(:,4)=fempop(:,4)+dt.*fempop(:,3)/5-dt.*fempop(:,4)/5;

fempop(:,5)=fempop(:,5)+dt.*fempop(:,4)/5-dt.*fempop(:,5)/5;

fempop(:,6)=fempop(:,6)+dt.*fempop(:,5)/5-dt.*fempop(:,6)/20;

fempop(:,7)=fempop(:,7)+dt.*fempop(:,6)/20-dt.*fempop(:,7)/20;

fempop(:,8)=fempop(:,8)+dt.*fempop(:,7)/20-dt.*fempop(:,8)/15;

fempop(:,9)=fempop(:,9)+dt.*fempop(:,8)/15;

% education secular trends across cohorts

fempop(1,:)=fempop(1,:)-dt.*theta(14).*fempop(1,:);

fempop(2,:)=fempop(2,:)+dt.*theta(14).*fempop(1,:)-dt.*theta(15).*fempop(2,:);

fempop(3,:)=fempop(3,:)+dt.*theta(15).*fempop(2,:)+dt.*theta(16).*fempop(3,:);

fempop(4,:)=fempop(4,:)+dt.*theta(16).*fempop(3,:);

fempop(5,:)=fempop(5,:)-dt.*theta(14).*fempop(5,:);

fempop(6,:)=fempop(6,:)+dt.*theta(14).*fempop(5,:)-dt.*theta(15).*fempop(6,:);

fempop(7,:)=fempop(7,:)+dt.*theta(15).*fempop(6,:)+dt.*theta(16).*fempop(7,:);

fempop(8,:)=fempop(8,:)+dt.*theta(16).*fempop(7,:);

% rural-urban migration; can disaggregate by ed category as described in main text

fempop(1:4,:)=fempop(1:4,:)+dt.*fempop(1:4,:)*theta(10);

fempop(5:8,:)=fempop(5:8,:)-dt.*fempop(1:4,:)*theta(10);

deaths(:,time)=sum(deathf(:,:,time)');

finalfempop(:,time)=sum(fempop')';

fitunurban(:,time)=sum(sum(fempop(1:4,:))');

fitunrural(:,time)=sum(sum(fempop(5:8,:))');

edprev(:,time)=[(sum(fempop(1:4,:)')./sum(sum(fempop(1:4,:)')))'; sum(fempop(5:8,:)')./sum(sum(fempop(5:8,:)')))'];

end

edprevmodel=[edprev(:,1);edprev(:,7);edprev(:,19);edprev(:,22)];

edprevmodel(33:34)=[0;0];

unmodel=[fitunurban;fitunrural]'; % sorting data to match UN pop size estimates

% overall data matrix output to fit against data

ydot=[fertmodel92 fertmodel98 fertmodel05 udmodel rdmodel unmodel edprevmodel];

% simulate estimated life expectancy

init=[user defined base year life exp by male/female urban/rural];

pars=[birth/death/ed trend/migration matrix by user for starting year];

popsize=[user defined starting pop size for simulated starting year];

for year=[starting year]:[ending year]

pop=zeros(3,popsize);

% urban

for age=0:65

if age<1 deathrate=exp((pars(1,9)*edprev(1)+pars(2,9)*edprev(2)+pars(3,9)*edprev(3)+pars(4,9)*edprev(4))*year+(pars(1,10)*edprev(1)+pars(2,10)*edprev(2)+pars(3,10)*edprev(3)+pars(4,10)*edprev(4)));

elseif age>=1 && age<10 deathrate=exp((pars(1,11)*edprev(1)+pars(2,11)*edprev(2)+pars(3,11)*edprev(3)+pars(4,11)*edprev(4))+(pars(1,12)*edprev(1)+pars(2,12)*edprev(2)+pars(3,12)*edprev(3)+pars(4,12)*edprev(4))*age+(pars(1,13)*edprev(1)+pars(2,13)*edprev(2)+pars(3,13)*edprev(3)+pars(4,13)*edprev(4))*year);

elseif age>=10

deathrate=exp((pars(1,14)*edprev(1)+pars(2,14)*edprev(2)+pars(3,14)*edprev(3)+pars(4,14)*edprev(4))+(pars(1,15)*edprev(1)+pars(2,15)*edprev(2)+pars(3,15)*edprev(3)+pars(4,15)*edprev(4))*age+(pars(1,16)*edprev(1)+pars(2,16)*edprev(2)+pars(3,16)*edprev(3)+pars(4,16)*edprev(4))*year);

end

prob=rand(1,length(pop));

pop(1,pop(1,:)==0&prob<deathrate)=1; % row 1 = death if =1, alive if =0

pop(2,pop(2,:)==0&prob<deathrate)=year; % row 2 = year of death

pop(3,pop(3,:)==0&prob<deathrate)=age; % row 3 = age of death

end

dle(year,3) = mean(pop(3,pop(3,:)>0));

clear pop;

% rural

pop=zeros(3,popsize);

for age=0:100

if age<1

deathrate=exp((pars(5,9)*edprev(1)+pars(6,9)*edprev(2)+pars(7,9)*edprev(3)+pars(8,9)*edprev(4))*year+(pars(5,10)*edprev(1)+pars(6,10)*edprev(2)+pars(7,10)*edprev(3)+pars(8,10)*edprev(4)));

elseif age>=1 && age<10

deathrate=exp((pars(5,11)*edprev(1)+pars(6,11)*edprev(2)+pars(7,11)*edprev(3)+pars(8,11)*edprev(4))+(pars(5,12)*edprev(1)+pars(6,12)*edprev(2)+pars(7,12)*edprev(3)+pars(8,12)*edprev(4))*age+(pars(5,13)*edprev(1)+pars(6,13)*edprev(2)+pars(7,13)*edprev(3)+pars(8,13)*edprev(4))*year);

elseif age>=10

deathrate=exp((pars(5,14)*edprev(1)+pars(6,14)*edprev(2)+pars(7,14)*edprev(3)+pars(8,14)*edprev(4))+(pars(5,15)*edprev(1)+pars(6,15)*edprev(2)+pars(7,15)*edprev(3)+pars(8,15)*edprev(4))*age+(pars(5,16)*edprev(1)+pars(6,16)*edprev(2)+pars(7,16)*edprev(3)+pars(8,16)*edprev(4))*year);

end

prob=rand(1,length(pop));

pop(1,pop(1,:)==0&prob<deathrate)=1; % row 1 = death if =1, alive if =0

pop(2,pop(2,:)==0&prob<deathrate)=year; % row 2 = year of death

pop(3,pop(3,:)==0&prob<deathrate)=age; % row 3 = age of death

end

dle(year,4) = mean(pop(3,pop(3,:)>0));

end

% final LE matrices

results=dle([starting year]:[ending year],:)-repmat(dle([starting year],:),length(dle([starting year]:[ending year],:)),1)+repmat(init,length(dle([starting year]:[ending year],:)),1);

## 5 References

1. Akaike H (1974) A new look at the statistical model identification. IEEE Trans Autom Control 19: 716–723.

2. Lee R (2000) The Lee-Carter method for forecasting mortality, with various extensions and applications. North Am Actuar J 4: 80–91.

3. Haario H, Laine M, Mira A, Saksman E (2006) DRAM: Efficient adaptive MCMC. Stat Comput 16: 339–354. doi:10.1007/s11222-006-9438-0.

4. Cowles MK, Carlin BP (1996) Markov chain Monte Carlo convergence diagnostics: a comparative review. J Am Stat Assoc 91: 883–904.

5. International Institute for Population Sciences (1995) National Family Health Survey, India 1992-93. Bombay: IIPS.

6. International Institute for Population Sciences (2001) National Family Health Survey, India 1998-99. Bombay: IIPS.

7. International Institute for Population Sciences (2008) National Family Health Survey, India 2005-06. Bombay: IIPS.

8. Ministry of Home Affairs (2011) Sample Registration System. New Delhi: Office of the Registrar General & Census Commissioner, India.

9. International Institute for Population Sciences (2010) District Level Household and Facility Survey 2007-08. Bombay: IIPS.

10. United Nations (2013) World Population Prospects: The 2012 Revision. Geneva: UN.

## 6 Table AF1. Fertility data.

(A) Cumulative total fertility rate (CTFR), recorded in the National Family Health Survey wave 1, 1992-1993 [5]. Educational categories are: 0: none; 1: 1 to 6 years; 2: >6 to 12 years; 3: >12 years.

| Urban/Rural status: | Urban | | | | Rural | | | |
| --- | --- | --- | --- | --- | --- | --- | --- | --- |
| Educational attainment: | 0 | 1 | 2 | 3 | 0 | 1 | 2 | 3 |
| Age of mother | CTFR | | | | | | | |
| 16 | 0.334 | 0.422 | 0.217 | 0.000 | 0.377 | 0.283 | 0.196 | 0.000 |
| 17 | 0.750 | 0.505 | 0.410 | 0.000 | 0.545 | 0.510 | 0.390 | 0.000 |
| 18 | 0.701 | 0.578 | 0.369 | 0.000 | 0.677 | 0.664 | 0.535 | 0.000 |
| 19 | 1.017 | 0.990 | 0.481 | 0.276 | 0.996 | 0.837 | 0.585 | 0.000 |
| 20 | 1.144 | 0.824 | 0.792 | 0.279 | 1.258 | 1.133 | 0.836 | 0.532 |
| 21 | 1.625 | 1.364 | 1.044 | 0.510 | 1.568 | 1.462 | 1.131 | 0.270 |
| 22 | 1.705 | 1.738 | 1.237 | 0.662 | 1.802 | 1.536 | 1.257 | 0.456 |
| 23 | 2.192 | 1.813 | 1.451 | 0.689 | 2.172 | 1.995 | 1.517 | 0.362 |
| 24 | 2.567 | 2.079 | 1.681 | 0.647 | 2.486 | 2.202 | 1.790 | 1.006 |
| 25 | 2.672 | 2.275 | 1.671 | 0.903 | 2.657 | 2.415 | 1.841 | 0.834 |
| 26 | 2.995 | 2.830 | 2.053 | 1.181 | 3.059 | 2.437 | 2.161 | 1.058 |
| 27 | 3.030 | 2.659 | 2.179 | 1.247 | 3.244 | 2.768 | 2.217 | 1.335 |
| 28 | 3.509 | 2.879 | 2.229 | 1.378 | 3.326 | 2.982 | 2.449 | 1.112 |
| 29 | 3.673 | 3.074 | 2.442 | 1.628 | 3.698 | 3.131 | 2.598 | 1.405 |
| 30 | 3.491 | 3.092 | 2.286 | 1.841 | 3.794 | 3.260 | 2.578 | 2.106 |
| 31 | 3.917 | 3.147 | 2.550 | 1.767 | 4.094 | 3.480 | 2.820 | 1.611 |
| 32 | 3.885 | 3.302 | 2.689 | 1.944 | 4.177 | 3.632 | 3.092 | 1.740 |
| 33 | 4.228 | 3.426 | 2.918 | 1.944 | 4.499 | 3.736 | 3.177 | 1.891 |
| 34 | 4.353 | 3.692 | 2.908 | 1.936 | 4.685 | 4.087 | 3.243 | 2.619 |
| 35 | 4.328 | 3.811 | 3.004 | 2.123 | 4.423 | 3.762 | 3.129 | 2.461 |
| 36 | 4.604 | 3.577 | 3.020 | 2.128 | 4.724 | 3.886 | 3.375 | 1.858 |
| 37 | 4.402 | 3.804 | 2.944 | 2.186 | 4.799 | 3.916 | 3.204 | 2.684 |
| 38 | 5.051 | 3.742 | 2.944 | 2.221 | 4.923 | 4.180 | 3.545 | 2.175 |
| 39 | 4.784 | 4.204 | 3.004 | 2.360 | 5.126 | 4.252 | 3.786 | 2.757 |
| 40 | 4.360 | 3.817 | 3.222 | 2.113 | 4.825 | 4.385 | 3.425 | 2.774 |
| 41 | 5.441 | 4.347 | 3.327 | 2.324 | 5.134 | 4.617 | 3.624 | 2.355 |
| 42 | 4.721 | 4.706 | 3.514 | 2.268 | 5.340 | 4.525 | 3.954 | 2.246 |
| 43 | 4.859 | 4.334 | 3.488 | 2.416 | 5.303 | 4.841 | 3.572 | 2.346 |
| 44 | 5.234 | 4.487 | 3.113 | 2.868 | 5.524 | 4.661 | 4.351 | 2.441 |
| 45 | 4.948 | 4.258 | 3.317 | 2.293 | 5.242 | 4.644 | 4.105 | 2.781 |
| 46 | 5.511 | 4.256 | 3.398 | 2.278 | 5.519 | 4.992 | 4.121 | 1.520 |
| 47 | 5.447 | 4.345 | 3.602 | 2.362 | 5.666 | 5.009 | 3.847 | 2.483 |
| 48 | 5.456 | 4.941 | 3.595 | 2.966 | 5.518 | 4.976 | 4.078 | 3.256 |
| 49 | 5.567 | 4.846 | 3.299 | 2.677 | 5.790 | 4.874 | 4.059 | 1.755 |

(B) Cumulative total fertility rate (CTFR), recorded in the National Family Health Survey wave 2, 1998-1999 [6].

| Urban/Rural status: | Urban | | | | Rural | | | |
| --- | --- | --- | --- | --- | --- | --- | --- | --- |
| Educational attainment: | 0 | 1 | 2 | 3 | 0 | 1 | 2 | 3 |
| Age of mother | CTFR | | | | | | | |
| 16 | 0.482 | 0.538 | 0.462 | 0.000 | 0.407 | 0.413 | 0.202 | 0.000 |
| 17 | 0.619 | 0.620 | 0.313 | 0.193 | 0.622 | 0.479 | 0.356 | 0.068 |
| 18 | 0.862 | 0.548 | 0.395 | 0.034 | 0.715 | 0.710 | 0.509 | 0.192 |
| 19 | 1.223 | 0.914 | 0.575 | 0.371 | 1.056 | 0.937 | 0.638 | 0.400 |
| 20 | 1.272 | 1.197 | 0.865 | 0.477 | 1.314 | 1.243 | 0.962 | 0.643 |
| 21 | 1.557 | 1.443 | 1.203 | 0.621 | 1.703 | 1.468 | 1.195 | 0.825 |
| 22 | 1.986 | 1.727 | 1.145 | 0.666 | 1.902 | 1.754 | 1.403 | 0.965 |
| 23 | 2.129 | 1.978 | 1.496 | 0.954 | 2.238 | 2.056 | 1.713 | 1.083 |
| 24 | 2.628 | 2.126 | 1.633 | 0.966 | 2.537 | 2.243 | 1.828 | 1.104 |
| 25 | 2.381 | 2.154 | 1.874 | 1.115 | 2.633 | 2.424 | 2.046 | 1.444 |
| 26 | 2.999 | 2.491 | 2.039 | 1.365 | 3.101 | 2.625 | 2.103 | 1.638 |
| 27 | 3.047 | 2.668 | 2.231 | 1.312 | 3.371 | 2.730 | 2.257 | 1.703 |
| 28 | 3.319 | 2.835 | 2.122 | 1.524 | 3.374 | 2.873 | 2.342 | 1.690 |
| 29 | 3.422 | 2.858 | 2.383 | 1.713 | 3.618 | 3.092 | 2.587 | 1.871 |
| 30 | 3.577 | 3.031 | 2.411 | 1.715 | 3.652 | 3.350 | 2.769 | 1.793 |
| 31 | 3.752 | 3.151 | 2.441 | 1.813 | 3.980 | 3.367 | 2.925 | 2.176 |
| 32 | 3.946 | 3.470 | 2.469 | 1.839 | 4.146 | 3.483 | 2.777 | 2.195 |
| 33 | 4.145 | 3.348 | 2.727 | 2.035 | 4.313 | 3.440 | 2.734 | 2.233 |
| 34 | 3.938 | 3.284 | 2.667 | 2.067 | 4.281 | 3.541 | 2.992 | 2.208 |
| 35 | 4.117 | 3.567 | 2.765 | 2.106 | 4.267 | 3.441 | 2.889 | 2.376 |
| 36 | 4.365 | 3.857 | 2.993 | 2.226 | 4.497 | 3.676 | 3.030 | 2.233 |
| 37 | 4.613 | 3.718 | 3.131 | 1.988 | 4.498 | 3.861 | 3.254 | 2.377 |
| 38 | 4.437 | 3.769 | 3.090 | 2.298 | 4.504 | 3.747 | 3.158 | 2.481 |
| 39 | 4.461 | 3.722 | 3.174 | 2.238 | 4.790 | 3.988 | 3.157 | 2.834 |
| 40 | 4.427 | 3.561 | 3.122 | 2.218 | 4.850 | 4.006 | 3.307 | 2.015 |
| 41 | 4.651 | 3.878 | 3.213 | 2.395 | 4.889 | 4.202 | 3.667 | 2.518 |
| 42 | 5.060 | 4.049 | 3.267 | 2.548 | 5.012 | 4.004 | 3.561 | 3.147 |
| 43 | 4.730 | 4.325 | 3.036 | 2.344 | 5.093 | 4.387 | 3.183 | 3.366 |
| 44 | 5.061 | 4.735 | 3.477 | 2.430 | 5.056 | 4.640 | 3.465 | 2.799 |
| 45 | 4.702 | 4.103 | 3.500 | 2.249 | 5.259 | 4.302 | 3.872 | 2.448 |
| 46 | 4.931 | 4.077 | 3.528 | 2.861 | 5.205 | 4.240 | 3.450 | 2.886 |
| 47 | 5.284 | 4.204 | 3.489 | 2.785 | 5.435 | 4.504 | 3.773 | 2.489 |
| 48 | 4.881 | 4.202 | 3.492 | 2.618 | 5.175 | 4.491 | 3.727 | 2.780 |
| 49 | 4.762 | 4.586 | 3.375 | 2.900 | 5.387 | 4.624 | 3.857 | 2.085 |

(C) Cumulative total fertility rate (CTFR), recorded in the National Family Health Survey wave 3, 2005-2006 [7].

| Urban/Rural status: | Urban | | | | Rural | | | |
| --- | --- | --- | --- | --- | --- | --- | --- | --- |
| Educational attainment: | 0 | 1 | 2 | 3 | 0 | 1 | 2 | 3 |
| Age of mother | CTFR | | | | | | | |
| 16 | 0.141 | 0.073 | 0.005 | 0.000 | 0.123 | 0.082 | 0.016 | 0.000 |
| 17 | 0.115 | 0.074 | 0.015 | 0.000 | 0.259 | 0.181 | 0.057 | 0.000 |
| 18 | 0.382 | 0.230 | 0.076 | 0.000 | 0.445 | 0.319 | 0.142 | 0.018 |
| 19 | 0.721 | 0.425 | 0.178 | 0.007 | 0.818 | 0.515 | 0.336 | 0.029 |
| 20 | 0.947 | 0.722 | 0.424 | 0.018 | 1.037 | 0.854 | 0.500 | 0.116 |
| 21 | 1.328 | 0.971 | 0.598 | 0.057 | 1.539 | 1.203 | 0.761 | 0.150 |
| 22 | 1.464 | 1.469 | 0.833 | 0.104 | 1.760 | 1.537 | 1.048 | 0.216 |
| 23 | 1.888 | 1.609 | 1.056 | 0.249 | 2.195 | 1.703 | 1.217 | 0.428 |
| 24 | 2.396 | 1.775 | 1.244 | 0.355 | 2.517 | 2.074 | 1.554 | 0.516 |
| 25 | 2.445 | 1.953 | 1.409 | 0.480 | 2.535 | 2.143 | 1.720 | 0.675 |
| 26 | 2.801 | 2.243 | 1.646 | 0.669 | 2.973 | 2.473 | 1.919 | 0.837 |
| 27 | 3.022 | 2.402 | 1.861 | 0.825 | 3.243 | 2.578 | 1.991 | 1.003 |
| 28 | 2.983 | 2.451 | 1.996 | 0.945 | 3.334 | 2.674 | 2.204 | 1.380 |
| 29 | 3.364 | 2.545 | 2.073 | 1.090 | 3.512 | 2.852 | 2.274 | 1.393 |
| 30 | 3.432 | 2.561 | 2.145 | 1.308 | 3.575 | 2.957 | 2.395 | 1.662 |
| 31 | 3.777 | 3.021 | 2.324 | 1.461 | 3.905 | 2.965 | 2.573 | 1.591 |
| 32 | 3.595 | 3.070 | 2.211 | 1.467 | 3.949 | 3.232 | 2.650 | 1.671 |
| 33 | 3.661 | 3.322 | 2.300 | 1.603 | 4.019 | 3.038 | 2.697 | 1.785 |
| 34 | 3.799 | 3.060 | 2.391 | 1.619 | 4.155 | 3.363 | 2.761 | 1.582 |
| 35 | 3.513 | 3.012 | 2.342 | 1.650 | 3.964 | 3.076 | 2.700 | 1.843 |
| 36 | 4.036 | 3.313 | 2.395 | 1.783 | 4.456 | 3.345 | 2.843 | 1.992 |
| 37 | 3.973 | 3.485 | 2.597 | 1.786 | 4.545 | 3.360 | 2.860 | 1.934 |
| 38 | 4.191 | 3.272 | 2.412 | 1.895 | 4.586 | 3.567 | 3.007 | 1.841 |
| 39 | 4.473 | 3.386 | 2.733 | 1.991 | 4.604 | 3.717 | 2.963 | 1.948 |
| 40 | 4.090 | 3.248 | 2.657 | 2.007 | 4.422 | 3.451 | 3.036 | 2.831 |
| 41 | 4.380 | 3.307 | 2.693 | 1.901 | 4.584 | 3.613 | 3.012 | 2.254 |
| 42 | 4.529 | 3.522 | 2.794 | 1.686 | 4.793 | 3.755 | 2.816 | 2.099 |
| 43 | 4.510 | 3.367 | 2.631 | 2.078 | 4.868 | 4.182 | 3.116 | 2.478 |
| 44 | 4.563 | 3.600 | 2.693 | 2.269 | 5.016 | 3.838 | 2.942 | 2.162 |
| 45 | 4.305 | 3.395 | 2.846 | 1.975 | 4.650 | 3.718 | 3.309 | 1.813 |
| 46 | 4.678 | 3.508 | 2.786 | 2.017 | 4.923 | 3.866 | 3.004 | 2.608 |
| 47 | 4.644 | 3.579 | 2.703 | 2.022 | 5.130 | 3.915 | 2.645 | 2.095 |
| 48 | 4.841 | 3.800 | 3.084 | 2.165 | 4.885 | 4.019 | 3.365 | 2.217 |
| 49 | 4.989 | 3.498 | 3.207 | 2.160 | 5.173 | 4.352 | 3.379 | 2.781 |

## 7 Table AF2. Mortality data.

Estimated death rates by age, year, urban/rural residence and education [8]. Educational categories are: 0: none; 1: 1 to 6 years; 2: >6 to 12 years; 3: >12 years.

1. Urban, educational category 0

|  | Years | | | | | | | | | | | |
| --- | --- | --- | --- | --- | --- | --- | --- | --- | --- | --- | --- | --- |
| Age | 1993 | 1996 | 1997 | 2000 | 2001 | 2002 | 2003 | 2004 | 2005 | 2006 | 2007 | 2008 |
| <1 | 0.0727 | 0.0687 | 0.0658 | 0.0638 | 0.0646 | 0.0641 | 0.0646 | 0.0632 | 0.0633 | 0.0599 | 0.0599 | 0.0573 |
| 1-<5 | 0.0079 | 0.0076 | 0.0073 | 0.0051 | 0.0048 | 0.0046 | 0.0044 | 0.0042 | 0.0043 | 0.0042 | 0.0039 | 0.0036 |
| 5-<10 | 0.0018 | 0.0021 | 0.0021 | 0.0016 | 0.0013 | 0.0013 | 0.0012 | 0.0012 | 0.0012 | 0.0012 | 0.0011 | 0.0010 |
| 10-<15 | 0.0013 | 0.0012 | 0.0011 | 0.0011 | 0.0011 | 0.0011 | 0.0011 | 0.0011 | 0.0010 | 0.0010 | 0.0010 | 0.0010 |
| 15-<20 | 0.0019 | 0.0020 | 0.0019 | 0.0017 | 0.0019 | 0.0019 | 0.0020 | 0.0020 | 0.0021 | 0.0019 | 0.0017 | 0.0016 |
| 20-<25 | 0.0027 | 0.0023 | 0.0023 | 0.0025 | 0.0023 | 0.0020 | 0.0021 | 0.0020 | 0.0019 | 0.0022 | 0.0021 | 0.0020 |
| 25-<30 | 0.0027 | 0.0026 | 0.0024 | 0.0028 | 0.0027 | 0.0025 | 0.0024 | 0.0022 | 0.0022 | 0.0021 | 0.0020 | 0.0021 |
| 30-<35 | 0.0030 | 0.0028 | 0.0025 | 0.0023 | 0.0023 | 0.0023 | 0.0023 | 0.0024 | 0.0025 | 0.0023 | 0.0022 | 0.0023 |
| 35-<40 | 0.0033 | 0.0030 | 0.0031 | 0.0031 | 0.0031 | 0.0028 | 0.0028 | 0.0026 | 0.0026 | 0.0025 | 0.0027 | 0.0027 |
| 40-<45 | 0.0039 | 0.0039 | 0.0041 | 0.0040 | 0.0038 | 0.0036 | 0.0037 | 0.0029 | 0.0028 | 0.0030 | 0.0029 | 0.0029 |
| 45-<50 | 0.0053 | 0.0059 | 0.0064 | 0.0056 | 0.0052 | 0.0051 | 0.0050 | 0.0056 | 0.0056 | 0.0059 | 0.0057 | 0.0059 |
| 50-<55 | 0.0094 | 0.0099 | 0.0102 | 0.0102 | 0.0099 | 0.0095 | 0.0092 | 0.0083 | 0.0081 | 0.0081 | 0.0081 | 0.0081 |
| 55-<60 | 0.0151 | 0.0161 | 0.0171 | 0.0168 | 0.0157 | 0.0157 | 0.0143 | 0.0138 | 0.0137 | 0.0142 | 0.0137 | 0.0140 |
| 60-<65 | 0.0269 | 0.0281 | 0.0317 | 0.0248 | 0.0238 | 0.0243 | 0.0238 | 0.0243 | 0.0248 | 0.0255 | 0.0255 | 0.0253 |
| >65 | 0.0393 | 0.0394 | 0.0451 | 0.0443 | 0.0431 | 0.0421 | 0.0419 | 0.0401 | 0.0377 | 0.0378 | 0.0384 | 0.0393 |

1. Urban, educational category 1

|  | Years | | | | | | | | | | | |
| --- | --- | --- | --- | --- | --- | --- | --- | --- | --- | --- | --- | --- |
| Age | 1993 | 1996 | 1997 | 2000 | 2001 | 2002 | 2003 | 2004 | 2005 | 2006 | 2007 | 2008 |
| <1 | 0.0727 | 0.0687 | 0.0658 | 0.0638 | 0.0646 | 0.0641 | 0.0646 | 0.0632 | 0.0633 | 0.0599 | 0.0599 | 0.0573 |
| 1-<5 | 0.0079 | 0.0076 | 0.0073 | 0.0051 | 0.0048 | 0.0046 | 0.0044 | 0.0042 | 0.0043 | 0.0042 | 0.0039 | 0.0036 |
| 5-<10 | 0.0018 | 0.0021 | 0.0021 | 0.0016 | 0.0013 | 0.0013 | 0.0012 | 0.0012 | 0.0012 | 0.0012 | 0.0011 | 0.0010 |
| 10-<15 | 0.0013 | 0.0012 | 0.0011 | 0.0011 | 0.0011 | 0.0011 | 0.0011 | 0.0011 | 0.0010 | 0.0010 | 0.0010 | 0.0010 |
| 15-<20 | 0.0019 | 0.0020 | 0.0019 | 0.0017 | 0.0019 | 0.0019 | 0.0020 | 0.0020 | 0.0021 | 0.0019 | 0.0017 | 0.0016 |
| 20-<25 | 0.0027 | 0.0023 | 0.0023 | 0.0025 | 0.0023 | 0.0020 | 0.0021 | 0.0020 | 0.0019 | 0.0022 | 0.0021 | 0.0020 |
| 25-<30 | 0.0027 | 0.0026 | 0.0024 | 0.0028 | 0.0027 | 0.0025 | 0.0024 | 0.0022 | 0.0022 | 0.0021 | 0.0020 | 0.0021 |
| 30-<35 | 0.0030 | 0.0028 | 0.0025 | 0.0023 | 0.0023 | 0.0023 | 0.0023 | 0.0024 | 0.0025 | 0.0023 | 0.0022 | 0.0023 |
| 35-<40 | 0.0033 | 0.0030 | 0.0031 | 0.0031 | 0.0031 | 0.0028 | 0.0028 | 0.0026 | 0.0026 | 0.0025 | 0.0027 | 0.0027 |
| 40-<45 | 0.0039 | 0.0039 | 0.0041 | 0.0040 | 0.0038 | 0.0036 | 0.0037 | 0.0029 | 0.0028 | 0.0030 | 0.0029 | 0.0029 |
| 45-<50 | 0.0053 | 0.0059 | 0.0064 | 0.0056 | 0.0052 | 0.0051 | 0.0050 | 0.0056 | 0.0056 | 0.0059 | 0.0057 | 0.0059 |
| 50-<55 | 0.0094 | 0.0099 | 0.0102 | 0.0102 | 0.0099 | 0.0095 | 0.0092 | 0.0083 | 0.0081 | 0.0081 | 0.0081 | 0.0081 |
| 55-<60 | 0.0151 | 0.0161 | 0.0171 | 0.0168 | 0.0157 | 0.0157 | 0.0143 | 0.0138 | 0.0137 | 0.0142 | 0.0137 | 0.0140 |
| 60-<65 | 0.0269 | 0.0281 | 0.0317 | 0.0248 | 0.0238 | 0.0243 | 0.0238 | 0.0243 | 0.0248 | 0.0255 | 0.0255 | 0.0253 |
| >65 | 0.0393 | 0.0394 | 0.0451 | 0.0443 | 0.0431 | 0.0421 | 0.0419 | 0.0401 | 0.0377 | 0.0378 | 0.0384 | 0.0393 |

1. Urban, educational category 2

|  | Years | | | | | | | | | | | |
| --- | --- | --- | --- | --- | --- | --- | --- | --- | --- | --- | --- | --- |
| Age | 1993 | 1996 | 1997 | 2000 | 2001 | 2002 | 2003 | 2004 | 2005 | 2006 | 2007 | 2008 |
| <1 | 0.0433 | 0.0409 | 0.0392 | 0.0380 | 0.0385 | 0.0382 | 0.0384 | 0.0376 | 0.0377 | 0.0357 | 0.0357 | 0.0341 |
| 1-<5 | 0.0047 | 0.0045 | 0.0043 | 0.0030 | 0.0028 | 0.0028 | 0.0026 | 0.0025 | 0.0026 | 0.0025 | 0.0023 | 0.0021 |
| 5-<10 | 0.0010 | 0.0012 | 0.0012 | 0.0010 | 0.0008 | 0.0008 | 0.0007 | 0.0007 | 0.0007 | 0.0007 | 0.0007 | 0.0006 |
| 10-<15 | 0.0008 | 0.0007 | 0.0007 | 0.0006 | 0.0007 | 0.0007 | 0.0007 | 0.0006 | 0.0006 | 0.0006 | 0.0006 | 0.0006 |
| 15-<20 | 0.0012 | 0.0012 | 0.0011 | 0.0010 | 0.0011 | 0.0012 | 0.0012 | 0.0012 | 0.0012 | 0.0011 | 0.0010 | 0.0010 |
| 20-<25 | 0.0016 | 0.0014 | 0.0014 | 0.0015 | 0.0014 | 0.0012 | 0.0012 | 0.0012 | 0.0011 | 0.0013 | 0.0013 | 0.0012 |
| 25-<30 | 0.0016 | 0.0015 | 0.0014 | 0.0016 | 0.0016 | 0.0015 | 0.0014 | 0.0013 | 0.0013 | 0.0012 | 0.0012 | 0.0012 |
| 30-<35 | 0.0018 | 0.0017 | 0.0015 | 0.0013 | 0.0014 | 0.0014 | 0.0014 | 0.0014 | 0.0015 | 0.0014 | 0.0013 | 0.0013 |
| 35-<40 | 0.0020 | 0.0018 | 0.0018 | 0.0018 | 0.0018 | 0.0017 | 0.0016 | 0.0016 | 0.0016 | 0.0015 | 0.0016 | 0.0016 |
| 40-<45 | 0.0023 | 0.0023 | 0.0025 | 0.0024 | 0.0022 | 0.0021 | 0.0022 | 0.0017 | 0.0017 | 0.0018 | 0.0017 | 0.0017 |
| 45-<50 | 0.0031 | 0.0035 | 0.0038 | 0.0033 | 0.0031 | 0.0030 | 0.0030 | 0.0033 | 0.0033 | 0.0035 | 0.0034 | 0.0035 |
| 50-<55 | 0.0056 | 0.0059 | 0.0060 | 0.0060 | 0.0059 | 0.0057 | 0.0055 | 0.0050 | 0.0048 | 0.0048 | 0.0048 | 0.0048 |
| 55-<60 | 0.0090 | 0.0096 | 0.0102 | 0.0100 | 0.0094 | 0.0094 | 0.0085 | 0.0082 | 0.0082 | 0.0085 | 0.0081 | 0.0083 |
| 60-<65 | 0.0160 | 0.0167 | 0.0189 | 0.0148 | 0.0141 | 0.0144 | 0.0142 | 0.0145 | 0.0148 | 0.0152 | 0.0152 | 0.0150 |
| >65 | 0.0234 | 0.0235 | 0.0268 | 0.0264 | 0.0256 | 0.0251 | 0.0249 | 0.0239 | 0.0224 | 0.0225 | 0.0229 | 0.0234 |

1. Urban, educational category 3

|  | Years | | | | | | | | | | | |
| --- | --- | --- | --- | --- | --- | --- | --- | --- | --- | --- | --- | --- |
| Age | 1993 | 1996 | 1997 | 2000 | 2001 | 2002 | 2003 | 2004 | 2005 | 2006 | 2007 | 2008 |
| <1 | 0.0299 | 0.0282 | 0.0270 | 0.0262 | 0.0265 | 0.0263 | 0.0265 | 0.0260 | 0.0260 | 0.0246 | 0.0246 | 0.0235 |
| 1-<5 | 0.0032 | 0.0031 | 0.0030 | 0.0021 | 0.0020 | 0.0019 | 0.0018 | 0.0017 | 0.0018 | 0.0017 | 0.0016 | 0.0015 |
| 5-<10 | 0.0007 | 0.0008 | 0.0009 | 0.0007 | 0.0005 | 0.0005 | 0.0005 | 0.0005 | 0.0005 | 0.0005 | 0.0005 | 0.0004 |
| 10-<15 | 0.0005 | 0.0005 | 0.0005 | 0.0004 | 0.0005 | 0.0005 | 0.0005 | 0.0004 | 0.0004 | 0.0004 | 0.0004 | 0.0004 |
| 15-<20 | 0.0008 | 0.0008 | 0.0008 | 0.0007 | 0.0008 | 0.0008 | 0.0008 | 0.0008 | 0.0008 | 0.0008 | 0.0007 | 0.0007 |
| 20-<25 | 0.0011 | 0.0009 | 0.0010 | 0.0010 | 0.0009 | 0.0008 | 0.0008 | 0.0008 | 0.0008 | 0.0009 | 0.0009 | 0.0008 |
| 25-<30 | 0.0011 | 0.0011 | 0.0010 | 0.0011 | 0.0011 | 0.0010 | 0.0010 | 0.0009 | 0.0009 | 0.0009 | 0.0008 | 0.0009 |
| 30-<35 | 0.0012 | 0.0012 | 0.0010 | 0.0009 | 0.0010 | 0.0010 | 0.0009 | 0.0010 | 0.0010 | 0.0009 | 0.0009 | 0.0009 |
| 35-<40 | 0.0013 | 0.0012 | 0.0013 | 0.0013 | 0.0013 | 0.0012 | 0.0011 | 0.0011 | 0.0011 | 0.0010 | 0.0011 | 0.0011 |
| 40-<45 | 0.0016 | 0.0016 | 0.0017 | 0.0016 | 0.0015 | 0.0015 | 0.0015 | 0.0012 | 0.0012 | 0.0012 | 0.0012 | 0.0012 |
| 45-<50 | 0.0022 | 0.0024 | 0.0026 | 0.0023 | 0.0022 | 0.0021 | 0.0021 | 0.0023 | 0.0023 | 0.0024 | 0.0023 | 0.0024 |
| 50-<55 | 0.0039 | 0.0041 | 0.0042 | 0.0042 | 0.0041 | 0.0039 | 0.0038 | 0.0034 | 0.0033 | 0.0033 | 0.0033 | 0.0033 |
| 55-<60 | 0.0062 | 0.0066 | 0.0070 | 0.0069 | 0.0065 | 0.0065 | 0.0059 | 0.0057 | 0.0056 | 0.0058 | 0.0056 | 0.0058 |
| 60-<65 | 0.0111 | 0.0116 | 0.0130 | 0.0102 | 0.0098 | 0.0100 | 0.0098 | 0.0100 | 0.0102 | 0.0105 | 0.0105 | 0.0104 |
| >65 | 0.0162 | 0.0162 | 0.0185 | 0.0182 | 0.0177 | 0.0173 | 0.0172 | 0.0165 | 0.0155 | 0.0155 | 0.0158 | 0.0162 |

1. Rural, educational category 0

|  | Years | | | | | | | | | | | |
| --- | --- | --- | --- | --- | --- | --- | --- | --- | --- | --- | --- | --- |
| Age | 1993 | 1996 | 1997 | 2000 | 2001 | 2002 | 2003 | 2004 | 2005 | 2006 | 2007 | 2008 |
| <1 | 0.1011 | 0.0987 | 0.0967 | 0.0912 | 0.0913 | 0.0908 | 0.0902 | 0.0870 | 0.0843 | 0.0800 | 0.0765 | 0.0730 |
| 1-<5 | 0.0093 | 0.0117 | 0.0115 | 0.0094 | 0.0084 | 0.0080 | 0.0077 | 0.0073 | 0.0073 | 0.0072 | 0.0070 | 0.0067 |
| 5-<10 | 0.0027 | 0.0031 | 0.0031 | 0.0026 | 0.0024 | 0.0022 | 0.0022 | 0.0021 | 0.0020 | 0.0019 | 0.0018 | 0.0017 |
| 10-<15 | 0.0015 | 0.0016 | 0.0016 | 0.0015 | 0.0014 | 0.0014 | 0.0013 | 0.0013 | 0.0013 | 0.0013 | 0.0013 | 0.0012 |
| 15-<20 | 0.0021 | 0.0025 | 0.0025 | 0.0026 | 0.0025 | 0.0024 | 0.0023 | 0.0022 | 0.0021 | 0.0021 | 0.0020 | 0.0019 |
| 20-<25 | 0.0026 | 0.0034 | 0.0029 | 0.0034 | 0.0032 | 0.0031 | 0.0030 | 0.0029 | 0.0028 | 0.0027 | 0.0026 | 0.0026 |
| 25-<30 | 0.0031 | 0.0035 | 0.0032 | 0.0034 | 0.0033 | 0.0032 | 0.0031 | 0.0029 | 0.0027 | 0.0026 | 0.0026 | 0.0025 |
| 30-<35 | 0.0037 | 0.0036 | 0.0035 | 0.0034 | 0.0032 | 0.0032 | 0.0030 | 0.0029 | 0.0028 | 0.0028 | 0.0027 | 0.0027 |
| 35-<40 | 0.0045 | 0.0037 | 0.0035 | 0.0038 | 0.0037 | 0.0037 | 0.0036 | 0.0035 | 0.0032 | 0.0032 | 0.0032 | 0.0032 |
| 40-<45 | 0.0062 | 0.0047 | 0.0047 | 0.0044 | 0.0043 | 0.0041 | 0.0040 | 0.0034 | 0.0034 | 0.0033 | 0.0033 | 0.0033 |
| 45-<50 | 0.0098 | 0.0064 | 0.0063 | 0.0063 | 0.0062 | 0.0061 | 0.0059 | 0.0068 | 0.0065 | 0.0065 | 0.0061 | 0.0061 |
| 50-<55 | 0.0146 | 0.0107 | 0.0106 | 0.0093 | 0.0091 | 0.0084 | 0.0082 | 0.0079 | 0.0078 | 0.0074 | 0.0076 | 0.0077 |
| 55-<60 | 0.0223 | 0.0150 | 0.0153 | 0.0154 | 0.0151 | 0.0146 | 0.0144 | 0.0139 | 0.0135 | 0.0131 | 0.0126 | 0.0122 |
| 60-<65 | 0.0346 | 0.0260 | 0.0255 | 0.0237 | 0.0231 | 0.0226 | 0.0225 | 0.0225 | 0.0227 | 0.0223 | 0.0223 | 0.0220 |
| >65 | 0.0509 | 0.0385 | 0.0383 | 0.0390 | 0.0383 | 0.0369 | 0.0361 | 0.0356 | 0.0348 | 0.0343 | 0.0355 | 0.0361 |

1. Rural, educational category 1

|  | Years | | | | | | | | | | | |
| --- | --- | --- | --- | --- | --- | --- | --- | --- | --- | --- | --- | --- |
| Age | 1993 | 1996 | 1997 | 2000 | 2001 | 2002 | 2003 | 2004 | 2005 | 2006 | 2007 | 2008 |
| <1 | 0.1011 | 0.0987 | 0.0967 | 0.0912 | 0.0913 | 0.0908 | 0.0902 | 0.0870 | 0.0843 | 0.0800 | 0.0765 | 0.0730 |
| 1-<5 | 0.0093 | 0.0117 | 0.0115 | 0.0094 | 0.0084 | 0.0080 | 0.0077 | 0.0073 | 0.0073 | 0.0072 | 0.0070 | 0.0067 |
| 5-<10 | 0.0027 | 0.0031 | 0.0031 | 0.0026 | 0.0024 | 0.0022 | 0.0022 | 0.0021 | 0.0020 | 0.0019 | 0.0018 | 0.0017 |
| 10-<15 | 0.0015 | 0.0016 | 0.0016 | 0.0015 | 0.0014 | 0.0014 | 0.0013 | 0.0013 | 0.0013 | 0.0013 | 0.0013 | 0.0012 |
| 15-<20 | 0.0021 | 0.0025 | 0.0025 | 0.0026 | 0.0025 | 0.0024 | 0.0023 | 0.0022 | 0.0021 | 0.0021 | 0.0020 | 0.0019 |
| 20-<25 | 0.0026 | 0.0034 | 0.0029 | 0.0034 | 0.0032 | 0.0031 | 0.0030 | 0.0029 | 0.0028 | 0.0027 | 0.0026 | 0.0026 |
| 25-<30 | 0.0031 | 0.0035 | 0.0032 | 0.0034 | 0.0033 | 0.0032 | 0.0031 | 0.0029 | 0.0027 | 0.0026 | 0.0026 | 0.0025 |
| 30-<35 | 0.0037 | 0.0036 | 0.0035 | 0.0034 | 0.0032 | 0.0032 | 0.0030 | 0.0029 | 0.0028 | 0.0028 | 0.0027 | 0.0027 |
| 35-<40 | 0.0045 | 0.0037 | 0.0035 | 0.0038 | 0.0037 | 0.0037 | 0.0036 | 0.0035 | 0.0032 | 0.0032 | 0.0032 | 0.0032 |
| 40-<45 | 0.0062 | 0.0047 | 0.0047 | 0.0044 | 0.0043 | 0.0041 | 0.0040 | 0.0034 | 0.0034 | 0.0033 | 0.0033 | 0.0033 |
| 45-<50 | 0.0098 | 0.0064 | 0.0063 | 0.0063 | 0.0062 | 0.0061 | 0.0059 | 0.0068 | 0.0065 | 0.0065 | 0.0061 | 0.0061 |
| 50-<55 | 0.0146 | 0.0107 | 0.0106 | 0.0093 | 0.0091 | 0.0084 | 0.0082 | 0.0079 | 0.0078 | 0.0074 | 0.0076 | 0.0077 |
| 55-<60 | 0.0223 | 0.0150 | 0.0153 | 0.0154 | 0.0151 | 0.0146 | 0.0144 | 0.0139 | 0.0135 | 0.0131 | 0.0126 | 0.0122 |
| 60-<65 | 0.0346 | 0.0260 | 0.0255 | 0.0237 | 0.0231 | 0.0226 | 0.0225 | 0.0225 | 0.0227 | 0.0223 | 0.0223 | 0.0220 |
| >65 | 0.0509 | 0.0385 | 0.0383 | 0.0390 | 0.0383 | 0.0369 | 0.0361 | 0.0356 | 0.0348 | 0.0343 | 0.0355 | 0.0361 |

1. Rural, educational category 2

|  | Years | | | | | | | | | | | |
| --- | --- | --- | --- | --- | --- | --- | --- | --- | --- | --- | --- | --- |
| Age | 1993 | 1996 | 1997 | 2000 | 2001 | 2002 | 2003 | 2004 | 2005 | 2006 | 2007 | 2008 |
| <1 | 0.0736 | 0.0718 | 0.0704 | 0.0664 | 0.0665 | 0.0661 | 0.0657 | 0.0633 | 0.0614 | 0.0582 | 0.0557 | 0.0531 |
| 1-<5 | 0.0068 | 0.0085 | 0.0083 | 0.0068 | 0.0061 | 0.0058 | 0.0056 | 0.0053 | 0.0053 | 0.0053 | 0.0051 | 0.0049 |
| 5-<10 | 0.0020 | 0.0022 | 0.0022 | 0.0019 | 0.0018 | 0.0016 | 0.0016 | 0.0015 | 0.0015 | 0.0014 | 0.0013 | 0.0012 |
| 10-<15 | 0.0011 | 0.0011 | 0.0012 | 0.0011 | 0.0011 | 0.0010 | 0.0010 | 0.0009 | 0.0009 | 0.0009 | 0.0009 | 0.0009 |
| 15-<20 | 0.0015 | 0.0018 | 0.0018 | 0.0019 | 0.0018 | 0.0017 | 0.0017 | 0.0016 | 0.0016 | 0.0015 | 0.0015 | 0.0014 |
| 20-<25 | 0.0019 | 0.0025 | 0.0021 | 0.0025 | 0.0024 | 0.0023 | 0.0022 | 0.0021 | 0.0020 | 0.0020 | 0.0019 | 0.0019 |
| 25-<30 | 0.0022 | 0.0025 | 0.0023 | 0.0024 | 0.0024 | 0.0023 | 0.0022 | 0.0021 | 0.0020 | 0.0019 | 0.0019 | 0.0018 |
| 30-<35 | 0.0027 | 0.0026 | 0.0025 | 0.0025 | 0.0023 | 0.0023 | 0.0021 | 0.0021 | 0.0020 | 0.0021 | 0.0020 | 0.0020 |
| 35-<40 | 0.0033 | 0.0027 | 0.0026 | 0.0028 | 0.0027 | 0.0027 | 0.0026 | 0.0026 | 0.0023 | 0.0023 | 0.0023 | 0.0023 |
| 40-<45 | 0.0045 | 0.0034 | 0.0034 | 0.0032 | 0.0031 | 0.0030 | 0.0029 | 0.0025 | 0.0024 | 0.0024 | 0.0024 | 0.0024 |
| 45-<50 | 0.0071 | 0.0047 | 0.0046 | 0.0046 | 0.0045 | 0.0045 | 0.0043 | 0.0050 | 0.0047 | 0.0047 | 0.0044 | 0.0044 |
| 50-<55 | 0.0106 | 0.0078 | 0.0078 | 0.0068 | 0.0066 | 0.0061 | 0.0060 | 0.0057 | 0.0056 | 0.0054 | 0.0055 | 0.0056 |
| 55-<60 | 0.0162 | 0.0109 | 0.0111 | 0.0112 | 0.0110 | 0.0107 | 0.0105 | 0.0101 | 0.0098 | 0.0095 | 0.0092 | 0.0089 |
| 60-<65 | 0.0252 | 0.0189 | 0.0186 | 0.0173 | 0.0168 | 0.0165 | 0.0164 | 0.0163 | 0.0165 | 0.0162 | 0.0162 | 0.0160 |
| >65 | 0.0370 | 0.0280 | 0.0279 | 0.0284 | 0.0279 | 0.0269 | 0.0263 | 0.0259 | 0.0254 | 0.0249 | 0.0259 | 0.0263 |

1. Rural, educational category 3

|  | Years | | | | | | | | | | | |
| --- | --- | --- | --- | --- | --- | --- | --- | --- | --- | --- | --- | --- |
| Age | 1993 | 1996 | 1997 | 2000 | 2001 | 2002 | 2003 | 2004 | 2005 | 2006 | 2007 | 2008 |
| <1 | 0.0527 | 0.0514 | 0.0504 | 0.0475 | 0.0475 | 0.0473 | 0.0470 | 0.0453 | 0.0439 | 0.0416 | 0.0399 | 0.0380 |
| 1-<5 | 0.0048 | 0.0061 | 0.0060 | 0.0049 | 0.0044 | 0.0042 | 0.0040 | 0.0038 | 0.0038 | 0.0038 | 0.0036 | 0.0035 |
| 5-<10 | 0.0014 | 0.0016 | 0.0016 | 0.0014 | 0.0013 | 0.0012 | 0.0011 | 0.0011 | 0.0010 | 0.0010 | 0.0010 | 0.0009 |
| 10-<15 | 0.0008 | 0.0008 | 0.0008 | 0.0008 | 0.0008 | 0.0007 | 0.0007 | 0.0007 | 0.0007 | 0.0007 | 0.0007 | 0.0006 |
| 15-<20 | 0.0011 | 0.0013 | 0.0013 | 0.0013 | 0.0013 | 0.0012 | 0.0012 | 0.0011 | 0.0011 | 0.0011 | 0.0010 | 0.0010 |
| 20-<25 | 0.0014 | 0.0018 | 0.0015 | 0.0018 | 0.0017 | 0.0016 | 0.0016 | 0.0015 | 0.0014 | 0.0014 | 0.0014 | 0.0013 |
| 25-<30 | 0.0016 | 0.0018 | 0.0017 | 0.0017 | 0.0017 | 0.0016 | 0.0016 | 0.0015 | 0.0014 | 0.0014 | 0.0013 | 0.0013 |
| 30-<35 | 0.0019 | 0.0019 | 0.0018 | 0.0018 | 0.0017 | 0.0016 | 0.0015 | 0.0015 | 0.0015 | 0.0015 | 0.0014 | 0.0014 |
| 35-<40 | 0.0024 | 0.0019 | 0.0018 | 0.0020 | 0.0019 | 0.0019 | 0.0018 | 0.0018 | 0.0017 | 0.0017 | 0.0017 | 0.0017 |
| 40-<45 | 0.0032 | 0.0025 | 0.0025 | 0.0023 | 0.0022 | 0.0021 | 0.0021 | 0.0018 | 0.0017 | 0.0017 | 0.0017 | 0.0017 |
| 45-<50 | 0.0051 | 0.0034 | 0.0033 | 0.0033 | 0.0032 | 0.0032 | 0.0031 | 0.0036 | 0.0034 | 0.0034 | 0.0032 | 0.0032 |
| 50-<55 | 0.0076 | 0.0056 | 0.0055 | 0.0049 | 0.0047 | 0.0044 | 0.0043 | 0.0041 | 0.0040 | 0.0039 | 0.0039 | 0.0040 |
| 55-<60 | 0.0116 | 0.0078 | 0.0080 | 0.0080 | 0.0078 | 0.0076 | 0.0075 | 0.0072 | 0.0070 | 0.0068 | 0.0066 | 0.0064 |
| 60-<65 | 0.0180 | 0.0135 | 0.0133 | 0.0124 | 0.0120 | 0.0118 | 0.0117 | 0.0117 | 0.0118 | 0.0116 | 0.0116 | 0.0115 |
| >65 | 0.0265 | 0.0201 | 0.0200 | 0.0203 | 0.0199 | 0.0192 | 0.0188 | 0.0185 | 0.0181 | 0.0178 | 0.0185 | 0.0188 |

## 8 Table AF3. Educational attainment data.

Proportion of females aged 20-24 years old in each educational attainment category [5–7,9]. Educational categories are: 0: none; 1: 1 to 6 years; 2: >6 to 12 years; 3: >12 years.

1. Urban

|  | Year | | | |
| --- | --- | --- | --- | --- |
| Educational attainment category | 1992-1993 | 1998-1999 | 2005-2006 | 2008-2009 |
| 0 | 0.332 | 0.332 | 0.248 | 0.192 |
| 1 | 0.140 | 0.140 | 0.146 | 0.118 |
| 2 | 0.448 | 0.448 | 0.506 | 0.534 |
| 3 | 0.081 | 0.081 | 0.100 | 0.156 |

1. Rural

|  | Year | | | |
| --- | --- | --- | --- | --- |
| Educational attainment category | 1992-1993 | 1998-1999 | 2005-2006 | 2008-2009 |
| 0 | 0.661 | 0.552 | 0.397 | 0.414 |
| 1 | 0.128 | 0.158 | 0.155 | 0.164 |
| 2 | 0.201 | 0.275 | 0.391 | 0.388 |
| 3 | 0.010 | 0.015 | 0.057 | 0.035 |

## 9 Table AF4. Migration data.

The proportion of women currently residing in an urban or rural zone who report having lived in a rural or urban zone within the last several years [6,7].

1. as of 1998, among women aged 20-24 years old:

| Current residence  (0: rural, 1: urban) | Education category | Time of migration  (within X years) | % who changed zones  (rural to urban or vice versa) |
| --- | --- | --- | --- |
| 0 | 0 | 6 | 0.84% |
| 0 | 0 | 12 | 3.00% |
| 0 | 0 | 22 | 3.94% |
| 0 | 1 | 6 | 2.55% |
| 0 | 1 | 12 | 6.84% |
| 0 | 1 | 22 | 8.38% |
| 0 | 2 | 6 | 6.02% |
| 0 | 2 | 12 | 13.80% |
| 0 | 2 | 22 | 15.56% |
| 0 | 3 | 6 | 33.03% |
| 0 | 3 | 12 | 41.79% |
| 0 | 3 | 22 | 42.10% |
| 1 | 0 | 6 | 15.66% |
| 1 | 0 | 12 | 41.54% |
| 1 | 0 | 22 | 56.90% |
| 1 | 1 | 6 | 13.75% |
| 1 | 1 | 12 | 39.19% |
| 1 | 1 | 22 | 46.85% |
| 1 | 2 | 6 | 15.25% |
| 1 | 2 | 12 | 32.46% |
| 1 | 2 | 22 | 35.15% |
| 1 | 3 | 6 | 11.75% |
| 1 | 3 | 12 | 14.27% |
| 1 | 3 | 22 | 15.18% |

1. as of 1998, among women aged 25-29 years old:

| Current residence  (0: rural, 1: urban) | Education category | Time of migration  (within X years) | % who changed zones  (rural to urban or vice versa) |
| --- | --- | --- | --- |
| 0 | 0 | 6 | 0.86% |
| 0 | 0 | 12 | 1.62% |
| 0 | 0 | 27 | 4.86% |
| 0 | 1 | 6 | 1.91% |
| 0 | 1 | 12 | 4.25% |
| 0 | 1 | 27 | 10.47% |
| 0 | 2 | 6 | 3.52% |
| 0 | 2 | 12 | 9.67% |
| 0 | 2 | 27 | 17.30% |
| 0 | 3 | 6 | 14.44% |
| 0 | 3 | 12 | 27.03% |
| 0 | 3 | 27 | 30.15% |
| 1 | 0 | 6 | 8.51% |
| 1 | 0 | 12 | 20.62% |
| 1 | 0 | 27 | 57.36% |
| 1 | 1 | 6 | 7.46% |
| 1 | 1 | 12 | 20.89% |
| 1 | 1 | 27 | 45.72% |
| 1 | 2 | 6 | 6.61% |
| 1 | 2 | 12 | 20.81% |
| 1 | 2 | 27 | 32.31% |
| 1 | 3 | 6 | 5.19% |
| 1 | 3 | 12 | 10.29% |
| 1 | 3 | 27 | 12.56% |

1. as of 2005, among women aged 20-24 years old:

| Current residence  (0: rural, 1: urban) | Education category | Time of migration  (within X years) | % who changed zones  (rural to urban or vice versa) |
| --- | --- | --- | --- |
| 0 | 0 | 6 | 2.28% |
| 0 | 0 | 12 | 6.02% |
| 0 | 0 | 22 | 7.50% |
| 0 | 1 | 6 | 3.71% |
| 0 | 1 | 12 | 8.84% |
| 0 | 1 | 22 | 10.55% |
| 0 | 2 | 6 | 7.79% |
| 0 | 2 | 12 | 15.30% |
| 0 | 2 | 22 | 16.55% |
| 0 | 3 | 6 | 17.46% |
| 0 | 3 | 12 | 23.67% |
| 0 | 3 | 22 | 24.62% |
| 1 | 0 | 6 | 16.25% |
| 1 | 0 | 12 | 41.48% |
| 1 | 0 | 22 | 51.85% |
| 1 | 1 | 6 | 12.10% |
| 1 | 1 | 12 | 36.34% |
| 1 | 1 | 22 | 41.79% |
| 1 | 2 | 6 | 14.95% |
| 1 | 2 | 12 | 29.26% |
| 1 | 2 | 22 | 31.42% |
| 1 | 3 | 6 | 9.99% |
| 1 | 3 | 12 | 12.62% |
| 1 | 3 | 22 | 13.46% |

1. as of 2005, among women aged 25-29 years old:

| Current residence  (0: rural, 1: urban) | Education category | Time of migration  (within X years) | % who changed zones  (rural to urban or vice versa) |
| --- | --- | --- | --- |
| 0 | 0 | 6 | 2.28% |
| 0 | 0 | 12 | 6.02% |
| 0 | 0 | 27 | 7.50% |
| 0 | 1 | 6 | 3.71% |
| 0 | 1 | 12 | 8.84% |
| 0 | 1 | 27 | 10.55% |
| 0 | 2 | 6 | 7.79% |
| 0 | 2 | 12 | 15.30% |
| 0 | 2 | 27 | 16.55% |
| 0 | 3 | 6 | 17.46% |
| 0 | 3 | 12 | 23.67% |
| 0 | 3 | 27 | 24.62% |
| 1 | 0 | 6 | 16.25% |
| 1 | 0 | 12 | 41.48% |
| 1 | 0 | 27 | 51.85% |
| 1 | 1 | 6 | 12.10% |
| 1 | 1 | 12 | 36.34% |
| 1 | 1 | 27 | 41.79% |
| 1 | 2 | 6 | 14.95% |
| 1 | 2 | 12 | 29.26% |
| 1 | 2 | 27 | 31.42% |
| 1 | 3 | 6 | 9.99% |
| 1 | 3 | 12 | 12.62% |
| 1 | 3 | 27 | 13.46% |

## 10 Table AF5. Population size data.

Estimates of total Indian female population size by urban/rural residence, in 1000’s [10].

| Year | Urban | Rural |
| --- | --- | --- |
| 1992 | 113008 | 322125 |
| 1993 | 116125 | 327389 |
| 1994 | 119276 | 332613 |
| 1995 | 122463 | 337804 |
| 1996 | 125685 | 342957 |
| 1997 | 128938 | 348064 |
| 1998 | 132225 | 353130 |
| 1999 | 135548 | 358161 |
| 2000 | 138906 | 363159 |
| 2001 | 142822 | 367610 |
| 2002 | 146789 | 372007 |
| 2003 | 150796 | 376318 |
| 2004 | 154824 | 380503 |
| 2005 | 158862 | 384534 |
| 2006 | 163040 | 388256 |
| 2007 | 167227 | 391817 |
| 2008 | 171433 | 395249 |
| 2009 | 175678 | 398602 |
| 2010 | 179976 | 401905 |

## 11 Table AF6. Fitted model parameters.

Summary statistics on posterior joint distributions of the fitted model parameters, which are also visually displayed in Figure AF1. Geweke is a test for a null hypothesis that the chain has converged (as shown, demonstrates convergence) [4]. RR: relative risk.

| Parameter | Definition | Mean | Std dev. | Geweke |
| --- | --- | --- | --- | --- |
| 1 | Cumulative total fertility rate, urban ed category 0, year 1992 (reference group) | 3.884 | 0.0003430 | 0.9998 |
| 2 | Median age of fertility, ref group | 25.582 | 0.0006441 | 0.9999 |
| 3 | Length of the age interval, ref group | 21.160 | 0.0004353 | 0.9999 |
| 4 | RR fertility urban ed cat 1 vs ref group | 0.837 | 0.0002408 | 0.9999 |
| 5 | RR fertility urban ed cat 2 vs ref group | 0.648 | 0.0008320 | 0.9974 |
| 6 | RR fertility urban ed cat 3 vs ref group | 0.429 | 0.0018994 | 0.9910 |
| 7 | RR fertility rural ed cat 0 vs ref group | 1.056 | 0.0003004 | 0.9994 |
| 8 | RR fertility rural ed cat 1 vs ref group | 0.884 | 0.0005077 | 0.9989 |
| 9 | RR fertility rural ed cat 2 vs ref group | 0.720 | 0.0019655 | 0.9943 |
| 10 | RR fertility rural ed cat 3 vs ref group | 0.434 | 0.0005240 | 0.9975 |
| 11 | Secular trend in cumulative total fertility rate | -0.001 | 0.0000121 | 0.9896 |
| 12 | Mortality model constant, ages <1, ref group | 31.098 | 0.0007677 | 0.9999 |
| 13 | Mortality model parameter multiplied by calendar year, ages <1, ref group | -0.017 | 0.0000162 | 0.9992 |
| 14 | Mortality model constant, ages 1-10, ref group | 64.218 | 0.0000230 | 0.9999 |
| 15 | Mortality model parameter multiplied by age, ages 1-10, ref group | -0.338 | 0.0003625 | 0.9978 |
| 16 | Mortality model parameter multiplied by calendar year, ages >10, ref group | -0.035 | 0.0000230 | 0.9993 |
| 17 | Mortality model constant, ages >10, ref group | 21.140 | 0.0002711 | 0.9999 |
| 18 | Mortality model parameter multiplied by age, ages >10, ref group | 0.058 | 0.0009145 | 0.9676 |
| 19 | Mortality model parameter multiplied by calendar year, ages >10, ref group | -0.014 | 0.0000183 | 0.9979 |
| 20 | RR death, urban ed cat 1 vs ref group | 0.912 | 0.0003319 | 0.9992 |
| 21 | RR death, urban ed cat 2 vs ref group | 0.659 | 0.0001221 | 0.9997 |
| 22 | RR death, urban ed cat 3 vs ref group | 0.485 | 0.0005366 | 0.9977 |
| 23 | RR death, rural vs urban | 1.340 | 0.0000169 | 0.9999 |
| 24 | Annual proportion of pop moving from ed cat 0 to 1 | 0.012 | 0.0001088 | 0.9848 |
| 25 | Annual proportion of pop moving from ed cat 1 to 2 | 0.024 | 0.0010627 | 0.9093 |
| 26 | Annual proportion of pop moving from ed cat 2 to 3 | 0.027 | 0.0000847 | 0.9933 |
| 27 | Net annual rural-to-urban migration probability, reference group | 0.026 | 0.0081102 | 0.9758 |
| 28 | RR migration with increase in each ed category | 1.172 | 0.0003721 | 0.9758 |

## 12 Table AF7. Comparison of models.

Comparisons of three calibrated models reveal that one incorporating secular trends is more consistent with the observed data. A model incorporating both education and migration rates best explains the variance in the data, even when penalizing the use of more parameters using the deviance information criterion (DIC). Note that lower DIC scores are considered better (reflecting better fit to data and fewer parameters to accomplish the fitting), and a >10 point difference is considered meaningful (Bolker 2008).

| **Model** | **Components** | ***Δ*DIC when fit against Table 1 data sources** |
| --- | --- | --- |
| **1** | Age, sex, urban/rural residence, fertility, mortality | Reference |
| **2** | Age, sex, urban/rural residence, fertility, mortality, educational attainment | +5.2 versus model 1 |
| **3** | Age, sex, urban/rural residence, fertility, mortality, educational attainment, migration | -259.1 versus model 2 |

## 13 Table AF8. Relative risk of death, by education level

The estimated relative risk of death declines significantly with education. Estimated mean relative risk of death by educational attainment category is listed with 95% confidence intervals in parentheses.

| **Educational level** | **RR of death – urban** | **RR of death – rural** |
| --- | --- | --- |
| 0 years | 0.92 (0.87-0.97) | 1.00 (referent) |
| >0-6 years | 0.76 (0.62-0.90) | 0.89 (0.85-0.95) |
| >6-12 years | 0.55 (0.48-0.63) | 0.72 (0.68-0.76) |
| >12 years | 0.38 (0.19-0.56) | 0.56 (0.41-0.71) |

## 14 Figure AF1: MCMC results.

## (A) Probability distributions of fitted parameters. Parameter labels (numbers above each graph) correspond to column 1 of Table AF6.


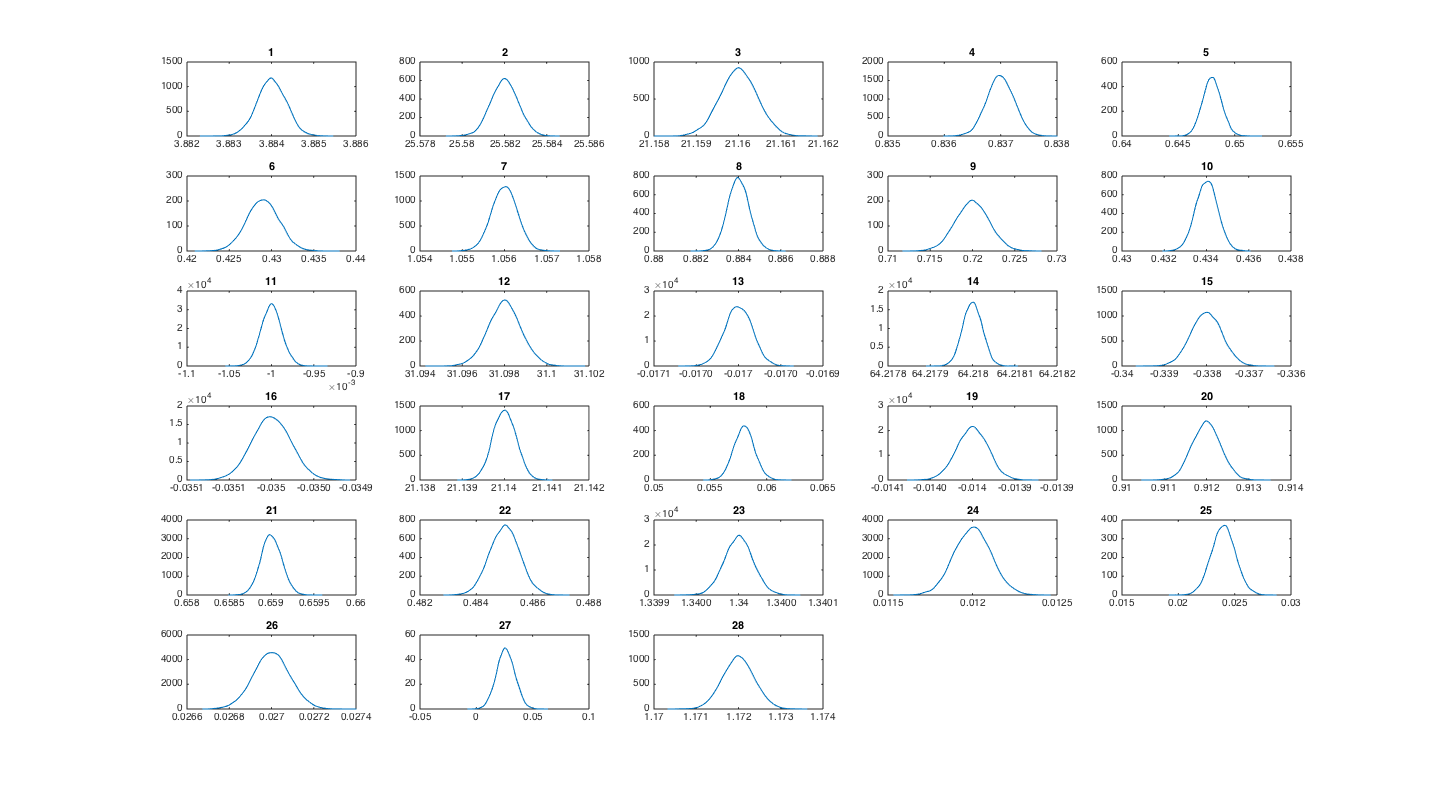


## (B) Traceplots of MCMC iterations.


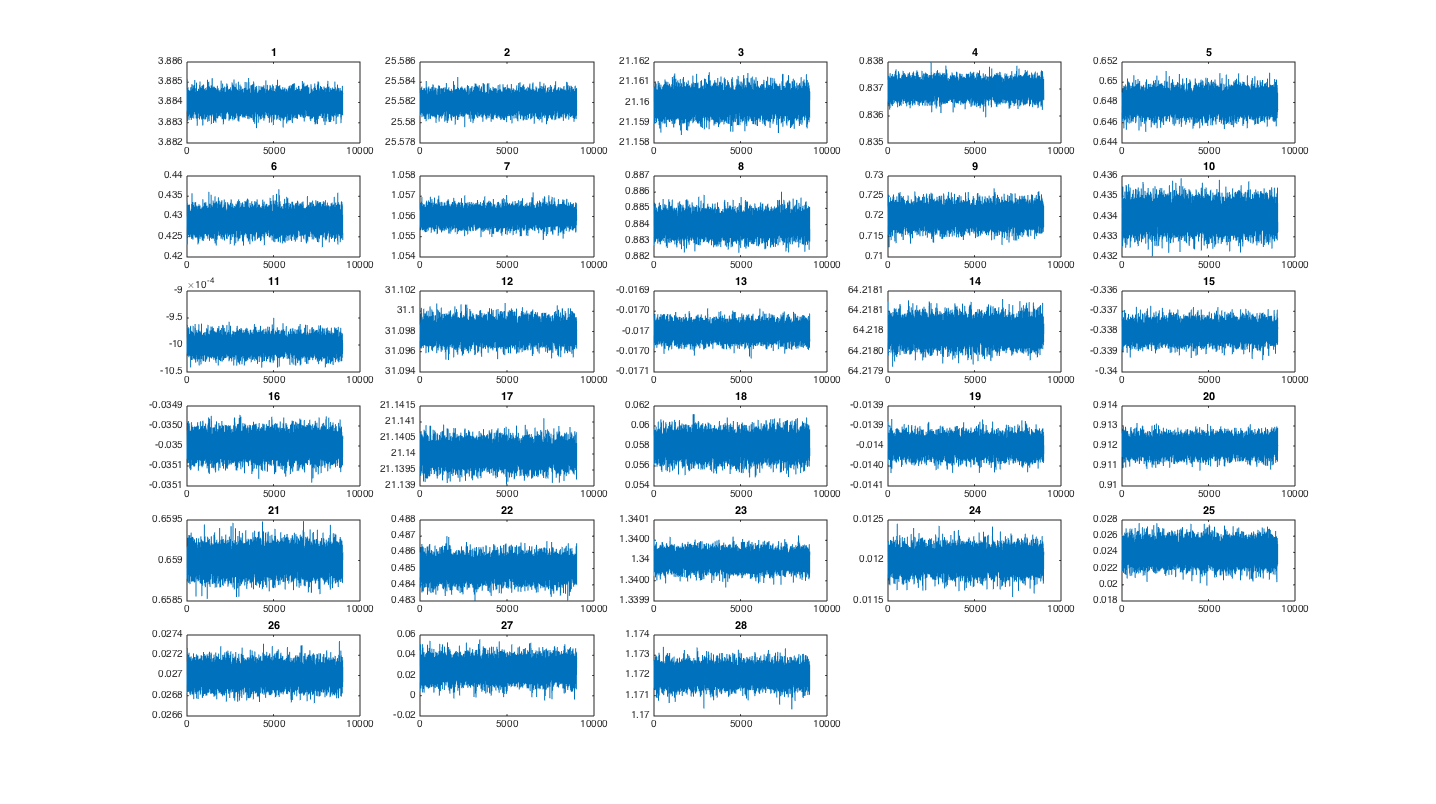


## 15 Figure AF2: Model fit to fertility data

Fertility data are provided in Table AF1. Educational categories are: 0: none; 1: 1 to 6 years; 2: >6 to 12 years; 3: >12 years. In all plots, gray shaded areas are results of 10,000 repeated samples from the posterior joint distribution of the fitted model (Figure 1C), with samples from the interquartile range as black lines and data displayed as dashed blue lines or circles reflecting the 95% confidence intervals of the input datasets.


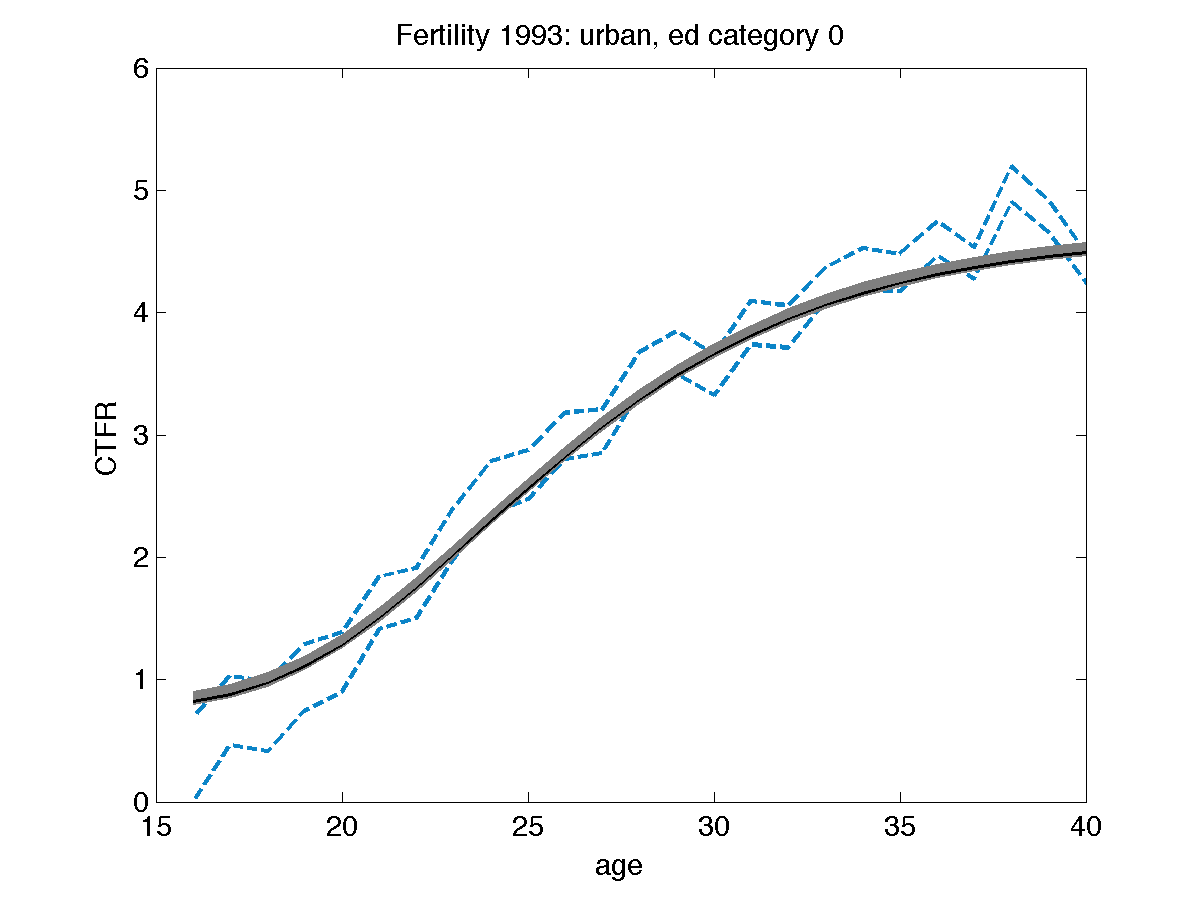


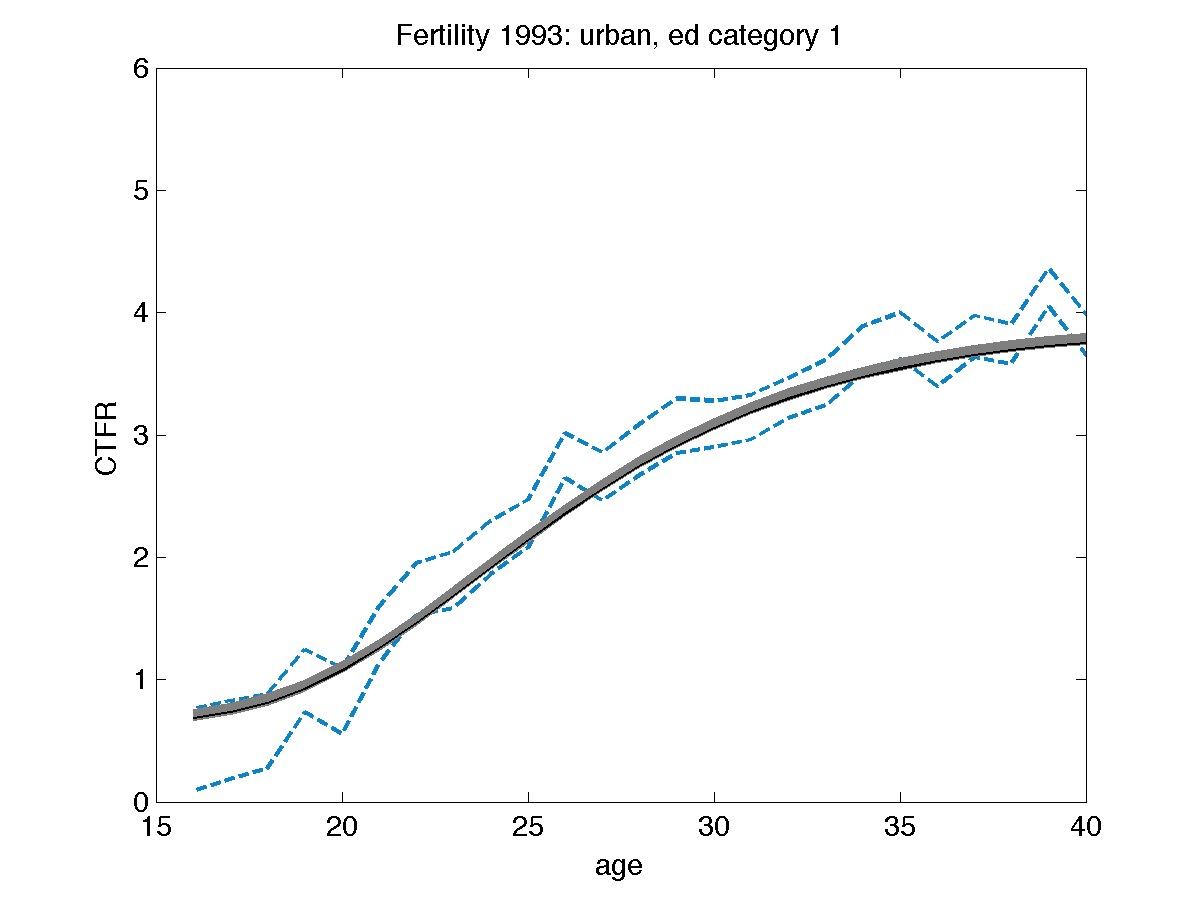


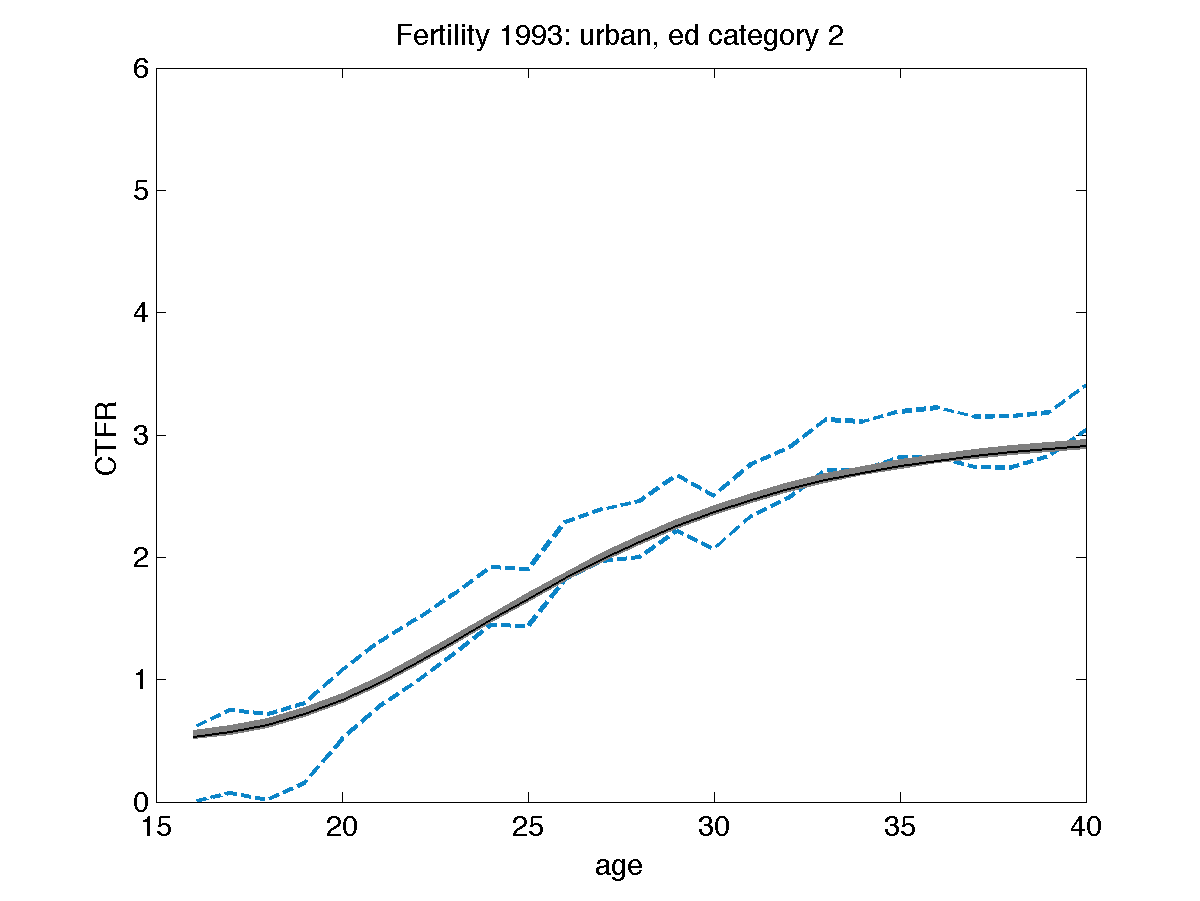


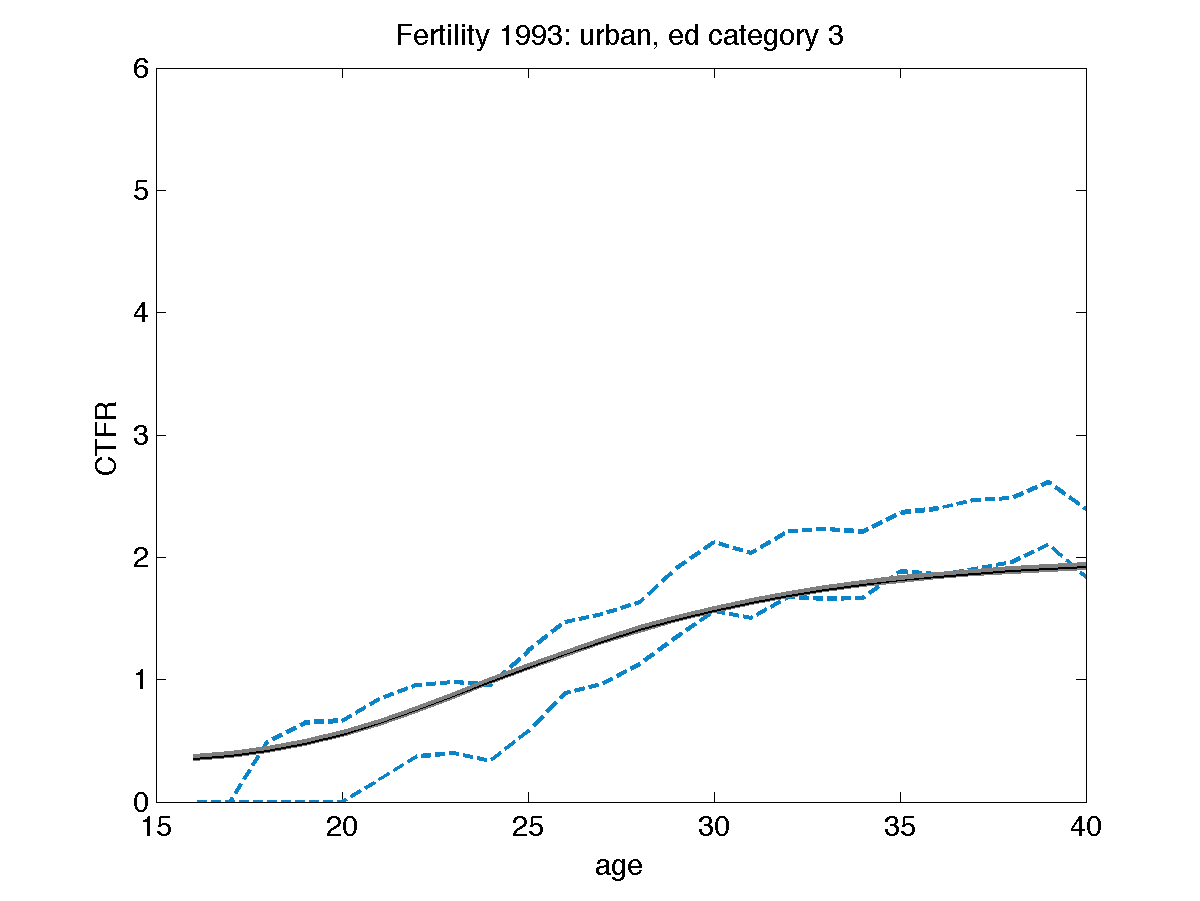


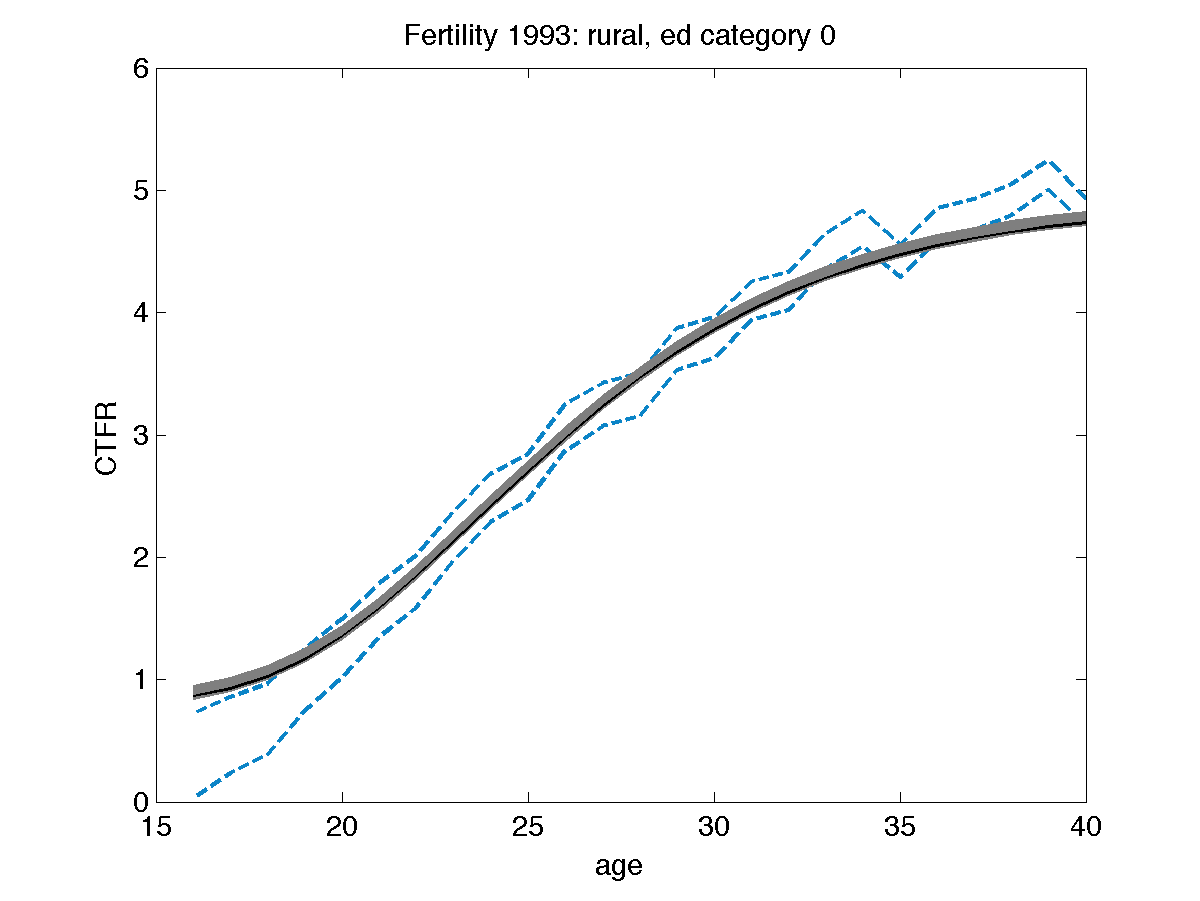


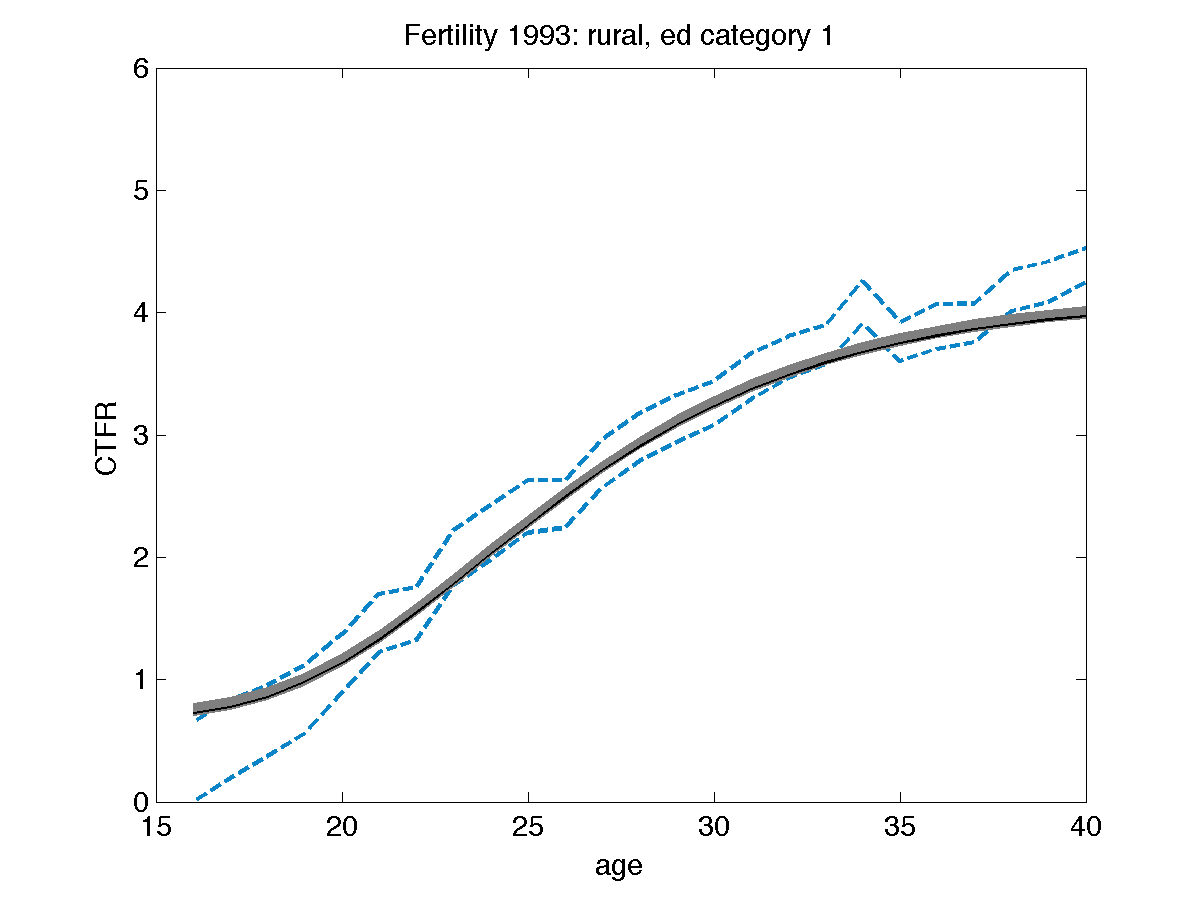


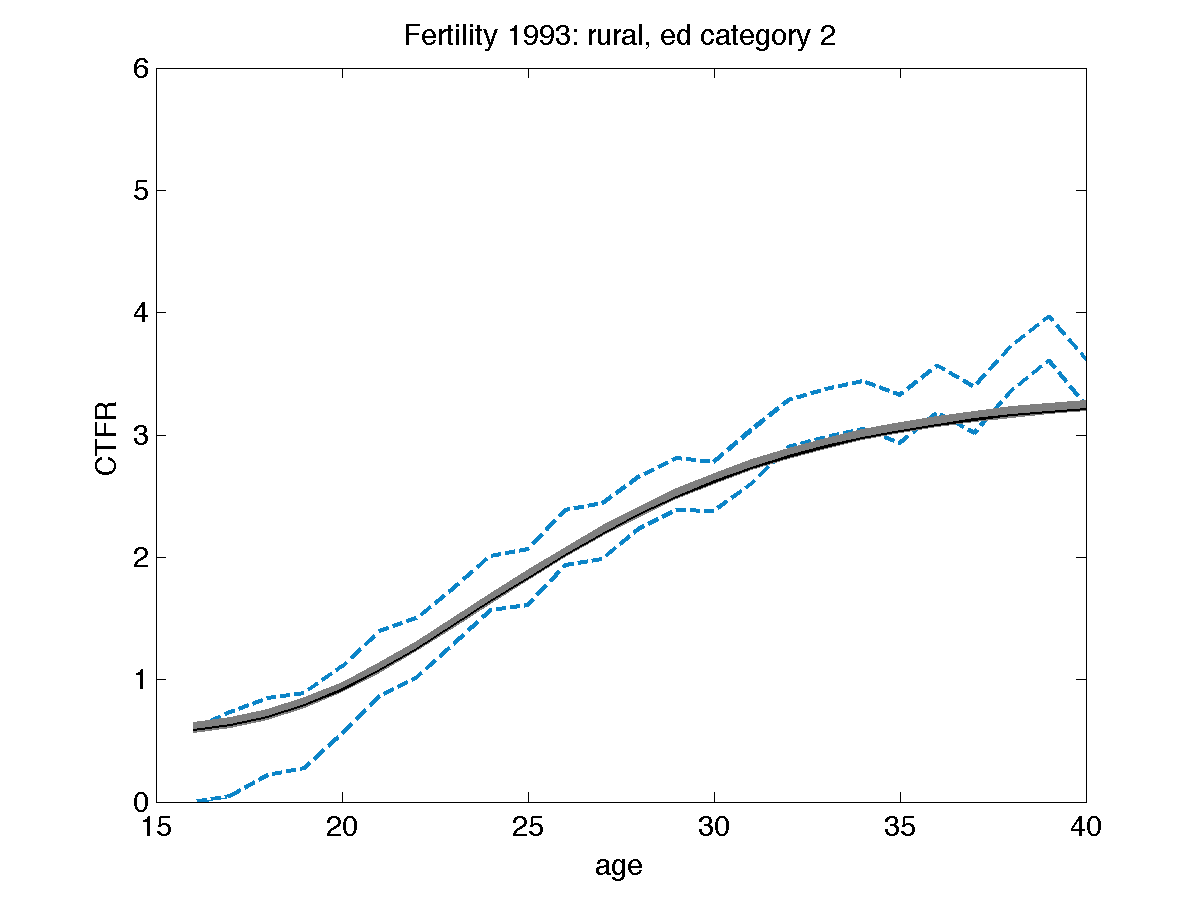


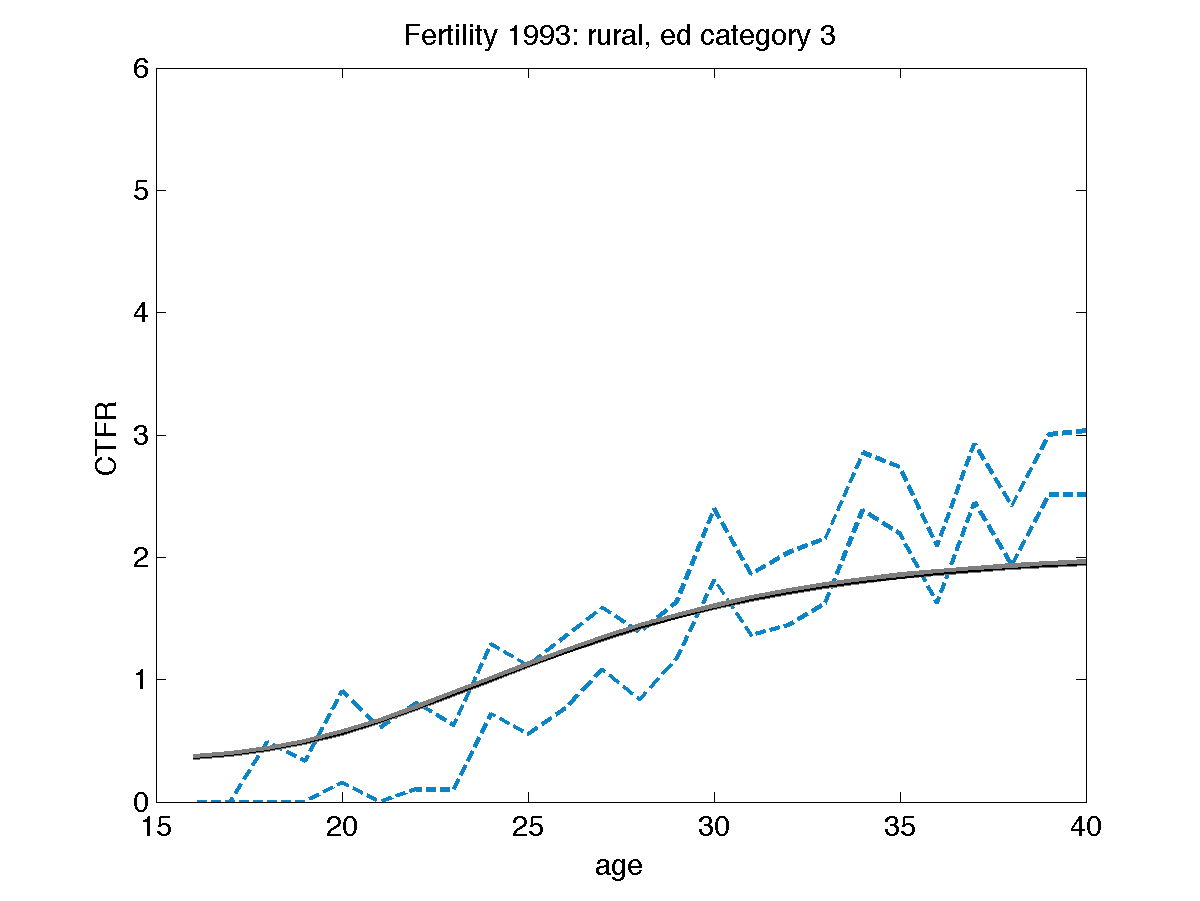


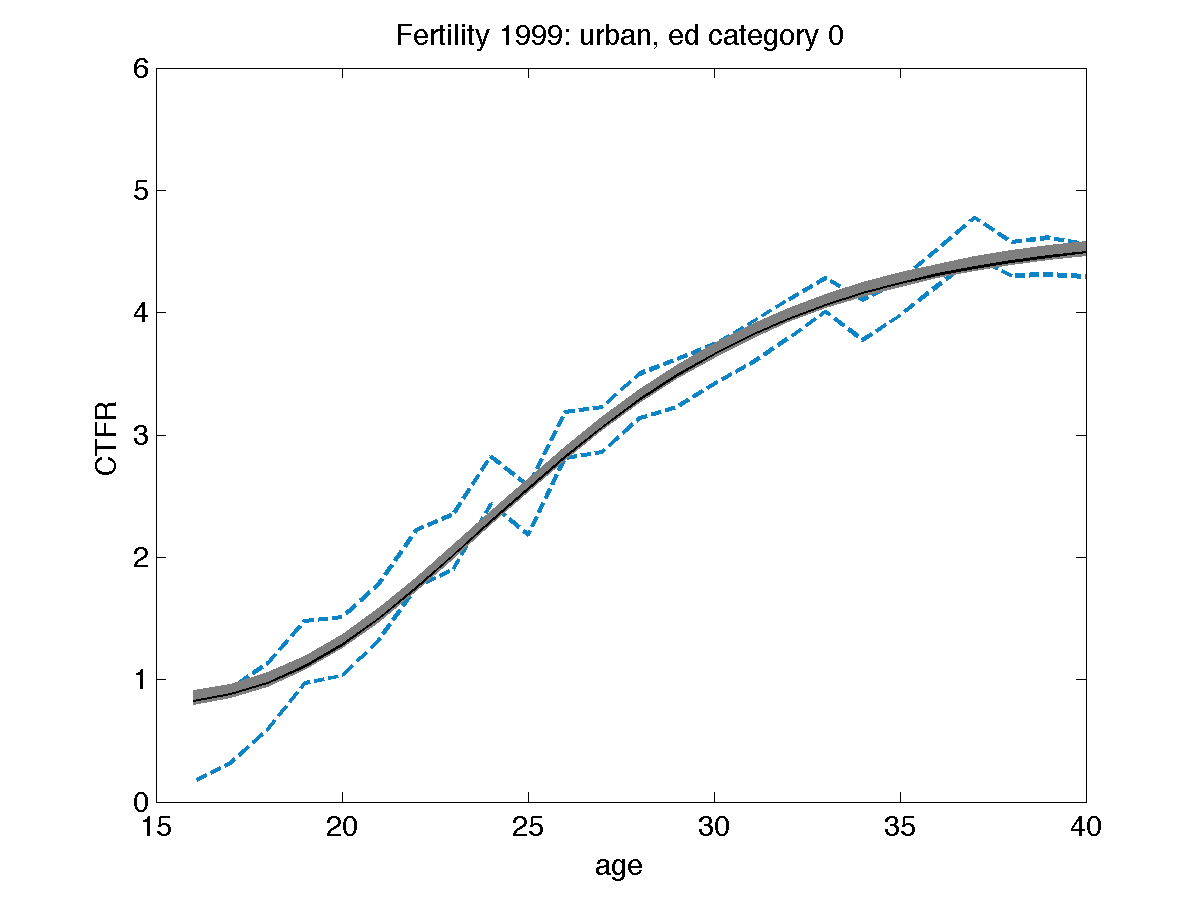


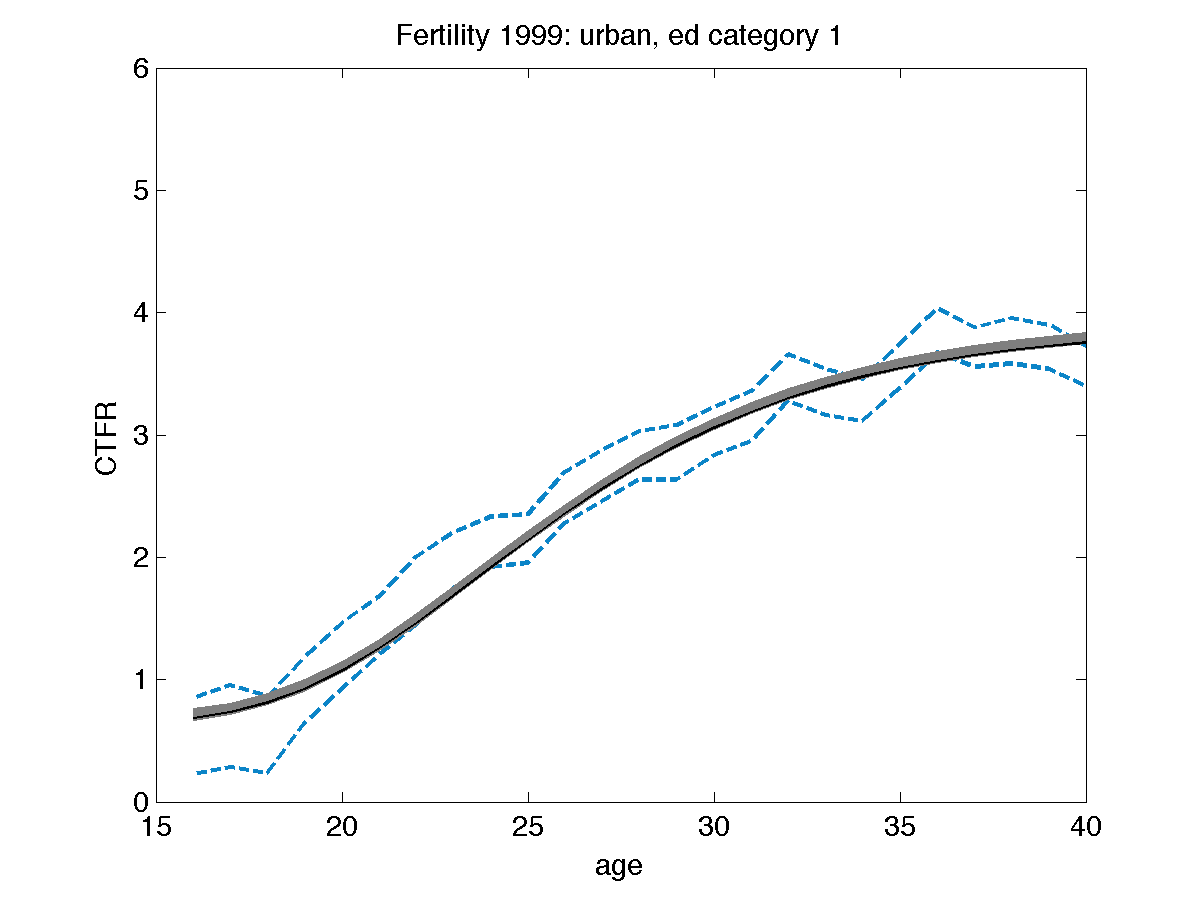


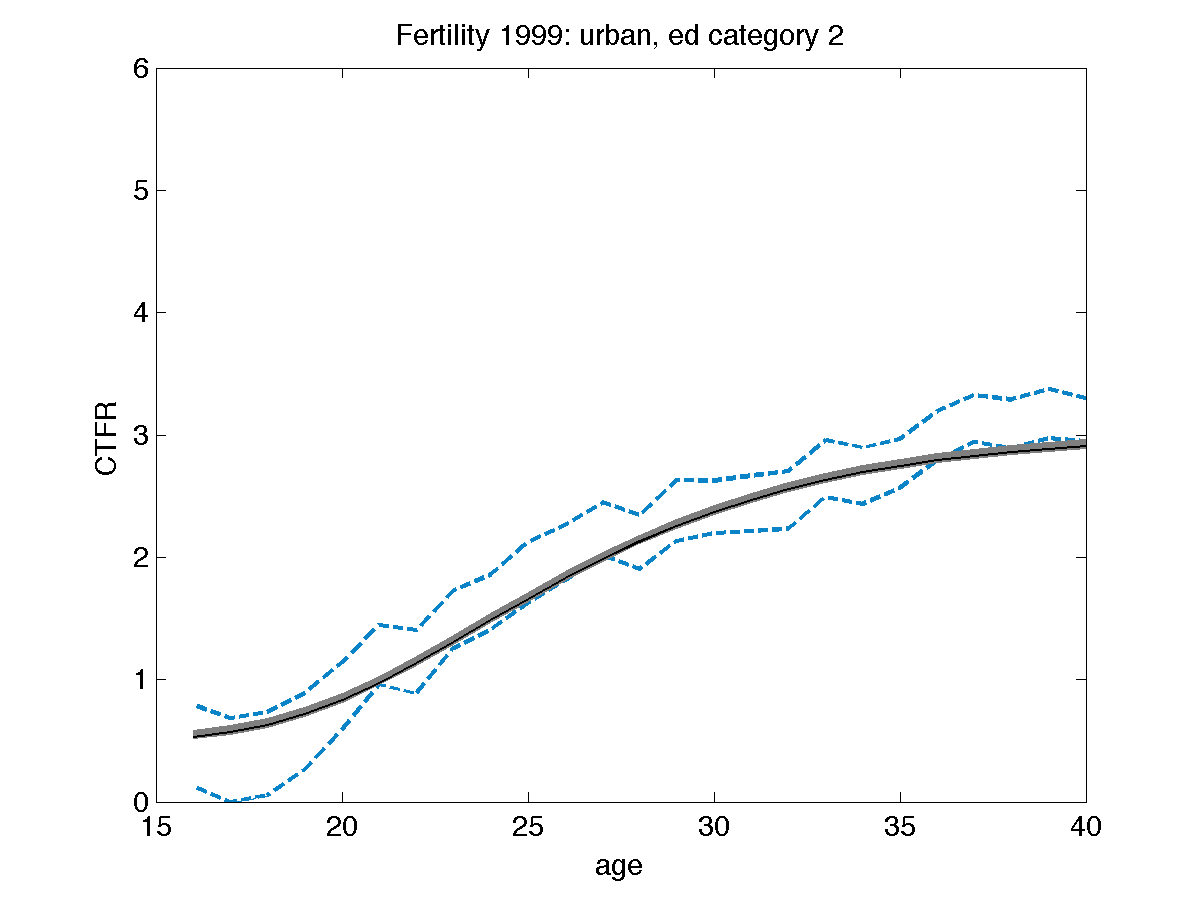


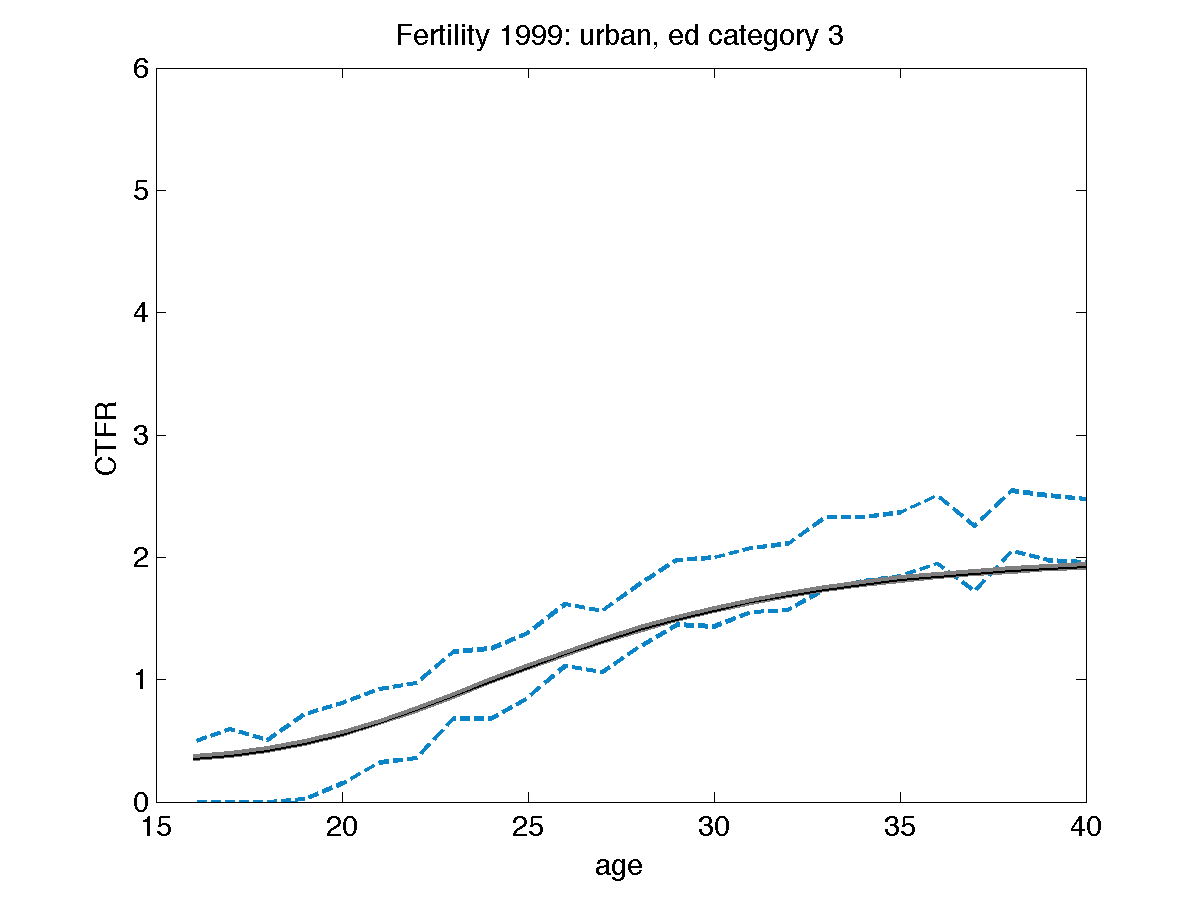


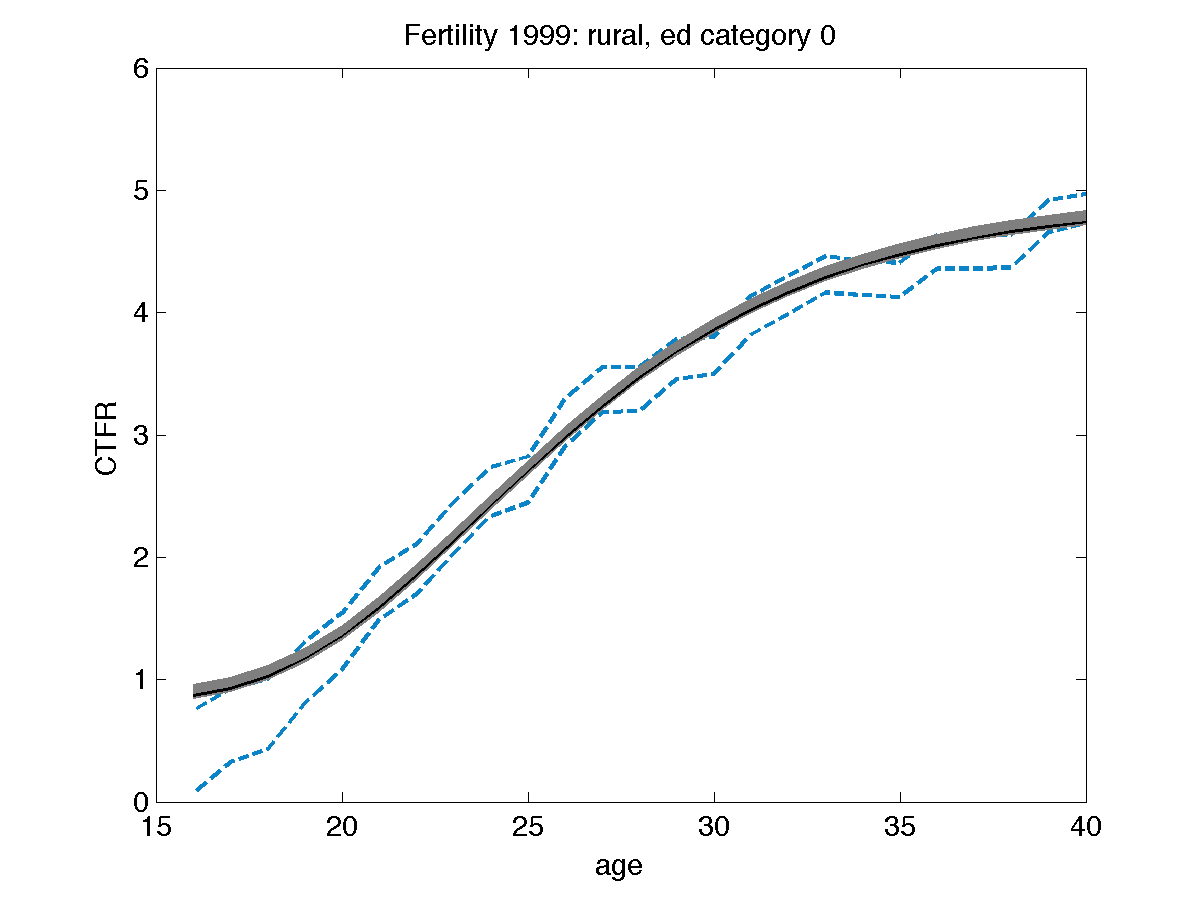


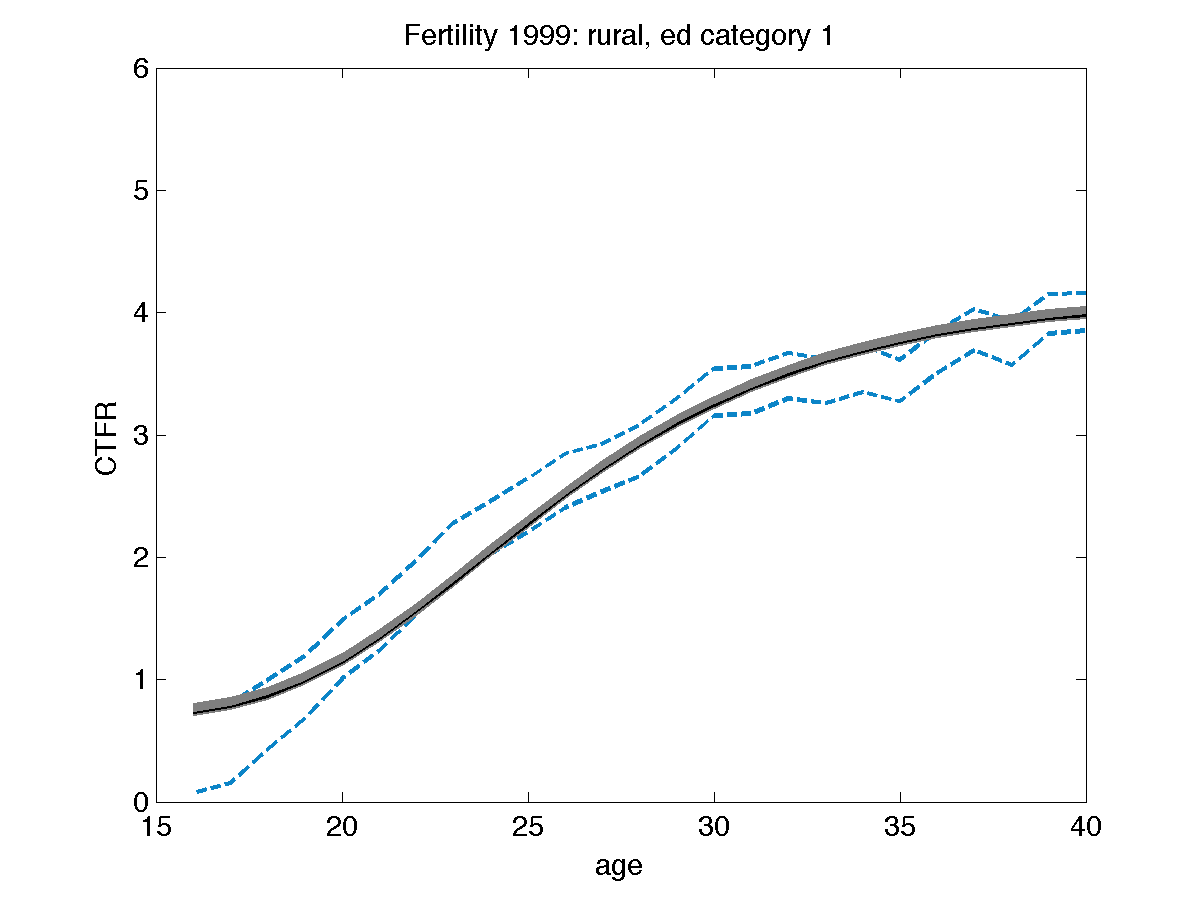


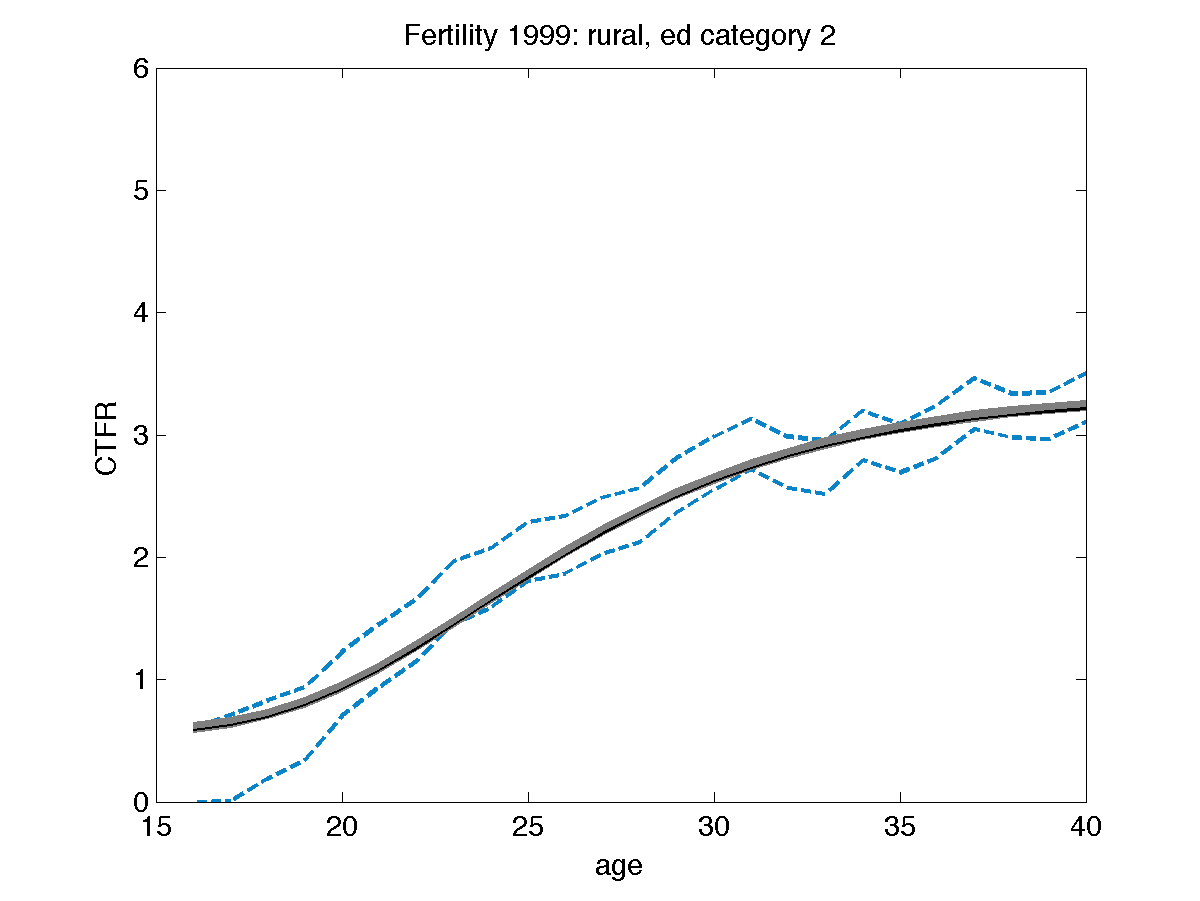


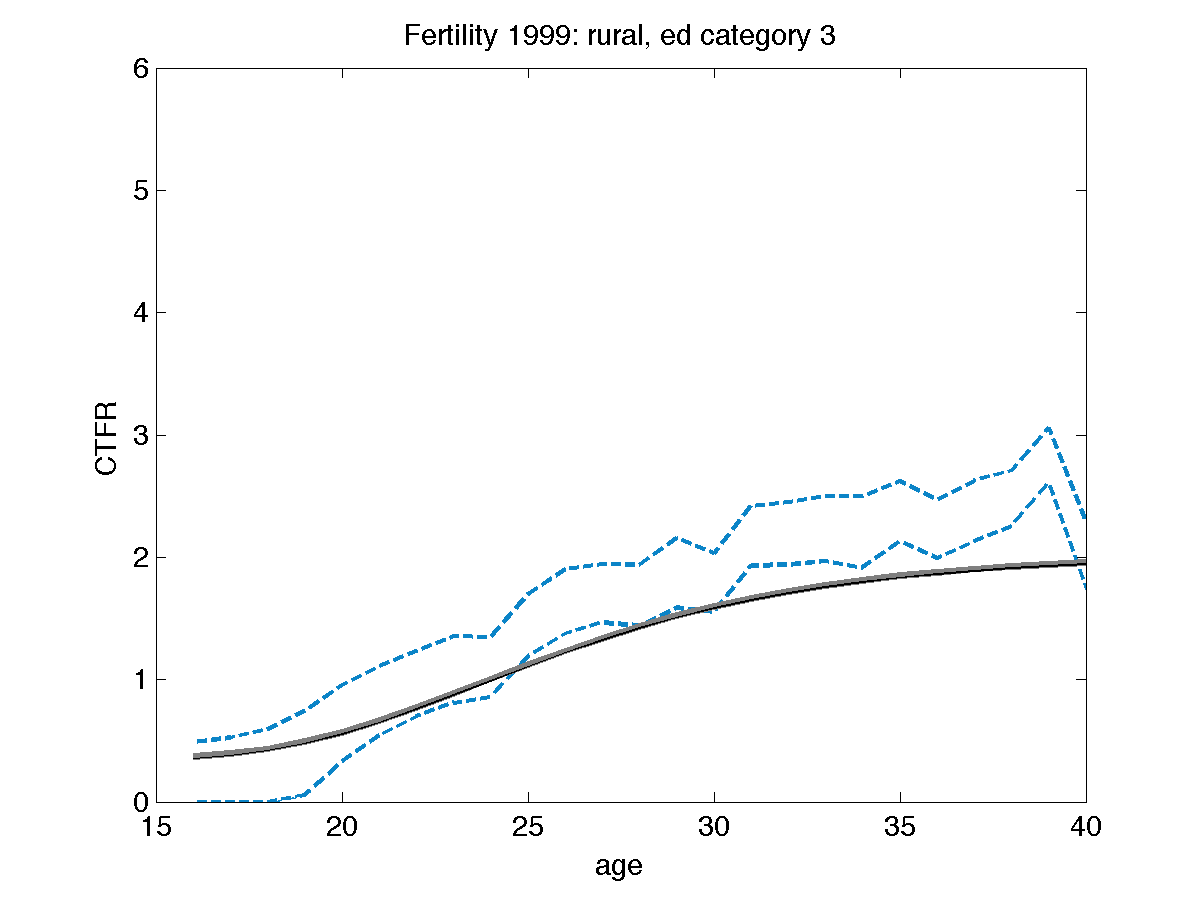


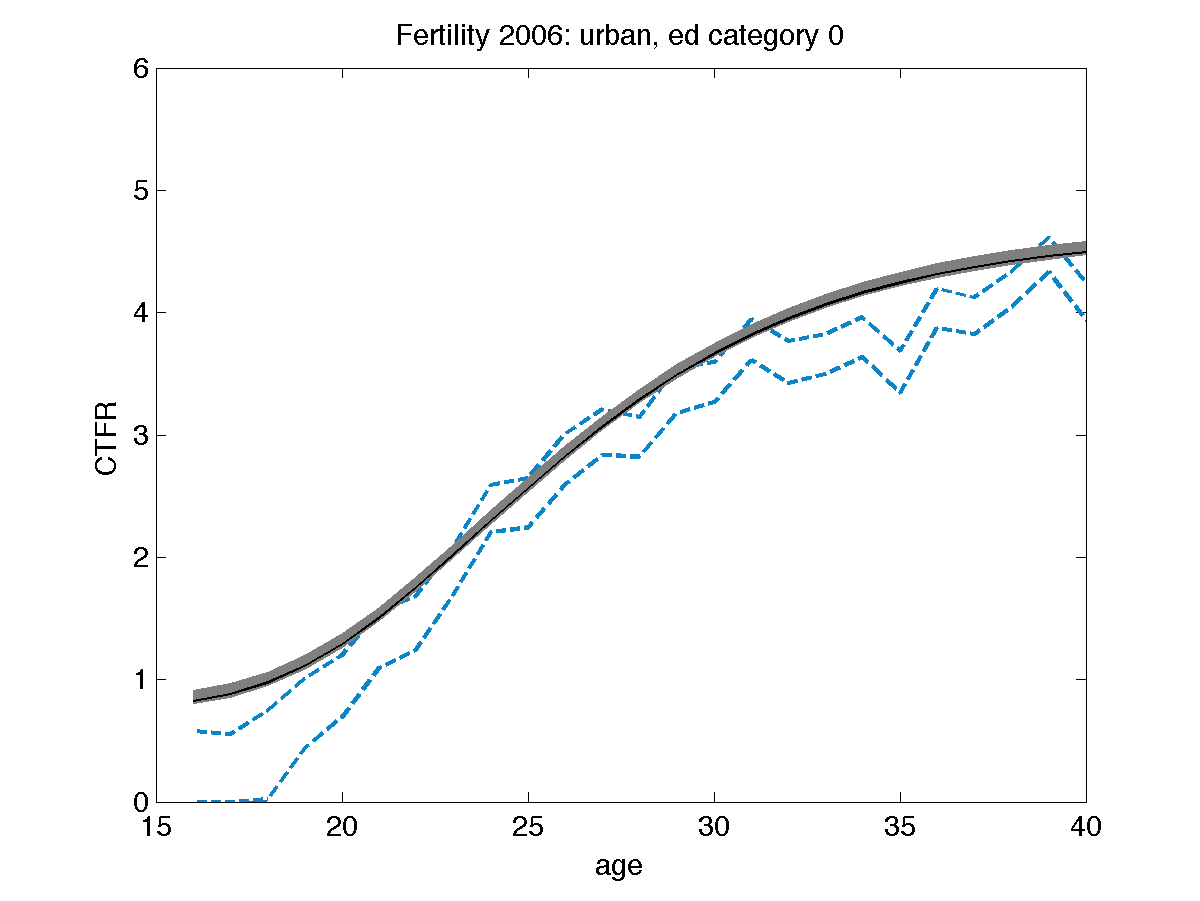


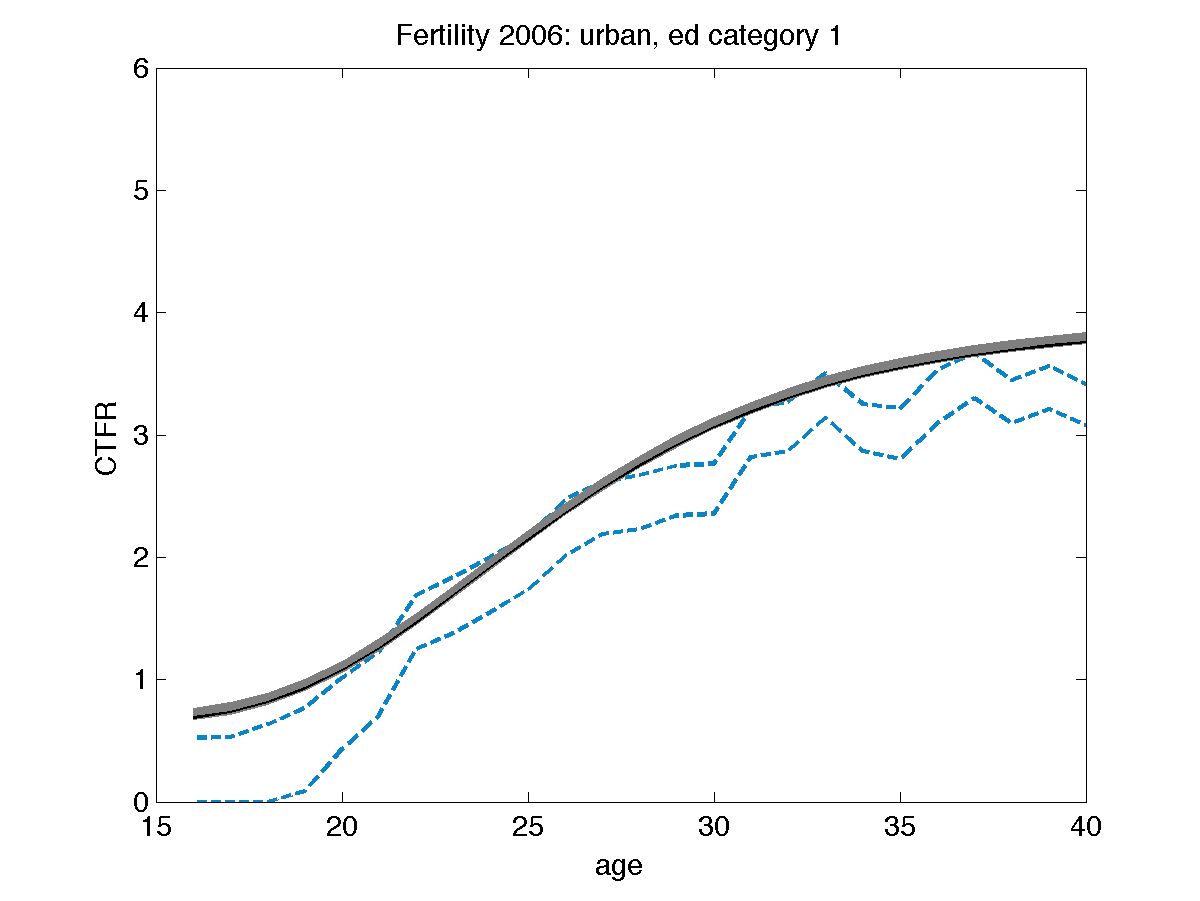


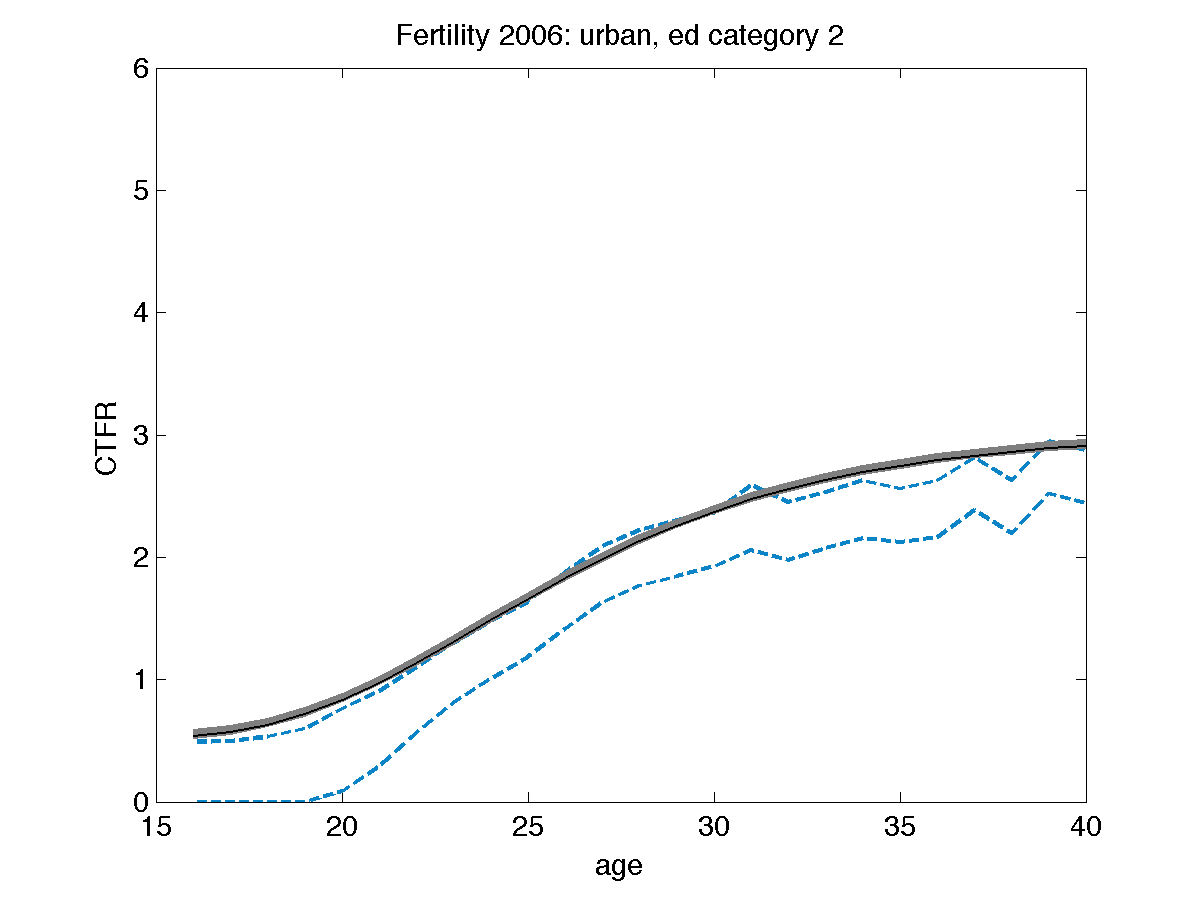


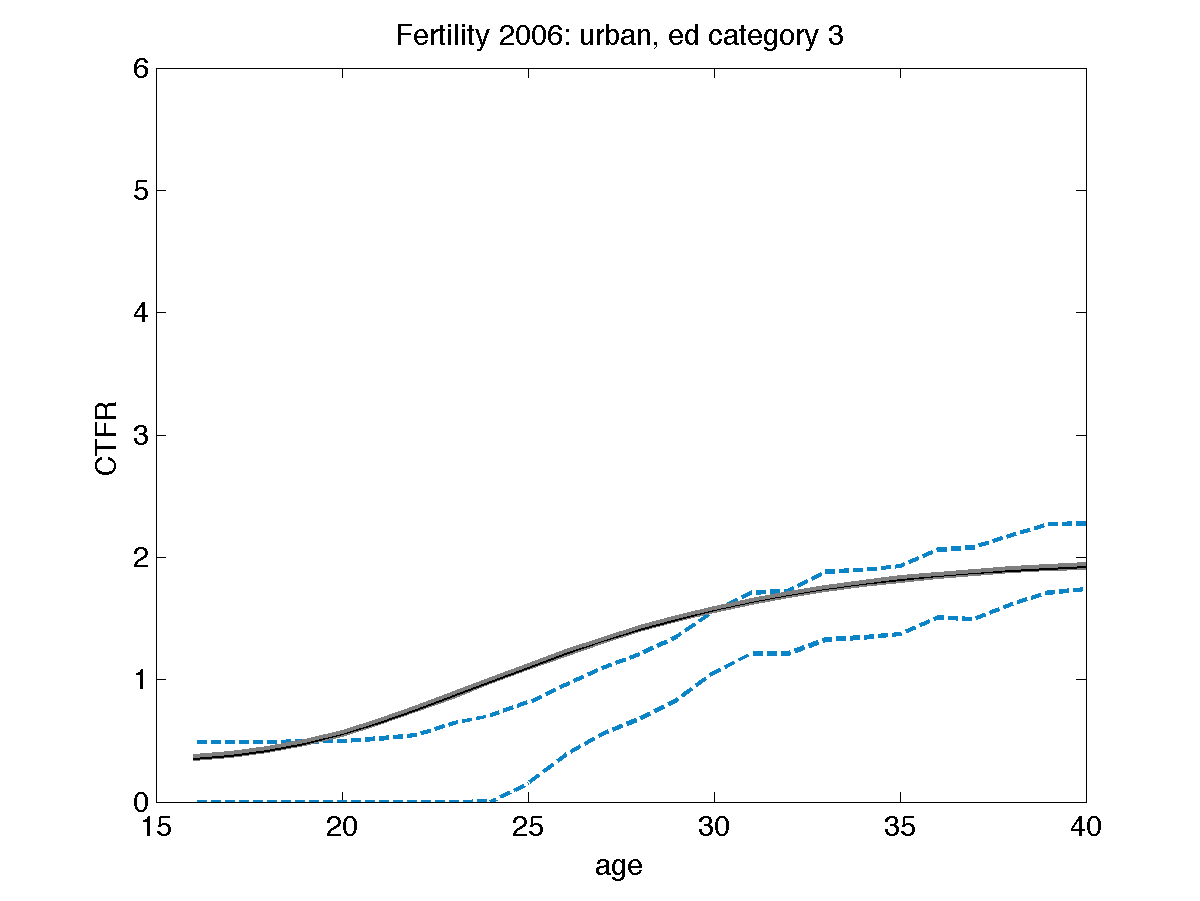


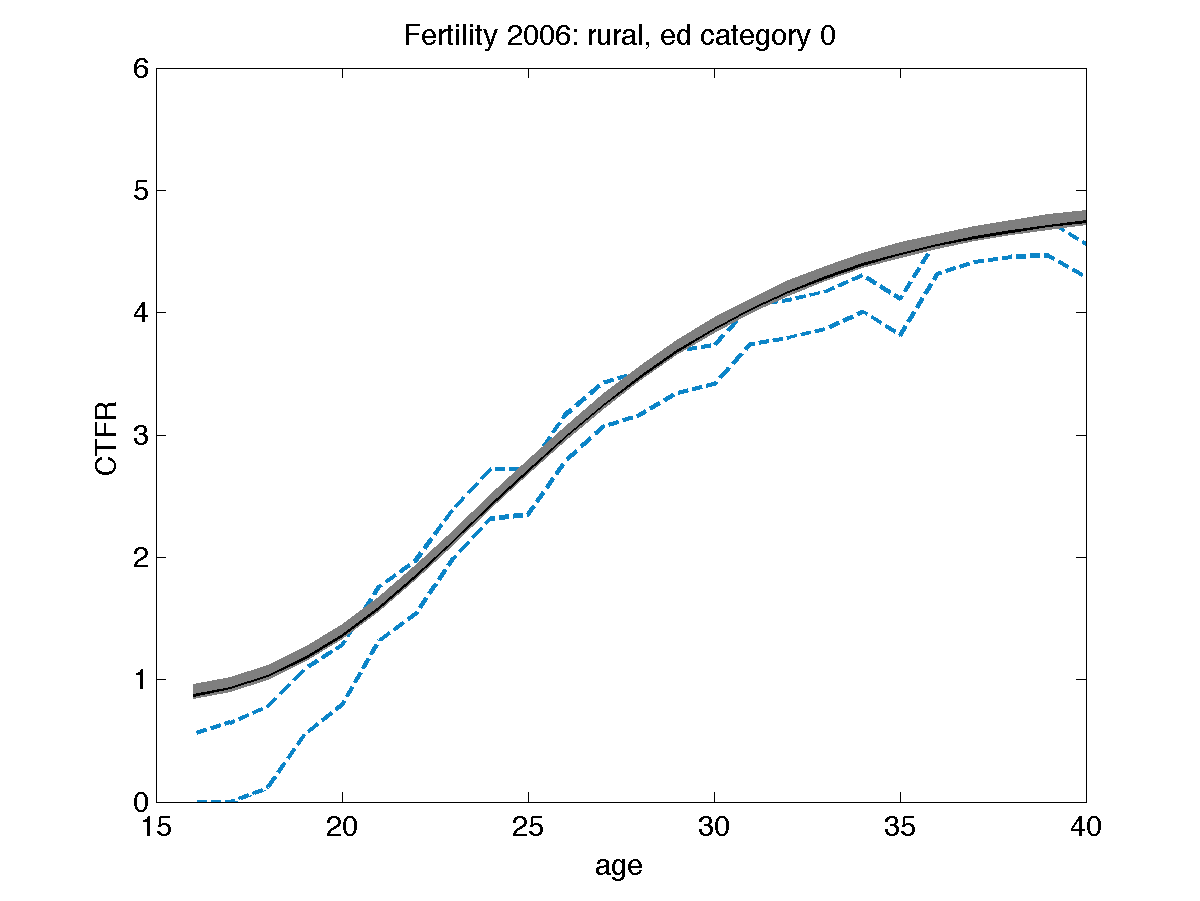


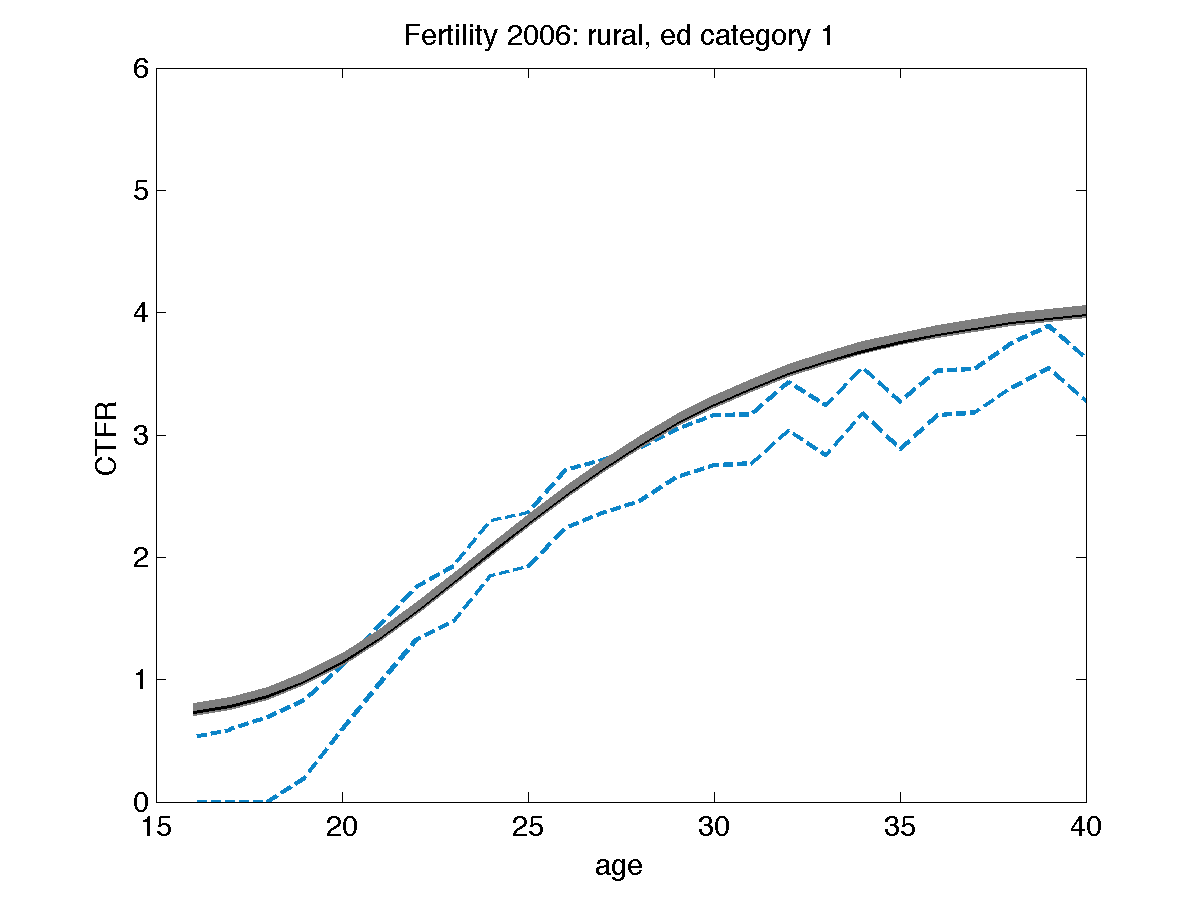


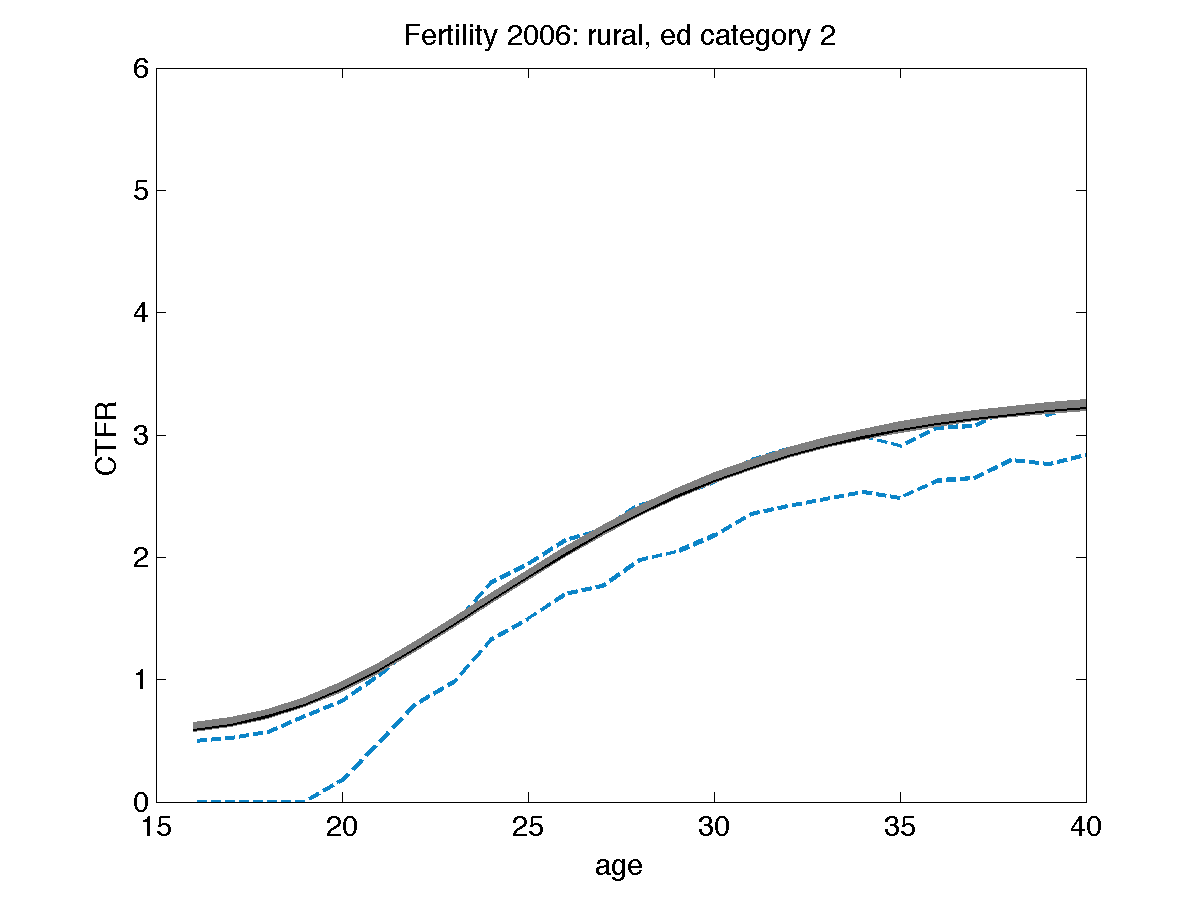


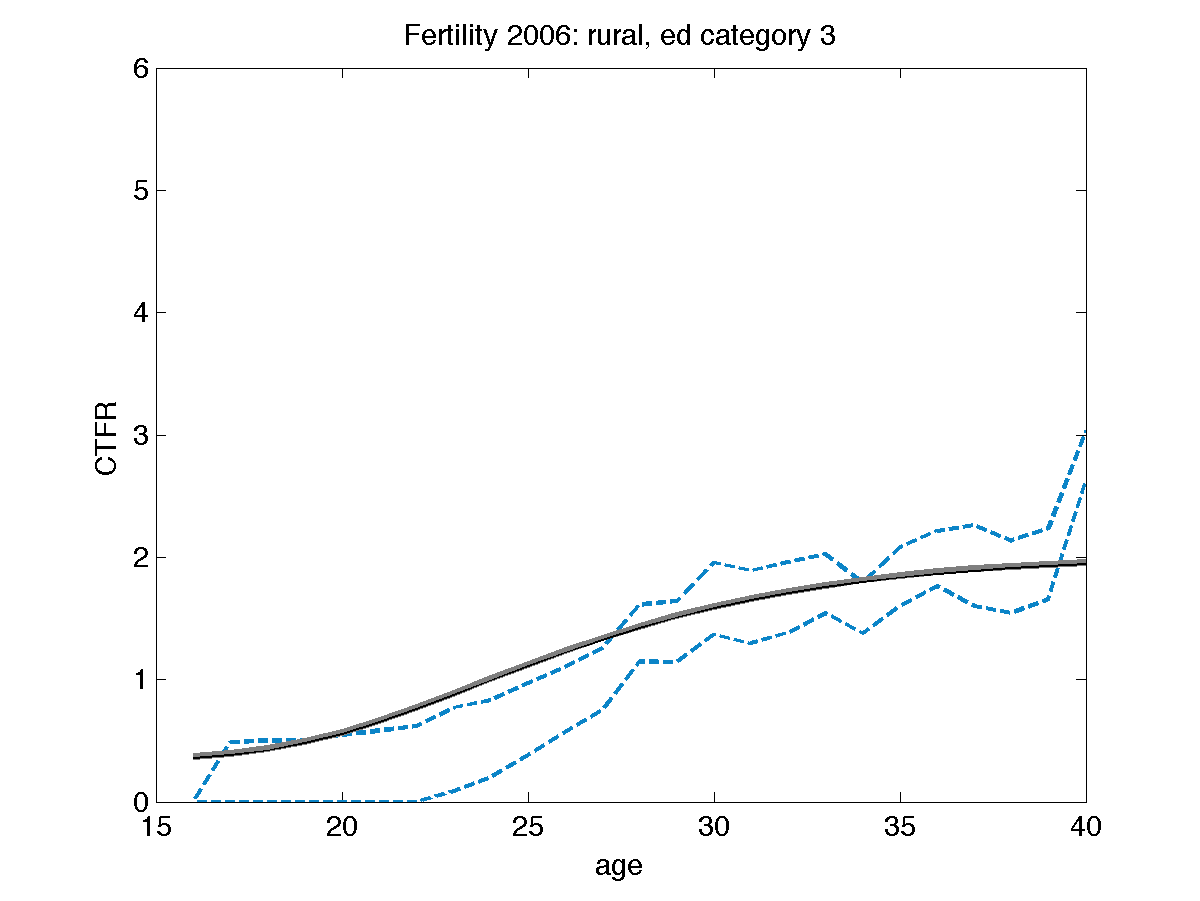


## 16 Figure AF3: Model fits to mortality data

Mortality data are provided in Table AF2. Educational categories are: 0: none; 1: 1 to 6 years; 2: >6 to 12 years; 3: >12 years. In all plots, gray shaded areas are results of 10,000 repeated samples from the posterior joint distribution of the fitted model (Figure 1C), with samples from the interquartile range as black lines and data displayed as dashed blue lines or circles reflecting the 95% confidence intervals of the input datasets.


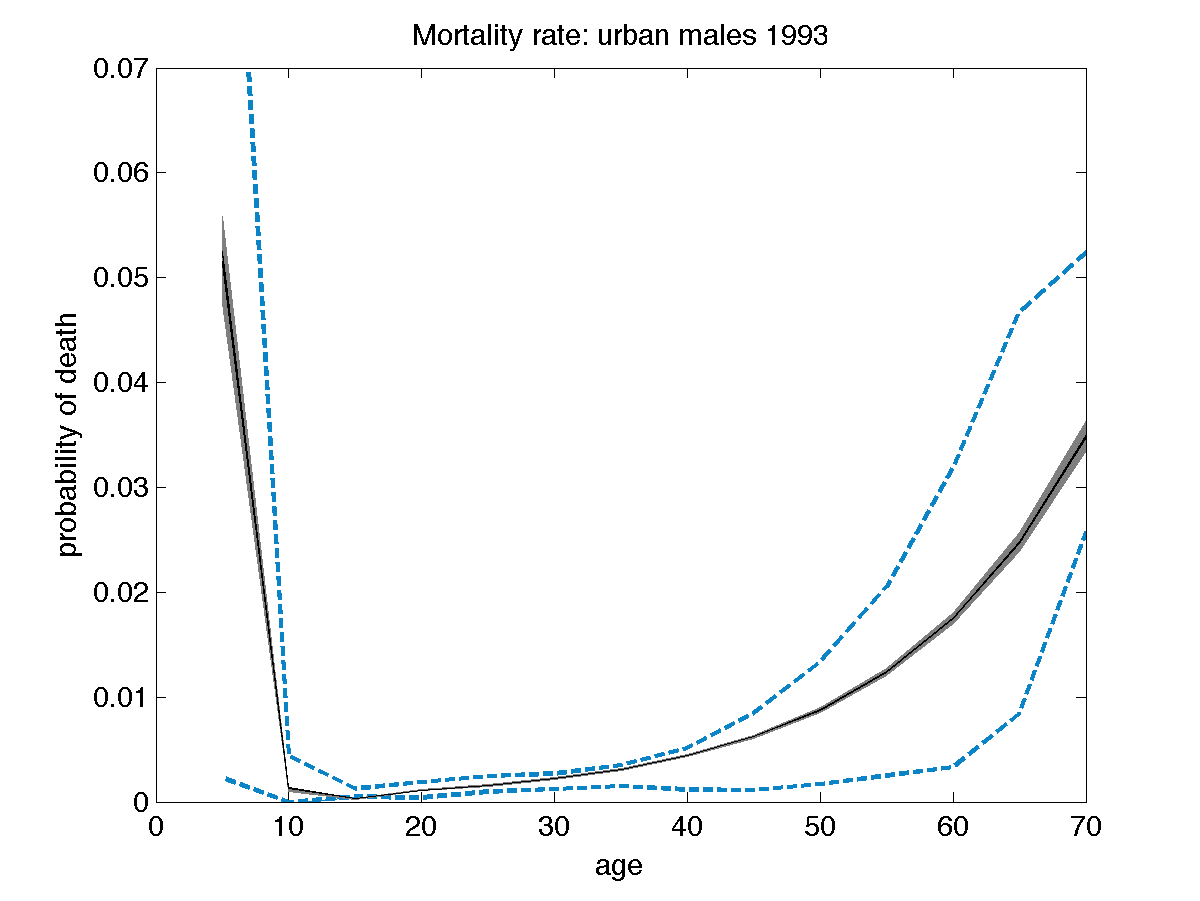


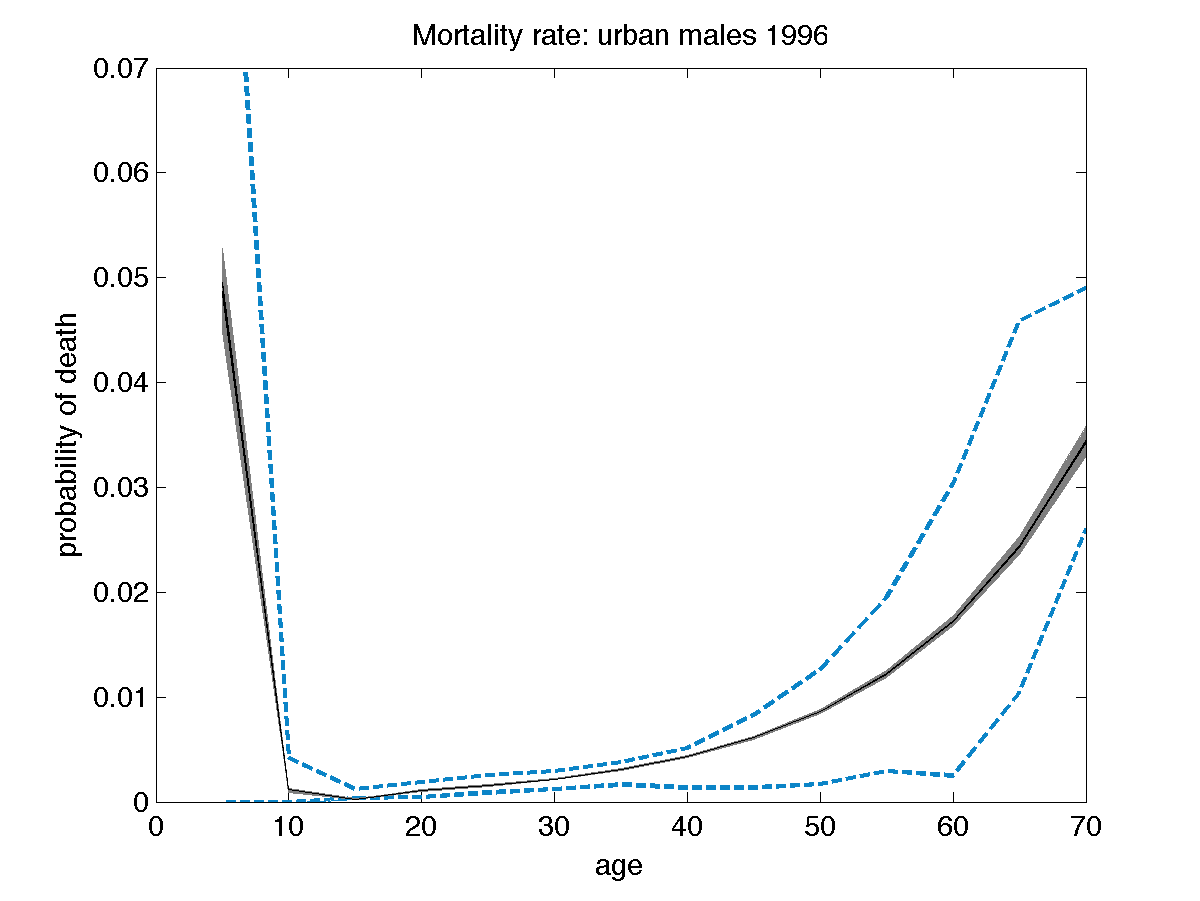


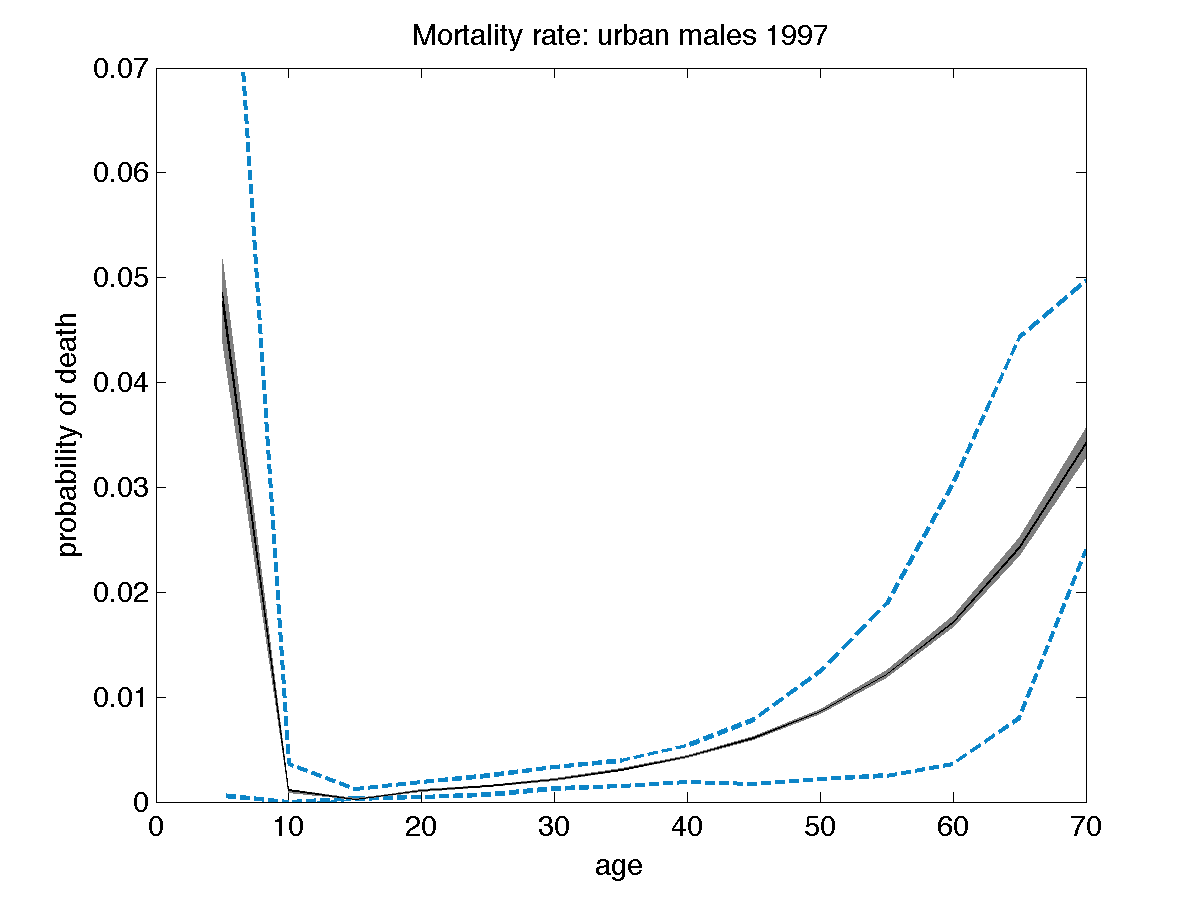


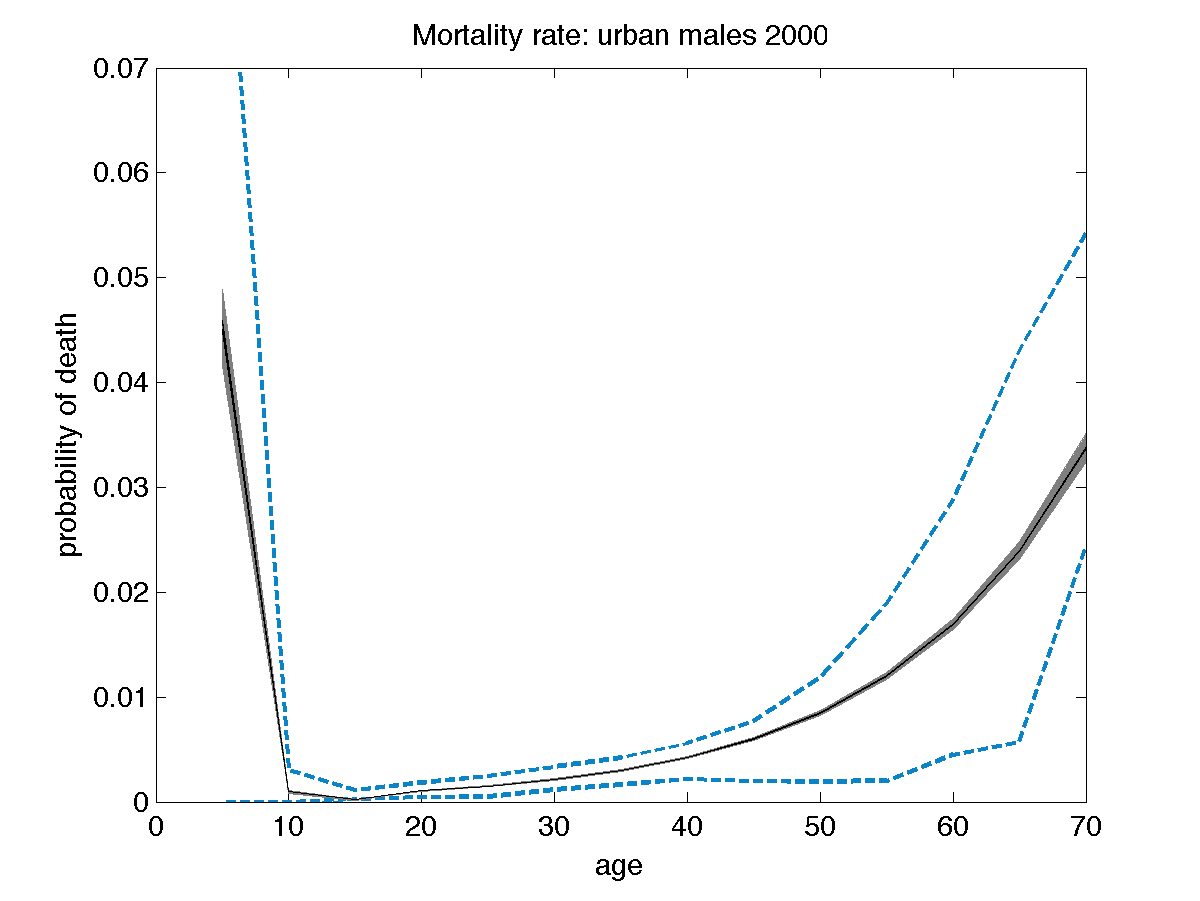


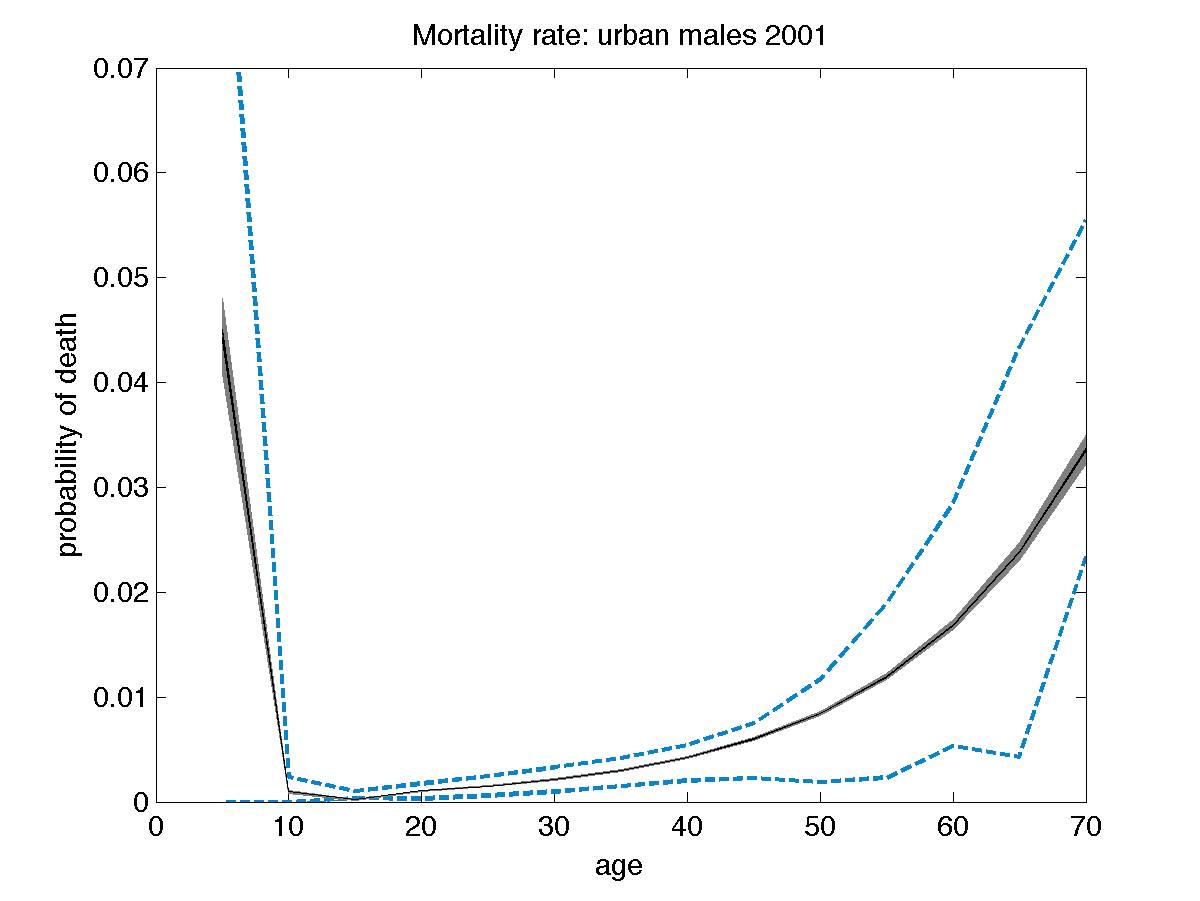


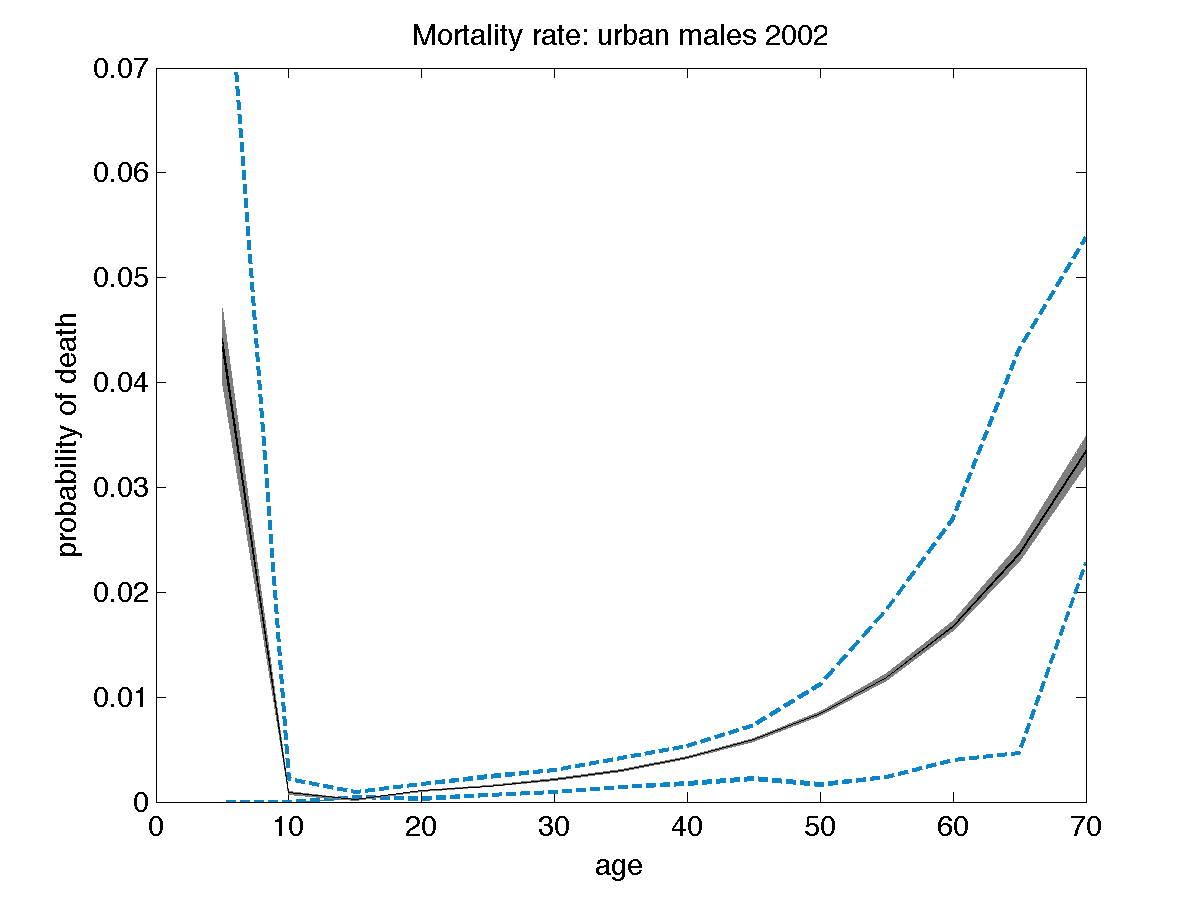


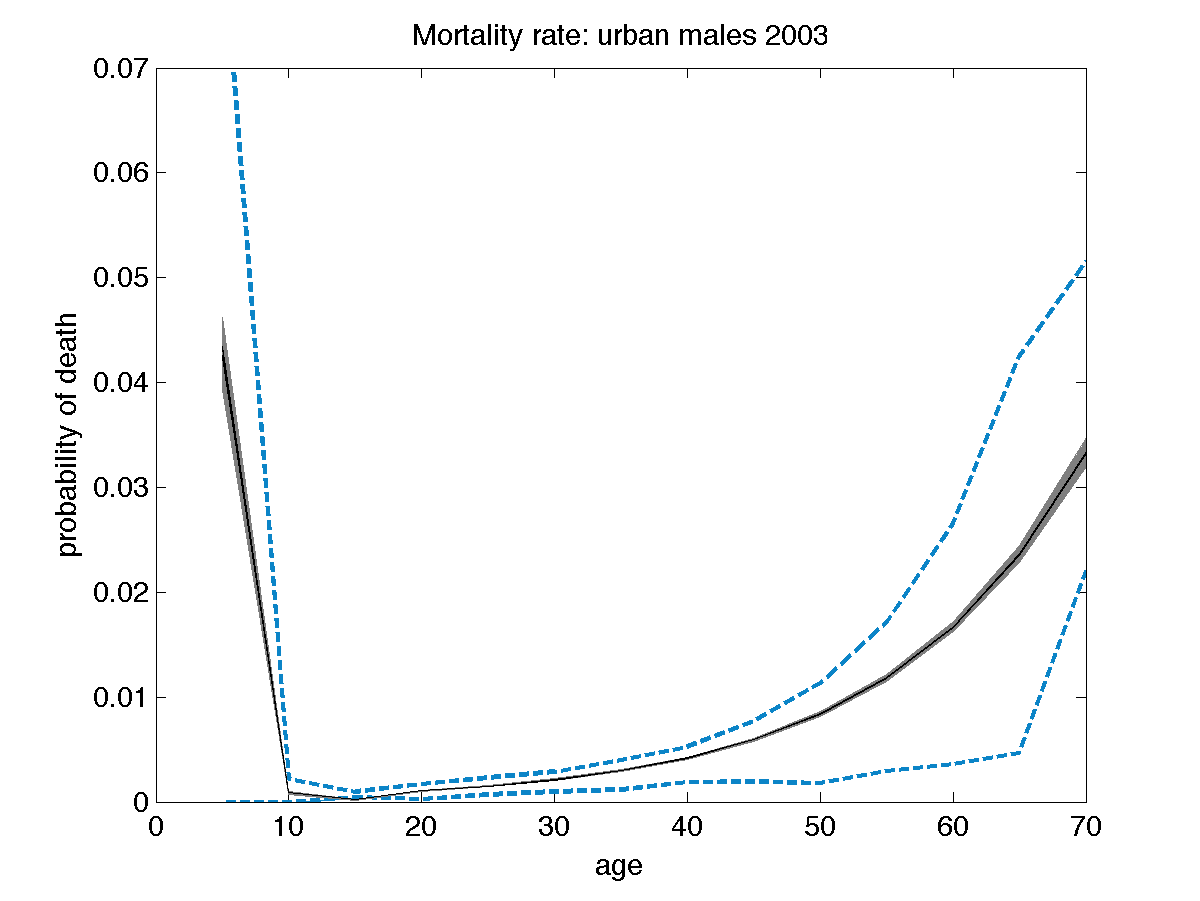


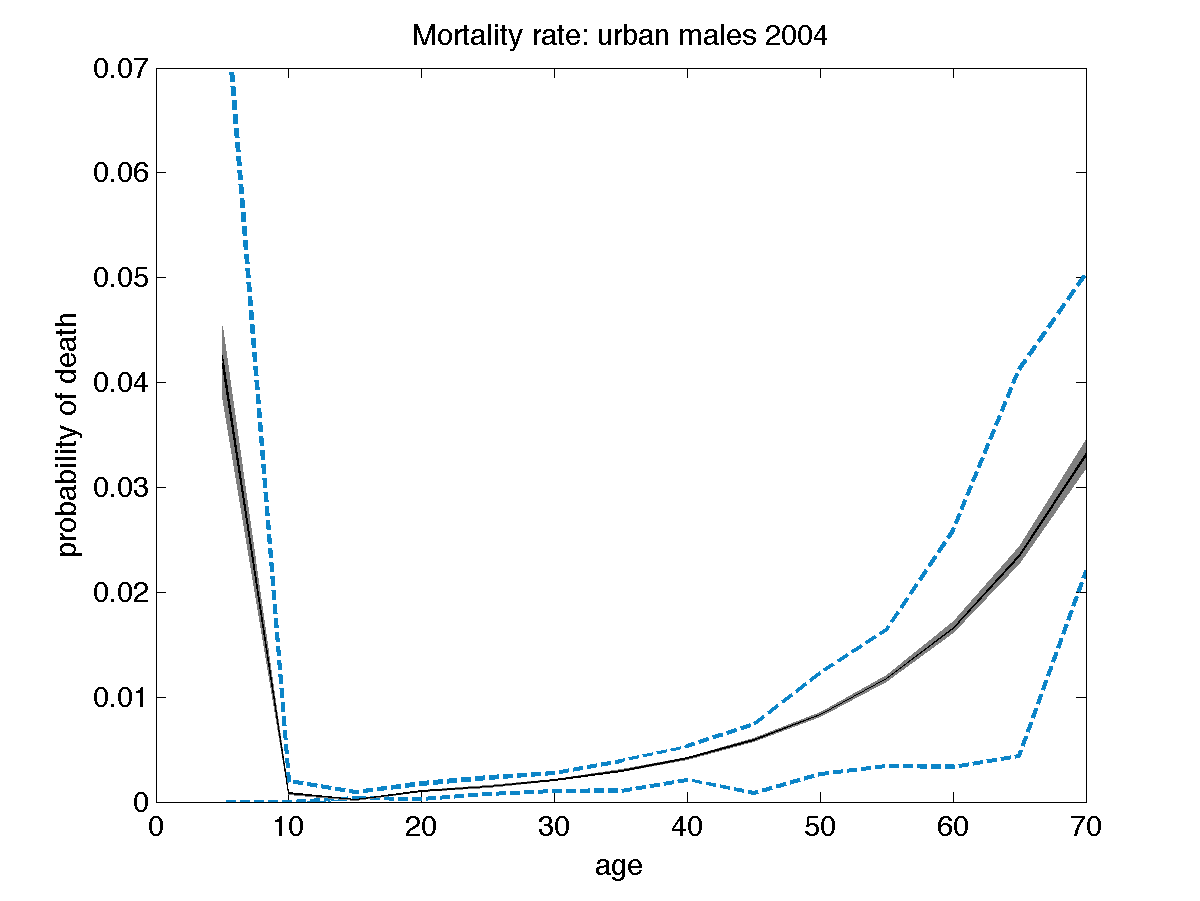


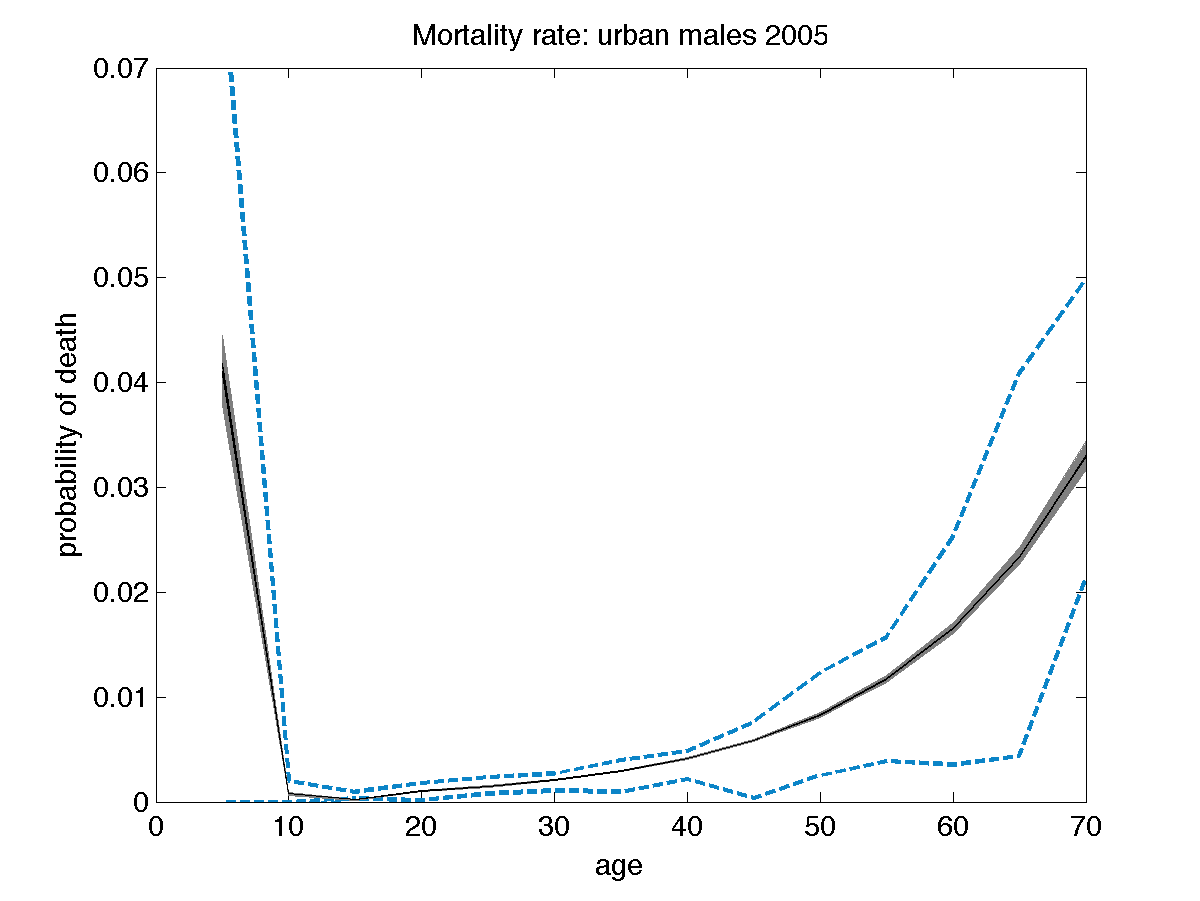


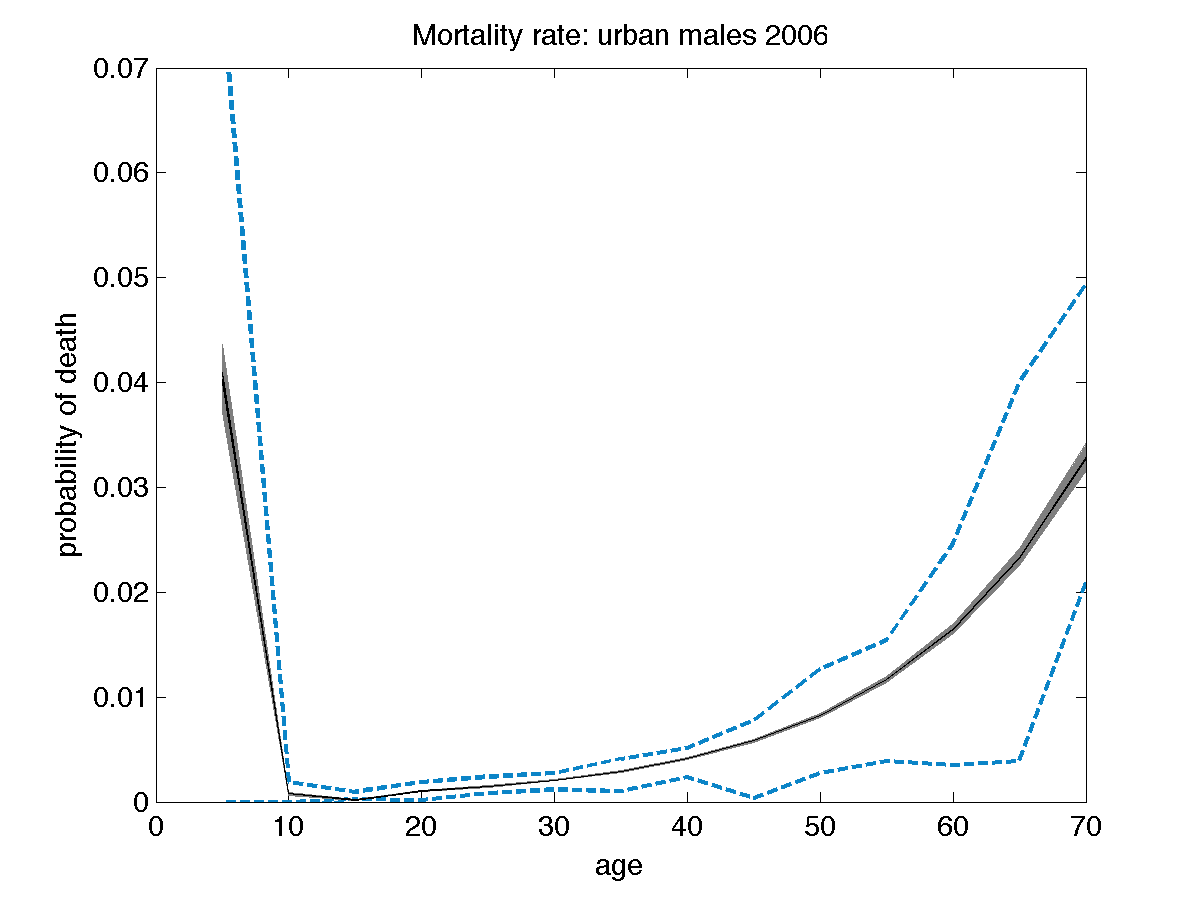


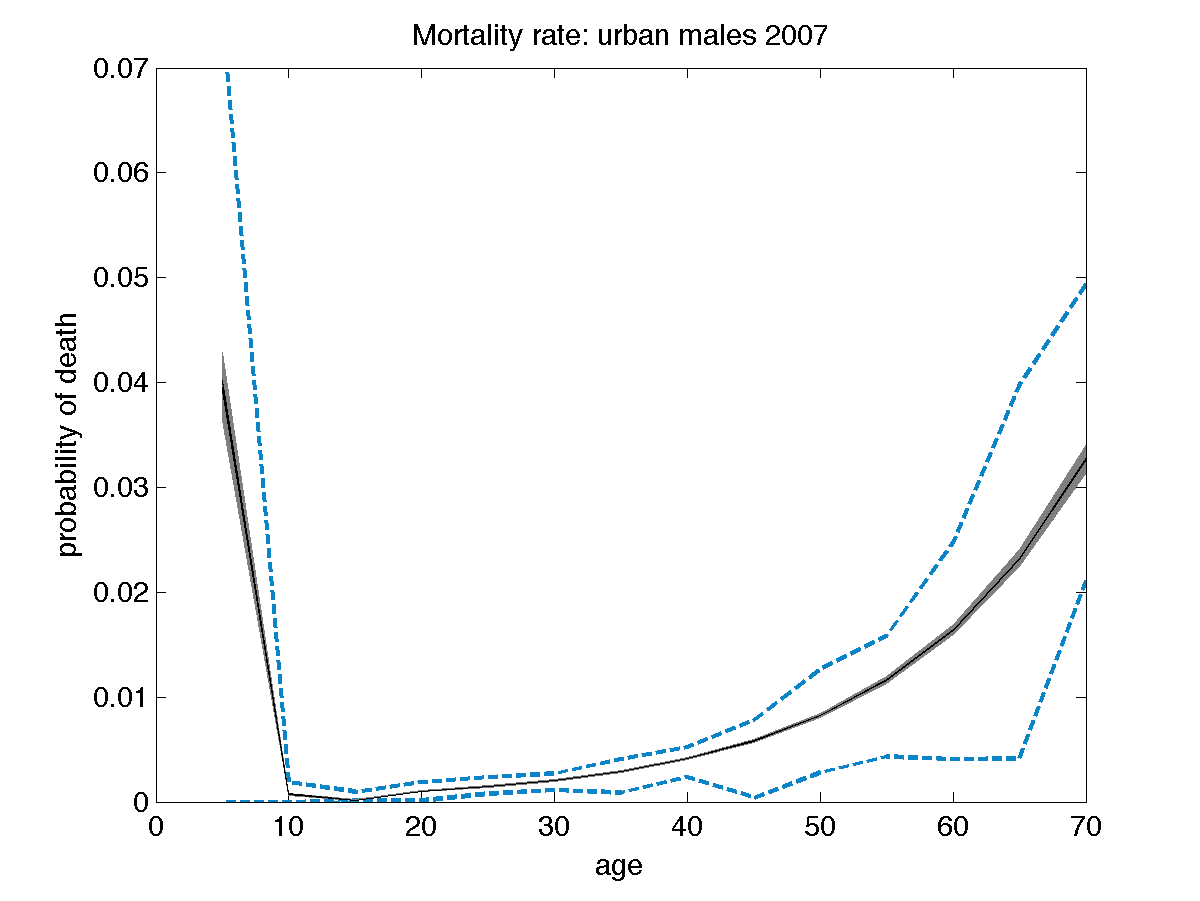


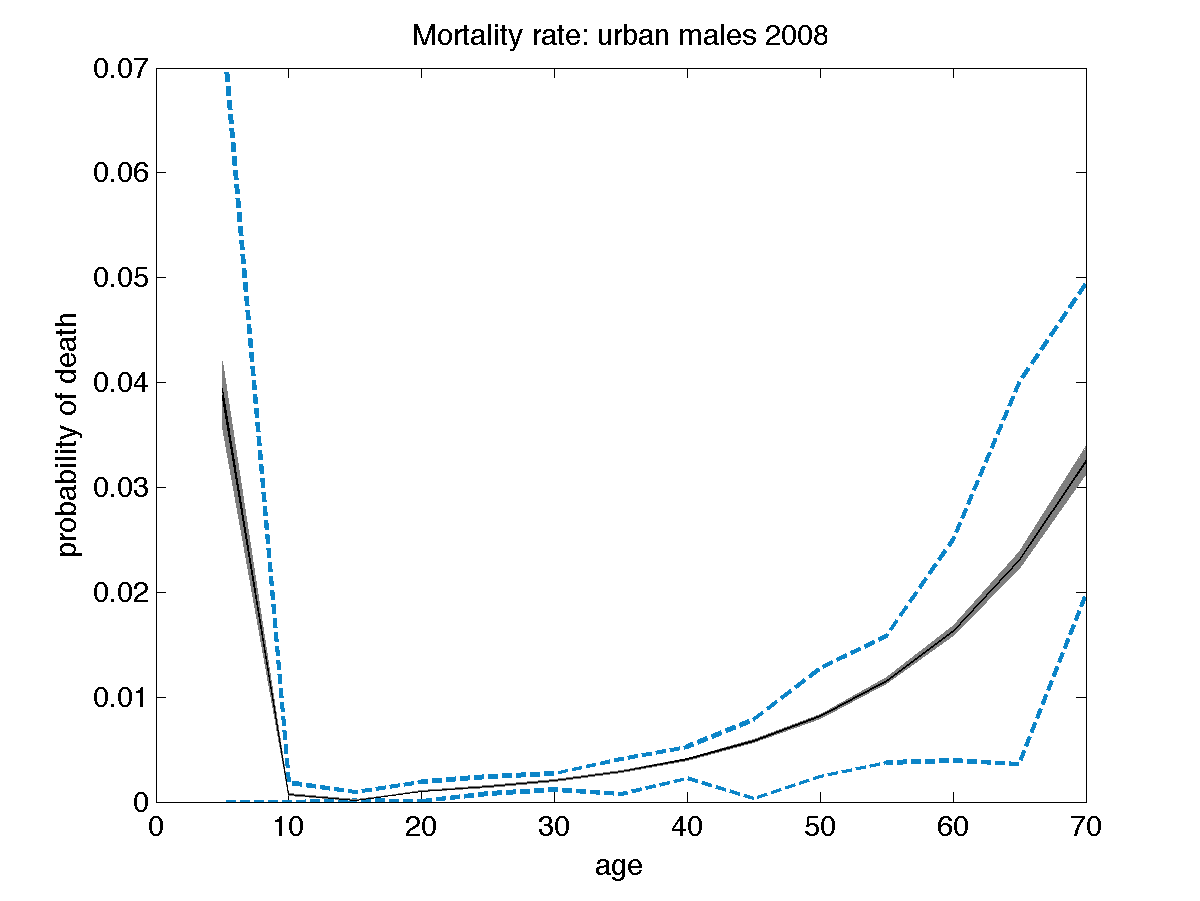


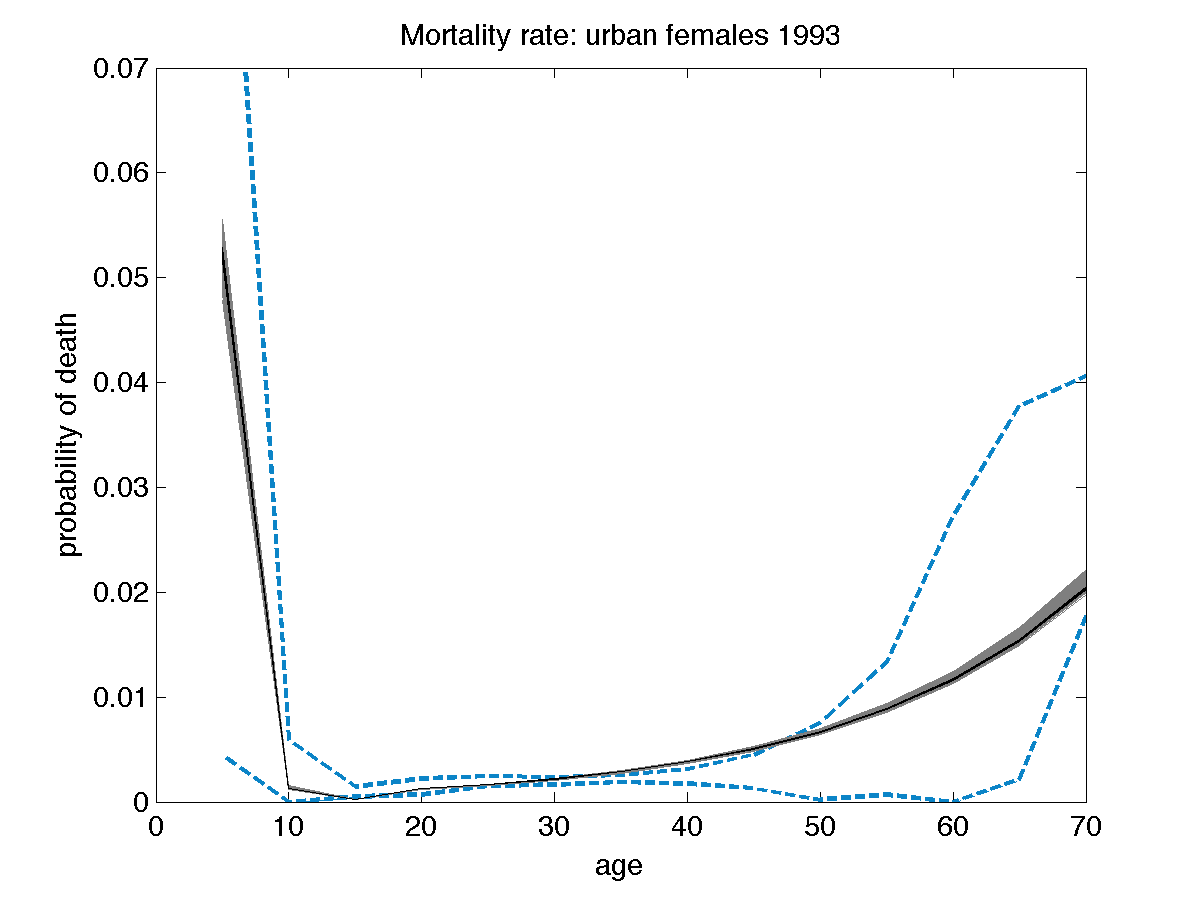


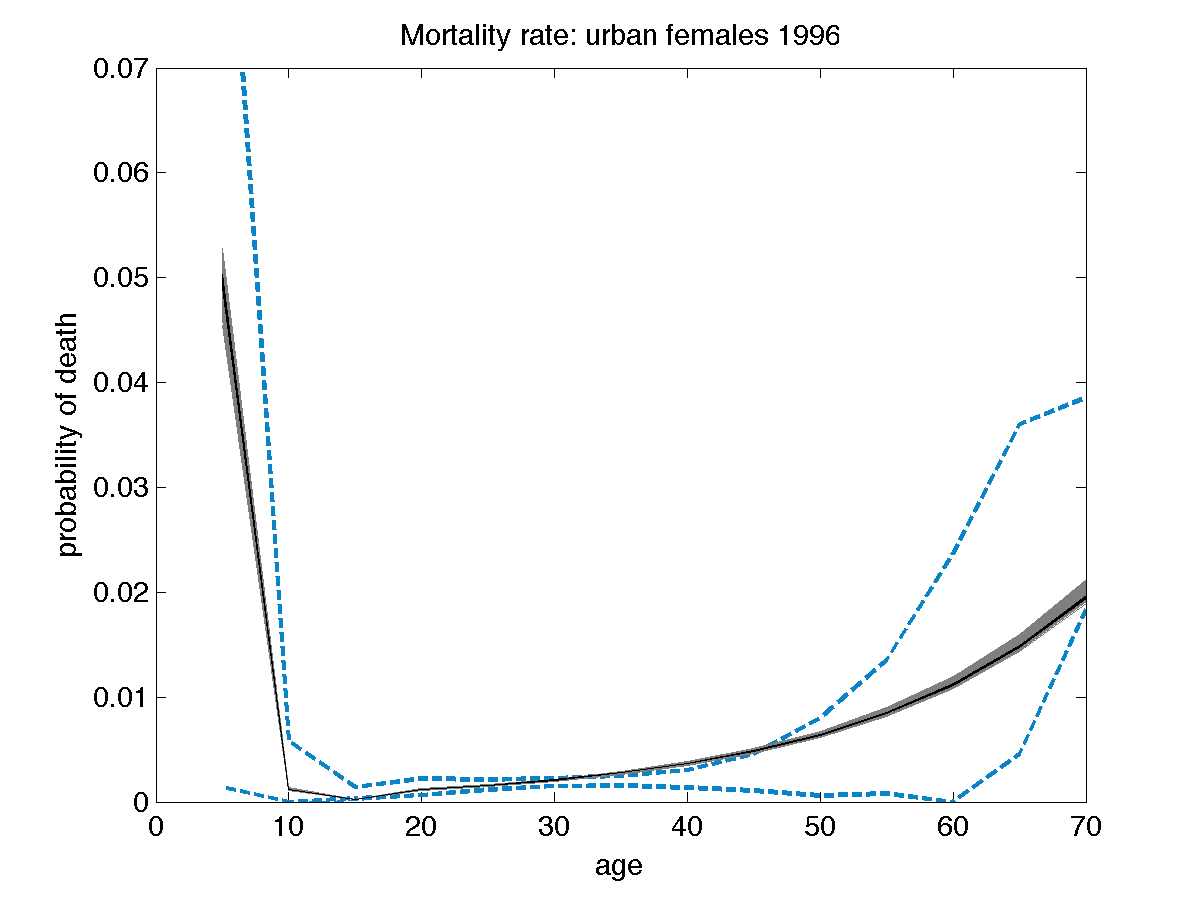


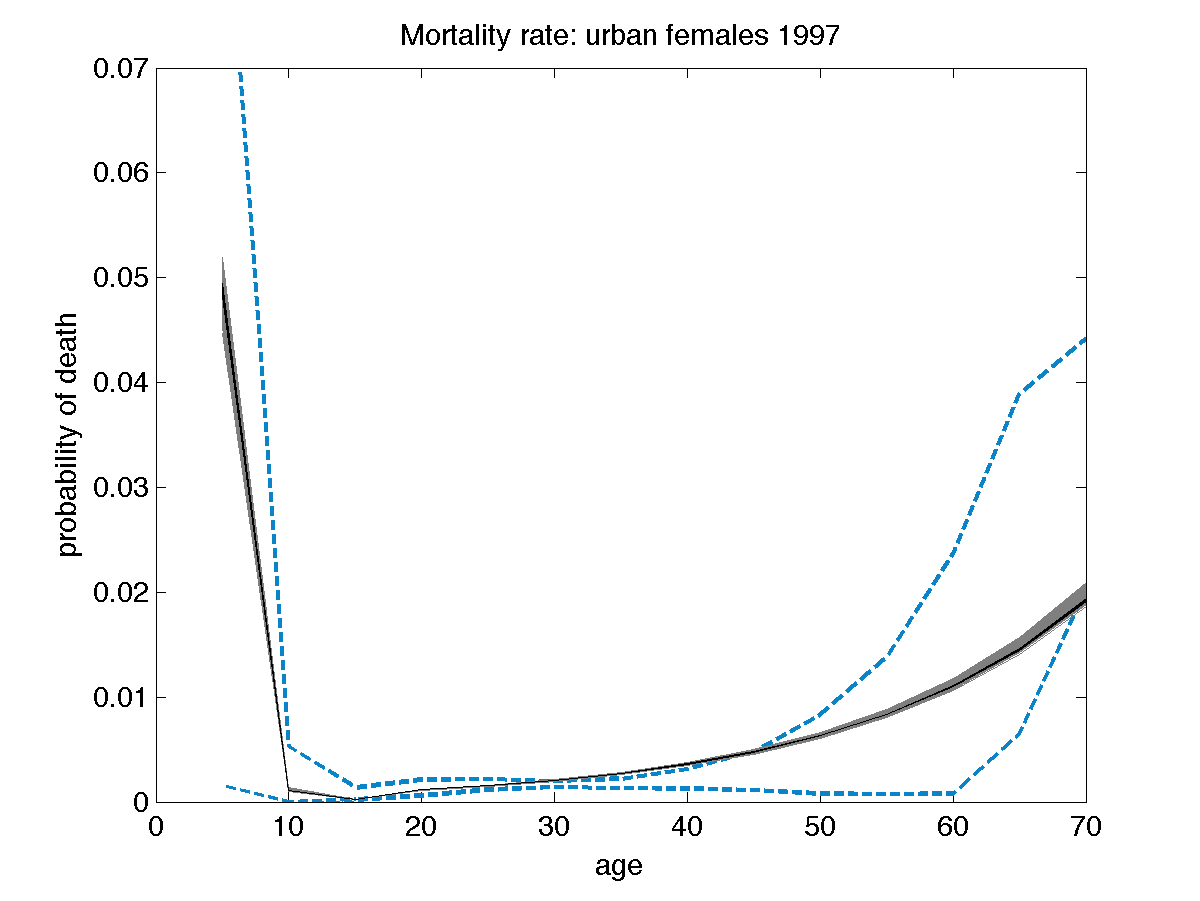


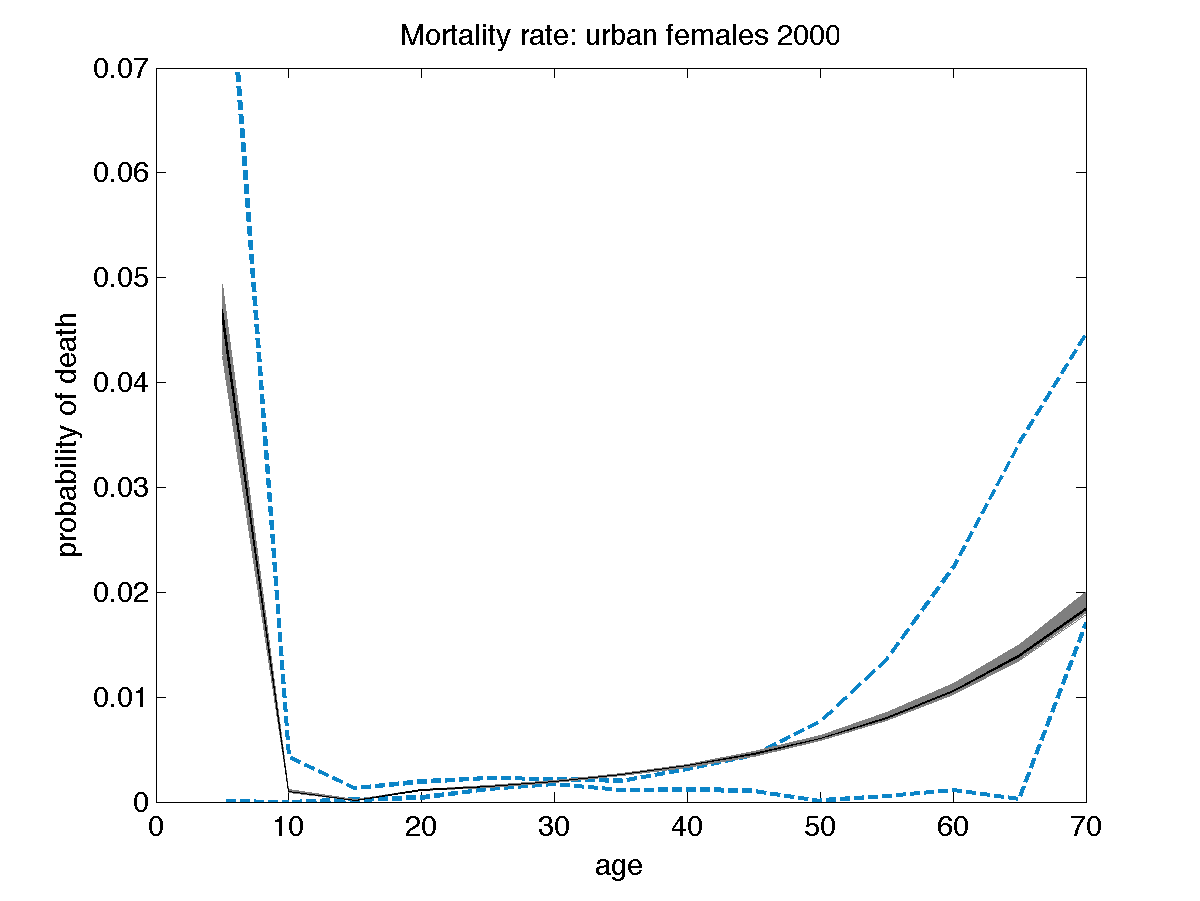


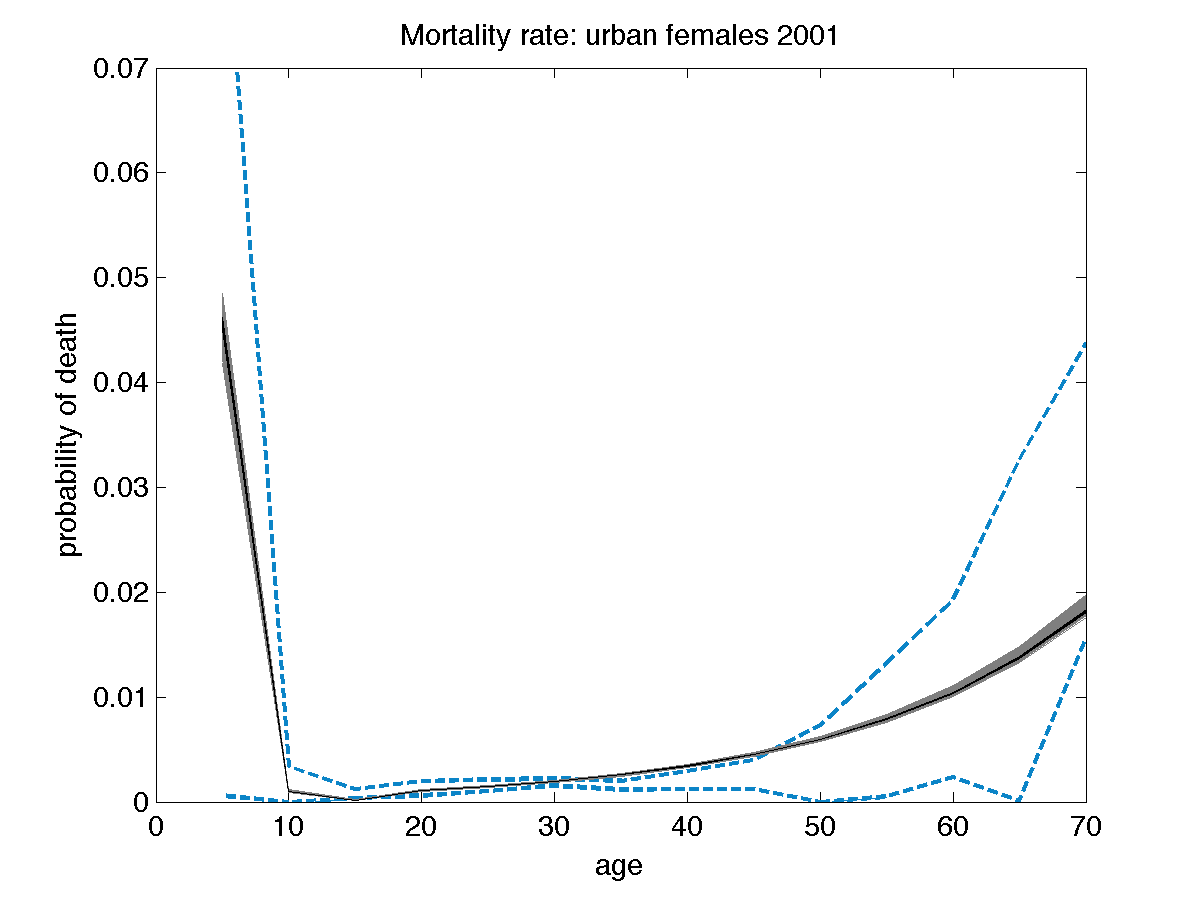


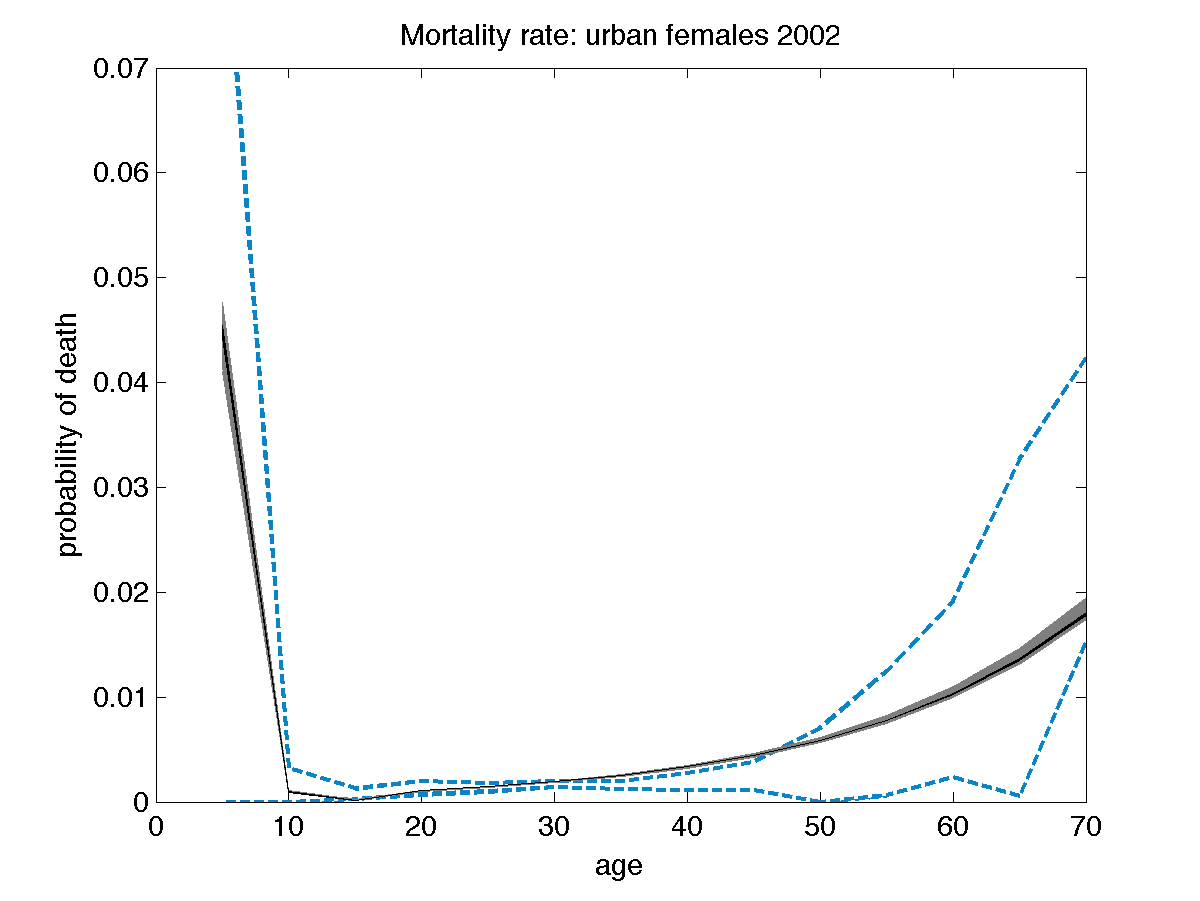


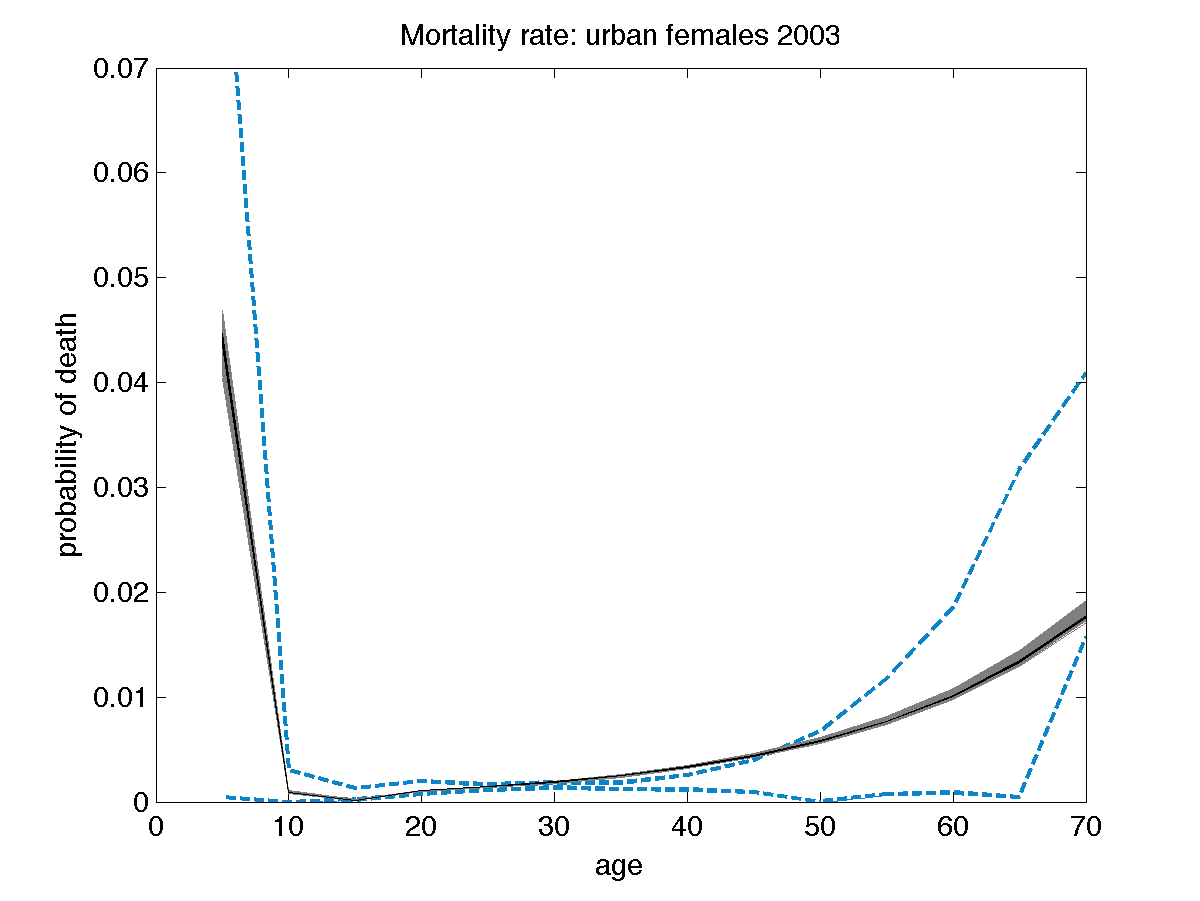


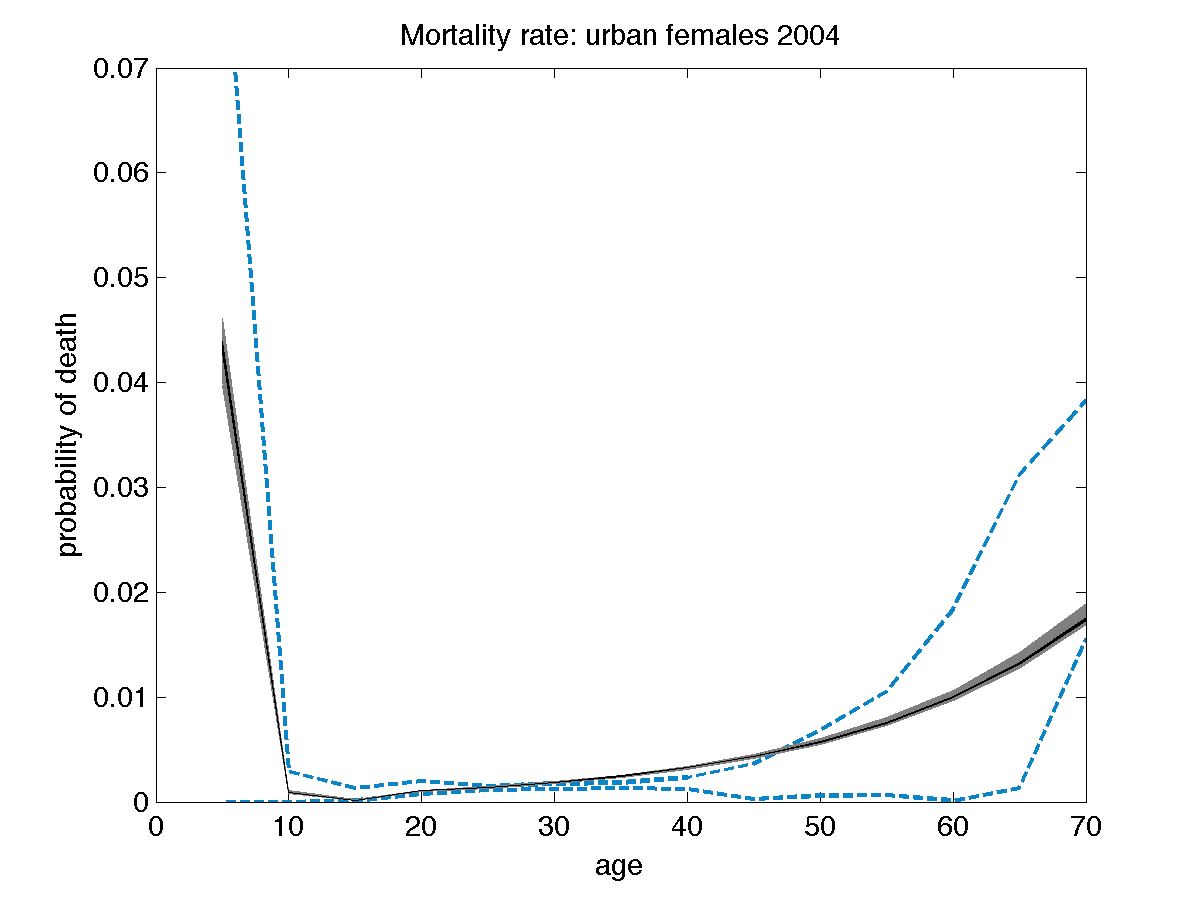


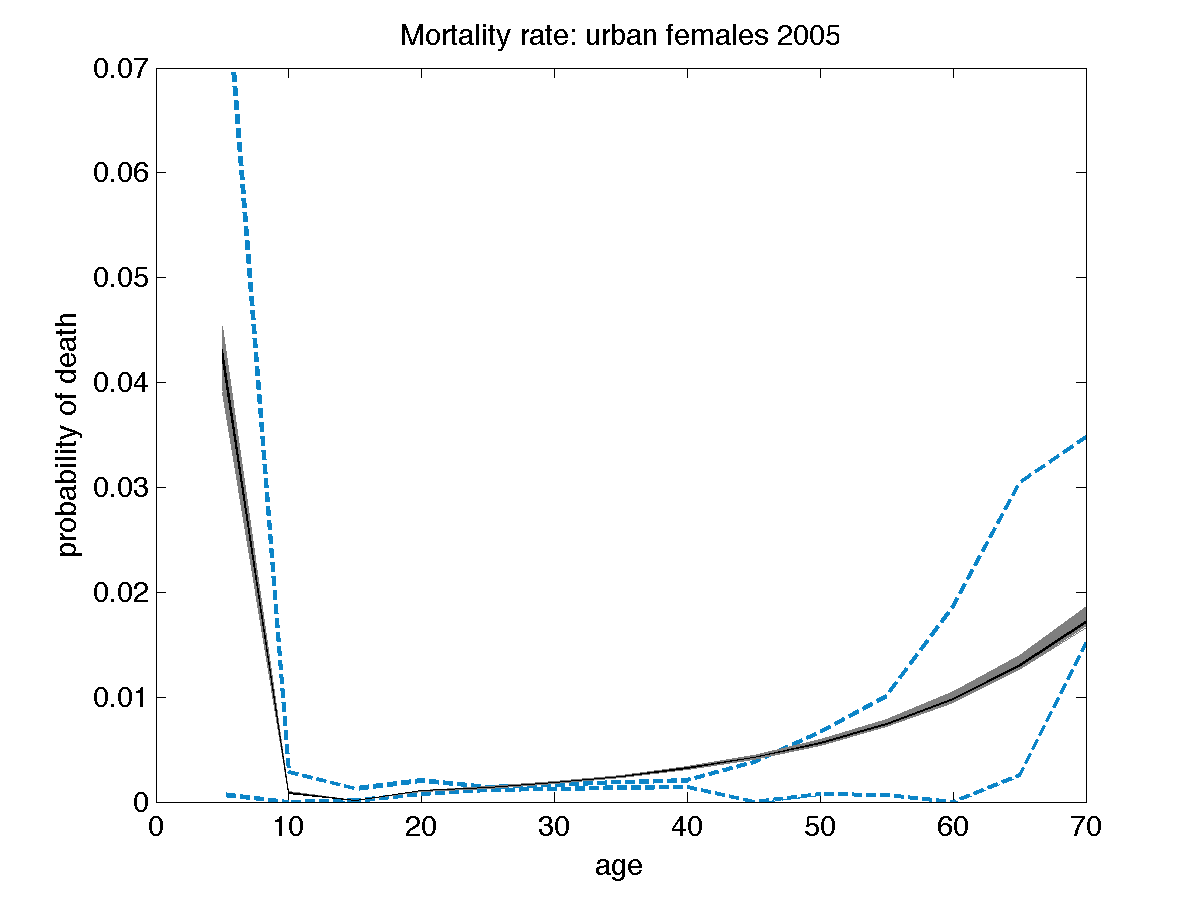


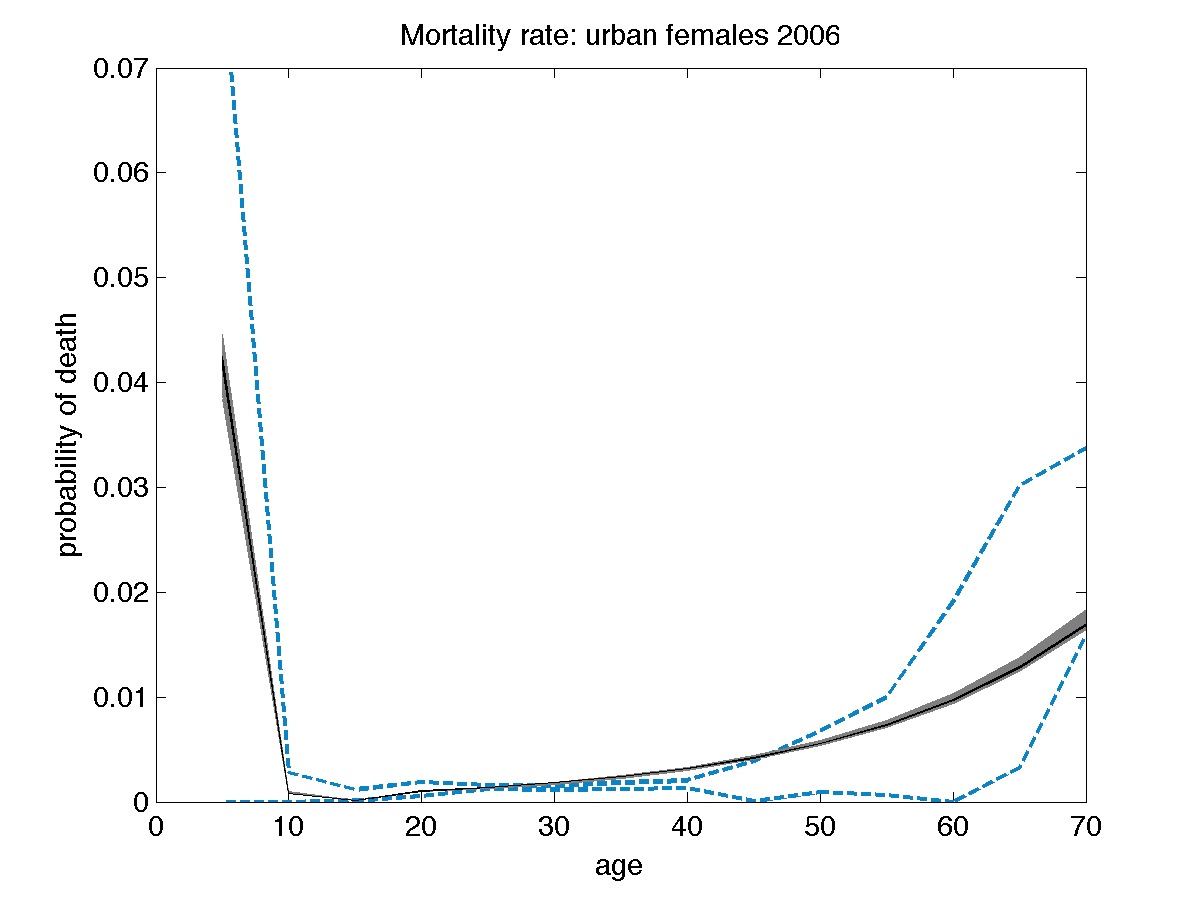


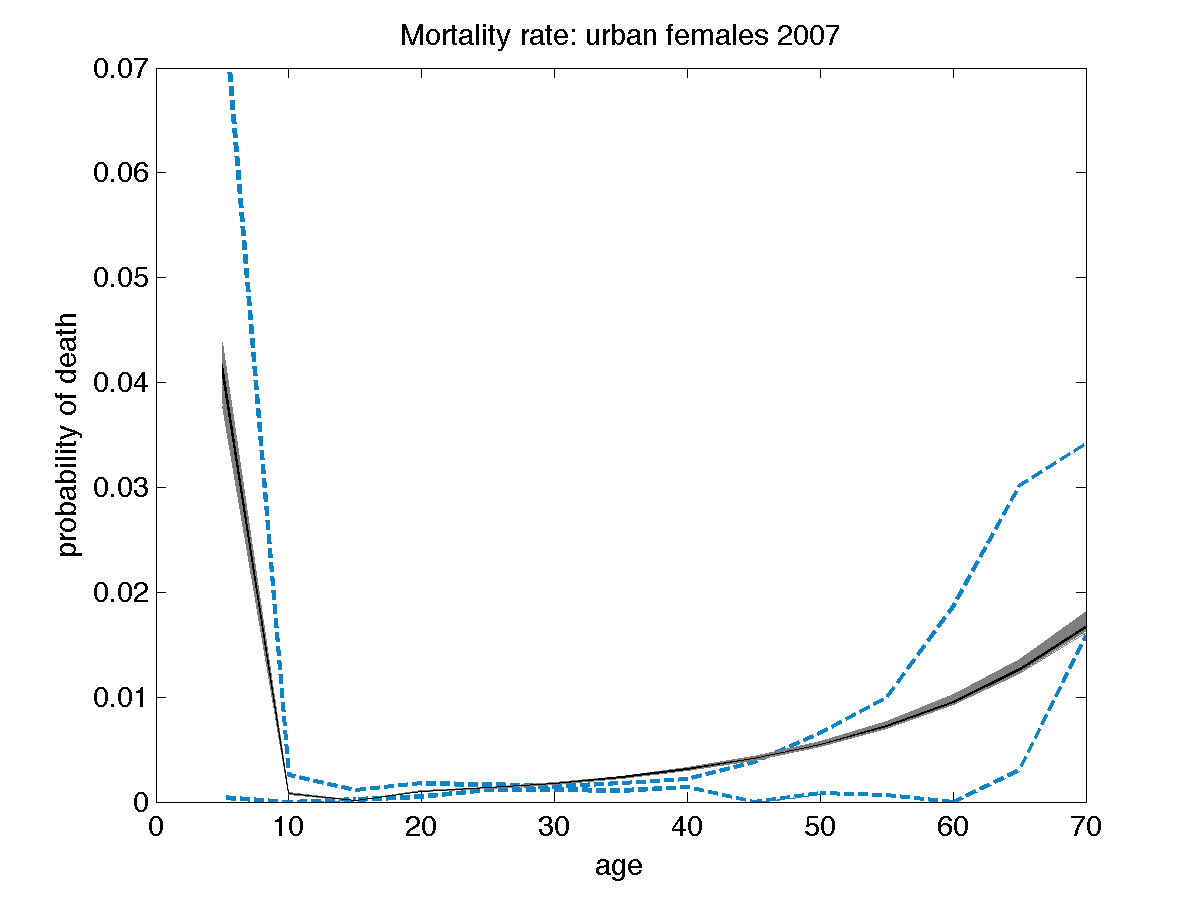


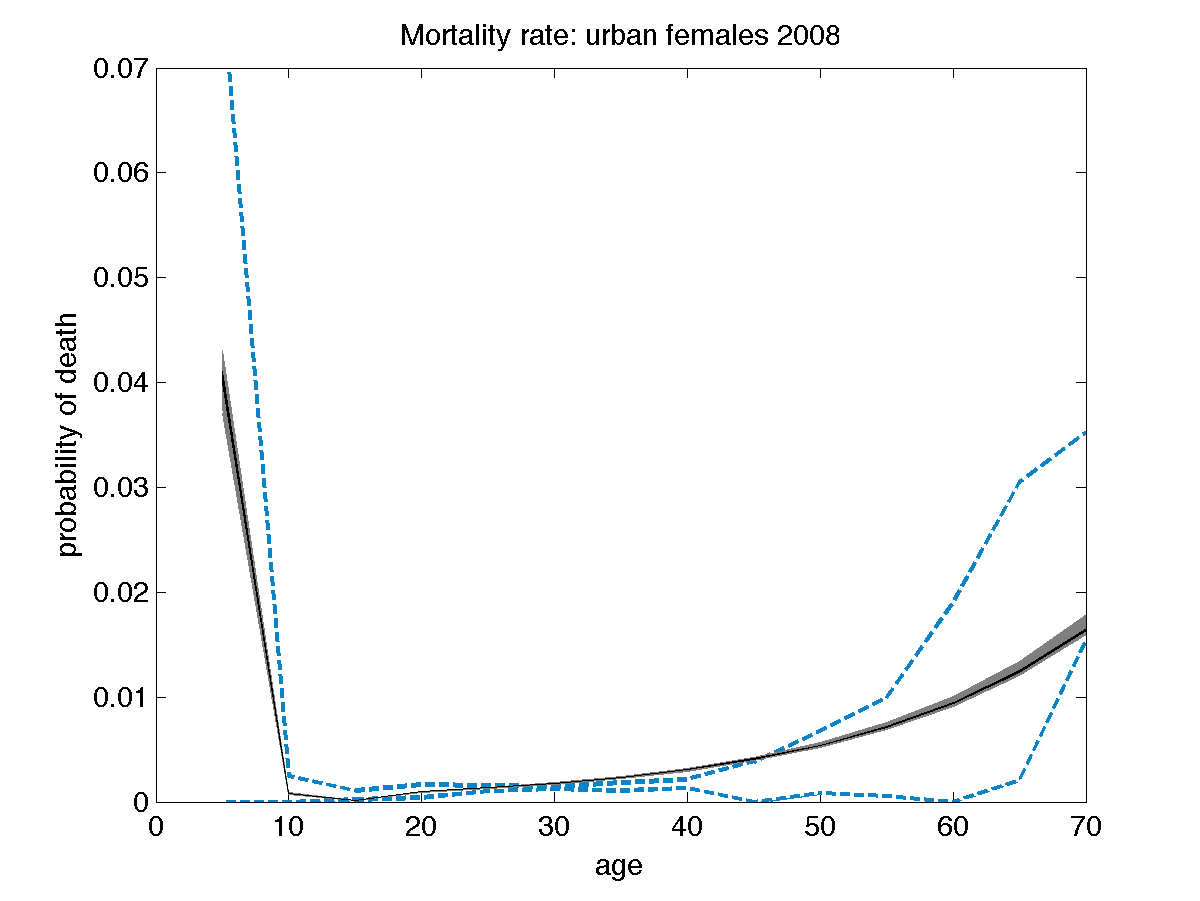


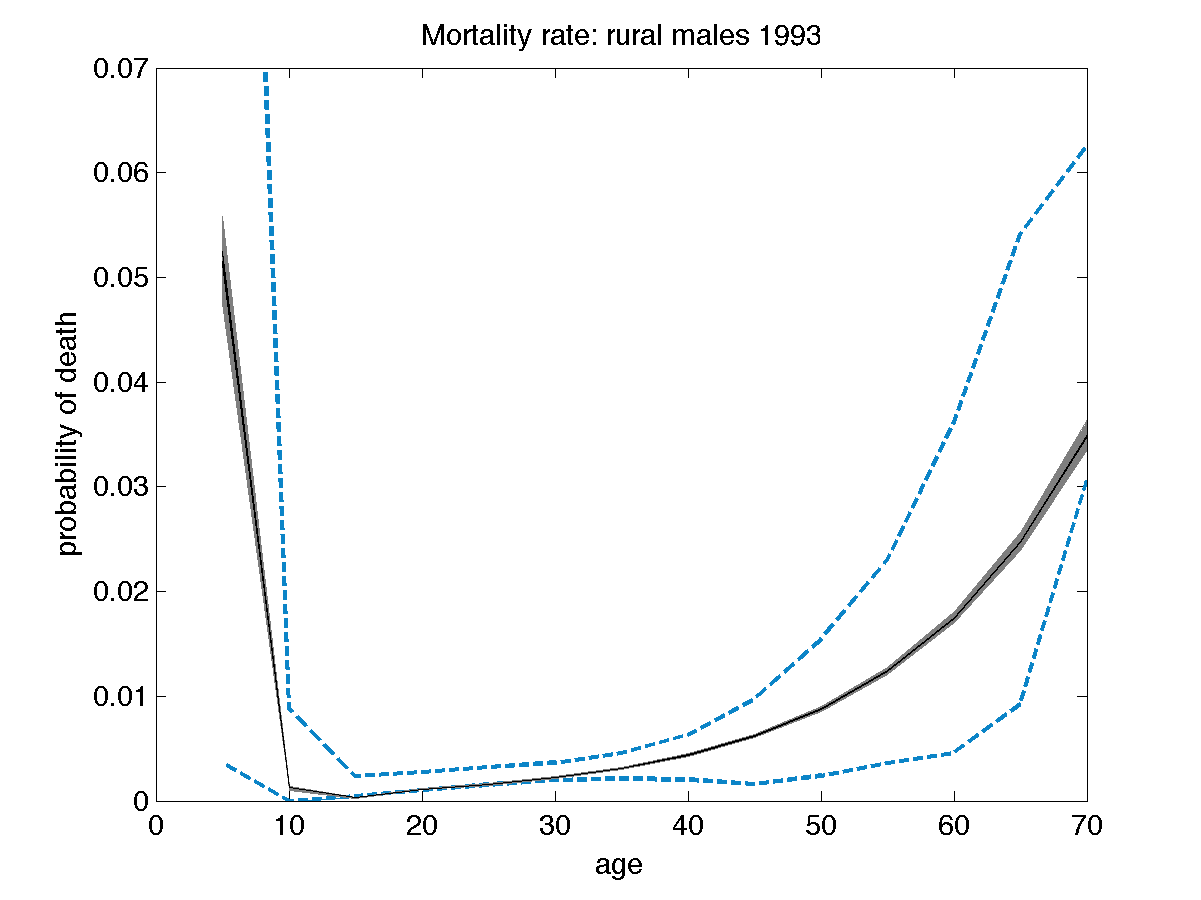


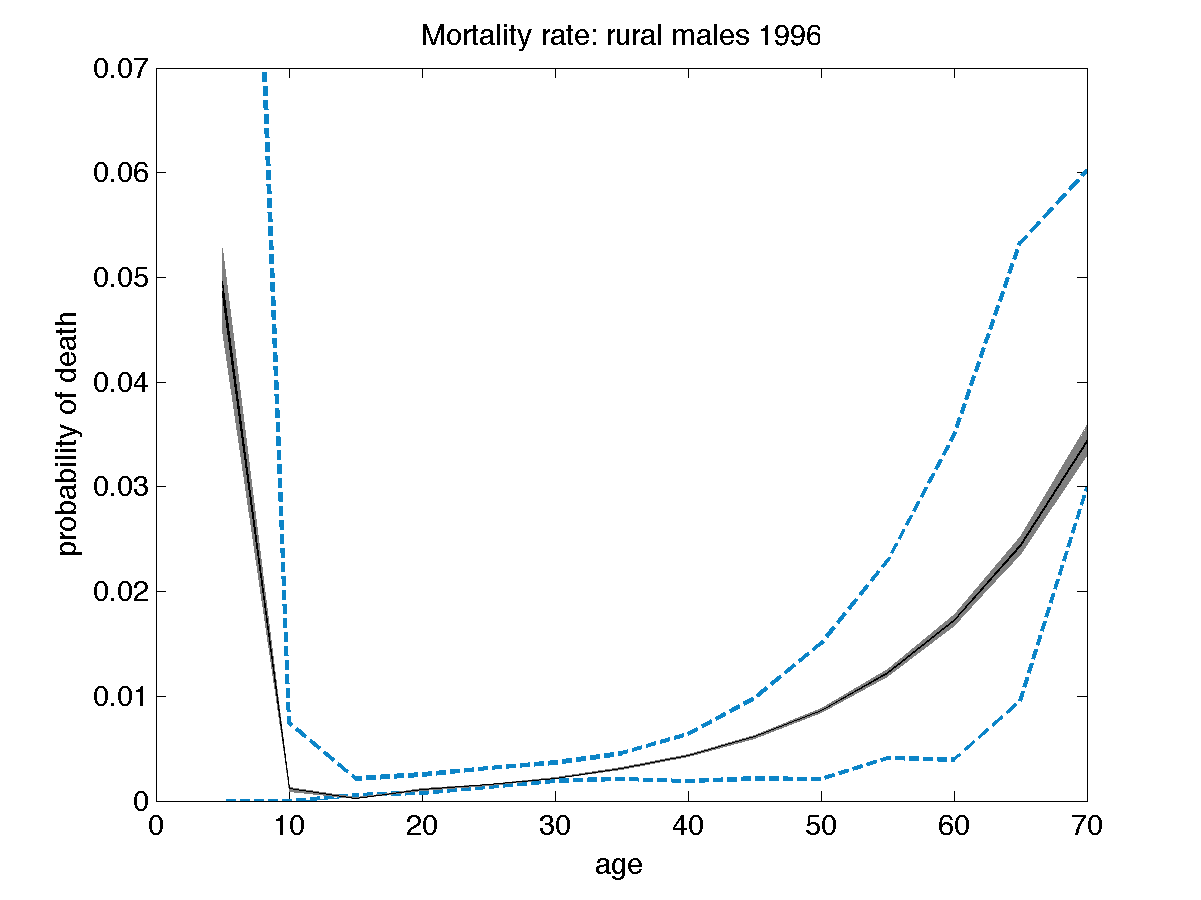


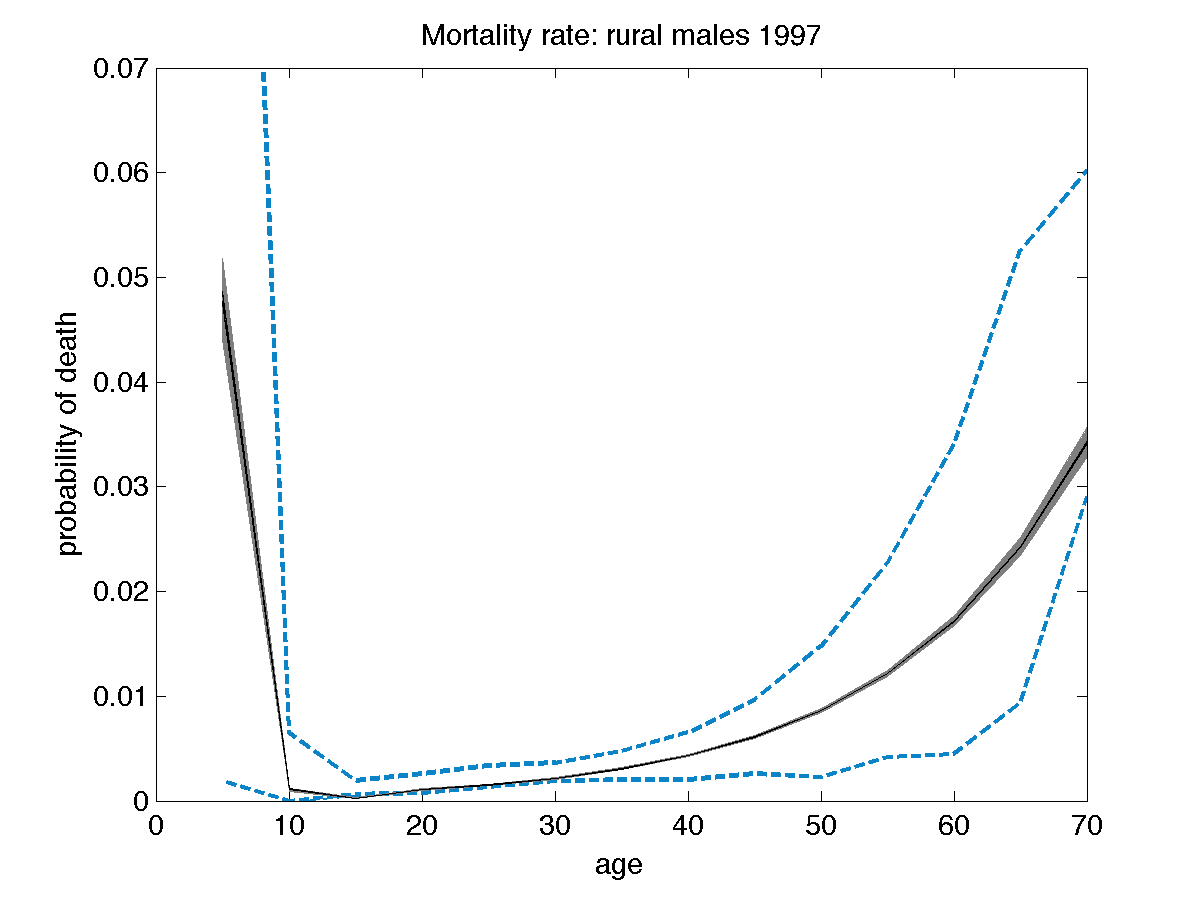


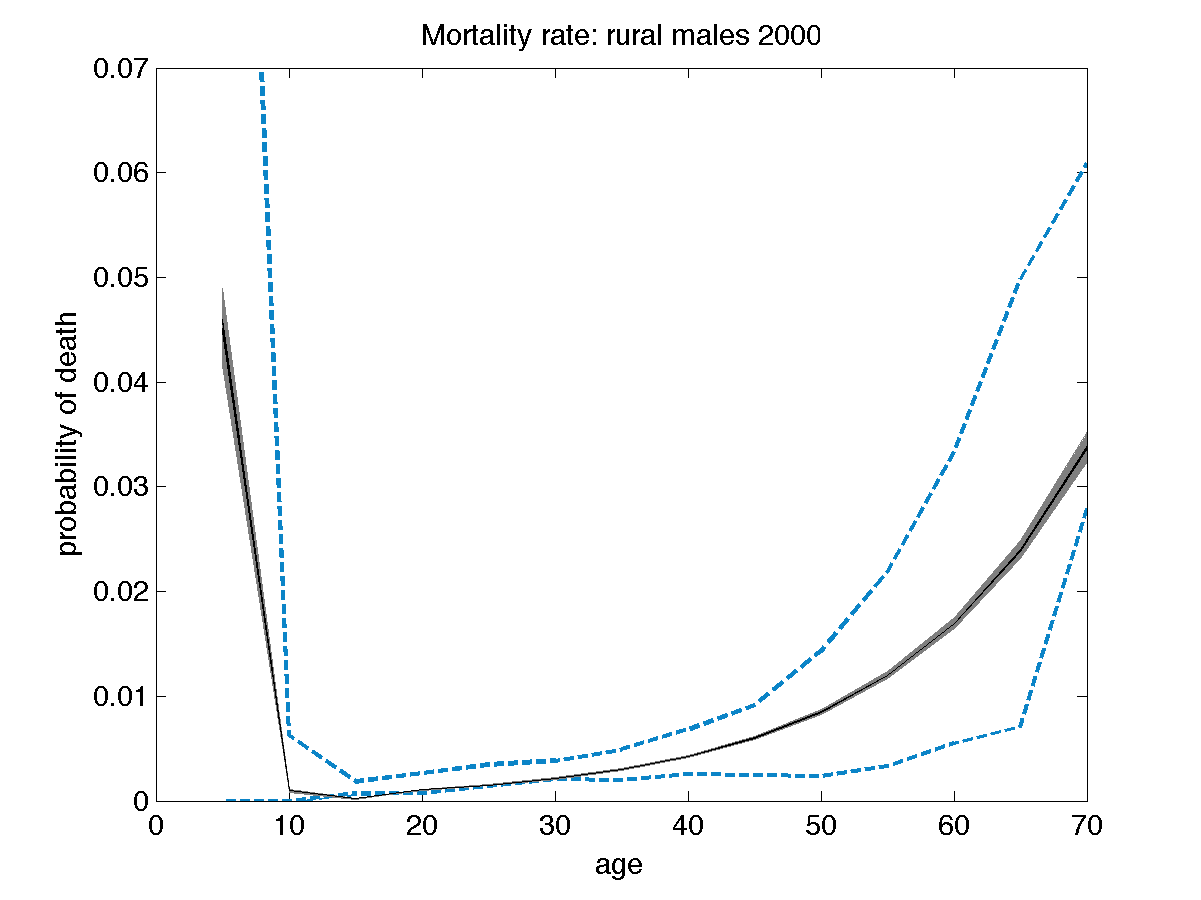


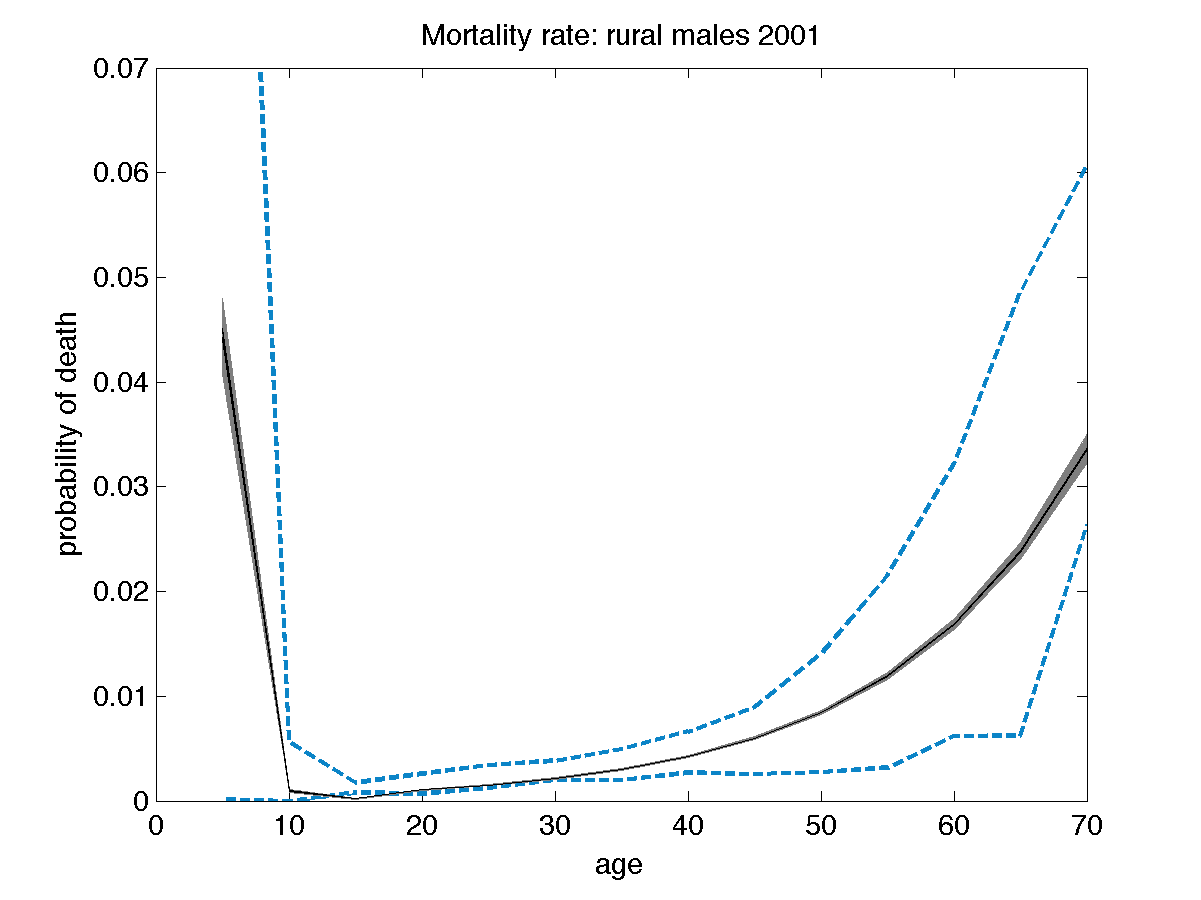


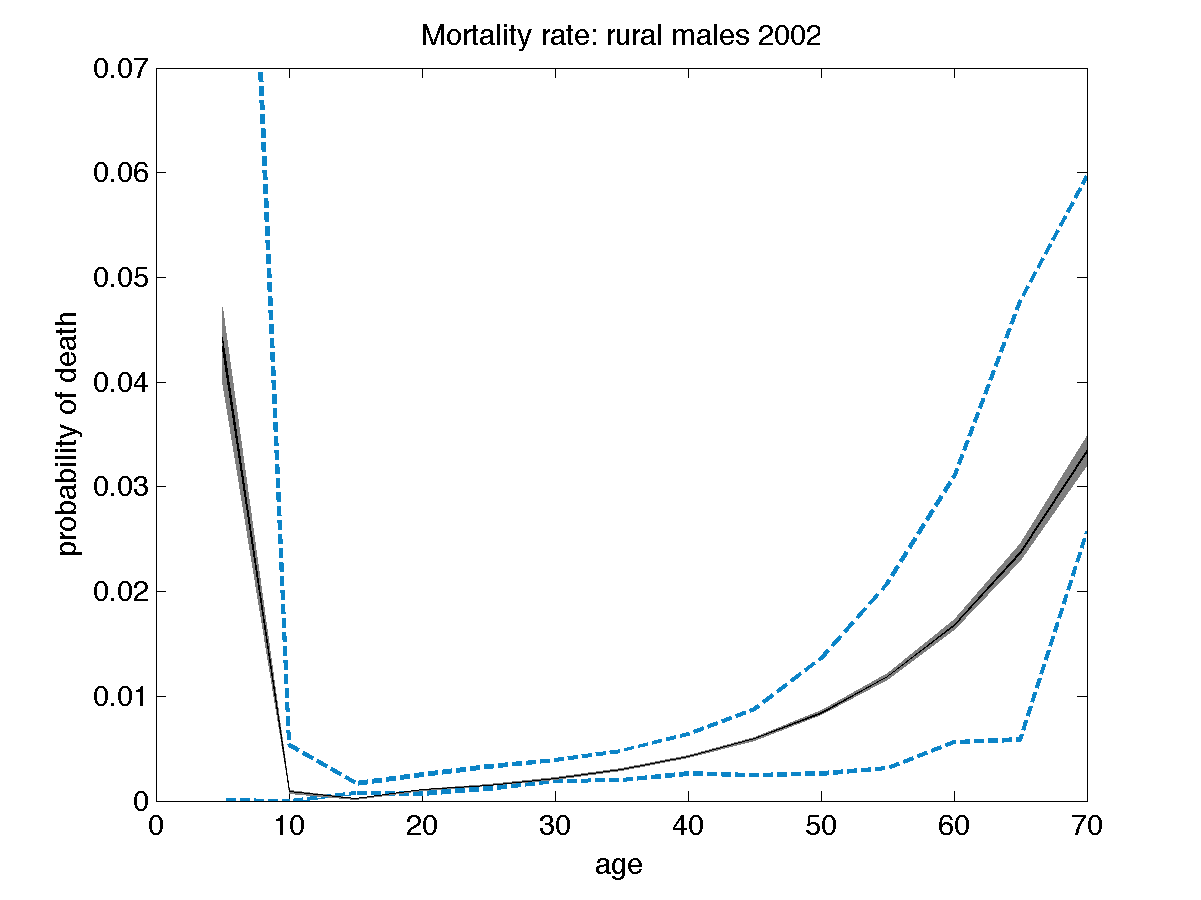


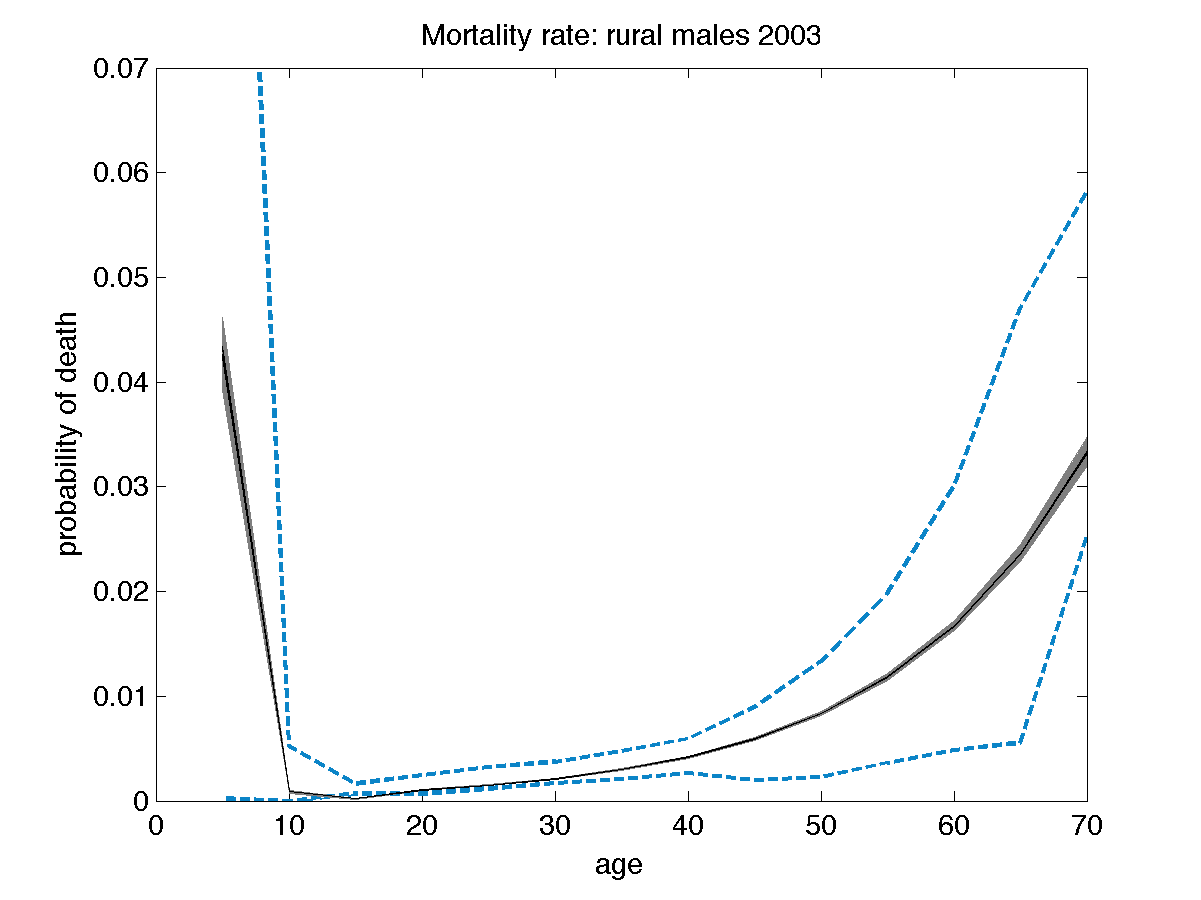


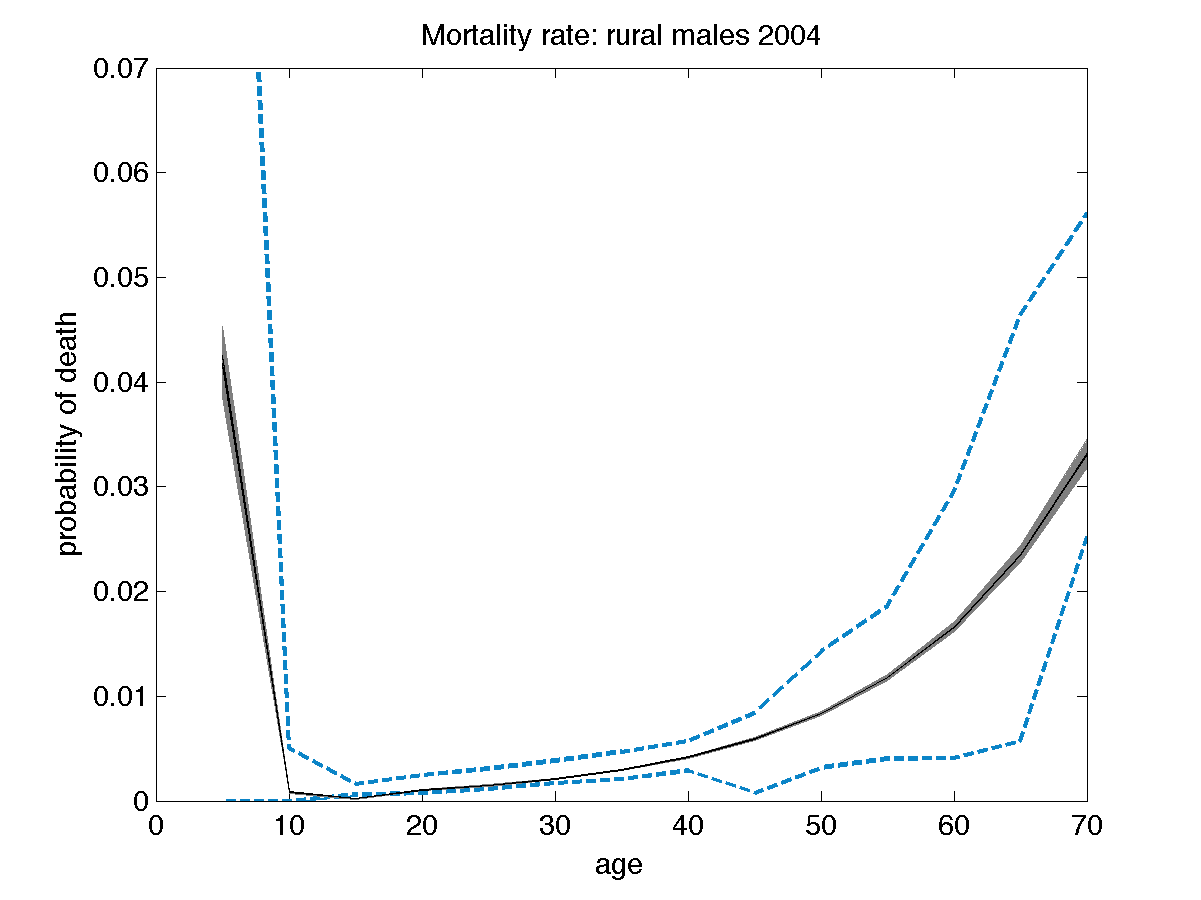


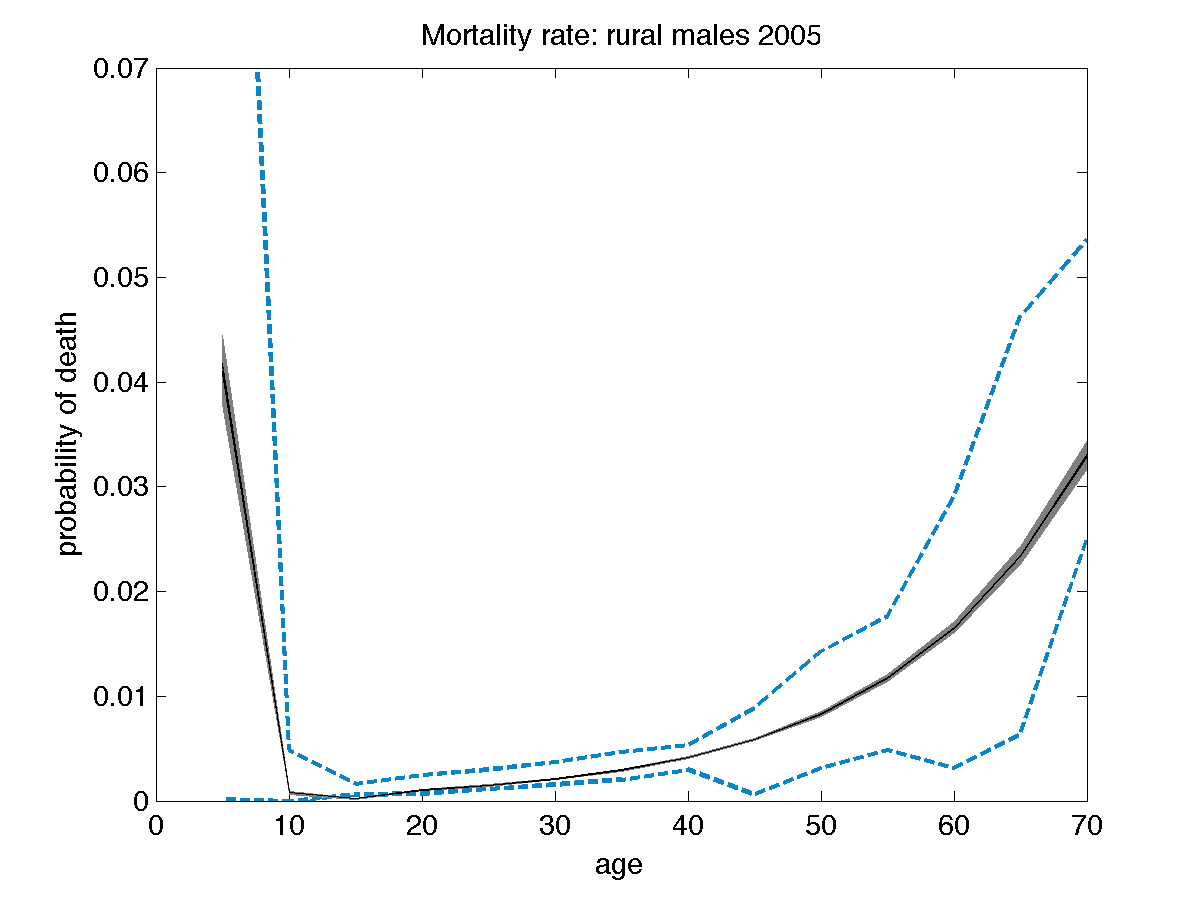


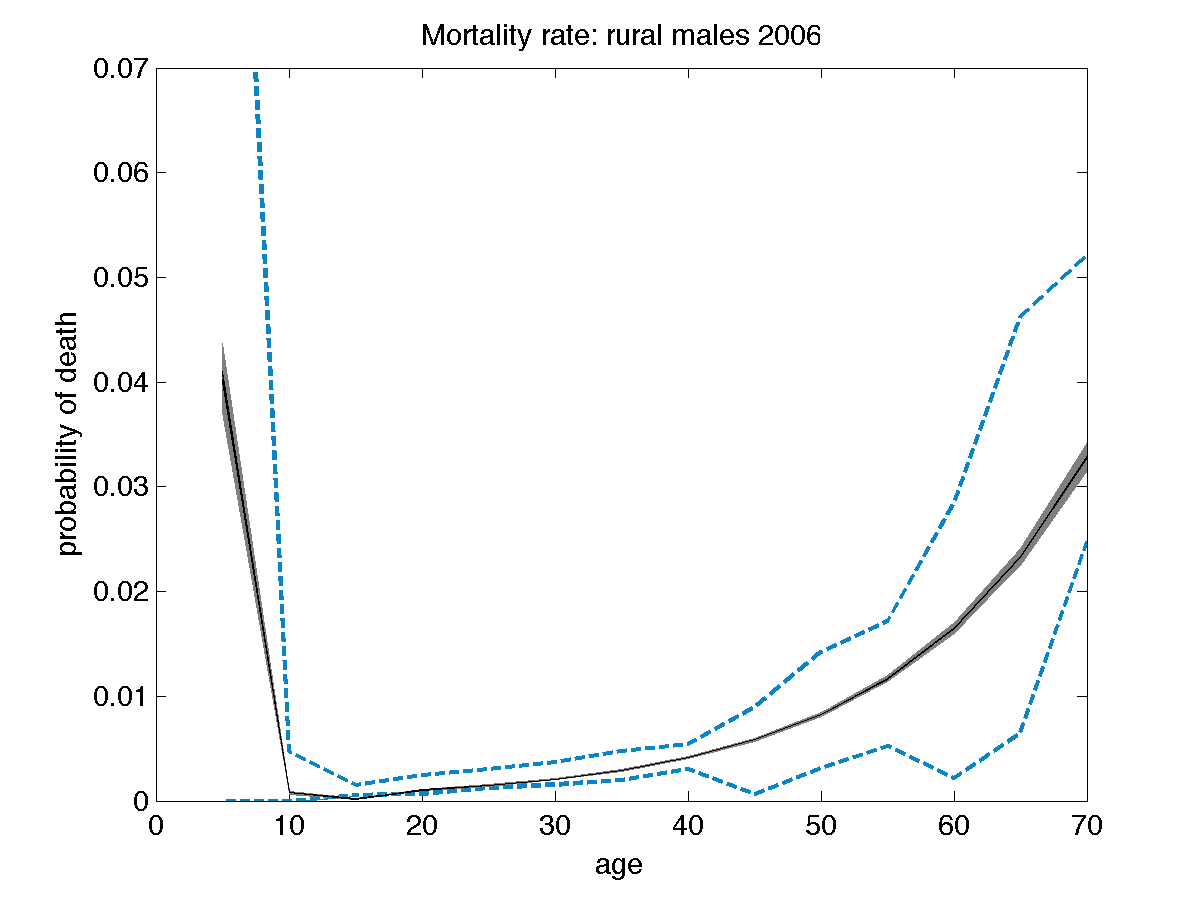


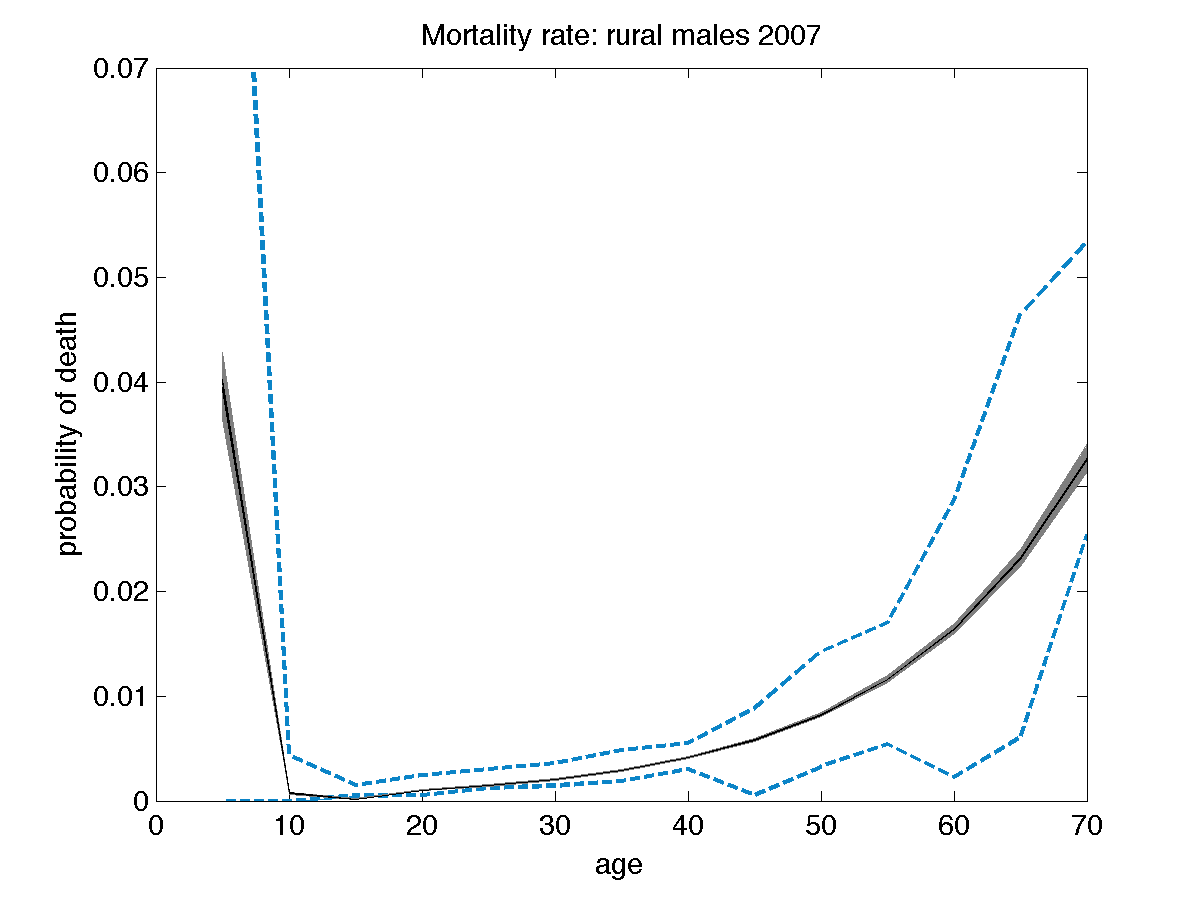


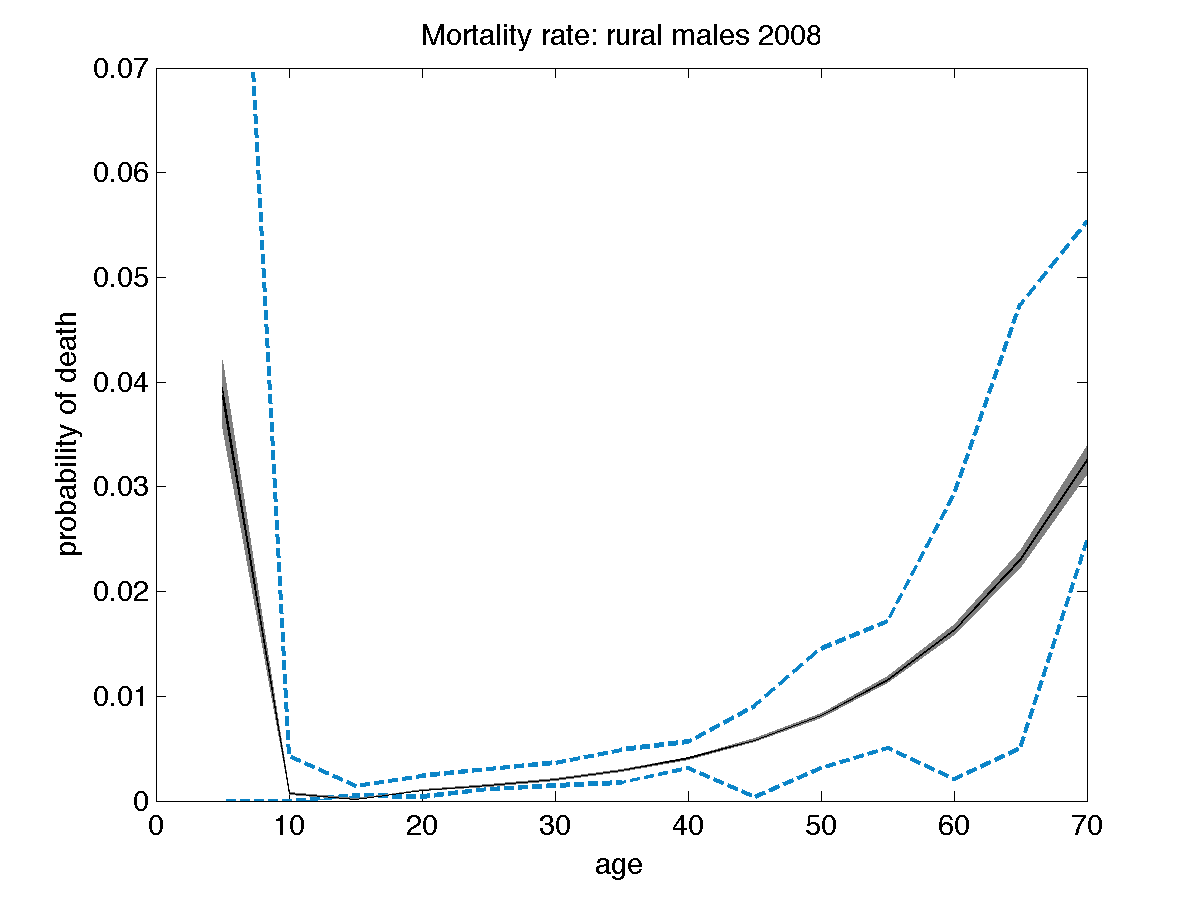


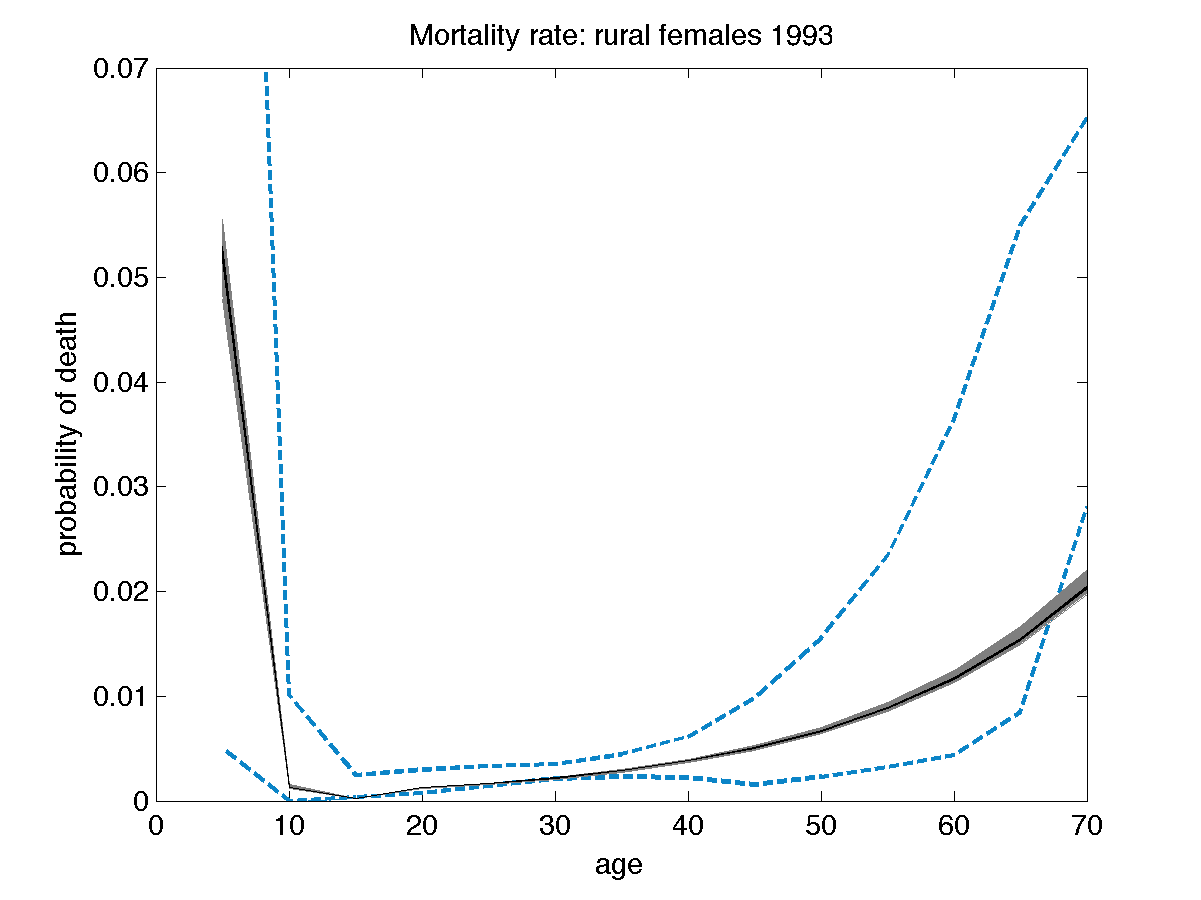


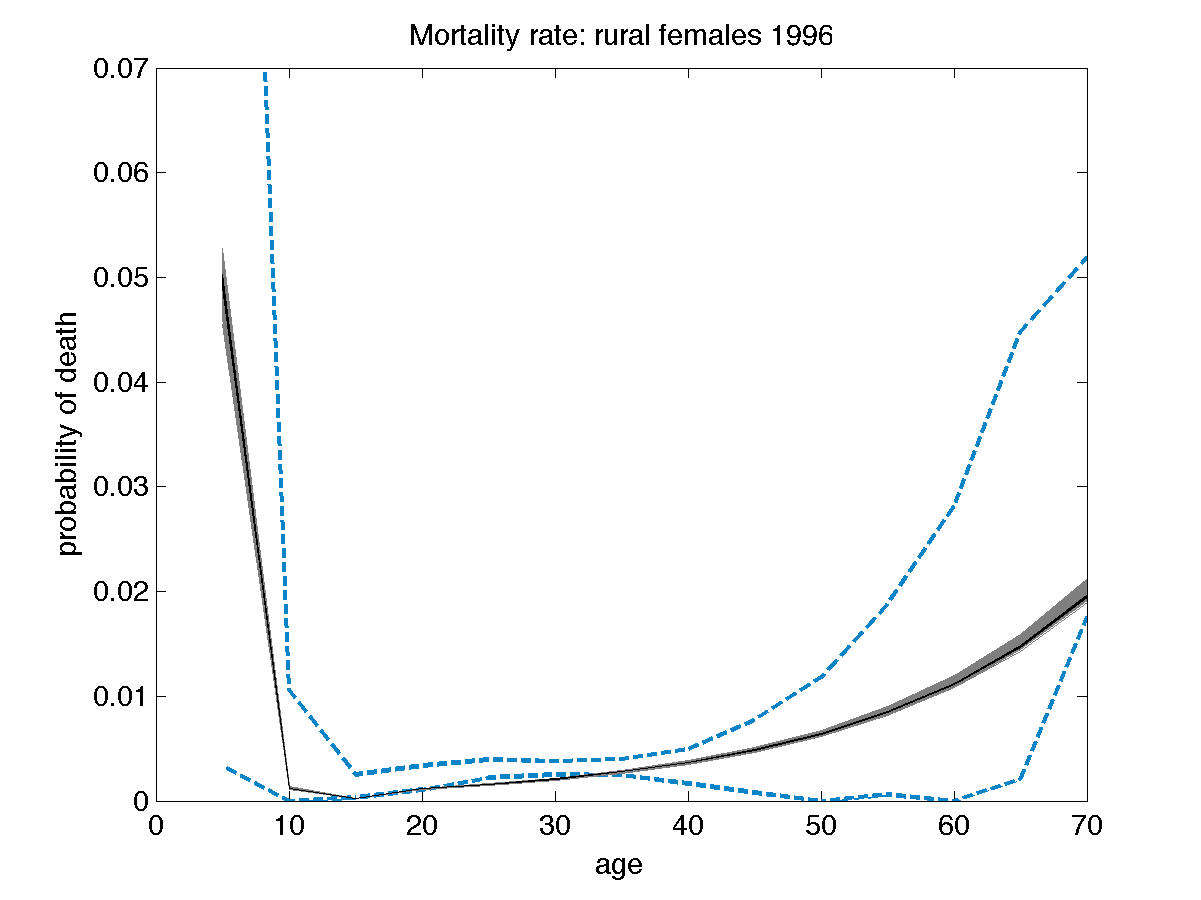


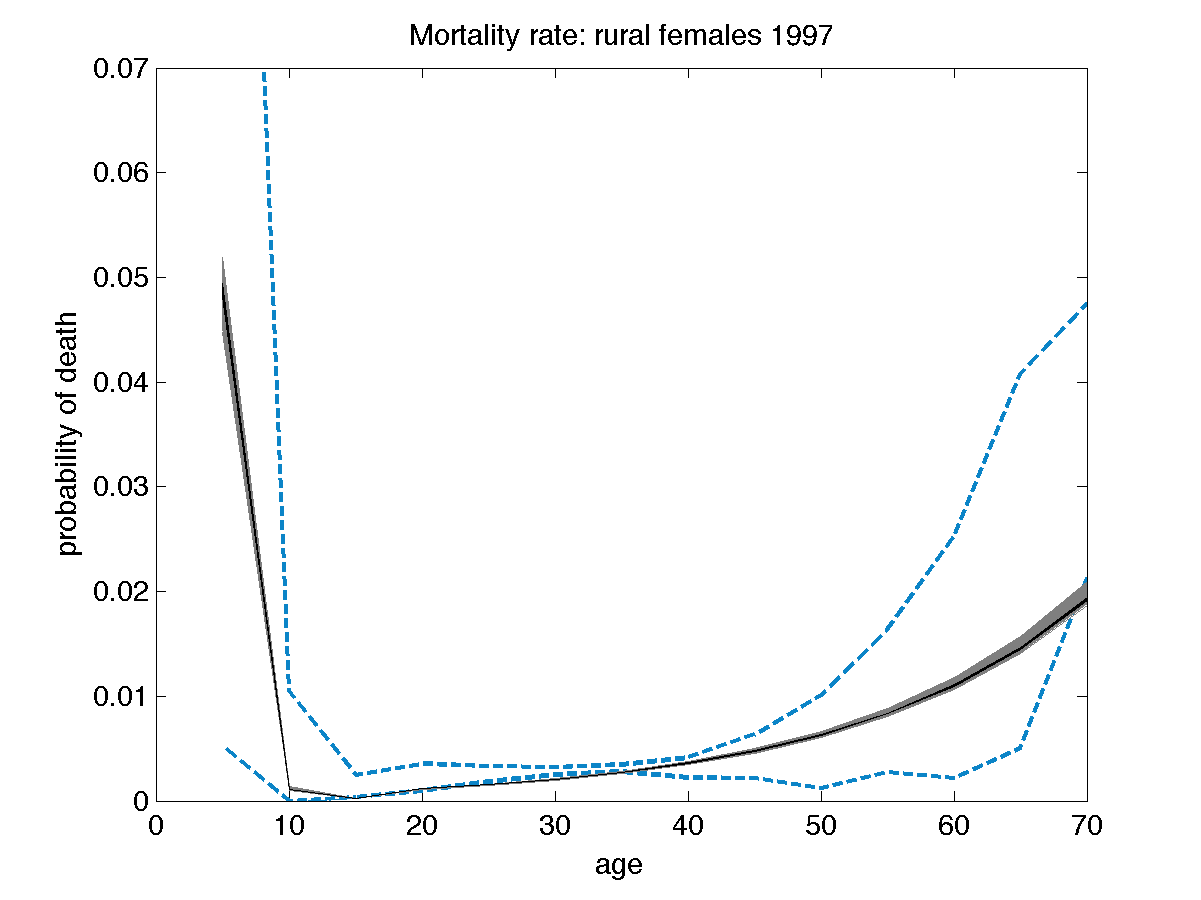


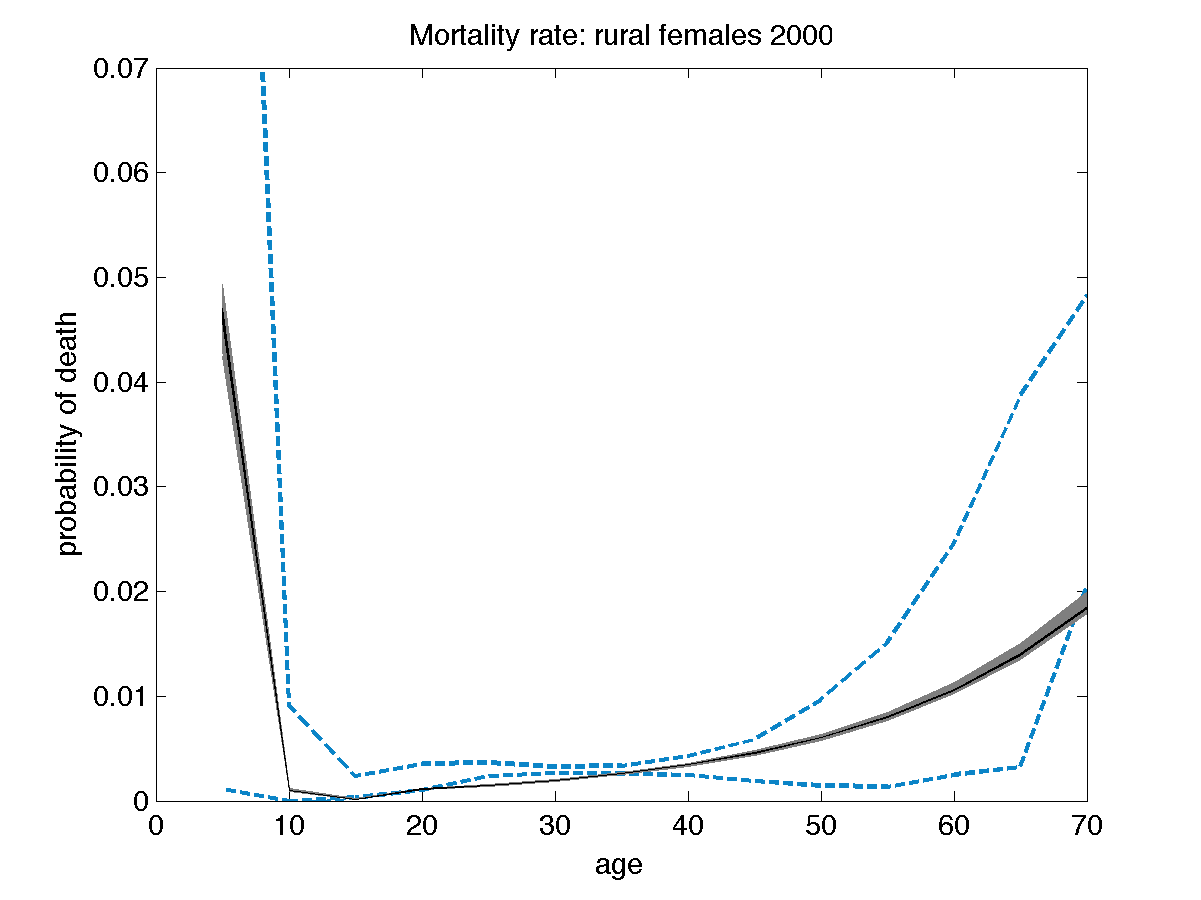


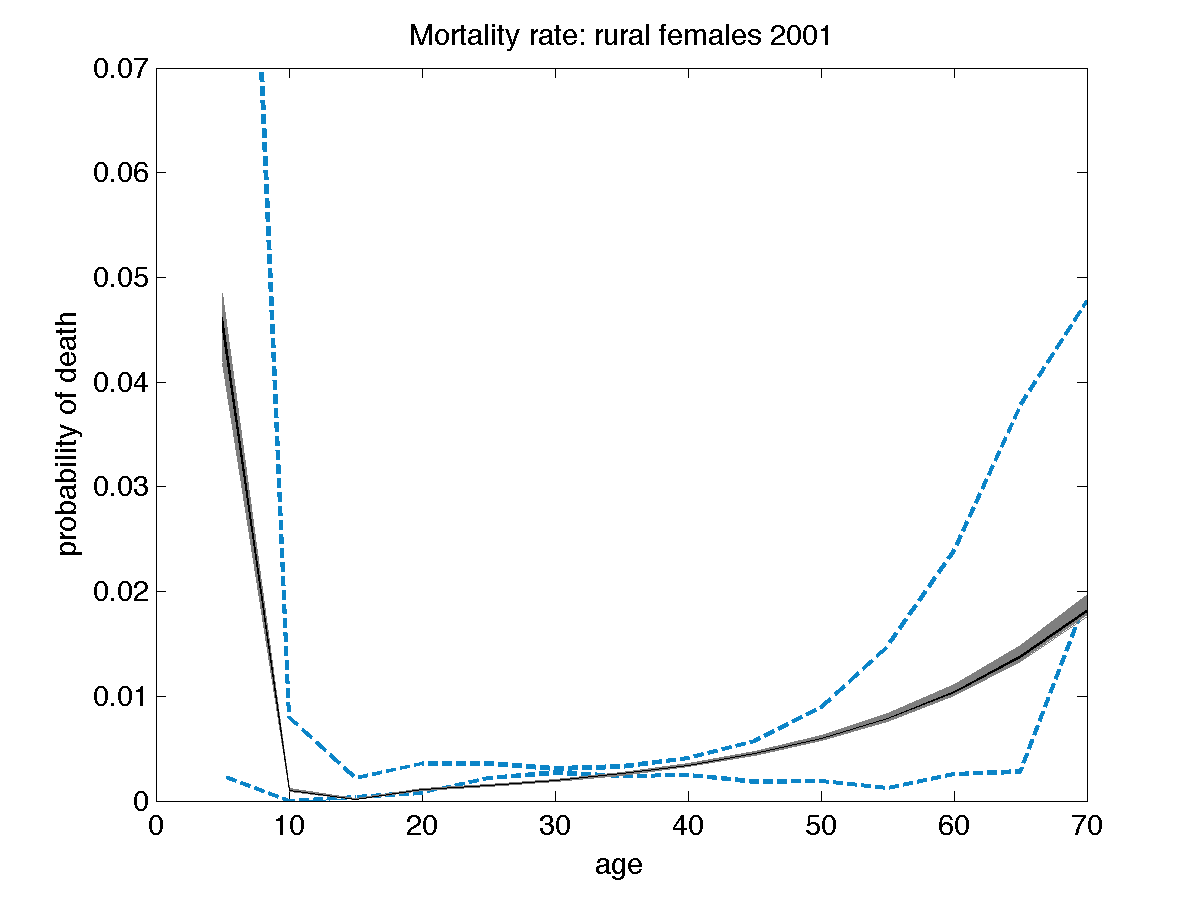


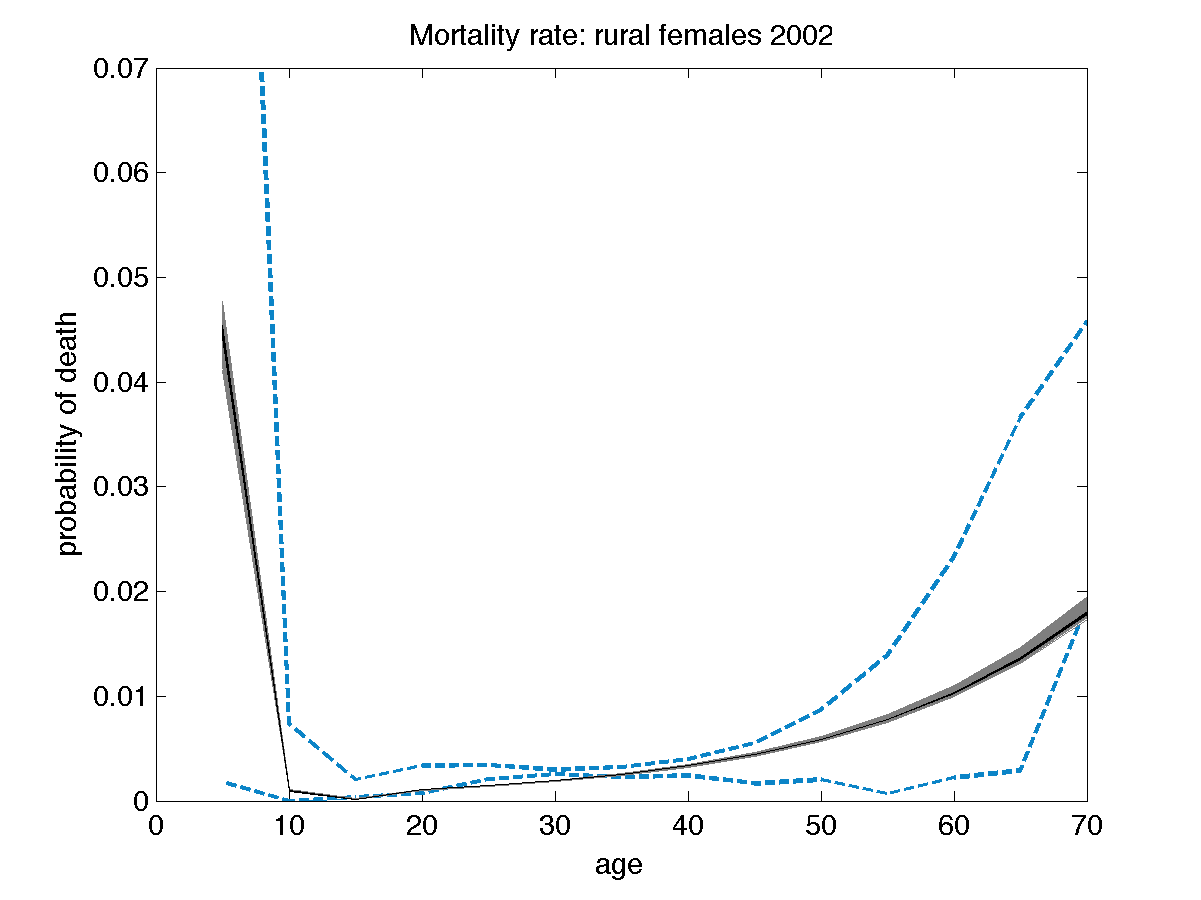


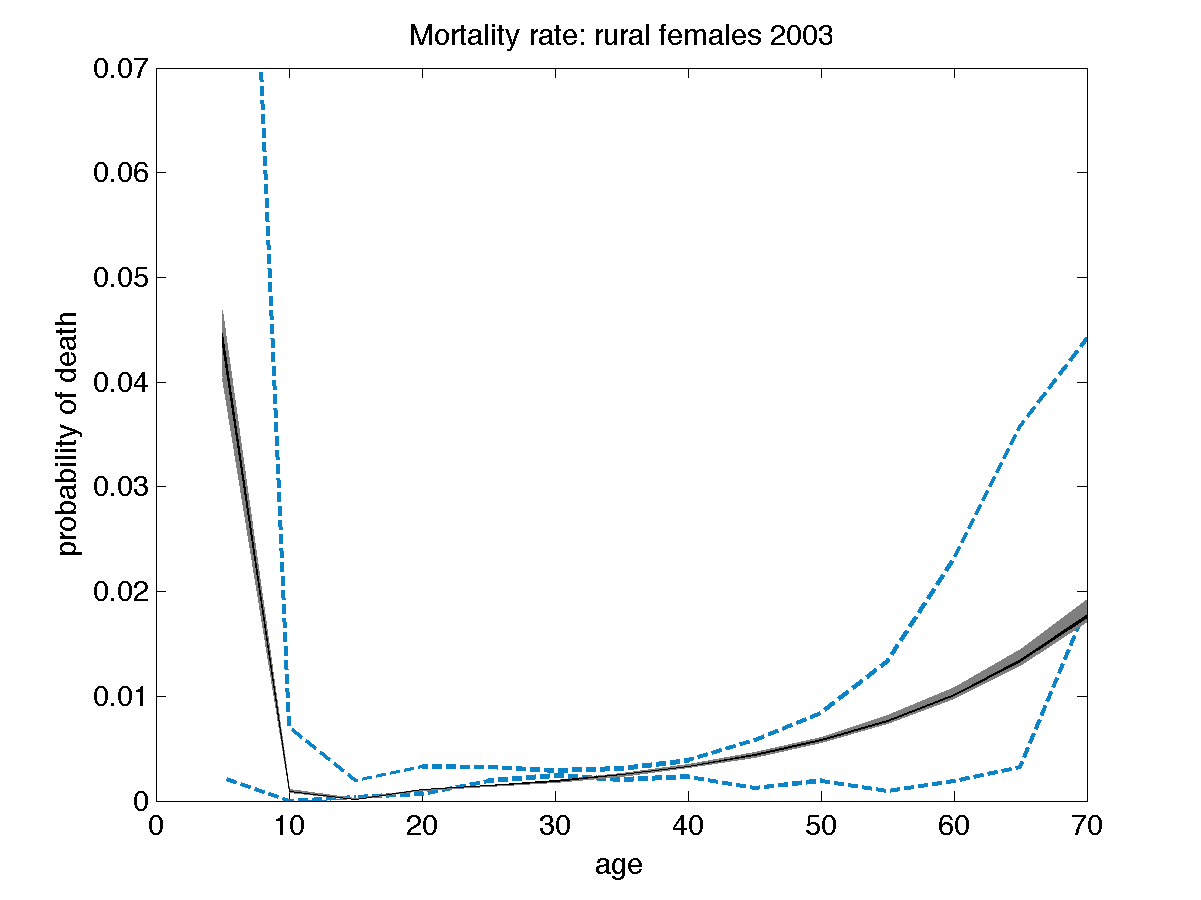


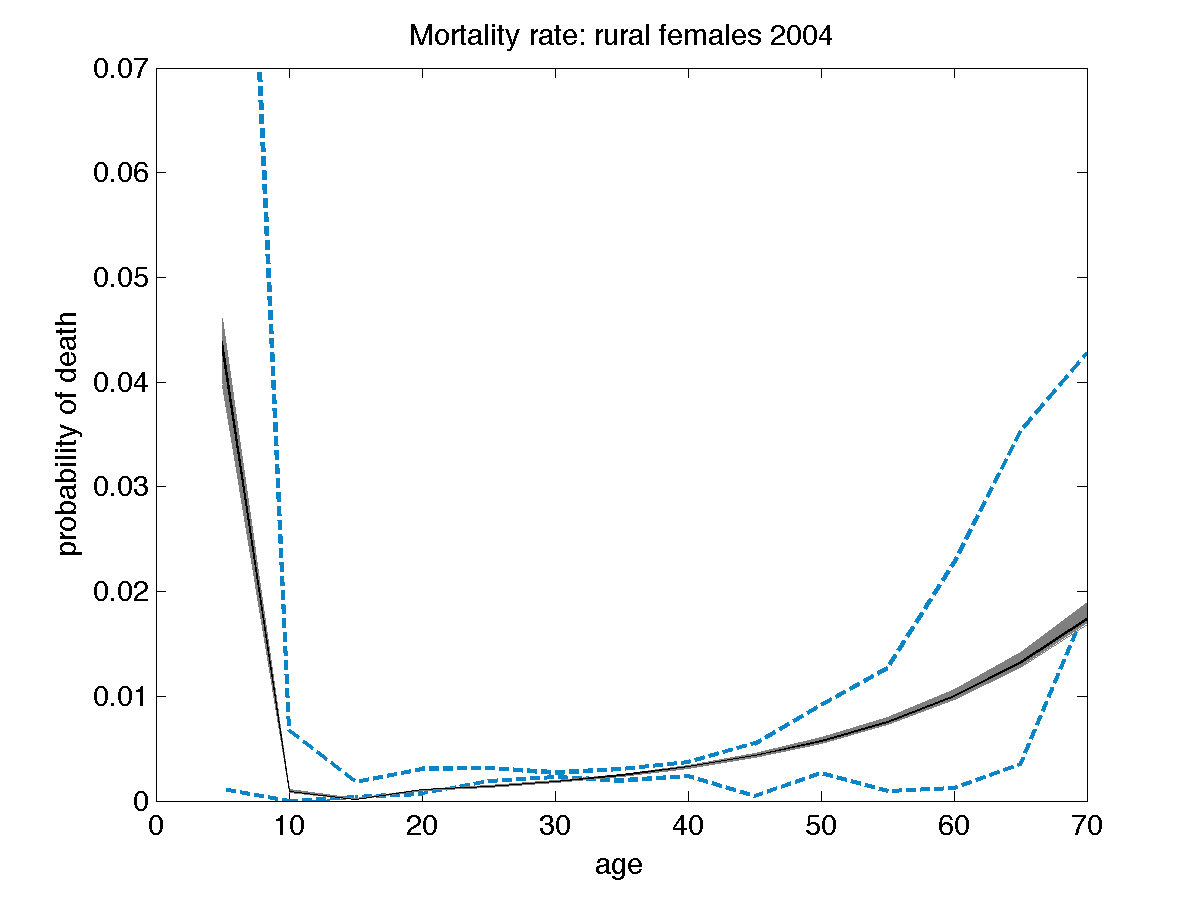


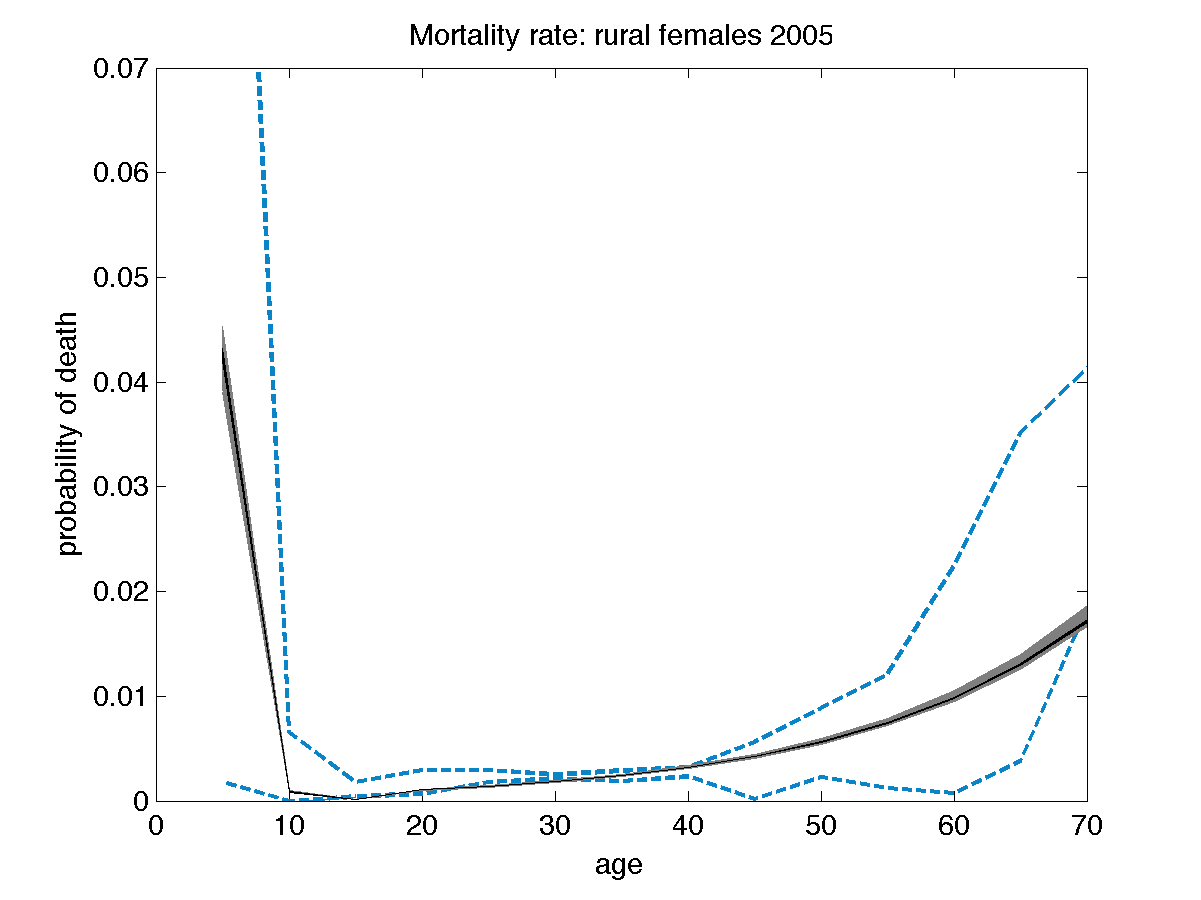


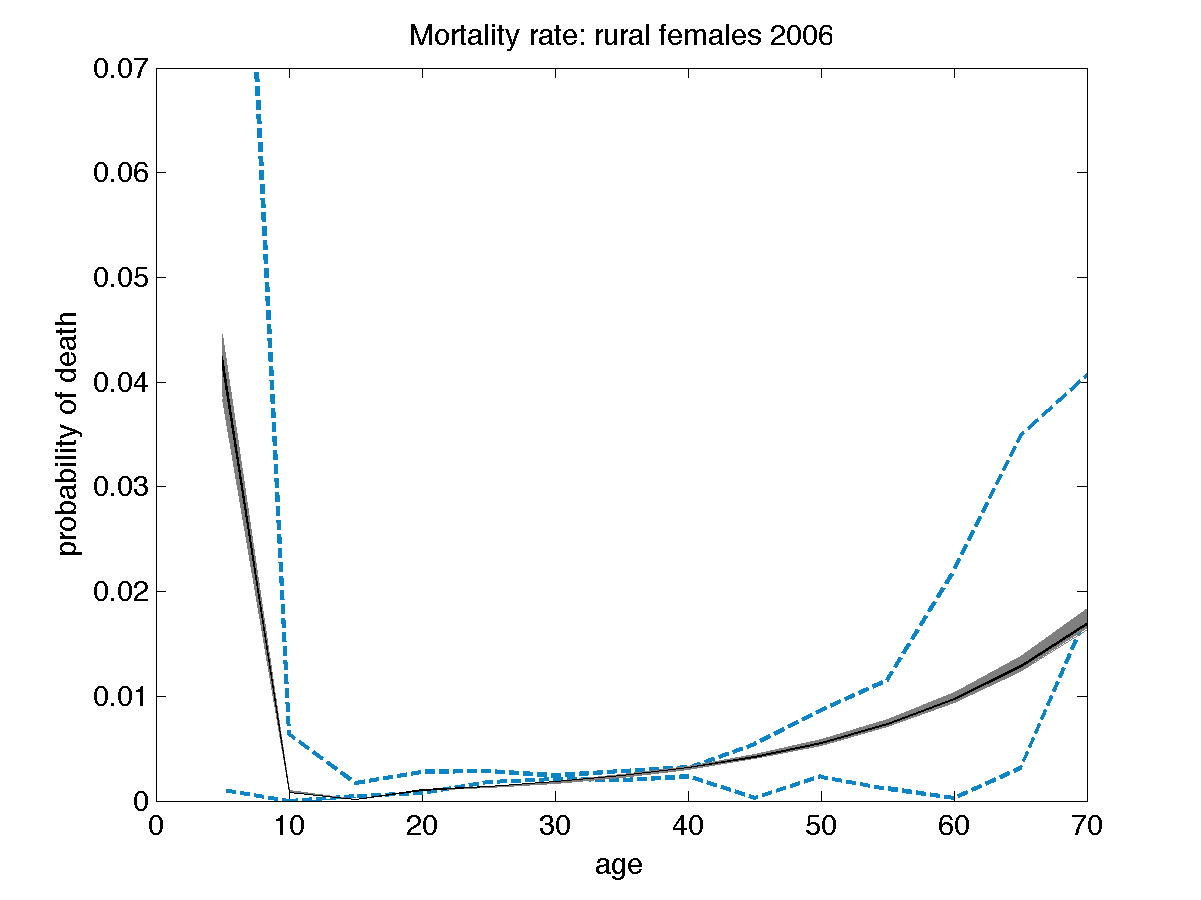


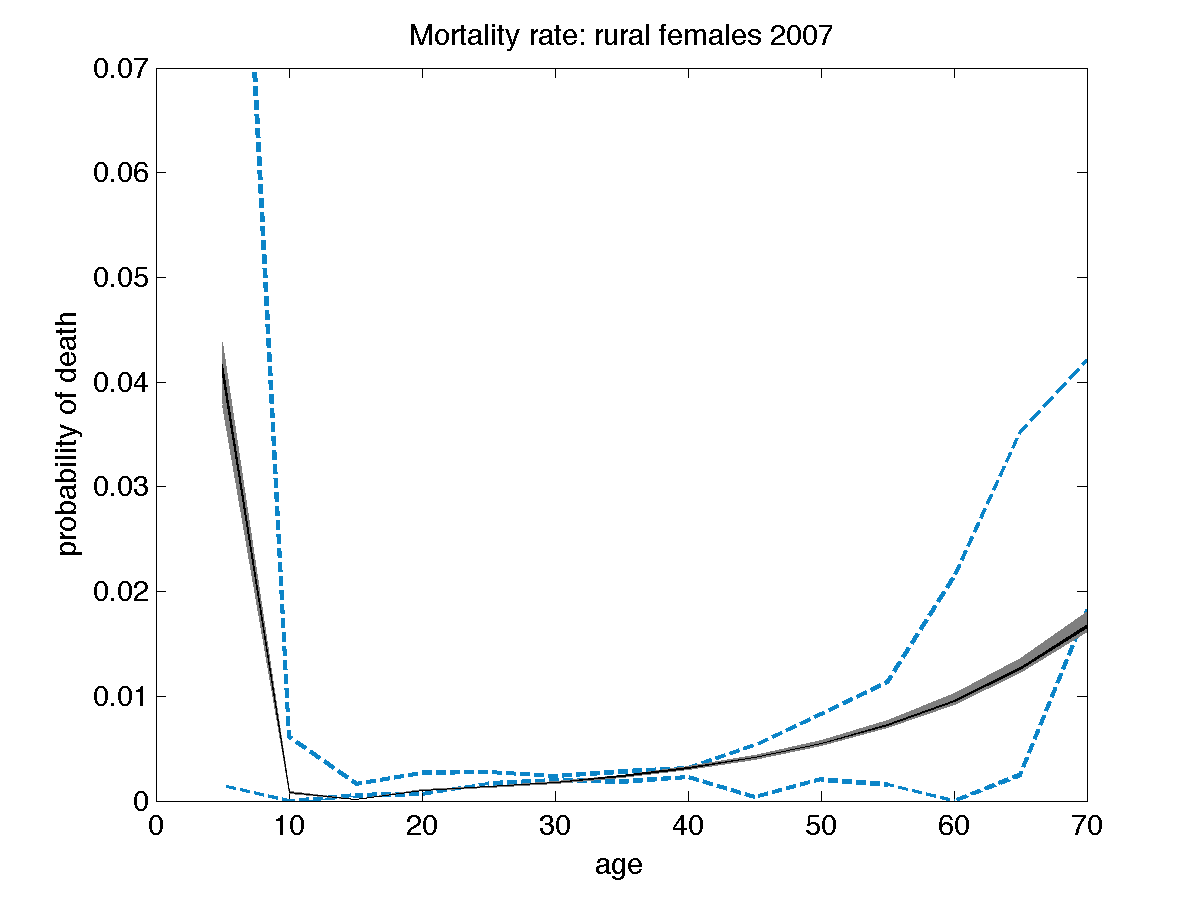


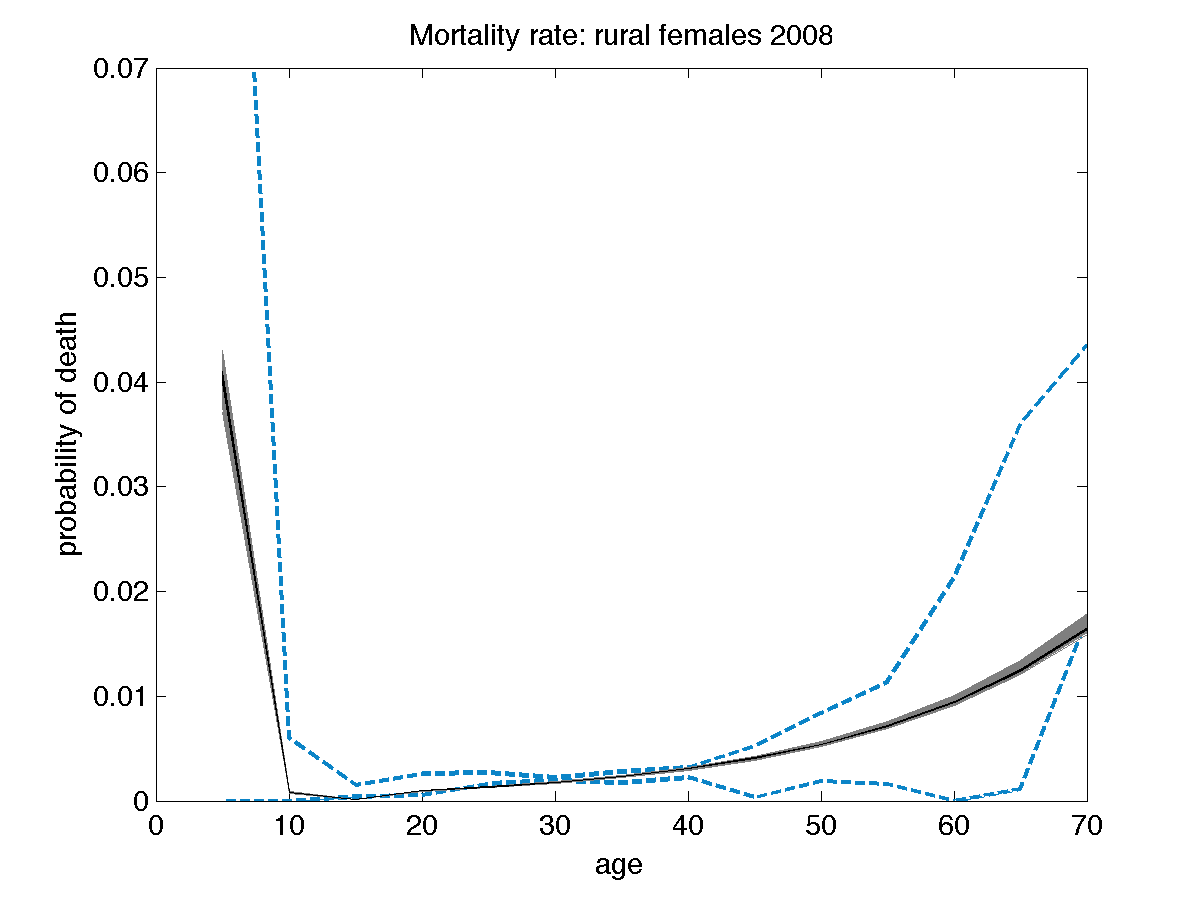


## 17 Figure AF4: Model fits to migration data

Migration data are provided in Table AF4. Educational categories are: 0: none; 1: 1 to 6 years; 2: >6 to 12 years; 3: >12 years. In all plots, gray shaded areas are results of 10,000 repeated samples from the posterior joint distribution of the fitted model (Figure 1C), with samples from the interquartile range as black lines and data displayed as dashed blue lines or circles reflecting the 95% confidence intervals of the input datasets.


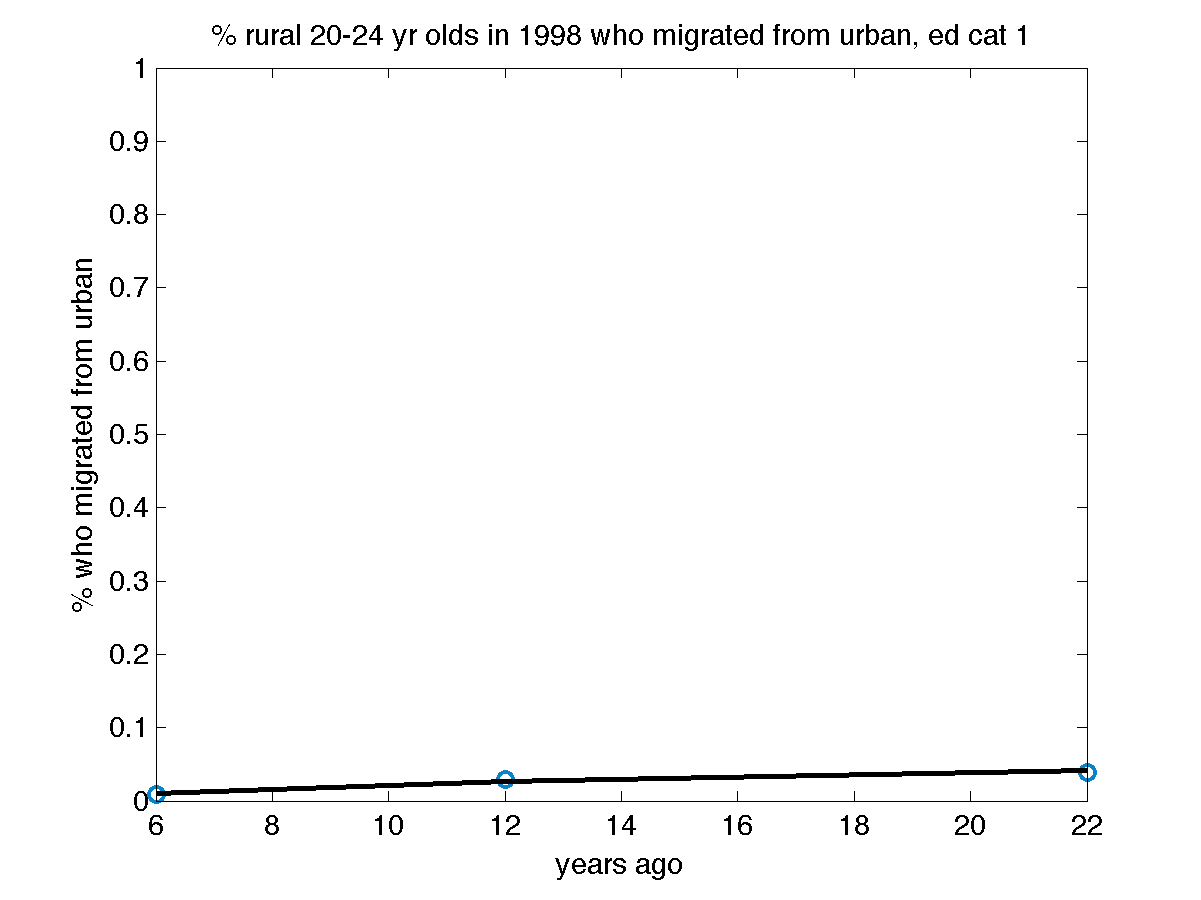


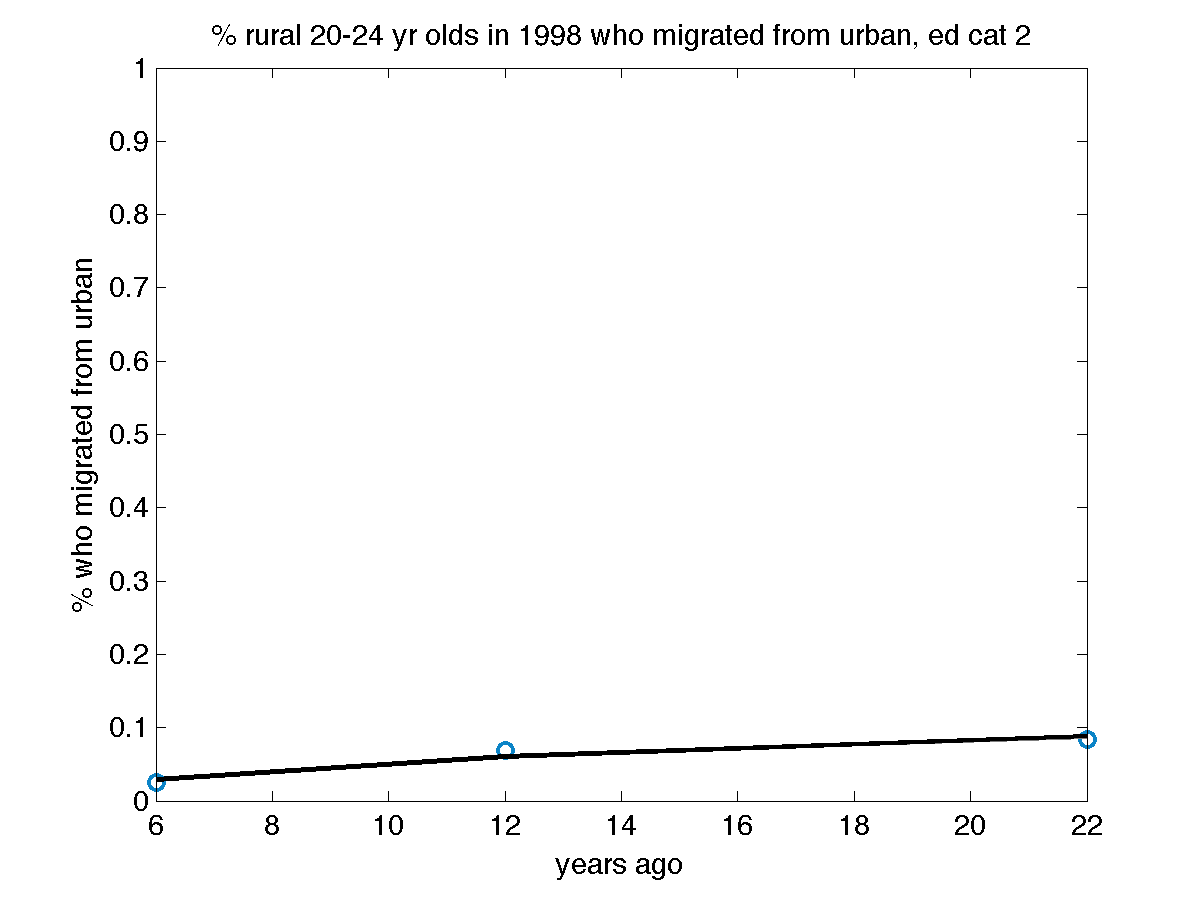


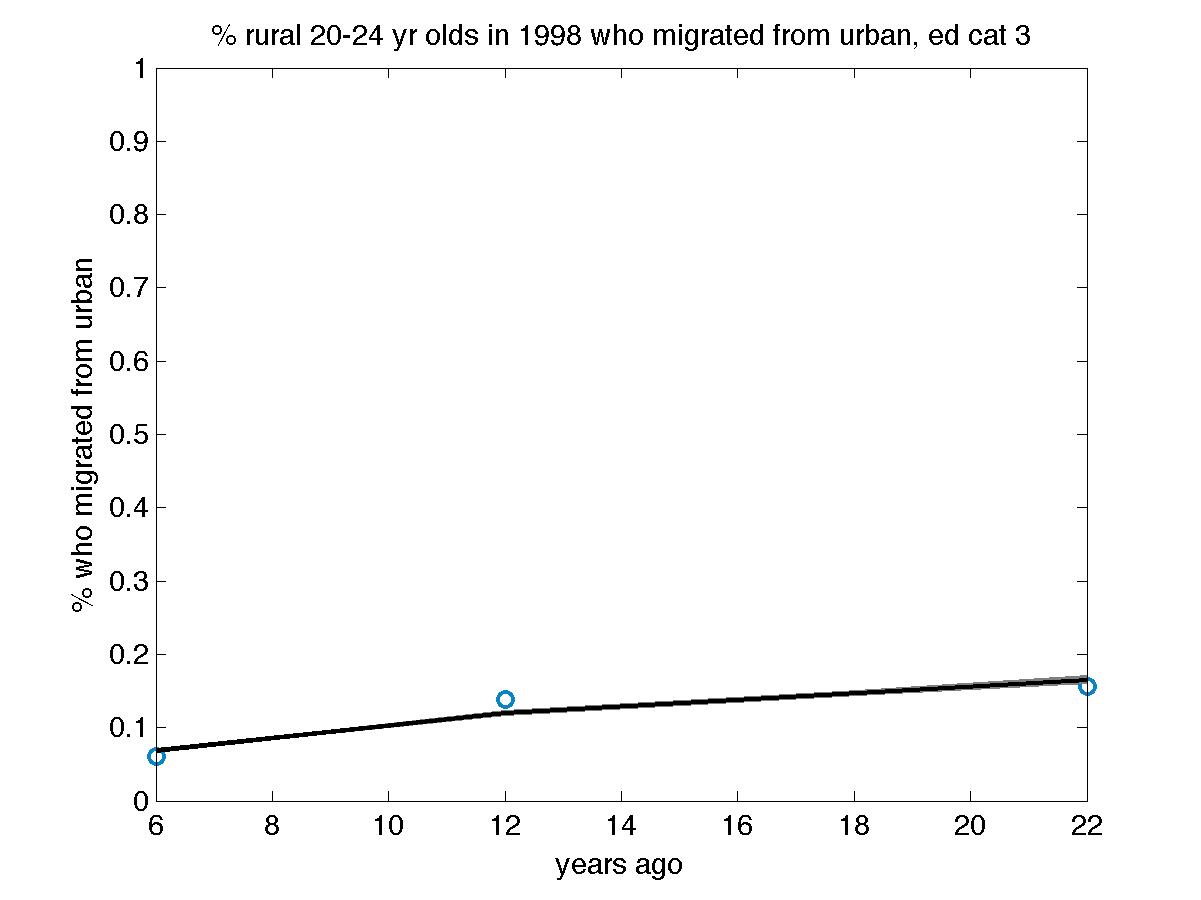


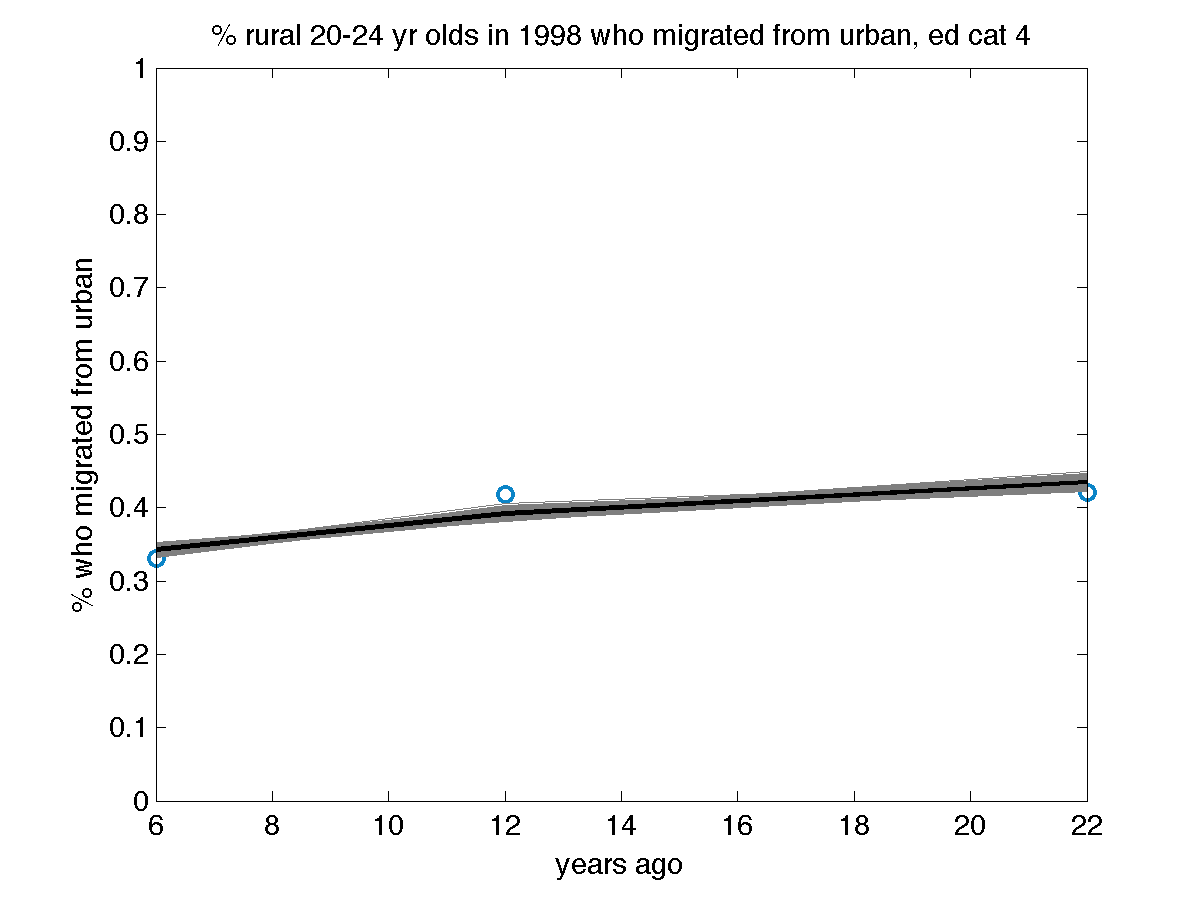


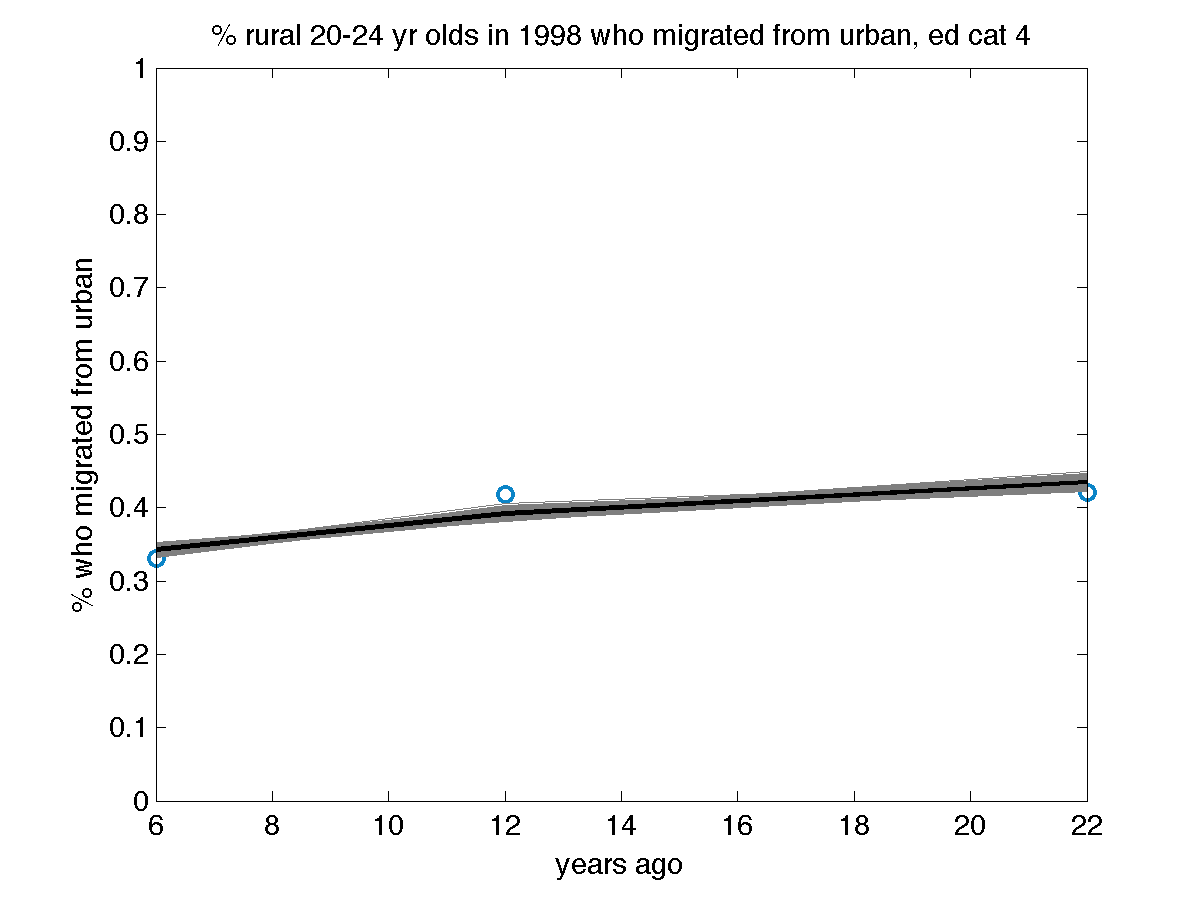


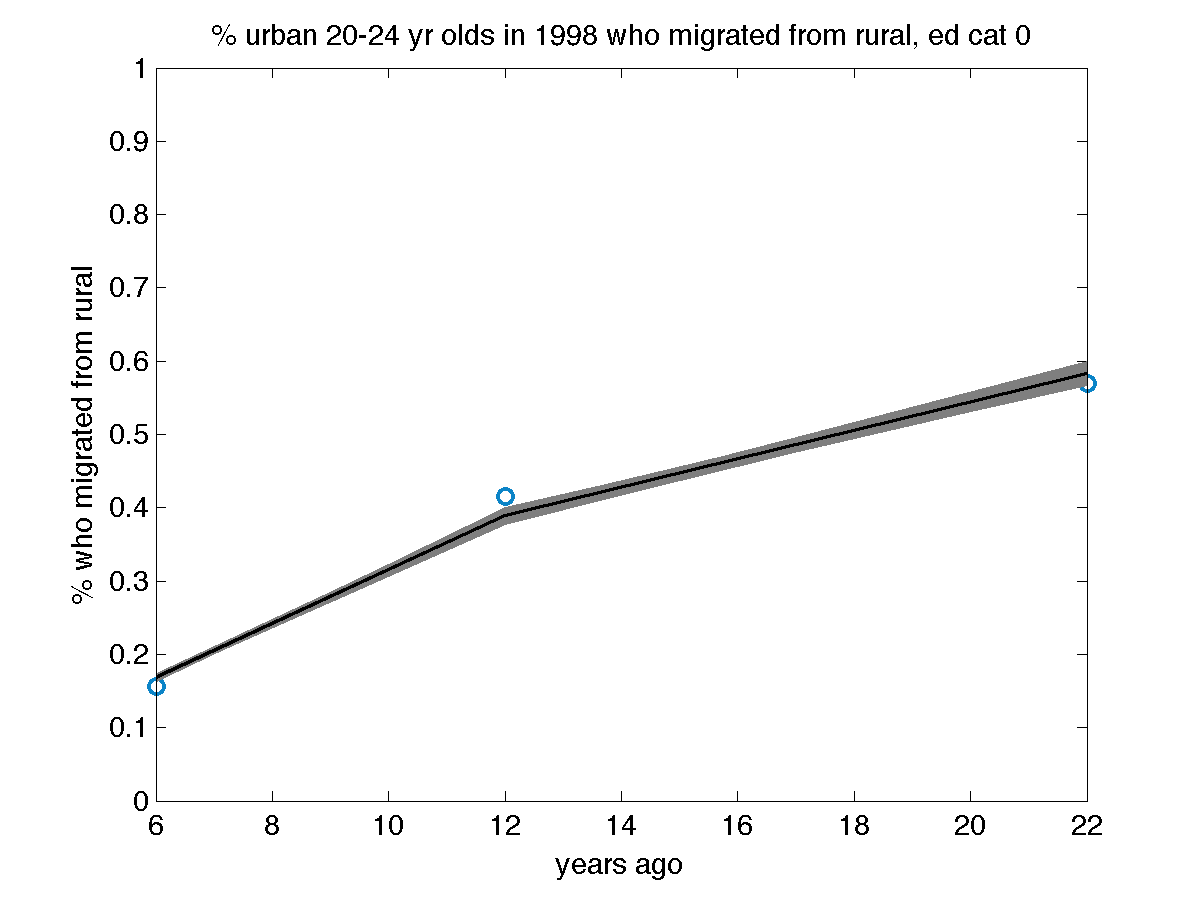


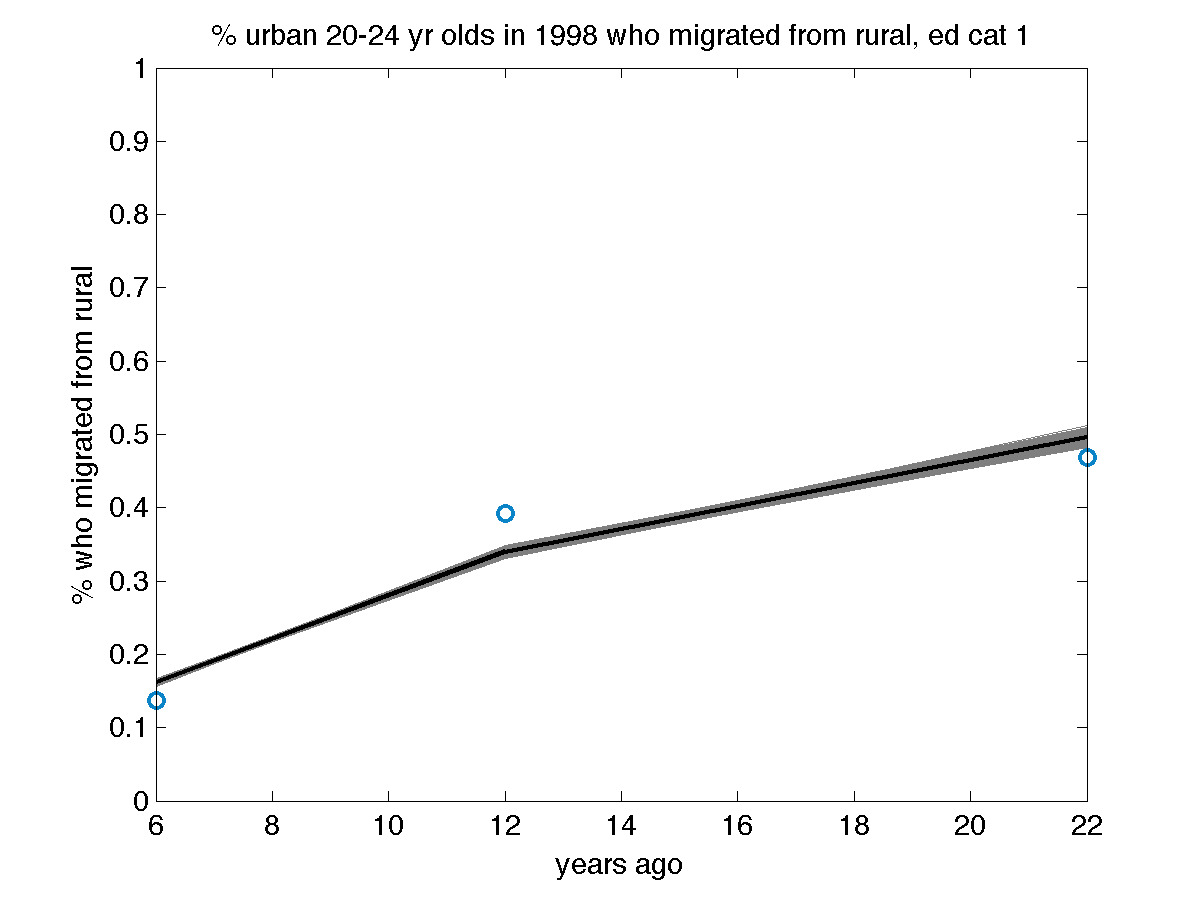


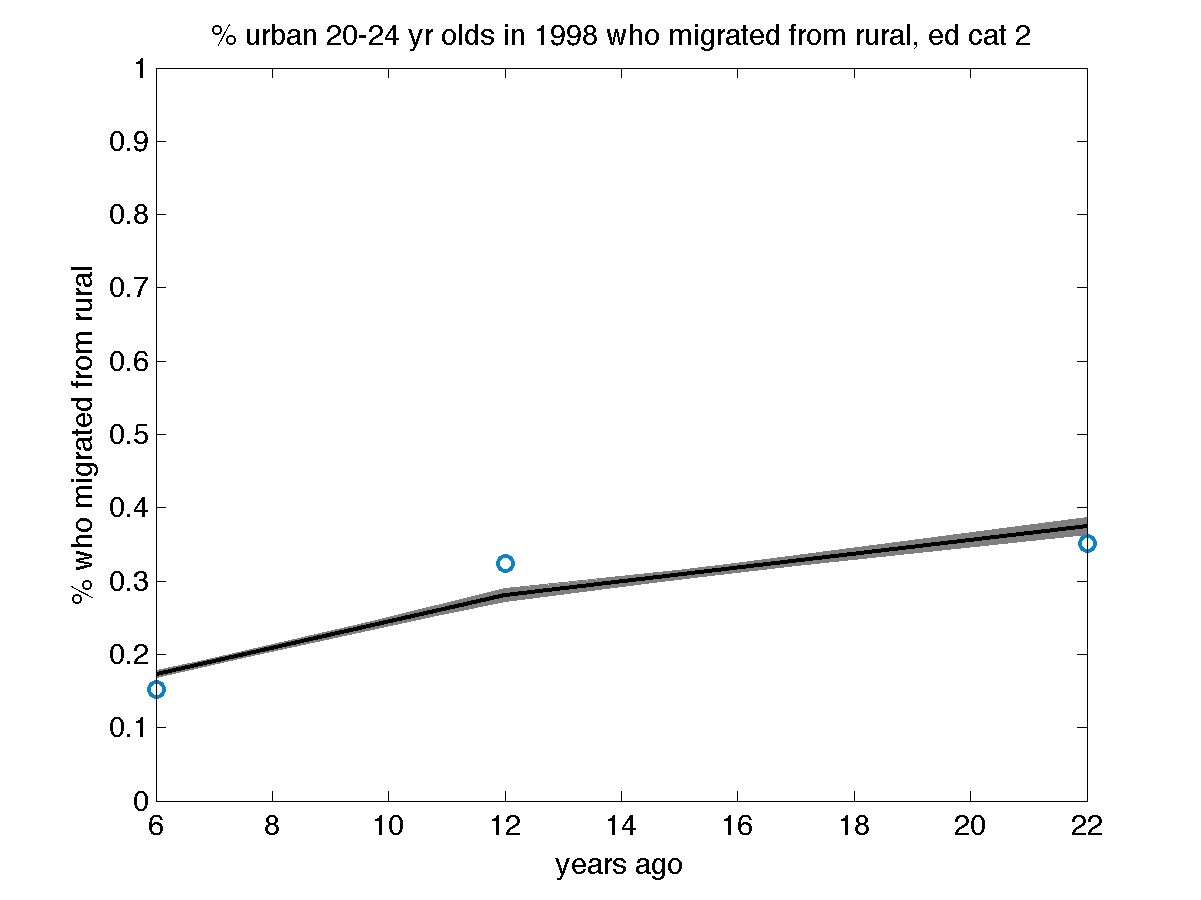


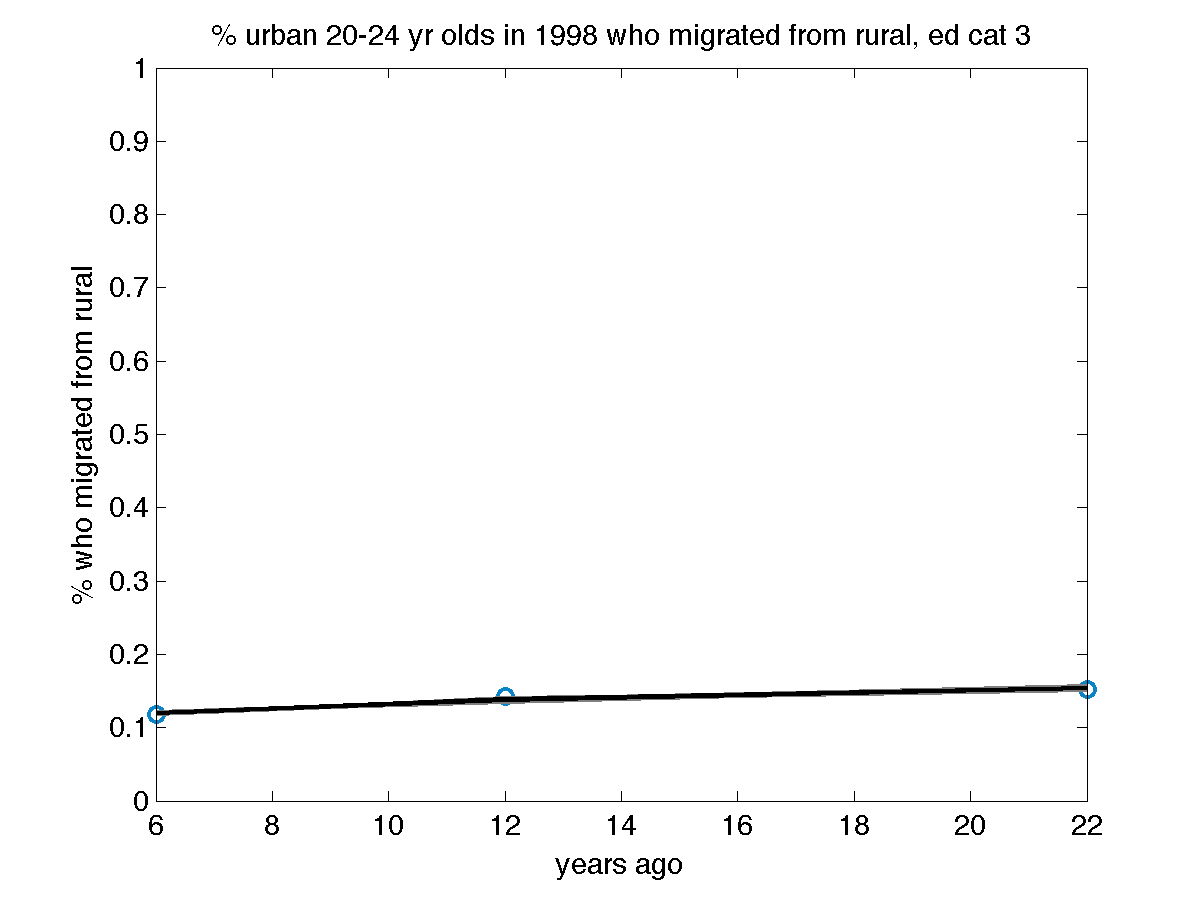


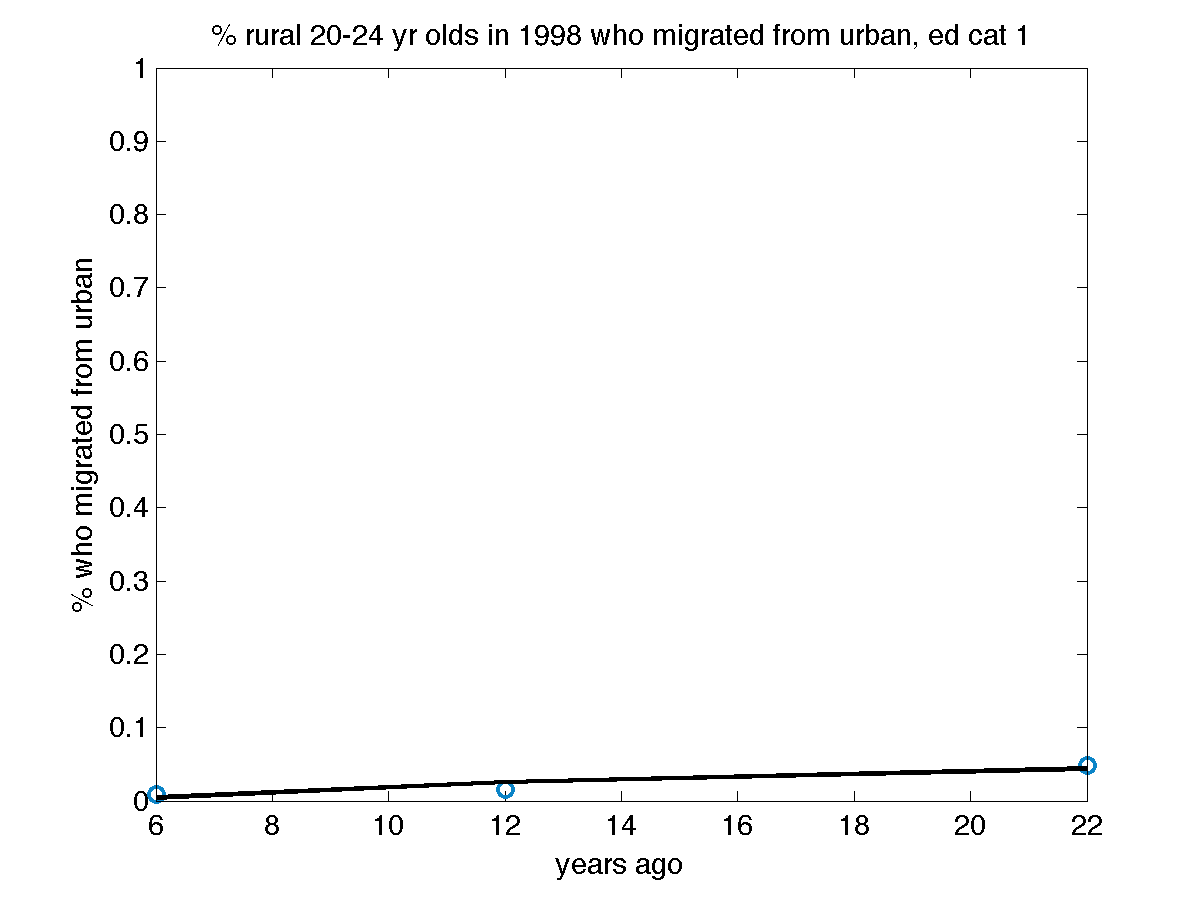


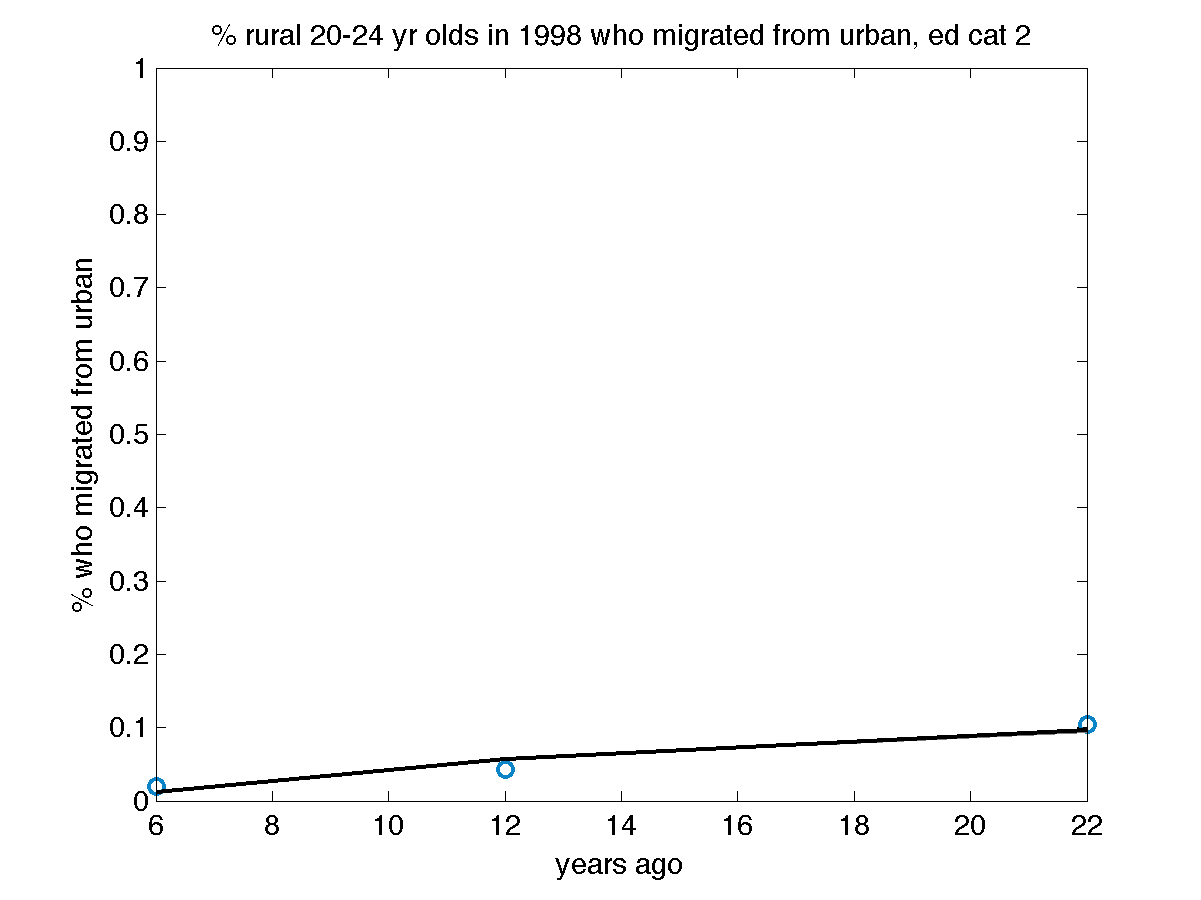


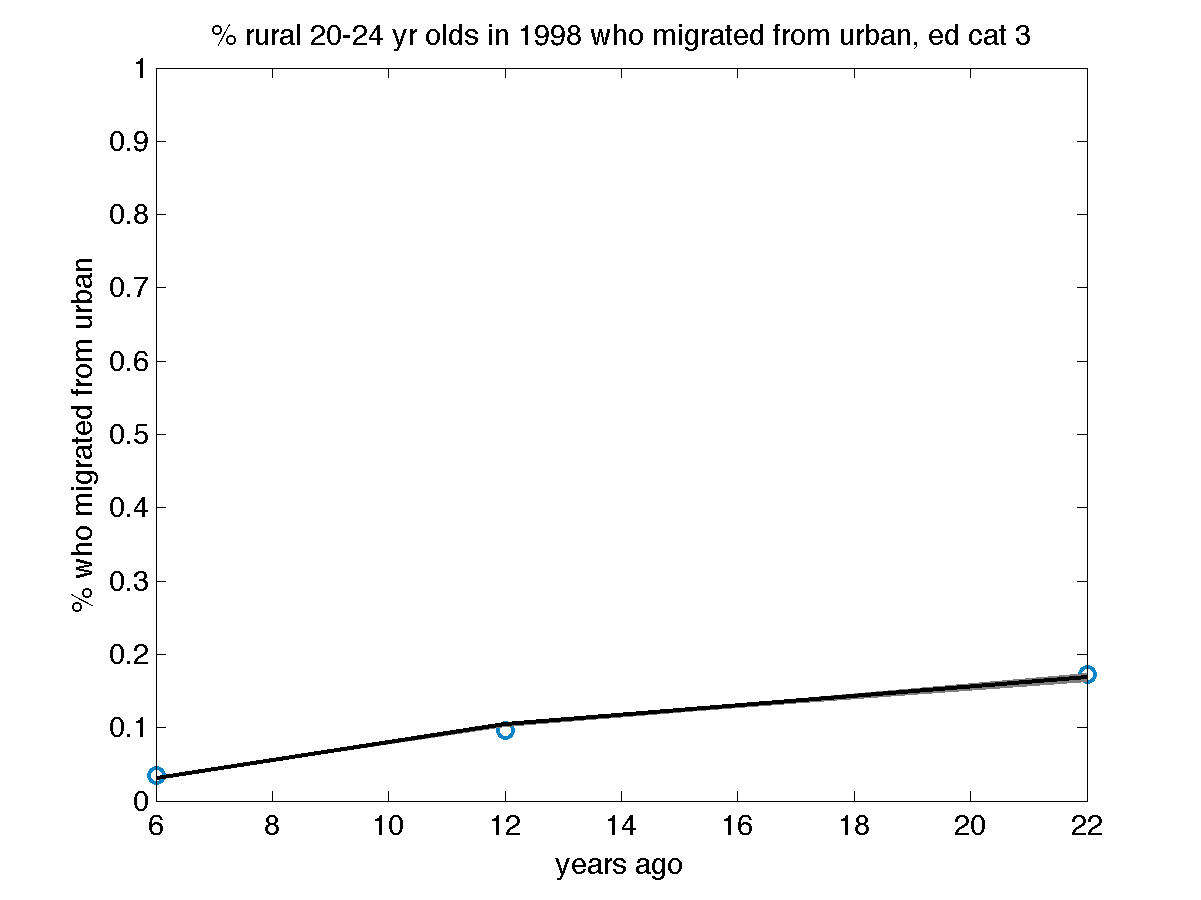


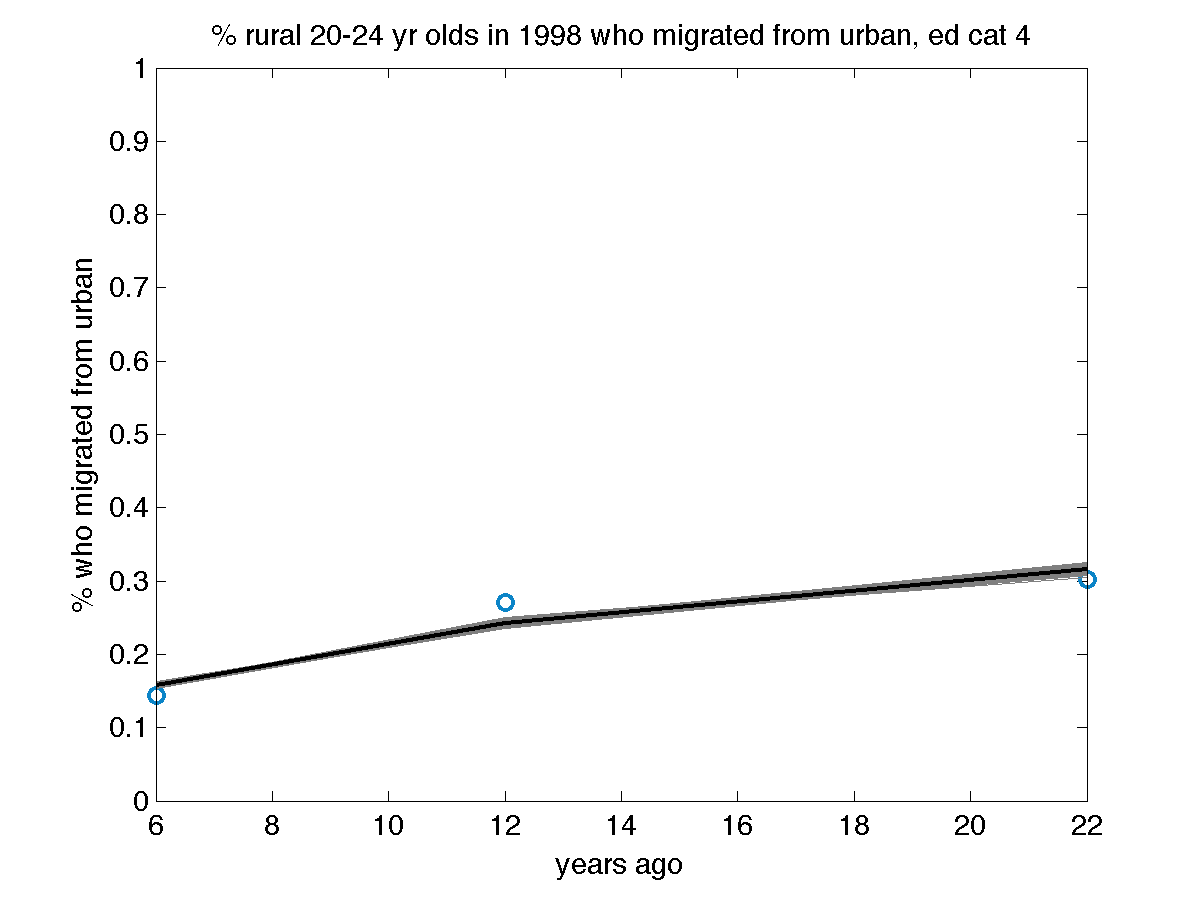


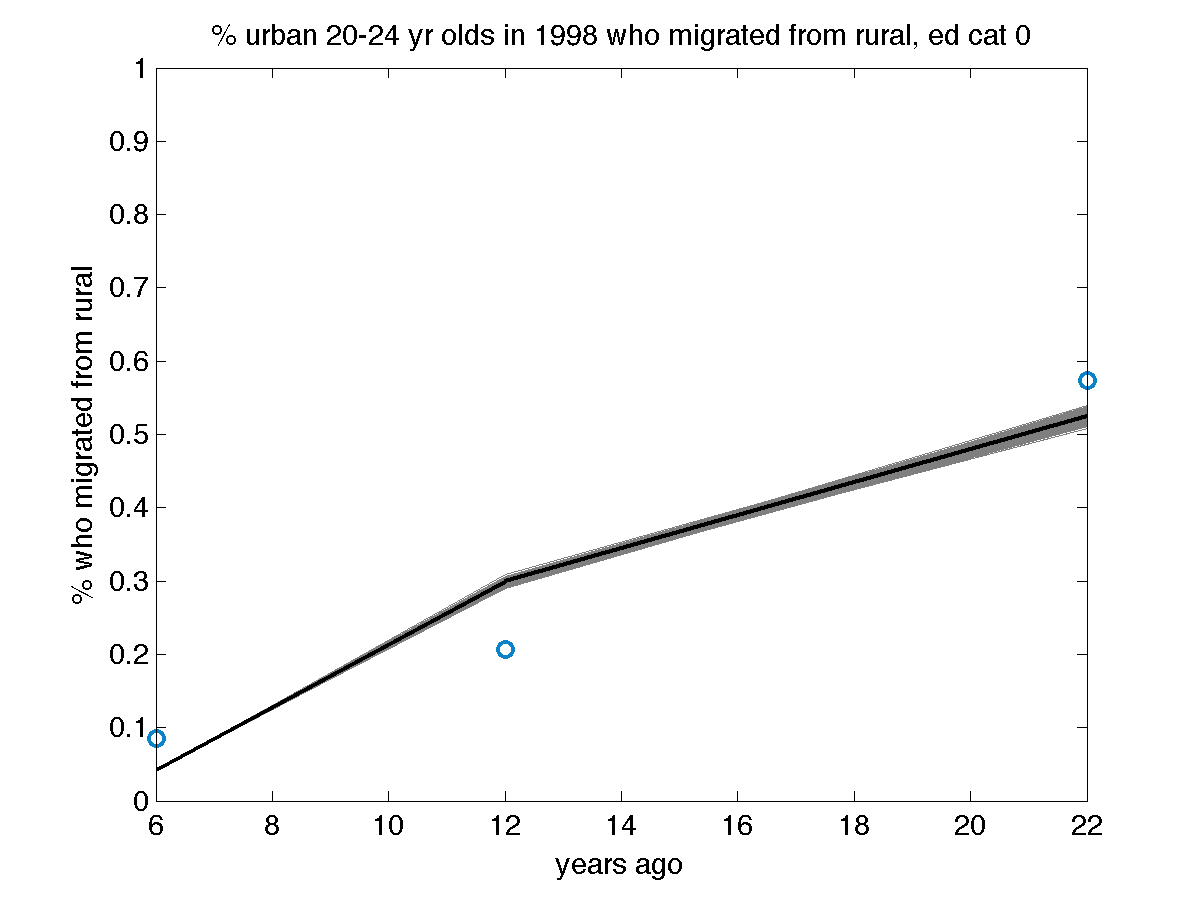


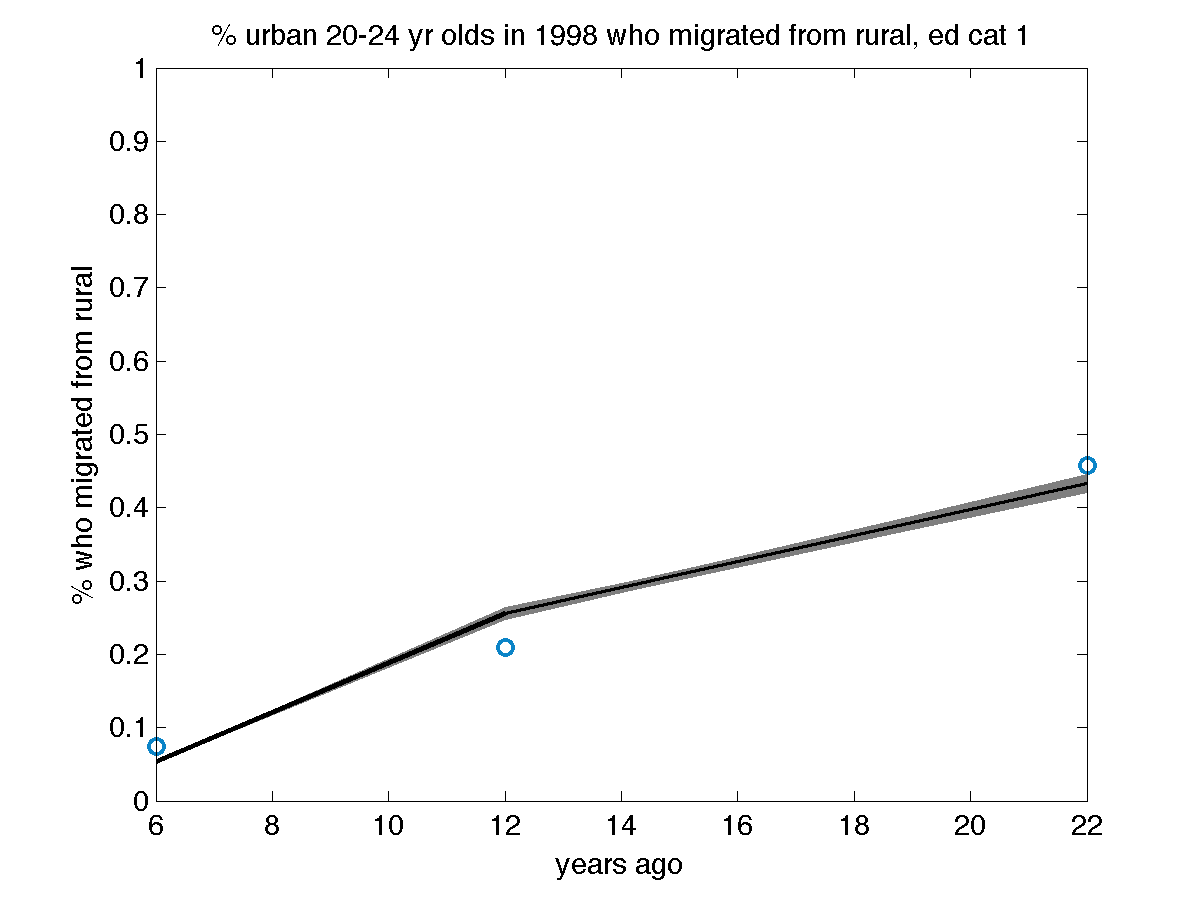


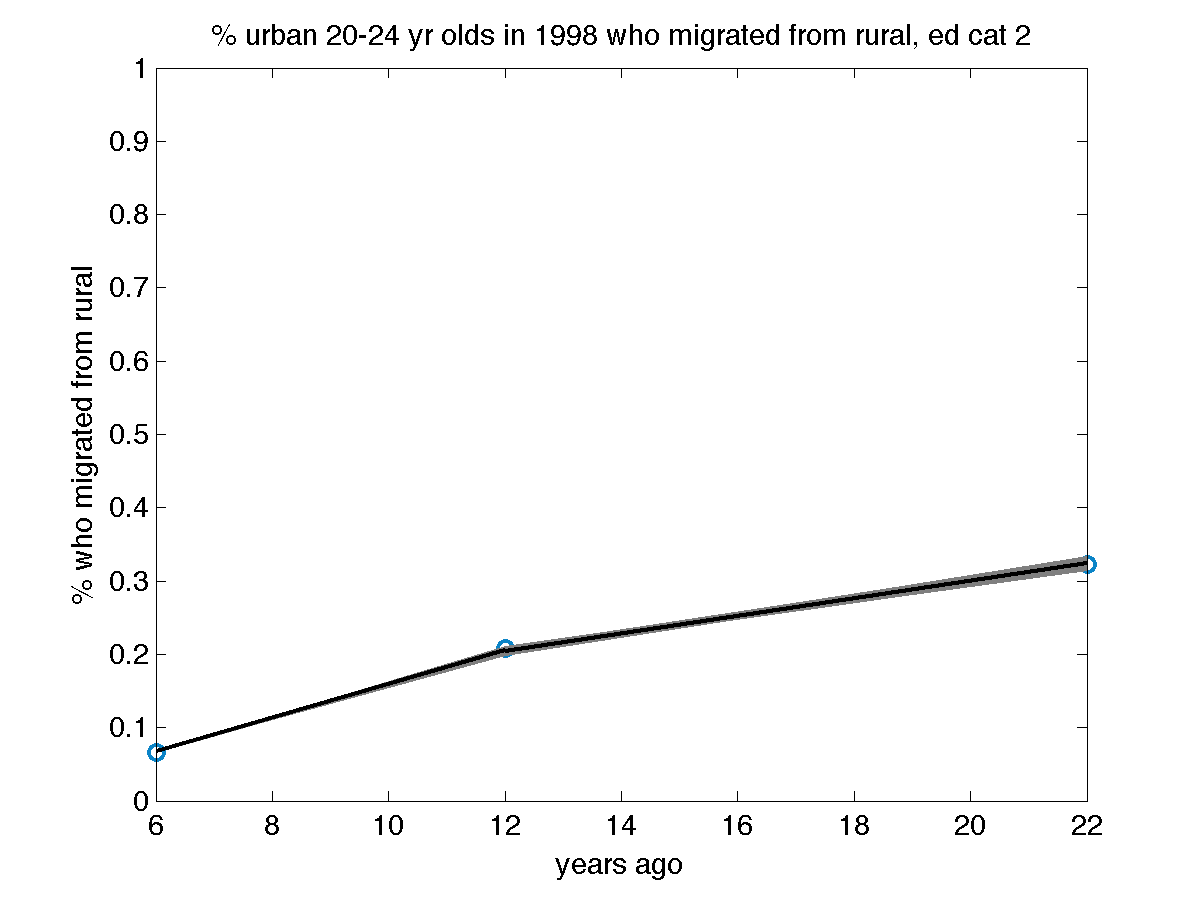


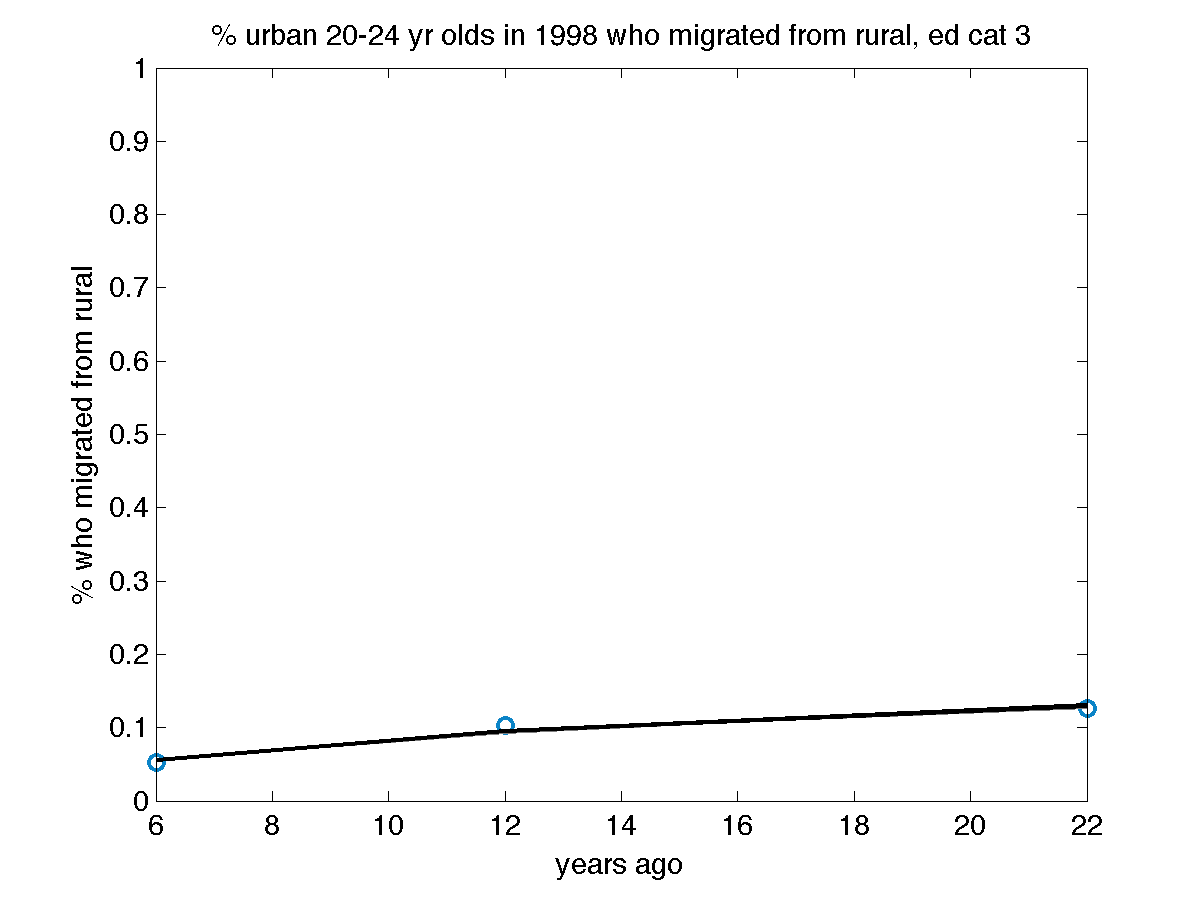


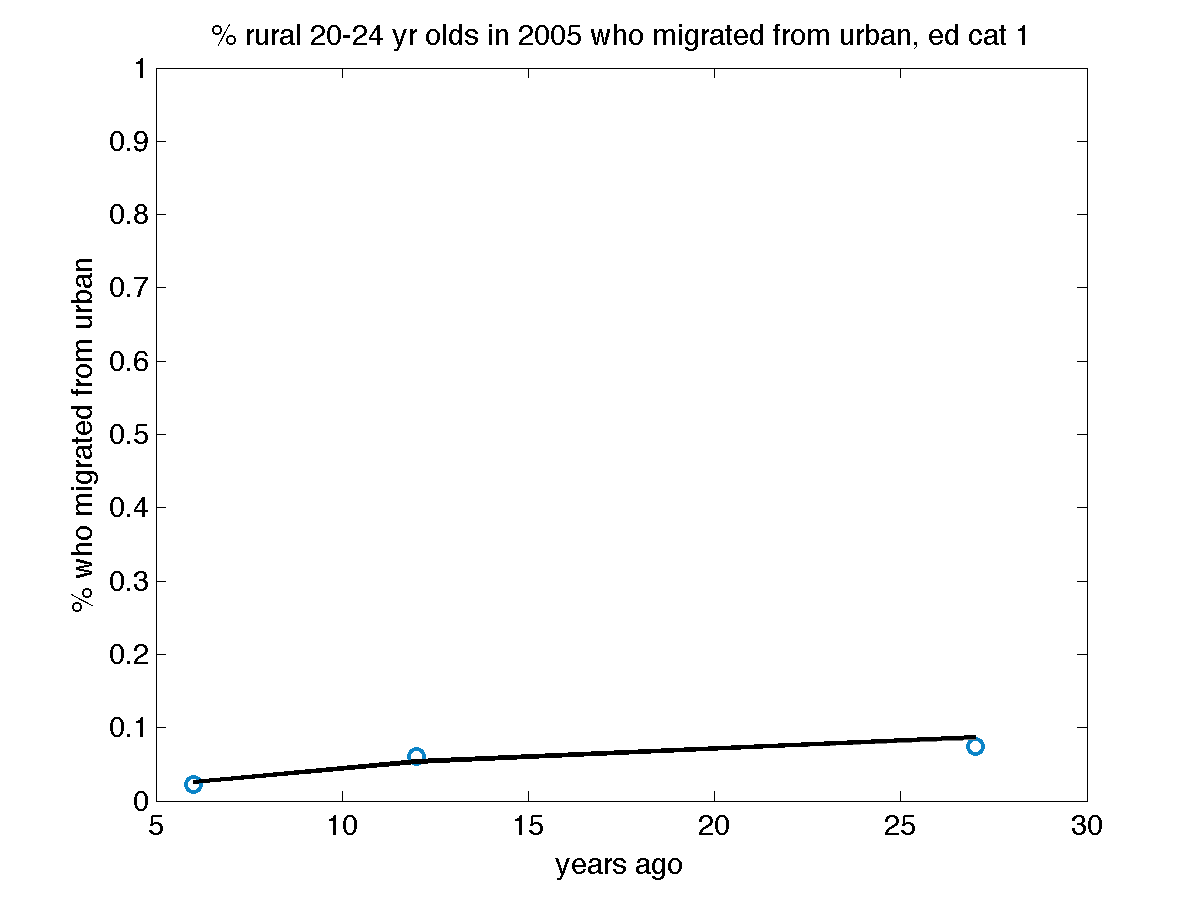


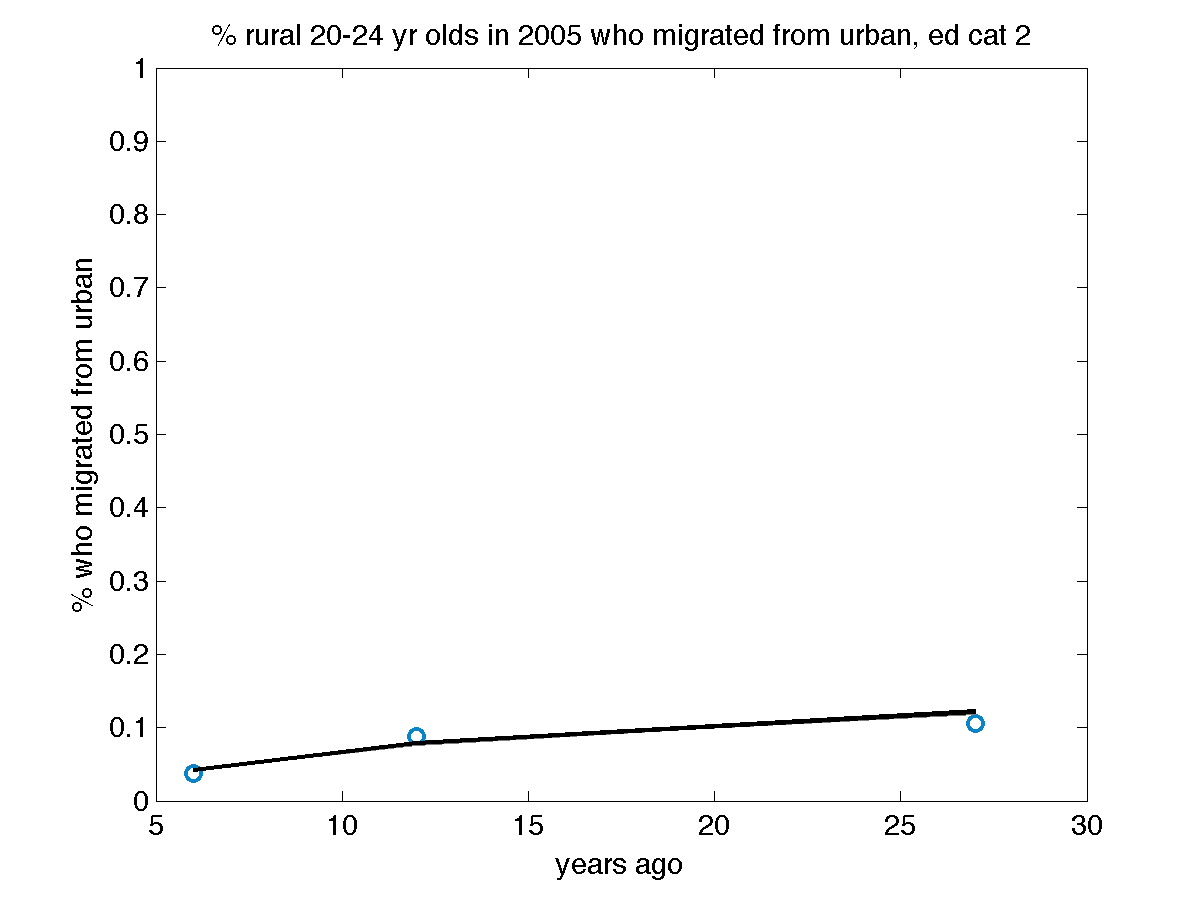


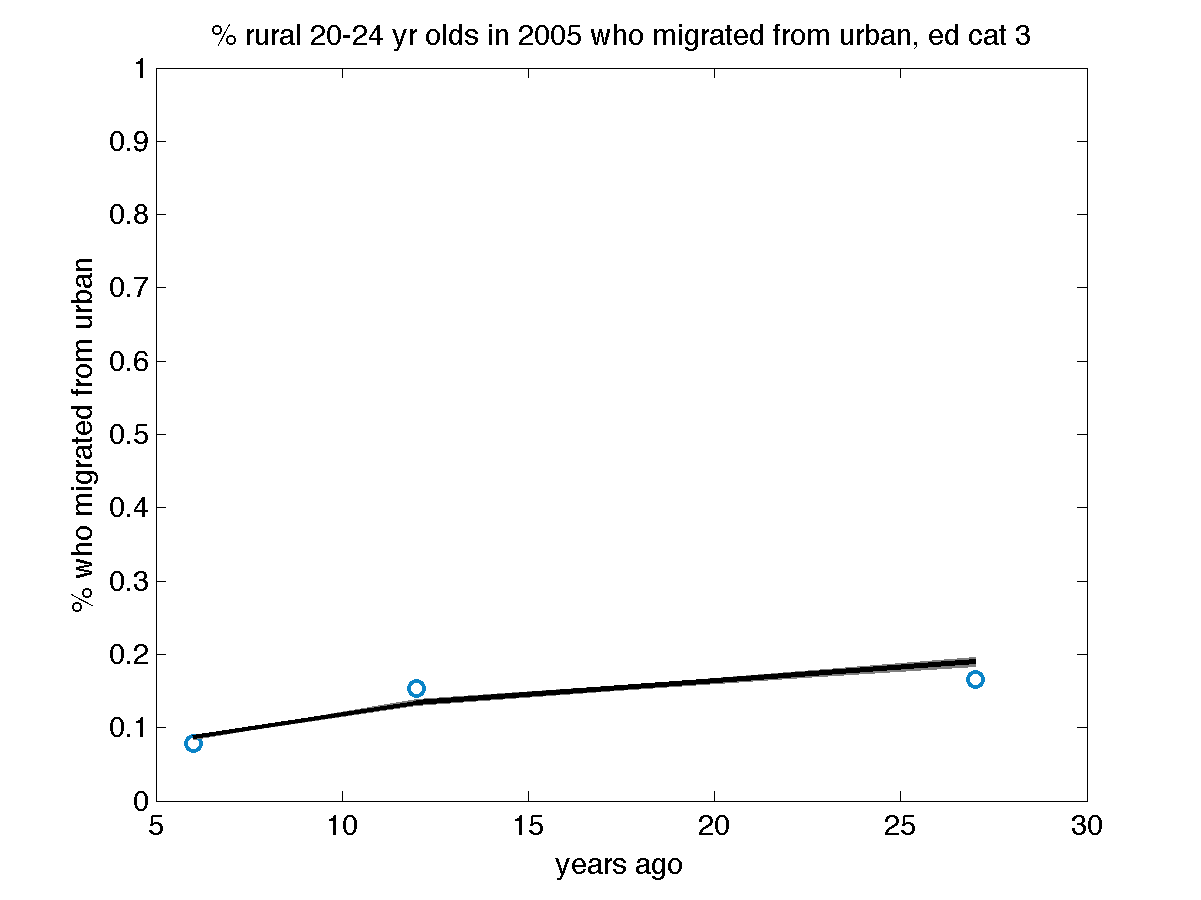


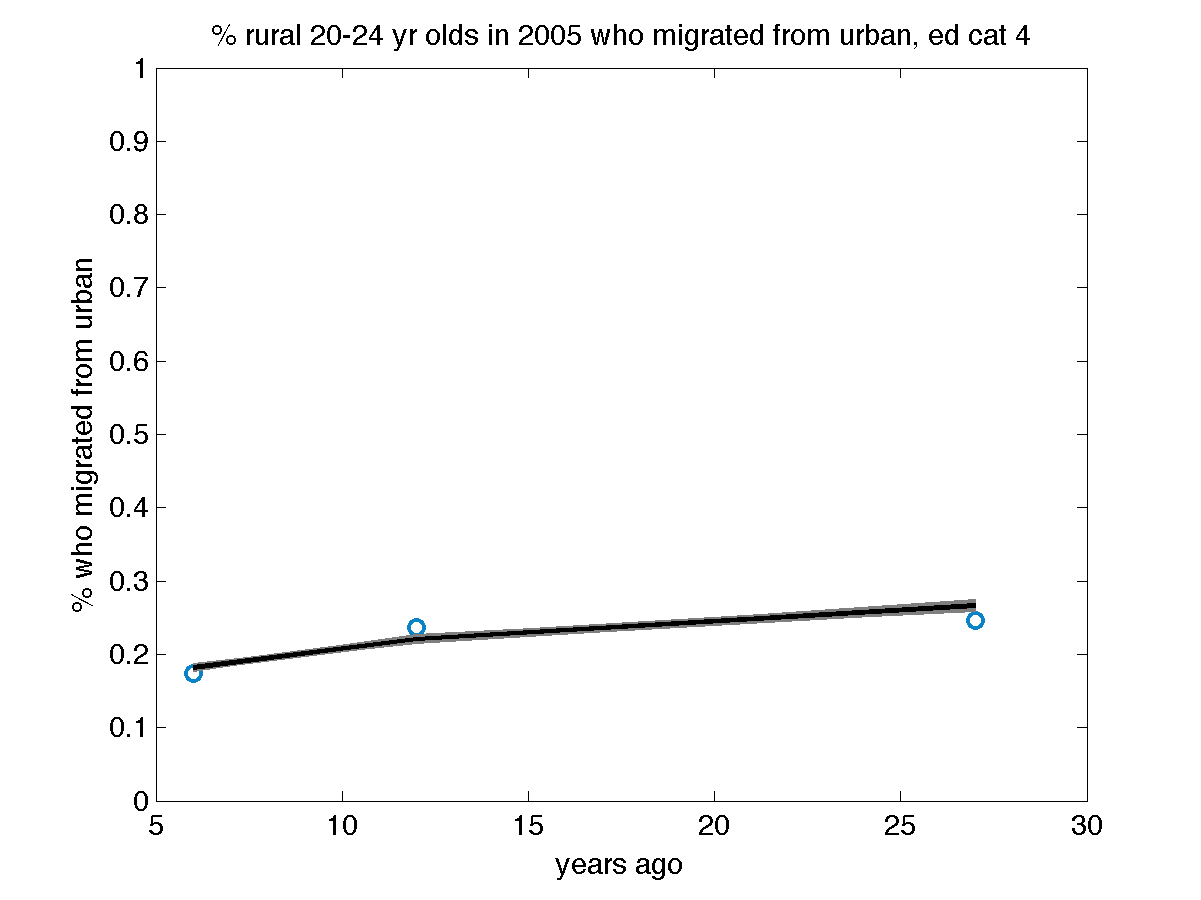


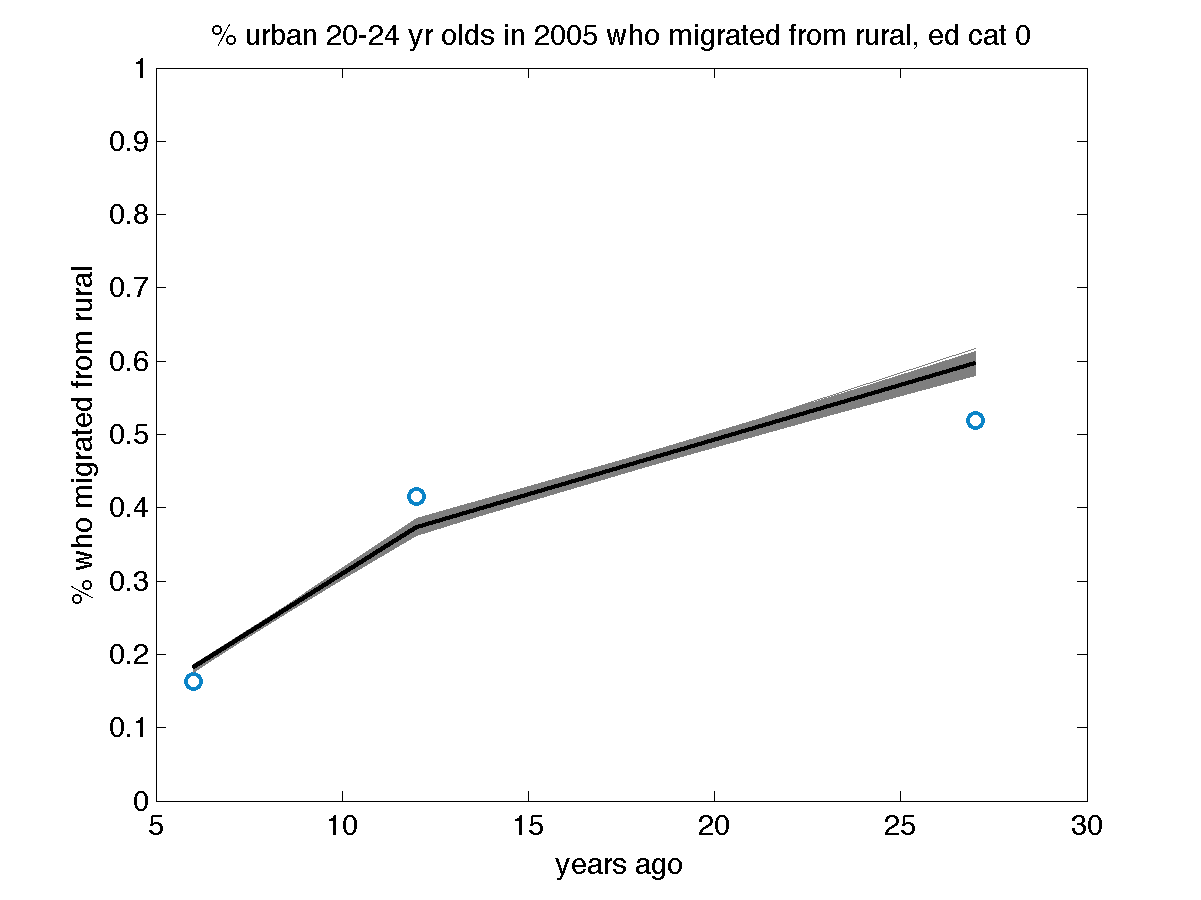


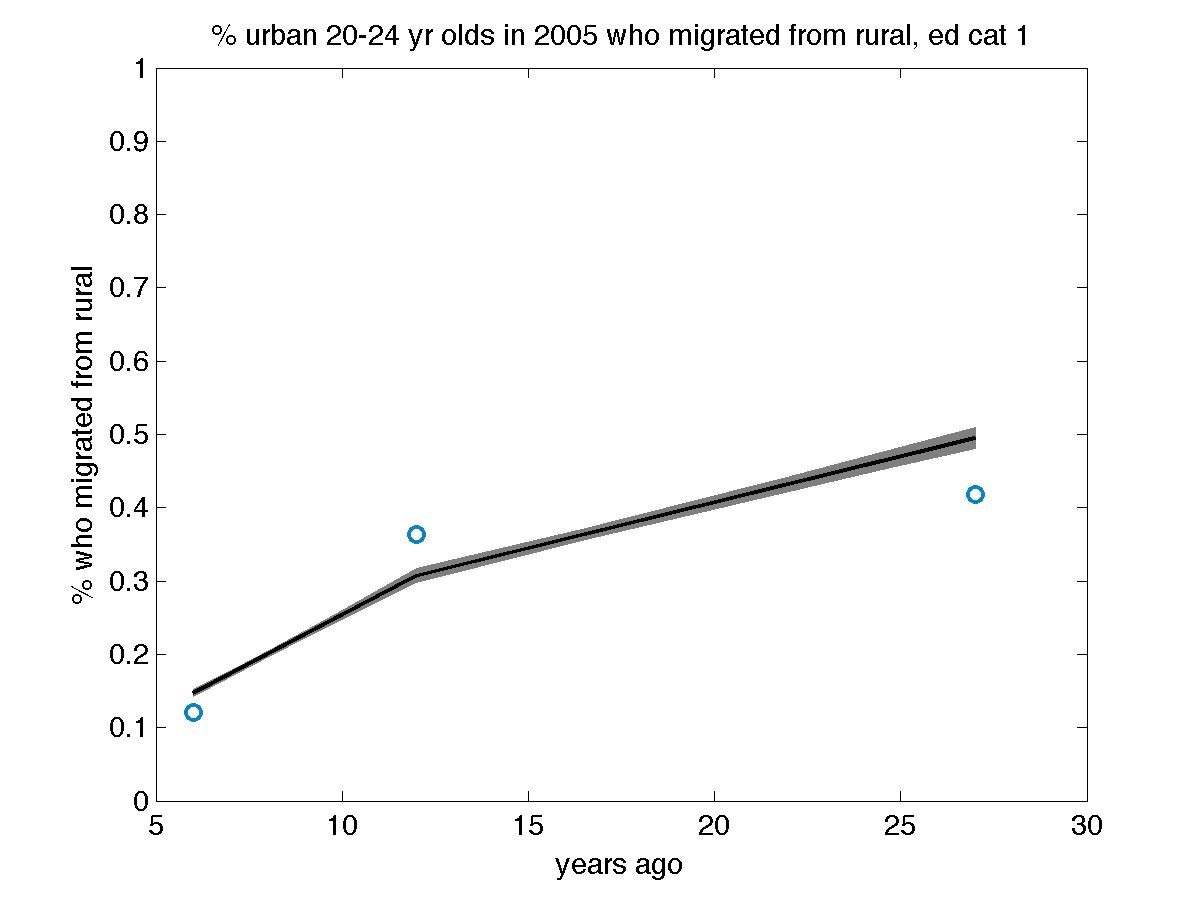


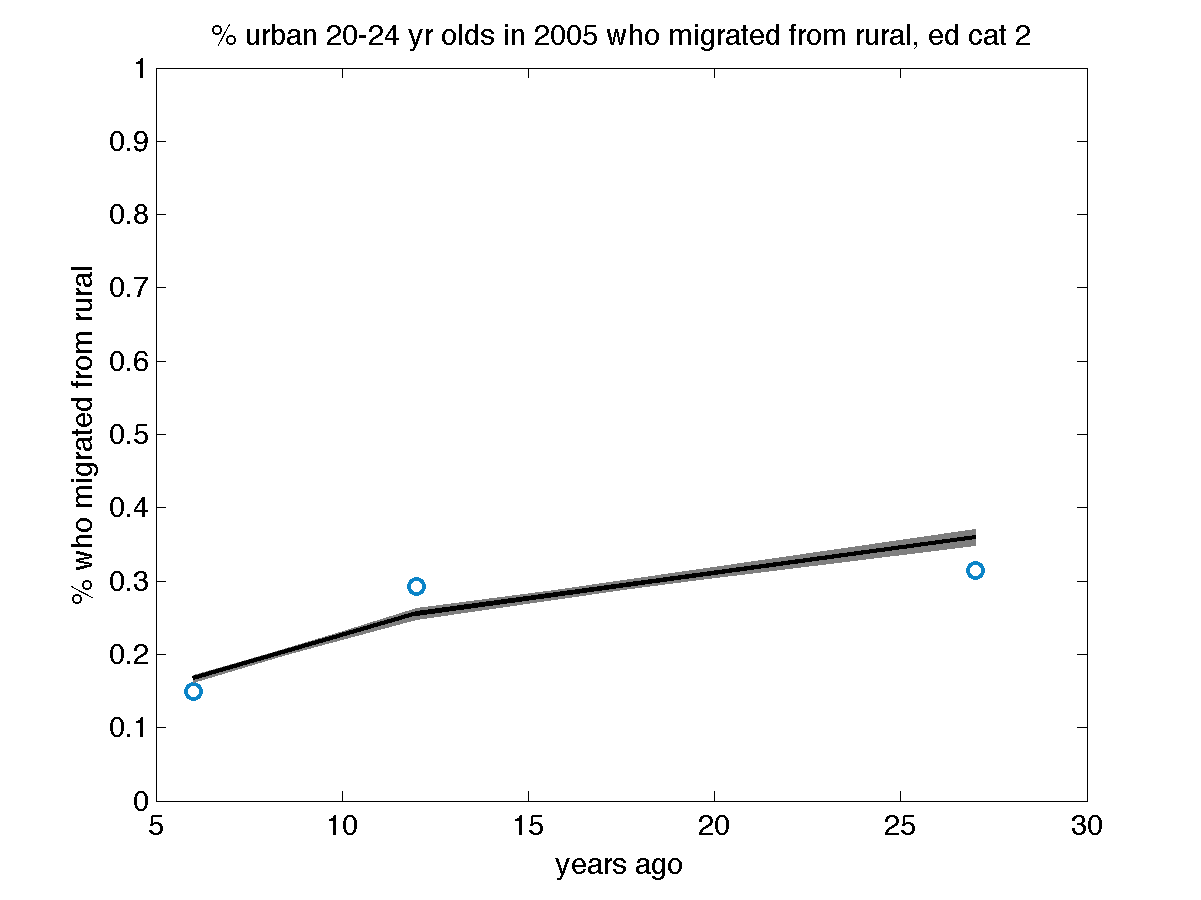


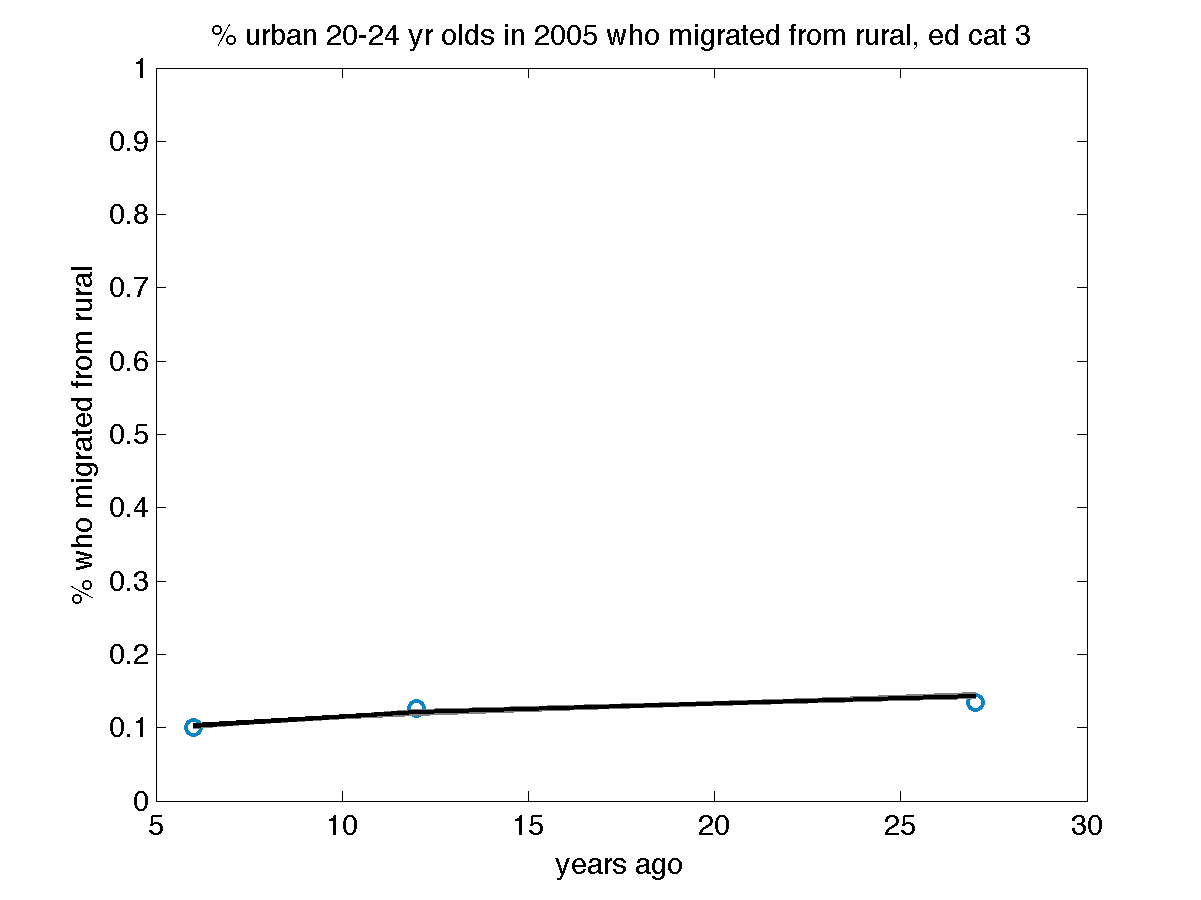


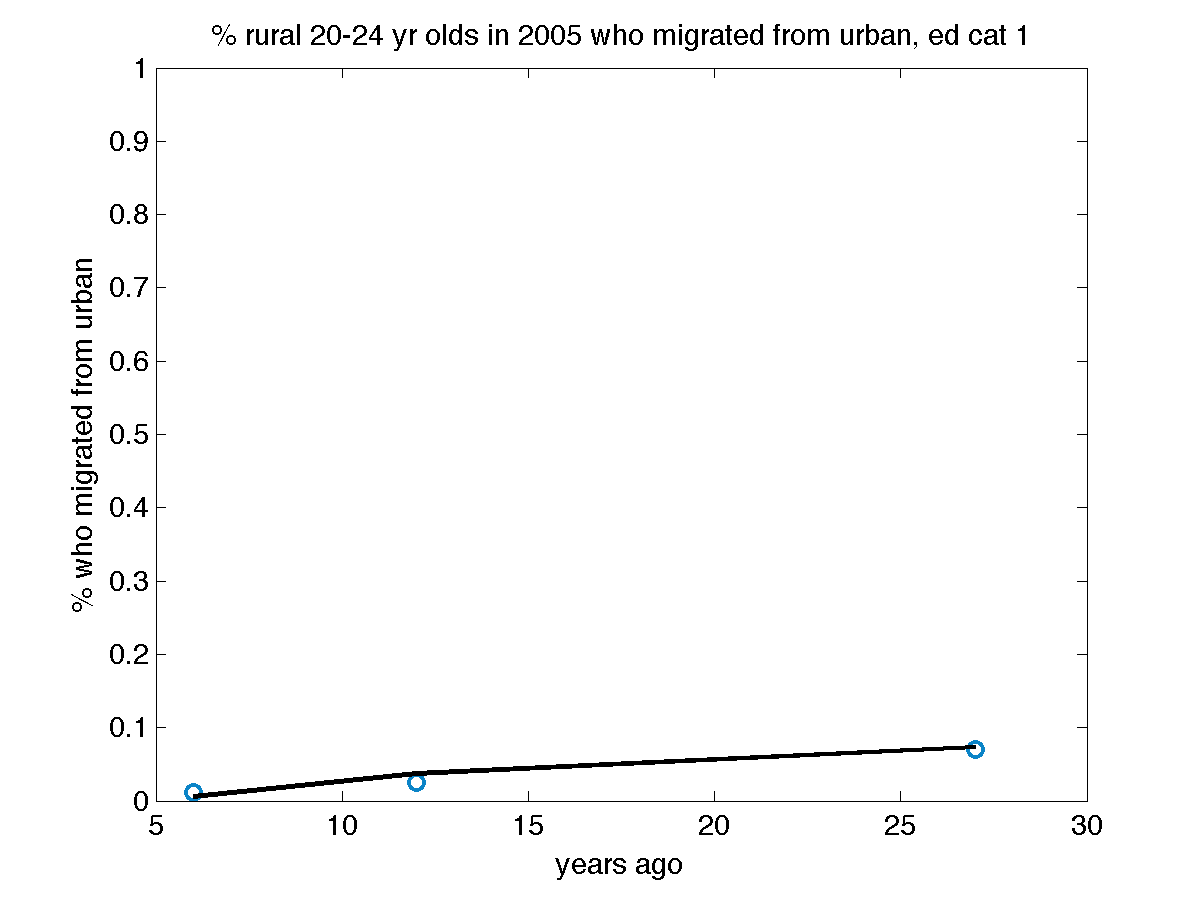


## 18 Figure AF5: Model fits to education data

Education data are provided in Table AF3. Educational categories are: 0: none; 1: 1 to 6 years; 2: >6 to 12 years; 3: >12 years. In all plots, gray shaded areas are results of 10,000 repeated samples from the posterior joint distribution of the fitted model (Figure 1C), with samples from the interquartile range as black lines and data displayed as dashed blue lines or circles reflecting the 95% confidence intervals of the input datasets. Note that educational attainment data from the first three years (1992, 1998, and 2005) are from the National Family Health Surveys [5–7], while for the final year (2008) the data are from the District Level Household Survey, which has slightly different sampling methodology but it also intended to be nationally-representative [9].

## 19 Figure AF6: Model fits to life expectancy data

Model-predicted life expectancy validated against independent estimated life expectancy (external validation) (World Bank 2014). Gray lines reflect results of 10,000 repeated samples from the posterior joint distribution of the fitted model (Figure 1C), black lines refer to the samples from the interquartile range of the probability distributions, and blue circles reflect data and its 95% confidence intervals (diameter of circles).
